# Cost-effective targets for anaemia reduction in 191 countries: a modelling study

**DOI:** 10.1016/S2352-3026(25)00168-1

**Published:** 2025-08-26

**Authors:** Robin Blythe, Natalie Carvalho, Jacinta Holloway-Brown, Sumie Leung, Victoria L Oliver, Yingying Wang, Clare Glover-Wright, Sant-Rayn Pasricha, Michael Bode

**Affiliations:** a Health Services and Systems Research, Duke–NUS Medical School, Singapore; b School of Mathematical Sciences, Queensland University of Technology, Brisbane, QLD, Australia; c Melbourne School of Population and Global Health, University of Melbourne, Melbourne, VIC, Australia; d School of Computer and Mathematical Sciences, University of Adelaide, Adelaide, SA, Australia; e Walter and Eliza Hall Institute of Medical Research, Melbourne, VIC, Australia; f Department of Clinical Haematology, Peter MacCallum Cancer Centre and The Royal Melbourne Hospital, Melbourne, VIC, Australia; g Department of Medical Biology, University of Melbourne, Parkville, VIC, Australia; h Securing Antarctica's Environmental Future, Queensland University of Technology, Brisbane, QLD, Australia

## Abstract

**Background:**

Anaemia causes widespread health and economic harm. Current international targets for reducing anaemia in women of reproductive age, including the Sustainable Development Goal of halving prevalence by 2030, are unlikely to be met by any signatory country. This outcome suggests that current targets were grounded in aspiration rather than a systematic assessment of what is achievable given current recommended interventions and national health-care priorities. We propose a novel method of target setting for global health goals, with reducing anaemia in women of reproductive age as an example.

**Methods:**

In this modelling study, we developed a country-level health economic model to support feasible and ambitious target-setting for anaemia for women of reproductive age (aged 15–49 years) based on cost-effectiveness analysis and applied it to 191 signatory countries. Our model integrated country-specific data on the current prevalence of anaemia, the effectiveness and current and maximal coverage of recommended interventions available to each country, the local unit costs of these interventions, and country-specific cost-effectiveness threshold estimates, including Global Burden of Disease data and data from the Demographic and Health Survey Program. Interventions were applied to maximise health gains subject to country-level cost-effectiveness thresholds at 1 × gross domestic product per capita. We assessed parameter uncertainty through Monte Carlo simulations and scenarios that considered alternative thresholds, constraints on cost, and coverage.

**Findings:**

Our results indicate that an ambitious, achievable, and cost-effective global target for anaemia reduction in women aged 15–49 years by 2030 is 17% (95% uncertainty interval [UI] 5–34). The maximum achievable target removing all cost constraints is a 22% (11–36) reduction. No scenario approached the current 50% global Sustainable Development Goal reduction target, indicating that this goal is unachievable with existing recommended interventions. Reduction targets for individual countries ranged from 0% to 29%, with substantial variation both between and within regions and income groups.

**Interpretation:**

Our findings suggest that a value-based global target for anaemia reduction will be substantially lower than the existing international commitment. Value-based targets using evidence from available interventions and cost-effectiveness for what is achievable given countries' differing contexts can provide better incentives for progress and offer more realistic forecasts of human development.

**Funding:**

Gates Foundation.

## Introduction

**Figure 1:**
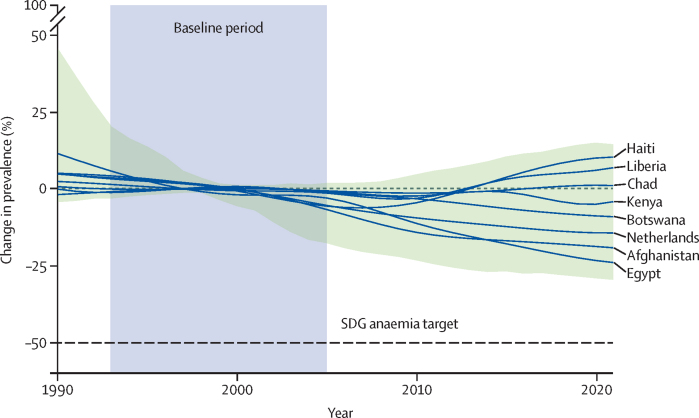
Global progress towards anaemia targets in women of reproductive age (ie, aged 15–49 years) between 1990 and 2021 Reproduced with permission from Atkinson et al.^6^ The shaded area encloses the estimated change in prevalence of anaemia in women of reproductive age, across all countries, as estimated by a Global Burden of Disease study.^7^ The dashed line shows the SDG and global nutrition target goal of a 50% reduction in prevalence. Individual lines show the trajectories of eight illustrative countries spanning a range of changes in anaemia prevalence over time. Values for countries are shown relative to their average prevalence of anaemia during 1993–2005, the baseline period used by the UN when deriving the SDG target indicator 2.2.3 for anaemia. SDG=Sustainable Development Goal.

In 2015, the UN Sustainable Development Goals (SDGs) committed signatory nations to halve the prevalence of anaemia in women of reproductive age by 2030 under SDG 2, zero hunger,^1^ reaffirming the pledges of the 2012 global nutrition targets.^2^, ^3^ Anaemia is a notable global health issue, disproportionately affecting women of reproductive age in low-income and middle-income countries (LMICs), and exacerbating health and economic inequalities.^2^, ^3^, ^4^ Effective diagnosis and treatment of anaemia are crucial to improving health outcomes.^2^ Substantial global efforts have been made to address anaemia, with 26 countries greatly reducing anaemia prevalence in women of reproductive age between 1990 and 2021.^4^ However, the population with anaemia has increased from 1·5 billion people in 1990 to 1·9 billion people in 2021.^5^ Although global prevalence decreased from 28% to 24% over this same period, no nation is projected to achieve the promised 50% reduction by the 2030 deadline (figure 1).^4^, ^8^

This projected outcome raises questions about how these international targets for anaemia were set and about global health target setting generally. If the purpose is to choose goals that are both ambitious and achievable,^2^, ^3^ then first understanding what is practically attainable given the available interventions is crucial. The value for money of these interventions is also important to consider given countries' limited budgets and competing health priorities, as is acknowledging that both probably differ between countries. Anaemia, especially in women of reproductive age, is a complex and multifaceted condition caused by many factors, including nutritional deficiencies, infections, chronic diseases, blood loss (eg, through heavy menstrual bleeding), and the increased haemoglobin demands of pregnancy and lactation.^9^ Although the most common cause of anaemia is iron deficiency,^5^ causes vary widely between populations due to demographics, diet, disease burden, and environmental conditions. The public health systems and economies of the world are equally diverse, with notable disparities in the structures, priorities, and funding of different health systems.^10^ These factors contribute to differences in anaemia prevalence, the effectiveness of public health solutions, and countries' capacities to invest in and successfully implement solutions at scale.^11^

**Research in context**

**Evidence before this study**

We searched PubMed on May 17, 2025, for articles that proposed or described target setting methods in the context of the 2015 Sustainable Development Goal (SDG) of halving anaemia in women of reproductive age with the search terms: (“Sustainable Development Goals”) AND (anaemia or anemia) AND (target OR goal). We included no language or publication date restrictions. Most studies related to the progress of individual countries towards the SDG or global nutrition targets or addressed factors that contributed to the burden of anaemia in the context of the 50% targeted reduction. We found two studies that discussed progress towards the SDG based on economic considerations of malnutrition and mentioned the economic infeasibility of reaching the malnutrition targets. One 2020 study reviewed the cost-effectiveness of different programmes for meeting the SDG malnutrition targets, including the scaling up existing interventions to a theoretical maximum coverage level, for 129 countries. One 2024 study evaluated the financial burden of failing to meet the global nutrition targets, providing calculators to estimate the costs of falling short of the targets to enhance decision making in the context of the broader economic costs of malnutrition. No studies proposed the use of cost-effectiveness as an alternative method of target setting.

**Added value of this study**

In this modelling study, we propose an evidence-based, value-driven approach to target setting in global health and development with cost-effectiveness modelling. Cost-effectiveness modelling provides a rigorous, transparent framework for establishing both individual country-level and global targets and can be applied to a wide variety of health and development goals. This is the first study to use cost-effectiveness analysis and simulation to estimate value-based global health and development targets.

**Implications of all the available evidence**

We suggest that future targets use cost-effectiveness as the framework for ambitious, achievable goals, not only for anaemia reduction, but for global health targets more generally. Although our approach leads to targets that are substantially lower than existing tragets, they are more likely to be achievable and are based on evidence from existing anaemia interventions. We propose an alternative methodology for target setting that considers the circumstances of each country, setting unique national targets that can be aggregated to form a single, easily communicated figure.

The upcoming post-SDG period presents an opportunity to reassess how international development targets are set, both for anaemia and for global development targets more generally. We aimed to propose a new method of target setting with cost-effectiveness analysis, an evidence-based process, for the next round of global health targets, with addressing anaemia in women of reproductive age as an example health objective. Our approach is grounded in health economic modelling of interventions: for each country, we aimed to incorporate variability in the underlying prevalence of anaemia, the costs and effectiveness of the different interventions recommended to address it, and the current and theoretical maximum levels of coverage of each intervention. When integrated with country-specific cost-effectiveness threshold (CET) estimates, indicating a country's willingness-to-pay for health gains, this approach creates a tailored national target for each signatory country that is both ambitious and more likely to be achievable. The data from this study inform section 4 of *The Lancet Haematology*'s Commission on meeting anaemia reduction targets.^6^

## Methods

### Model design

In this modelling stuy, we used a cost-effectiveness framework to identify cost-effective interventions for reducing the prevalence of anaemia in women of reproductive age (ie, aged 15–49 years) across 191 signatory countries. We integrated four different factors that influence country-level anaemia targets: the current prevalence of anaemia; the effectiveness and current and maximal coverage of recommended interventions available to each country; the local unit costs of these interventions; and country-specific CET estimates. We followed the Consolidated Health Economic Evaluation Reporting Standards framework for reporting (appendix pp 27–31).

**Figure 2:**
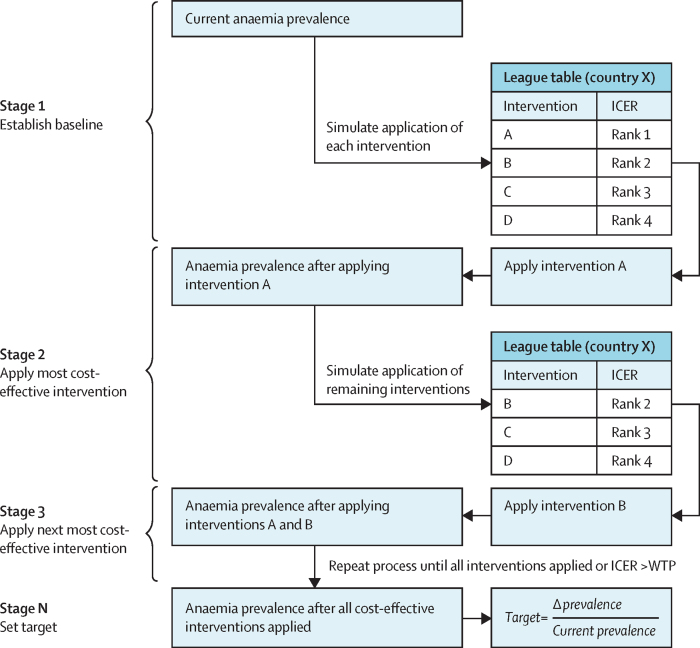
Flowchart for establishing national anaemia reduction targets The effects of each intervention are simulated sequentially until only those with an ICER above the country's CET remain unimplemented. CET=cost-effectiveness threshold. ICER=incremental cost-effectiveness ratio. WTP=Willingness-to-Pay.

Data on the first three factors were combined in a country-level simulation model to calculate an incremental cost-effectiveness ratio (ICER) for each intervention compared with the status quo, reflecting the incremental cost of reducing the burden of anaemia in disability-adjusted life-years (DALYs). This process is illustrated in figure 2. We assumed no mortality attributable to anaemia, and thus DALYs are equivalent to years of life lived with disability (YLDs). We also only considered reductions in YLDs attributable to anaemia; YLDs averted due to malaria burden were not included in our model.

A 1-year time horizon was taken due to the nature of the interventions being modelled, which were assumed to be immediately effective, and the requirement that nutritional anaemia interventions be continuously administered. Costs and benefits were therefore not discounted as no future costs or outcomes were considered. ICERs were compared with country-specific CETs, representing each country's willingness-to-pay for additional averted DALYs. Interventions with ICERs below the threshold were considered cost-effective.

We generated league tables for each country, ordering interventions by increasing ICER. If one or more interventions were cost-effective, the intervention with the lowest ICER was applied at its maximal level of coverage and the resulting impact on anaemia prevalence was estimated. The ICERs of the remaining interventions were then recalculated on the basis of the new (lower) anaemia prevalence. This process was iterated until either all interventions were applied or the remaining interventions were no longer cost-effective. The resulting anaemia prevalence was deemed the maximum cost-effective improvement in anaemia. We report median reductions in anaemia prevalence for each country with uncertainty intervals and then average these targets across all 191 countries to create a single global target that is simple to communicate. Targets are also reported by income grouping, as per the World Bank groupings in 2025.^12^

### Data

Current anaemia prevalence in each country was based on the Global Burden of Disease (GBD) database.^5^ These data are disaggregated by anaemia severity (ie, mild, moderate, or severe), age, sex, and year. We focused on the target population, women of reproductive age (ie, aged 15–49 years), and used the most recent (2021) data. Severity was classified in the GBD study by published WHO haemoglobin concentration thresholds for mild, moderate, and severe anaemia in both pregnant and non-pregnant women of reproductive age (appendix pp 2–3).^5^, ^13^ We supplemented the GBD data with pregnancy and malaria rates from the literature (table). Additional detail is provided in the appendix (pp 3–6).

**Table:** Summary of parameters used to model target populations, intervention effectiveness, and intervention coverage

		**Use in model**	Derivation and source*
**Prevalence of anaemia and population receiving interventions**			
Population of anaemic women aged 15–49 years in 2021 by country (A)		Population benefitting from oral iron supplementation and staple food fortification	2021 GBD study^5^
Population of all women aged 15–49 years in 2021 by country (B)		Population receiving oral iron supplementation	Derived from GBD estimates of prevalence and the rate of anaemia among women aged 15–49 years^5^
Total population in 2021 by country (C)		Population receiving staple food fortification	Derived from GBD estimates of prevalence and the rate of anaemia among individuals of all ages and sexes^5^
Population of pregnant women in 2021 by country (D)		Population receiving oral iron supplementation and presumptive antimalarials	Derived from fertility and stillbirth rates* and the population of women aged 15–49 years (B)
Population of pregnant anaemic women in 2021 by country (E)		Population benefitting from oral iron supplementation	Derived from the population of women of reproductive age (B) and GBD estimates of anaemia prevalence among women aged 15–49 years^5^
Population of pregnant women with malaria and anaemia in 2017 by country (F)		Population benefitting from presumptive antimalarials	Derived from the population of pregnant anaemic women (E) and estimates of malaria prevalence*
**Intervention effectiveness**			
RR of still being anaemic after intervention			
	Staple foods fortification	Effectiveness: RR 0·73 (95% CI 0·55–0·97)	Based on a Cochrane review of wheat-flour fortification*
	Antenatal antimalarials	Effectiveness: RR 0·90 (95% CI 0·87–0·93)	Based on a meta-analysis on IPTp-SP*
	Oral iron supplementation in pregnant women	Effectiveness: RR 0·30 (95% CI 0·20–0·47)	Cochrane review of daily oral iron supplementation in pregnancy*
	Oral iron supplementation in non-pregnant women	Effectiveness: RR 0·65 (95% CI 0·49–0·87)	Cochrane review of intermittent oral iron supplementation in adolescent and adult menstruating women*
Decrease in intervention effectiveness after scale-up from trial to real-world settings		Reduction applied to effectiveness of oral iron supplementation: 38% (95% CI 21–69)	Von Klinggraeff et al^14^
**Intervention coverage**			
Current coverage level			
	Staple foods fortification	Fraction of eligible population currently receiving intervention	Estimated from coverage of fortified staple foods after accounting for compliance and quality*
	Antenatal antimalarials	Fraction of eligible population currently receiving intervention	Estimated from the most recent DHS* data on the percentage of women receiving three or more sulfadoxine–pyrimethamine tablets during pregnancy
	Oral iron supplementation in pregnant women	Fraction of eligible population currently receiving intervention	Estimated from DHS* data on the percentage of women receiving any amounts of iron tablets during pregnancy
	Oral iron supplementation in non-pregnant women	Fraction of eligible population currently receiving intervention	Estimated as a fraction (0·41) of pregnant women receiving iron supplementation on the basis of NHANES data on uptake of iron tablets in pregnant and non-pregnant women*
Theoretical maximum coverage level			
	Staple foods fortification	Fraction of eligible population receiving intervention after scale-up	Estimated from coverage of industrially processed staple foods*
	Antenatal antimalarials	Fraction of eligible population receiving intervention after scale-up	Estimated from DHS* and MICS data on coverage of antenatal care
	Oral iron supplementation in pregnant women	Fraction of eligible population receiving intervention after scale-up	Estimated from DHS* and MICS data on coverage of antenatal care
	Oral iron supplementation in non-pregnant women	Fraction of eligible population receiving intervention after scale-up	Assumed to be equivalent to coverage of antenatal care

A full list of calculations for each variable can be found in the appendix (pp 4–13). DHS=demographic and health surveys. GBD=Global Burden of Disease. IPTp-SP=intermittent preventive treatment of malaria in pregnancy with sulfadoxine–pyrimethamine. MICS=multiple indicator cluster survey. MIS=malaria indicator survey. NHANES=national health and nutrition examination survey. RR=relative risk.

* More detail on the derivation and sources for all parameters is provided in the appendix (pp 3–15).

To ensure consistency with global health guidelines, we compiled a list of interventions for anaemia on the basis of their inclusion in both the WHO e-Library of Evidence for Nutrition Actions (eLENA)^15^ and the World Bank's investment framework for meeting the global nutrition target for anaemia:^16^ oral iron supplementation in menstruating and pregnant women; fortification of staple foods (eg, rice, wheat, and maize) with iron; and intermittent preventive treatment with sulfadoxine–pyrimethamine during pregnancy. Iron requirements for pregnant women are notably higher than those for menstruating women,^17^ and were therefore considered a separate intervention.

We obtained effectiveness estimates from the WHO guidelines in eLENA. Effectiveness is quantified as the relative risk of remaining anaemic after treatment, estimated for each intervention from efficacy estimates in published meta-analyses of randomised trials. Intervention effectiveness in observational studies often differs from treatment efficacy within trials due to factors such as lower compliance rates, resource constraints, and variations in programme delivery. We adjusted the reported efficacy of oral iron supplementation by reducing treatment effectiveness during roll-out, on the basis of previous scaled-up individual nutrition interventions.^14^ We did not apply this reduction to staple food fortification or presumptive treatment of malaria for pregnant women as these treatments are less likely to be affected by factors related to compliance, adherence, and programme delivery.

Baseline coverage data for each modelled intervention were sourced from international and national datasets and large-scale population surveys. These datasets provide national estimates of the proportion of the target population currently receiving each intervention, or proxy indicators of those interventions. Details are provided in the table and the appendix (pp 32–456).

To estimate unit costs, we adopted a microcosting approach from a health-care system perspective, considering both programmatic and point-of-care delivery costs. The costs for these interventions were derived from commodity costs, supply chain costs, service delivery costs, and programme-level costs. To account for inflation and currency conversion, we followed best practices for costing in health economic studies by Turner and colleagues.^18^ We used the latest available data on country-specific gross domestic product (GDP) deflation factors to adjust for inflation of non-tradable goods and purchasing power parity factors to convert between local currencies and US dollars. Accordingly, costs are reported in 2023 US dollars. Details are provided in the appendix (pp 14–16) and in the publication by Victoria L Oliver and colleagues.^19^ We considered a range of potential CETs. As our base case, we used a 1 × GDP per capita threshold, recommended by the WHO Commission on Macroeconomics.^20^ However, this threshold does not recognise opportunity costs, which acknowledge that resources for health are finite, and that the amount invested in an intervention should not exceed the next best use of those resources.^21^ Accordingly, for a conservative estimate, we used country-specific estimates of CETs (in dollars) per quality-adjusted life-year (QALY) gained, based on total health expenditures per capita and life expectancy derived by Pichon-Riviere and colleagues^22^ and extrapolated to countries without CETs (appendix pp 16–17)*.* This threshold is similar to opportunity cost-based thresholds in the literature that consider the displacement of resources from other treatments.^21^ We also considered several alternative thresholds and a scenario in which interventions were scaled up to their theoretical maximum regardless of cost, representing the maximum achievable reduction (appendix pp 20–23).

### Uncertainty analysis

Uncertainty in all model input parameters was modelled using 1000 Monte Carlo simulations. Rather than using fixed point estimates, we sampled from probability distributions for each input parameter on the basis of published evidence. This approach generated a distribution of plausible anaemia reduction targets for each country, reflecting combined uncertainty across all parameters. We calculated the median and 95% uncertainty intervals for each country across simulations, calculating a global target by averaging across all 191 countries. We conducted scenario analyses to evaluate the basic structure of the model: we tested intervention coverage assumptions by estimating reductions given zero baseline coverage, and tested resource constraints by removing CETs entirely, implementing all interventions. A full description of the uncertainty analyses is provided in the appendix (pp 16–23). All analyses were done in R (version 4.2.2)^23^ and further details are provided in the appendix (p 1).

### Role of the funding source

The funder had no role in study design, data collection, data analysis, data interpretation, or writing of the report.

## Results

**Figure 3:**
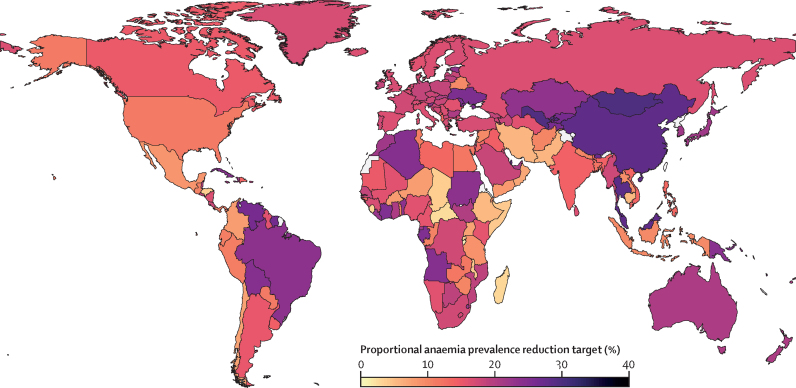
Recommended national anaemia reduction targets based on country-specific health economic modelling Map showing proposed national targets for anaemia reduction among women of reproductive age (ie, aged 15–49 years), derived with our health economic model and a cost-effectiveness threshold of 1 gross domestic product per capita per disability-adjusted life-year averted. Colours indicate the percentage reduction in anaemia prevalence that each country could feasibly and cost-effectively achieve by scaling up available interventions over the next international development goal period. Uncertainty ranges associated with these targets are shown in figure 4.

**Figure 4:**
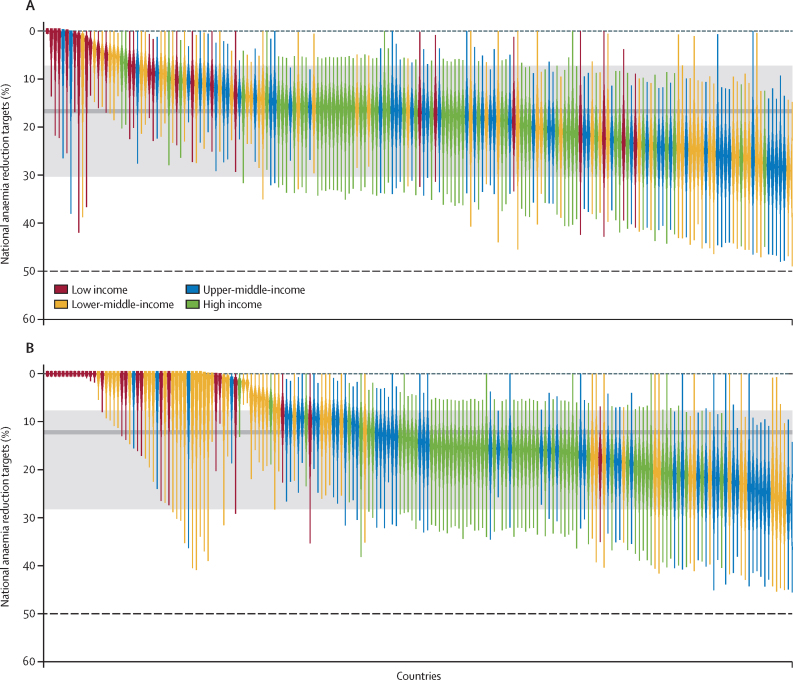
Global and national anaemia reduction targets under different CETs Reproduced with permission from Sarah H Atkinson and collagues.^6^ Each panel shows the recommended anaemia reduction targets for women of reproductive age (ie, aged 15–49 years) in 191 signatory countries, colour-coded by country income level. Coloured squares represent the mean target for each country; vertical, coloured lines represent the effects of uncertainty in model parameters (eg, intervention costs, effectiveness, and coverage); solid grey lines indicate global mean targets with 95% uncertainty intervals shown by grey shading; and the dashed line indicates the current SDG anaemia reduction target of 50%. (A) Targets under a CET of 1 × GDP per capita. (B) Targets under a more conservative CET based on Pichon-Rivière and colleagues.^22^ CET=cost-effectiveness threshold. GDP=gross domestic product. SDG=Sustainable Development Goal.

Recommended country-level anaemia reduction targets varied by geographical region (figure 3). Our model estimated that, with available interventions, the international community can set an achievable and ambitious 17% (95% uncertainty interval [UI] 5–34) global target for reduction in anaemia prevalence among women of reproductive age for the next iteration of international development goals under a willingness-to-pay threshold of 1 × GDP per capita (figure 4A). Under a more conservative CET incorporating opportunity costs, this global target decreases to 12% (UI 3–29; figure 4B).

Recommended national targets vary substantially between countries, spanning from 0% to 29% (figure 4). Targets tend to be higher in upper-middle-income and high-income countries. The mean target for LMIC countries was lower than the target for all countries in both the baseline scenario (12% *vs* 17%) and with alternative thresholds. Full details on national-level targets are included in the appendix (pp 32–456)*.*

**Figure 5:**
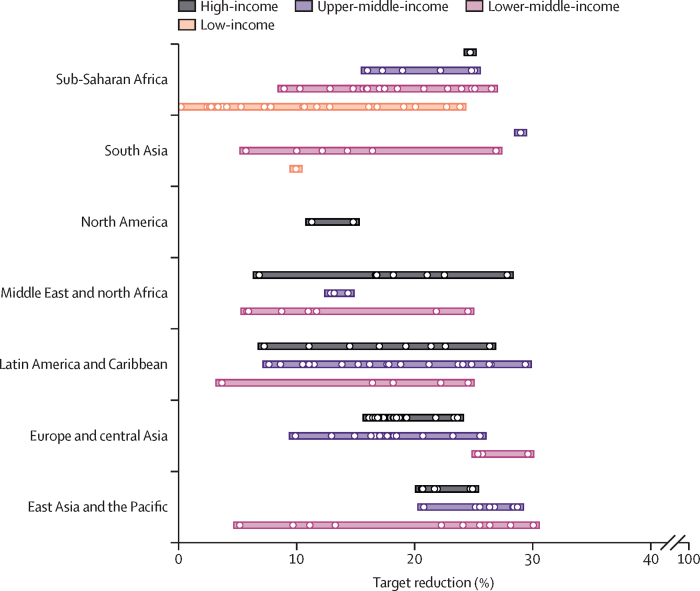
National anaemia reduction targets grouped by region and income level Dots indicate the median recommended targets for each country within a region–income grouping; horizontal bars encompass 95% of the targets observed across all countries and Monte Carlo iterations in each group. The horizontal bars represent country-level differences in suggested targets rather than the sampling variation in each target. Results are based on a cost-effectiveness threshold of 1 × gross domestic product per capita.

Regionally, mean anaemia reduction targets range from 6% in sub-Saharan Africa to 18% in any region in East Asia and the Pacific (figure 5). North America and Europe and central Asia share similar mean targets (14% reduction and 16% reduction, respectively), whereas targets for south Asia (9% reduction) and the Middle East and north Africa (12% reduction) reflect more modest achievable gains. Despite these trends, variation within regions and income groups (ie, the dispersion of dots in each row) was often greater than the variation between groups (ie, the differences in targets between rows; figure 5).

Interventions by World Bank region and income group are ranked in the appendix (p 19), providing a league table of interventions based on cost-effectiveness and feasibility (national level data are also shown in the appendix [pp 32–456]). Staple food fortification was nearly always the most cost-effective intervention despite its limited effectiveness (table). Oral iron supplementation for pregnant women was the second most cost-effective intervention and oral iron supplementation for all women was the third most cost-effective intervention. Preventive antimalarial drugs during pregnancy ranked lowest among interventions across all regions.

Removing cost constraints altogether increased the global reduction target to 22% (UI 11–36), representing the maximum achievable reduction in anaemia prevalence. This target is similar to the 24% (UI 9–44) reduction target observed when assuming zero baseline coverage for all interventions (appendix pp 20–23).

## Discussion

Our results support a global target of a 17% reduction in anaemia in women of reproductive age given currently recommended interventions for anaemia, falling well short of the current SDG target. Across all modelled scenarios, including optimistic assumptions about willingness-to-pay thresholds and coverage, the highest achievable global target was less than half the existing target of a 50% reduction in prevalence. Within global headline targets, there was wide variation in national targets, reflecting the diversity of national capacities and constraints. In LMICs, CETs are generally lower, resulting in fewer cost-effective interventions. Regional differences reinforce the importance of tailoring national investment strategies to context-specific constraints and opportunities, underscoring the need for country-specific analysis when setting realistic national and global targets.

Uniform international development targets hold some countries to unrealistic standards and are insufficiently ambitious for others. Our process tailors each country's target to its particular combination of capacity and challenges, serving as a globally consistent foundation for country-level targets that reflect specific demographic, economic, and public-health contexts. By identifying what is locally achievable for each country, this approach creates a pathway towards meaningful progress on anaemia reduction while maintaining a collective international goal. It also adheres to principles of good governance, such as transparency, efficiency, and inclusivity, ensuring that the target-setting process is both evidence-based and participatory.

Our approach is consistent with and expands on the methodological process laid out by WHO's Choosing Interventions that are Cost-Effective (CHOICE) programme.^24^ We expanded on these methods by simulating the implementation of these interventions to derive ICERs and subsequently using said ICERs to show how implementation affects prevalence. Just as cost-effectiveness should be used to prioritise competing interventions, we argue that it should also inform realistic, value-based disease reduction targets that consider the many competing demands on health budgets. To our knowledge, this analysis reflects the first use of cost-effectiveness methods to inform national targets for global development indicators.

Although ambitious goals can serve as so-called stretch targets to inspire progress, unachievably high targets might instead lead to demoralisation and criticism when countries fail to reach them, despite making meaningful progress.^25^, ^26^ Criticism of the SDG predecessors, the Millenium Development Goals, included the implausibility of some countries' abilities to reach the targets, contributing to pessimism about their capabilities and progress,^27^, ^28^ a deeply unattractive outcome given the voluntary nature of international development goals. Unachievable targets could also encourage overinvestment in anaemia relative to competing health priorities, leading to fewer health gains than addressing diseases with more cost-effective solutions.

Our targets assume that anaemia will be addressed in isolation. However, anaemia reduction is intertwined with other public health challenges, including malnutrition and malaria. Societal and systemic factors, including women's education and access to primary and reproductive care, including hormonal contraception for heavy menstrual bleeding, also play crucial roles in shaping outcomes for women of reproductive age.^29^ Analyses that focus solely on the incremental cost-effectiveness of individual interventions risk missing the opportunity presented by broader solutions. Our analysis might therefore overlook the multisectoral context of public health, in which indirect interventions, for example increased access to basic health care, might prove more cost-effective in the long term and facilitate widespread public health improvements beyond anaemia reduction. The framework of international development targets, particularly the SDGs, is predicated on recognising and addressing the interconnected nature of development challenges. Our approach to analysing anaemia interventions does not fully engage with this complexity, highlighting a need for the integration of broader systemic and societal considerations, including what constitute as benefits and costs.

Similarly, although we incorporate the overall burden of anaemia through DALYs and cost-effectiveness, our approach addresses this burden through the lens of prevalence due to the framing of the SDGs. As we note in the methods section, the burden of anaemia does not fall equally on all women, and the severity of anaemia should be used to inform national and international decisions about priority. We suggest that future global health targets begin incorporating DALYs or QALYs to focus on populations with the greatest burden.

A potential criticism of cost-effectiveness approaches is that they set low expectations for, and imply minimal effort from, many countries with low targets. Substantial investment in domestic anaemia treatment for these countries might not be as cost-effective as investing in other health priorities, especially as reaching full coverage might incur diminishing returns on investment.^30^ However, due to their sizeable health budgets, signatory higher-income countries could use their purchasing power to fund anaemia reduction efforts in countries with insufficient health budgets to treat endemic anaemia. This approach would require that signatory countries be accountable for global reductions, acknowledging differences in anaemia burden. Under individual targets, countries with an anaemia prevalence of 4% could achieve a relative reduction of 25% by reducing their absolute prevalence by 1%. This relative reduction would give the illusion of substantial progress while making little impact on the global burden of anaemia, especially compared with reductions that could be achieved by investing in other nations' progress.

Our approach considers allocative efficiency, rather than affordability related to the availability of public health funds. This distinction highlights constraints on the efficiency of implementing these interventions relative to the opportunity cost of expenditures on other priorities and the total available funding to implement these programmes. Our analysis does not address the latter, which would require estimation of national health budgets, for which we were unable to collect sufficient data. We recommend that this constraint be defined by participating nations as a basis for negotiation on future targets.

Our health economic modelling is based on datasets that are consistently available at a global level. Although this approach ensures comparability across countries, it simplifies the complex realities of intervention implementation. For example, in some settings, increasing the coverage of oral iron supplementation might be hindered by stigma around pill-taking, which can be misinterpreted as indicative of illness or disease.^31^ Such factors vary among economies, regions, and subpopulations within countries. The knowledge of local public health experts in each country will better reflect local contexts. We therefore highly encourage use of our GitHub code repository, in which we provide open sharing of code, data, and analytical tools, to refine these national targets with local knowledge and experience.

Our proposed methods, and the achievement of anaemia targets generally, will probably rely on international collaboration, including cooperation on collecting and compiling data for surveillance and analysis and support for intergovernmental agencies that coordinate global health efforts. Politically motivated threats to these programmes and institutions undermine not only efforts to reach national anaemia targets but also the information required for global progress.

Targets have previously been chosen through consensus-building negotiations, which tend to emphasise aspirational goals over evidence-based feasibility. In this Article, we do not argue against negotiated targets but suggest that evidence of what is feasible forms the basis for such negotiations. The failure to achieve the current 50% target is a clear example of differences in capacity, local contexts, and resourcing leading to potentially unrealistic and disheartening outcomes. The proposed framework provides a roadmap for target setting in other areas of global health, including malaria, maternal mortality, and childhood stunting.

In 2030, the global community will probably confront a stark reality: the ambitious anaemia reduction targets set in the SDGs and global nutrition targets have not been achieved. Cuts to global health budgets and the undermining of the institutions committed to meeting these targets will probably lead to reflection on how best to define progress while fostering accountability and ambition. We present an approach grounded in evidence that, although less ambitious than the current 50% goal, might offer a useful blueprint for designing future global targets for both anaemia and global health generally. Transparency enables stakeholders to trace decisions to their underlying data and assumptions, encouraging buy-in and shared ownership of outcomes. This approach aligns with the ethos of the SDGs, which seek to inspire societal change through collective responsibility and shared motivation. As we seek to improve the lives of people worldwide, this evidence-driven approach aligns aspirations with actions, enabling nations to establish goals they can achieve while striving for impactful progress regarding human health and quality of life.

This study reports that the SDG target for anaemia in women of reproductive age is unlikely to be achievable on the basis of currently recommended interventions. Our results suggest that the maximum achievable reduction is just half of the 2030 target. We recommend that future targets in global health and development are based on evidence from available interventions and cost-effectiveness, and present an alternative target-setting approach following these principles.

### Contributors

### Data sharing

All data used in this Article are available on GitHub, and the code, data, and analytical tools used are in the appendix (pp 2–20).

## Declaration of interests

SRP has received grants from the National Health and Medical Research Council, royalties and patents from Silence Therapeutics, and has participated on advisory boards for CSL-Vifor; holds an unpaid role as Director of the WHO Collaborating Centre for Anaemia Detection and Control; and has received a grant (INV-059675) from the Gates Foundation that supported the Commission, including salaries for SL, RB, NC, CG-W, VLO, and YW, and travel support for Commission meetings for SRP, SL, MB, RB, NC, VLO, and YW. All other authors declare no competing interests.
