# Supplementary material for: Cost-effective targets for anaemia reduction in 191 countries: a modelling study
Source: Lancet Haematol. 2025 Aug 26;12(9):e674–83. doi: 10.1016/S2352-3026(25)00168-1 (PMC12405179; doi:10.1016/S2352-3026(25)00168-1)
Supplement: Supplementary appendix [file mmc1.pdf]

THE LANCET
Haematology

Supplementary appendix

This appendix formed part of the original submission and has been peer reviewed. We post it as supplied by the authors.

Supplement to: Blythe R, Carvalho N, Holloway-Brown J, et al. Cost-effective targets for anaemia reduction in 191 countries: a modelling study. *Lancet Haematol* 2025; published online Aug 26. https://doi.org/10.1016/S2352-3026(25)00168-1.

Supporting Information to:

Cost-effective targets for anaemia reduction in 191 countries: a modelling study

In this supplementary file, we provide additional details on the datasets and analyses reported in the main text. To see how the data were used in the analysis, please see https://github.com/robinblythe/Lancet_anaemia. All analysis was conducted in R version 4.2.2[1] utilising the countrycode[2] and EnvStats[3] packages.

SUPPORTING INFORMATION 1: ANAEMIA PREVALENCE DATA

SUPPORTING INFORMATION 2: INTERVENTIONS

2.1 Selected interventions
2.2 Voltage drops
2.3 Intervention effect sizes
2.3.1 Staple food fortification
2.3.2 Intermittent preventive treatment with sulfadoxine-pyrimethamine (IPTp-SP)
2.3.3 Iron supplementation for pregnant women
2.3.4 Iron supplementation for non-pregnant women of reproductive age

SUPPORTING INFORMATION 3: COST AND COST-EFFECTIVENESS THRESHOLD ESTIMATION

3.1 Iron supplementation and IPTp-SP
3.2 Staple food fortification
3.3 Cost uncertainty
3.4 Cost-effectiveness thresholds

SUPPORTING INFORMATION 4: SIMULATION METHODS

4.1 Objective and approach
4.2 Modelling the effect of increases in intervention coverage
4.3 Identifying the optimal package of interventions
4.4 Propagating parameter uncertainty through to results
4.5 Model assumptions and limitations
4.6 Exploring structural uncertainty using scenario analyses

REFERENCES USED IN SUPPORTING INFORMATION 1 – 4

SUPPORTING INFORMATION 5: CHEERS CHECKLIST

SUPPORTING INFORMATION 6: LEAGUE TABLES AND TARGETS FOR 191 COUNTRIES

SUPPORTING INFORMATION 1: ANAEMIA PREVALENCE DATA

We obtained anaemia prevalence data from 1990-2021 from the Global Burden of Disease (GBD) study.[4] The GBD estimated disease prevalence using a sophisticated integrative approach.[5] It should be noted that GBD data are the result of statistical modelling, and prevalence and rate data are reported with their own lower and upper uncertainty bounds. We sampled from these bounds, as well as the point estimate, using triangular distributions. The prevalence of anaemia, expressed as a proportion of the population of women of reproductive age, is shown in Figure S1.

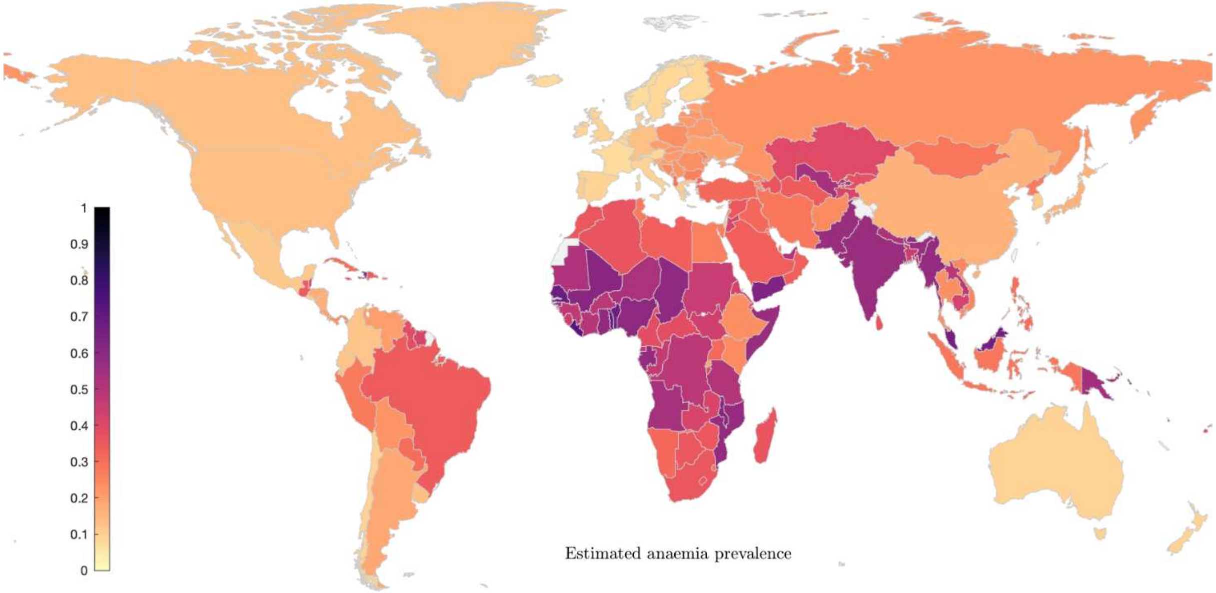

Figure S1: Estimated prevalence of overall anaemia *(mild, moderate, severe) as a proportion, across all modelled countries. Values are estimated by the Global Burden of Disease Study.*

We also incorporated the GBD data on burden of anaemia, reported as Years Lived with a Disability (YLDs). We converted means and standard errors of YLD estimates to Beta distributions using the method of moments. Briefly, YLDs are equivalent to a Disability-Adjusted Life Year (DALY) when there is no excess mortality attributed to the disease or disability.[4] Anaemia is modelled as having the following associated YLDs based on severity (Figure S2):

$$\text{mild anaemia DW} \sim \text{Beta}(\alpha = 4.0, \beta = 1091.3)$$

$$\text{moderate anaemia DW} \sim \text{Beta}(\alpha = 23.0, \beta = 410.4)$$

$$\text{severe anaemia DW} \sim \text{Beta}(\alpha = 25.2, \beta = 141.6)$$

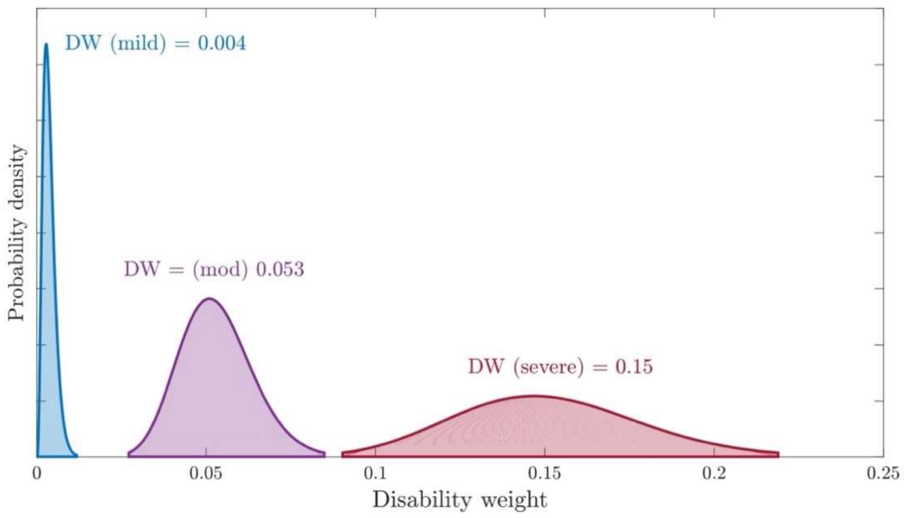

Figure S2: Estimated disability weights (DW) for mild, moderate, and severe anaemia*, based on Global Burden of Disease (GBD) study classifications. These weights are used to quantify the burden of anaemia in terms of Disability-Adjusted Life Years (DALYs).*

We combined 2021 pregnancy and stillbirth rates[6,7] in our dataset to estimate the proportion of pregnant women at any given time. Fertility rates, representing number of live births per woman, were assumed to be uniformly distributed across the population of women of reproductive age. Stillbirths, reported as a median estimate per 1,000 women, were divided by 1,000 and multiplied by the fertility rate. Assuming that the duration of pregnancy leading to a live birth was 9 months and the duration of pregnancy leading to a stillbirth was slightly shorter at 7.8 months, we combined published rates to obtain a proportion, and multiplied this proportion by the population of women of reproductive age.

Malaria rates for each country, obtained from the Malaria Atlas Project,[8,9] were calculated by converting cases per 1,000 to a percentage of the population that was likely to be infected with malaria at any given time. For example, if Angola had an incidence of 270 cases per 1,000 in 2020, we estimated that the probability of a pregnant woman in Angola presenting to an antenatal clinic with malaria was 27%. This simplifying assumption was unlikely to have a significant impact on results, given it was likely an overestimate that did not lead to antimalarials being funded as cost-effective in most countries where malaria was endemic.

SUPPORTING INFORMATION 2: INTERVENTIONS

While there are many interventions that target or affect anaemia, most lack supportive evidence of effectiveness, particularly when implemented as public health interventions. Given that our analysis was designed to present a new methodology rather than specify exactly how each country should address anaemia burden, we followed the set of interventions recommended by the World Bank's Investment Framework for Nutrition (2017).[10] This set aligns with those presented in the World Health Organisation (WHO) e-Library of Evidence for Nutrition Actions (eLENA).[11] We hope that our method and code support the development of further analysis that include a wider range of interventions that have (or accumulate) a solid evidence basis.

2.1 Selected interventions

Four interventions were identified:

1. Fortification of industrially produced staple foods (wheat, rice, maize) with iron compounds. This strategy is aimed at increasing dietary iron intake by enriching widely consumed staples to address chronic deficiencies across populations.
2. Intermittent preventative treatment of malaria in pregnancy with sulfadoxine-pyrimethamine (IPTp-SP), an established and cost-effective anti-malarial regimen. Given the relationship between malaria and anaemia, especially for pregnant women, preventing malaria in antenatal clinics is included in our analysis.[12]
3. Regular iron supplementation for pregnant women, a widely recommended practice to address increased iron demands during pregnancy and improve maternal and neonatal health.
4. Iron supplementation for menstruating adult women and adolescent girls, aiming to address chronic dietary deficiencies and improve overall health outcomes in non-pregnant women of reproductive age.

Note:

- Although preventive deworming chemotherapy for women of reproductive age in areas where anaemia is a severe public health problem is a WHO conditional recommendation, the WHO states that there was a lack of evidence that deworming treatments improve anaemia and so this intervention has not been included in our analysis.[13]
- Intervention 3 often includes recommendations for broader micronutrient supplementation, where iron supplementation is included alongside at least two or more other micronutrients (e.g., folic acid, vitamin A, zinc). However, because these micronutrient formulations come at an increased cost without a difference in efficacy for reducing anaemia compared to iron supplementation alone,[14] we focused only on iron supplementation.

2.2 Voltage drops

The outcomes observed in real-world implementation of public health interventions often differ from those observed in trials. This gap between the tightly controlled conditions of trials and the complexity of large-scale implementation is often referred to as the "voltage drop."[15] Factors contributing to this drop include challenges in ensuring adequate supply chains, maintaining program quality, and ensuring compliance among target populations in diverse and often resource-constrained settings.[16-18]

To account for this difference in our model, we adjusted the reported efficacy of iron supplementation interventions reported in meta-analysis of clinical trials by applying a 38% [21%, 69%] reduction in treatment effectiveness during increases in population coverage. This adjustment, based on observed reductions in effectiveness across 10 scaled-up individual nutrition interventions,[19] moderates our model predictions to provide a more realistic basis for target-setting. However, we note that the studies in this review primarily focused on dietary diversity interventions implemented in high-income countries, rather than anaemia interventions for anaemia in low- and middle-income countries (LMICs).[18] These interventions were typically delivered in structured community or institutional settings with superior resources and access to trained personnel than LMIC.

We applied this voltage drop to our modelled effect sizes for iron supplementation, as this intervention requires women to take tablets (daily or weekly) at home and is therefore susceptible to compliance issues which may affect intervention effectiveness. However, we do not apply it for fortification of industrially produced staple foods with iron compounds or IPTp-SP. In the former case, the measured effect size of staple food fortification has often been estimated from observational studies of populations who are exposed to the scaled-up version of the intervention. IPTp-SP is administered by skilled providers at health care visits

and is therefore less dependent on individual behaviour or compliance, key factors responsible for the voltage drop. However, we acknowledge that there is often a drop in adherence, for example due to non-attendance, that might be observed in the scale-up of an intervention to business-as-usual.

2.3 Intervention effect sizes

This section summarises the relative rates (RRs) of effectiveness for the four interventions included in our analysis. Relative rates report the proportional reduction in anaemia prevalence for all individuals treated with each intervention, as derived from systematic reviews and meta-analyses. These effect sizes provide the foundation for our modelling approach, allowing us to estimate the reduction in anaemia prevalence that could be achieved under various levels of intervention coverage.

Each intervention's RR is presented as a probability distribution to capture the inherent uncertainty in its effectiveness (Figure S3). These distributions (either beta or normal distributions) were fit to the mean effectiveness and uncertainty of the available evidence.

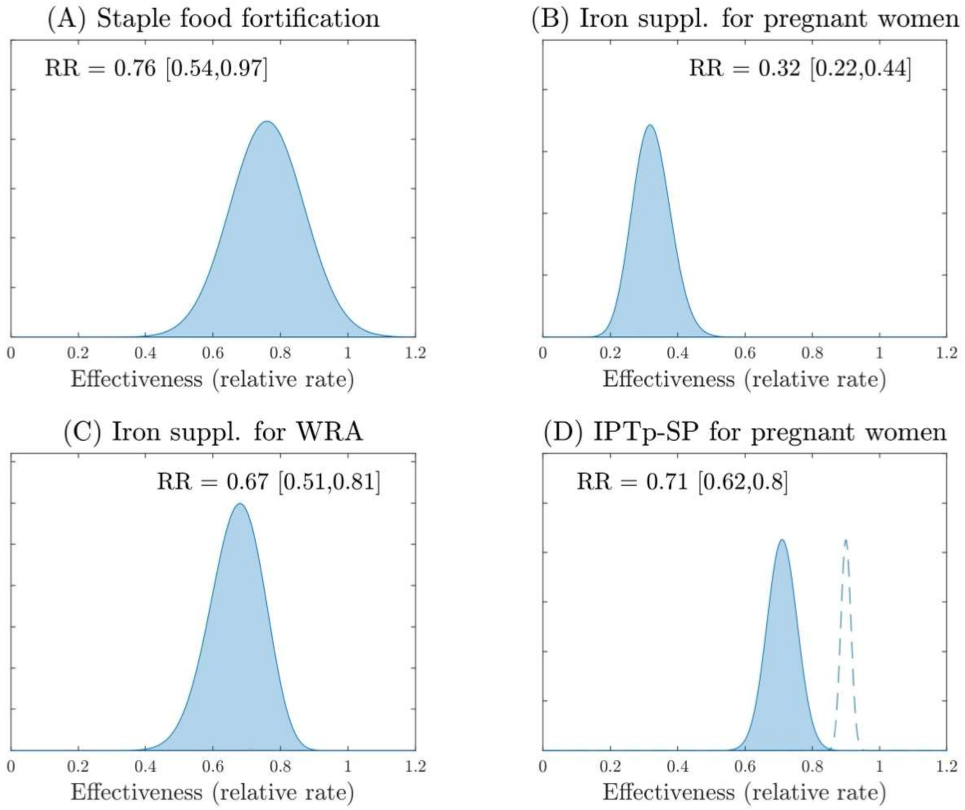

***Figure S3: Estimated effectiveness, expressed as relative rates (RR) for the four interventions** modelled in this study. Each panel shows the effectiveness of a particular intervention, with associated uncertainty, as derived from systematic reviews. These distributions are incorporated into our Monte Carlo simulations to propagate uncertainty through the anaemia target-setting model. In panel (D), we show both the RR for the intervention when applied to the subpopulation of pregnant women with malaria, and also the original effect size measured by the systematic review (dashed line), when applied to all pregnant women in a country with a 35% prevalence of malaria.*

2.3.1 Staple food fortification

We model fortification in the three most widely consumed staple foods: maize flour, rice, and wheat flour. Fortification of **maize flour** with iron is recommended to prevent iron deficiency in populations, particularly vulnerable groups such as children and women.[20] Fortification of **rice** with iron is recommended as a public health strategy to improve the iron status of populations, in settings where rice is a staple food[21] Fortification of **wheat flour** with iron is recommended to improve haemoglobin concentrations and iron status and to prevent anaemia and iron deficiency in populations.[22] Compared to wheat flour (with no added iron), iron-fortified wheat flour has been shown to reduce the risk of anaemia in populations.[23]

The effectiveness of fortification varies between staples. A 2021 Cochrane systematic review[23] of 5 studies estimated that the relative rate of anaemia in individuals who consumed fortified wheat flour is:

RR = 0.73 [0.55 - 0.97]

Which we applied to all countries, based on the use of wheat in nearly all countries where staple food fortification is mandated.[24] We approximated this distribution with a normal distribution with parameters $\mu = 0.76, \sigma = 0.11$. While this resulted in a slightly higher mean relative rate, it accurately reproduced the confidence bounds (Figure S3). Consumption of staple foods varies greatly across signatory countries. A more nuanced approach than applying wheat flour evidence to all staple food fortification would be to use individual risk ratios (RRs) for each country based on their relative prevalence of each type of fortified cereal grain. We encourage further work in this area, particularly through large, sufficiently powered trials.

Maximum intervention coverage: We considered maximum coverage to be the fortification of all industrially produced wheat flour, maize flour, and rice (Figure S4). Rather than model each staple food explicitly, we calculated these quantities for each of these staple foods and aggregated the results proportional to the national consumption of each staple. We used data from the UN Food and Agriculture Organisation (FAO)[25] on the domestic supply of each staple to estimate these proportional consumption values in all countries, following the methods outlined by the WHO.[26]

We then used data from the Global Fortification Data Exchange (GFDE)[27] to determine the current proportion of each staple in each country that is produced industrially and is therefore available for potential fortification. In some countries, the GFDE only provided industrial production levels for a subset of the staples. In those cases, we estimated the missing industrial production percentages from the average of those staples where data was available. For countries where no data on industrial fortification was available for any staple (49 countries out of 218, primarily small island nations), we inferred fortification levels from the average across all countries with data in the same Organisation for Economic Co-operation and Development (OECD) region and income level. Accordingly, small island countries are heavily represented in the countries with high targets, likely due to this limitation.

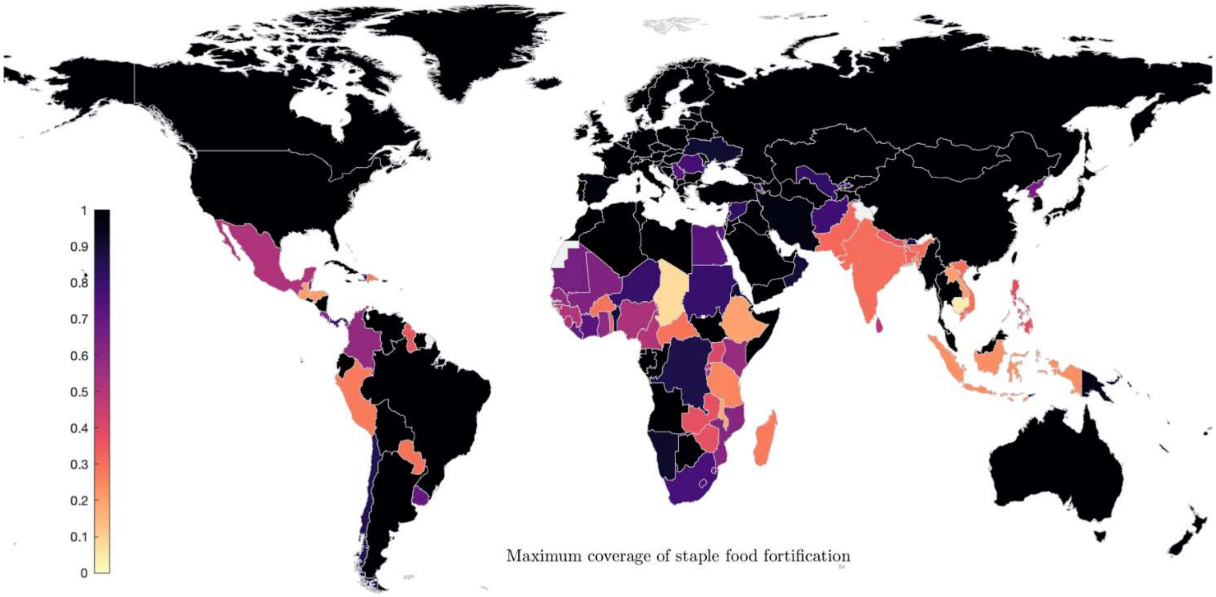

Figure S4: Estimated maximum feasible coverage for staple food fortification*, as a proportion, across all modelled countries. Data are taken from the Global Fortification Data Exchange*

Current intervention coverage: The same GFDE and FAO databases were used to estimate the current coverage of staple food fortification with WHO-approved iron fortificants (Figure S5). For each country, GFDE data was used to estimate what percentage of the average requirement for iron fortification was present in each staple, after accounting for compliance and quality. Note that we did not limit our analyses to only consider WHO-recommended iron fortification compounds. Many countries had voluntary or mandatory fortification of staple foods, but most countries (96%) who reported realised levels of iron fortification in their staple foods, reported that they were below WHO-recommended levels (by an average of 75%).

Across typical levels of fortification (e.g., less than 15mg/kg), blood iron levels respond linearly to intake of dietary supplement.[28] We therefore considered current coverage levels to be the product of the proportion of currently fortified staples, and the proportion of recommended fortification in those staples (both as reported on the GFDE). For example, if 50% of a country's wheat flour is produced industrially, and iron fortification levels in wheat flour are at 50% of recommended levels after accounting for compliance and quality, we assumed that the current coverage levels are 25%, and that the maximum feasible level of coverage would be 50%.

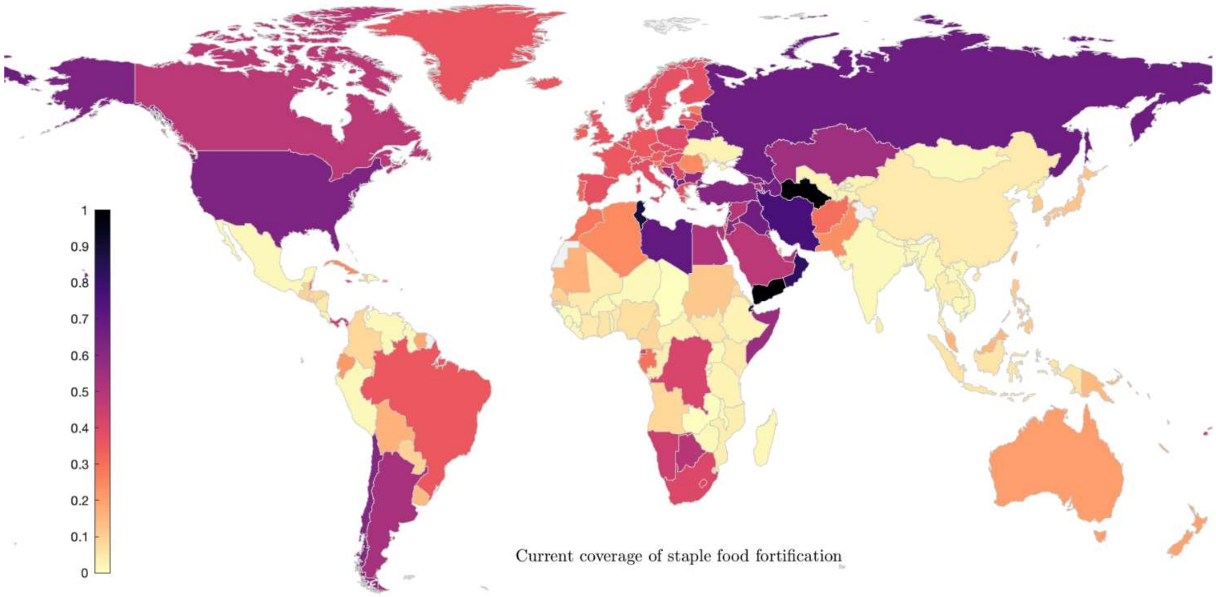

Figure S5: Estimated current coverage for staple food fortification*, as a proportion, across all modelled countries. Data are taken from the Global Fortification Data Exchange [GFDE 2024]*

2.3.2 Intermittent preventive treatment with sulfadoxine-pyrimethamine (IPTp-SP)

Malaria infection during pregnancy increases the risk of a range of illnesses, including anaemia. WHO recommends a package of interventions for preventing and controlling malaria during pregnancy.[29] These includeIPTp-SP in areas with moderate to high transmission of *P. falciparum*, for all pregnant women in their first or second pregnancy. Dosing should start in the second trimester and doses should be given at least 1 month apart, with the objective of ensuring that three recommended doses are received.[30]

As reported in the WHO guidelines, based on a systematic review of 53 studies, IPTp-SP reduces the overall prevalence of maternal anaemia. We applied the relative rate estimates reported in this review:

RR = 0.90 [CI: 0.87 - 0.93].

This estimate was based on studies undertaken in countries with high malaria endemicity, between 1957 and 2008: 3 trials in Nigeria, 3 in The Gambia, 3 in Kenya, 2 in Mozambique, 2 in Uganda, 1 in Cameroon, 1 in Burkina Faso, 2 in Thailand. Malaria prevalence in these countries across this period is approximately 35%.[31] Since the treatment was prophylactic, it was given to all women in the intervention group, regardless of their malarial status. This effect size is therefore an average of no-effect for the 65% of women without malaria, and a higher effectiveness for those women in the treatment group who did have malaria. The higher effectiveness can be calculated as follows:

RR = 1 – (1- 0.90)/0.35
= 0.71 [CI: 0.63 – 0.80]

We modelled this relative rate as the normal distribution $N(\mu = 0.71, \sigma = 0.045)$. To adjust intervention effectiveness for country-specific malaria prevalence, this higher efficacy is applied to the subpopulation of pregnant women with malaria at the time of treatment, while the intervention is modelled to have no effect in the remainder of women Note however, the cost is calculated based on the total number of pregnant women receiving treatment, not just the subset with malaria.

Maximum intervention coverage: The WHO's Roll Back Malaria initiative suggests that high levels of IPTp-SP coverage are achievable,[32] with countries like Ghana achieving coverage levels in excess of 50%. Poor exposure to antenatal care is considered a primary barrier to coverage of the IPTs-SP intervention.[33] We therefore set the maximum currently feasible coverage to be the proportion of pregnancies in each country that received antenatal care from a care provider (i.e., a doctor, midwife, nurse or auxiliary nurse, health worker, or traditional birth attendant), as reported by the Demographic and Health Surveys (DHS) Program (Figure S6).[34] Where data were unavailable, they were extrapolated from countries that belonged to the same OECD region, and shared the same income level. For countries without such double-matched criteria, we first searched for a country from the same region, then from the same income level, and finally we used the global average of measured values.

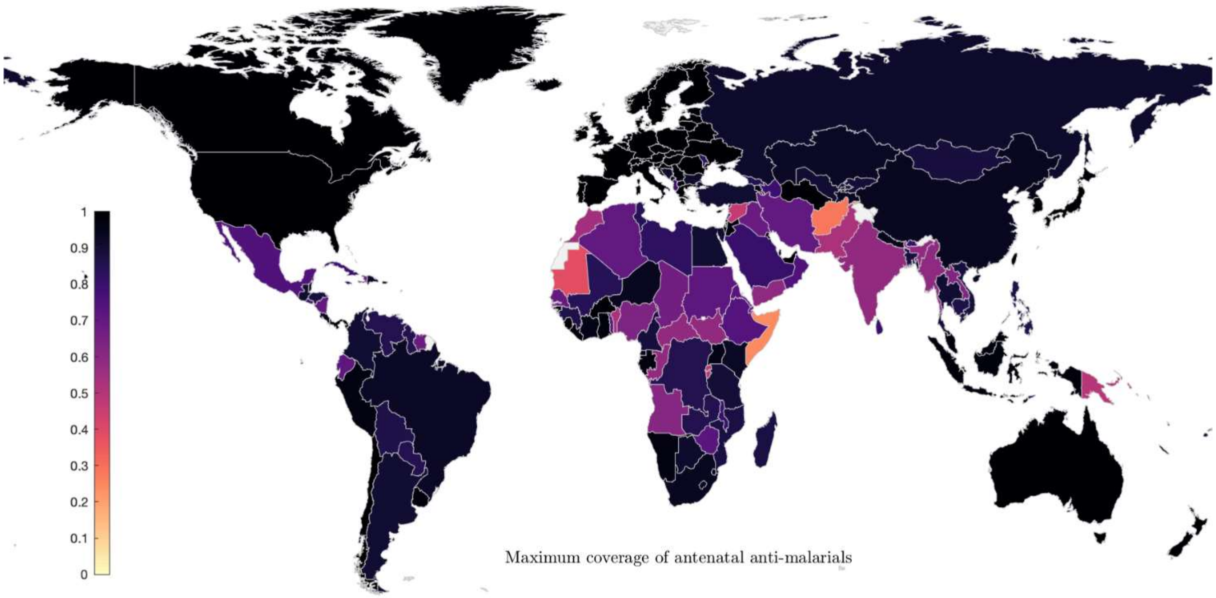

Figure S6: Estimated maximum feasibly achievable coverage for IPTp-SP*, as a proportion, across all modelled countries. Data are based on the level of antenatal care available in each country.*

Current intervention coverage: To estimate current levels of coverage for this intervention, we estimated the percentage of women with a live birth in the past two years who took three or more doses of sulfadoxine-pyrimethamine during pregnancy. Data on these rates were taken from the most recent reports from DHS and the Malaria Indicator Survey programme. For those countries with missing data, we only interpolated missing values if there were other countries in the dataset that shared both the same income level and regional classification. If no such countries exist, we assumed that the current level of coverage was zero (Figure S7).

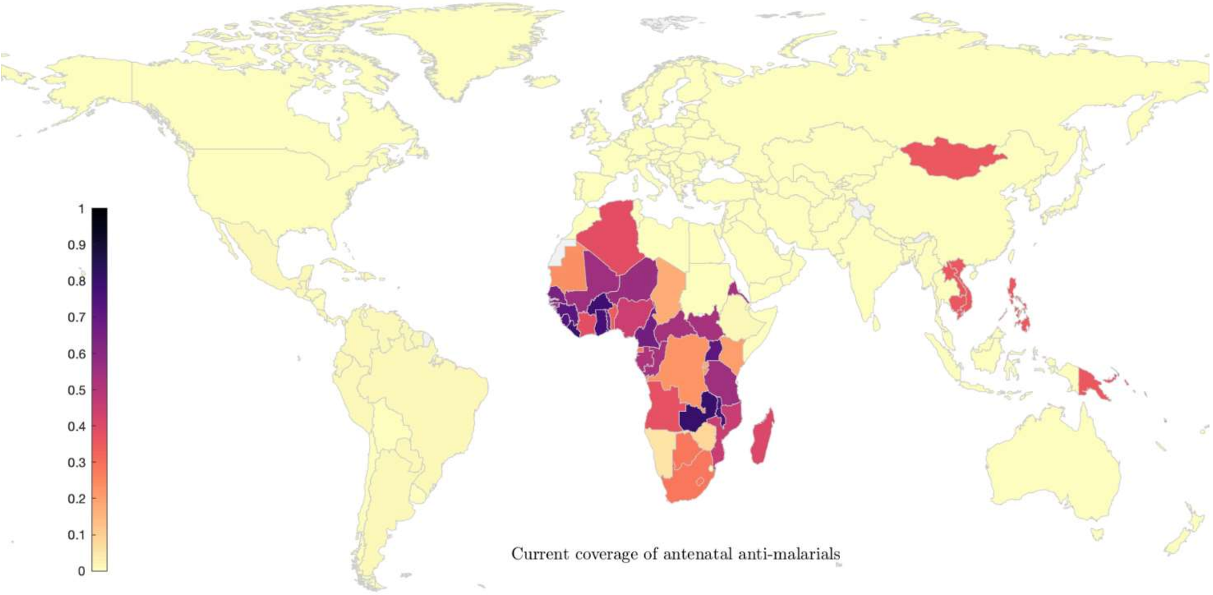

Figure S7: Estimated current coverage for antenatal preventative antimalarials*, as a proportion, across all modelled countries.*

2.3.3 Iron supplementation for pregnant women

According to WHO recommendation on antenatal care,[35] daily oral iron and folic acid supplementation is recommended for pregnant women to prevent maternal anaemia (as well as puerperal sepsis, low birth weight, and preterm birth). The WHO recommends that, where the prevalence of anaemia in pregnant women is high (40% or more), supplementation should continue for 3 months in the postpartum period. This effectively increases the pregnancy rate in the population by 33%.

A 30mg to 60mg dose of elemental iron, and a 400µg (0.4 mg) dose of folic acid is recommended. In settings where anaemia in pregnant women is a severe public health problem (i.e. where at least 40% of pregnant women have a blood haemoglobin [Hb] concentration < 110 g/L), a daily dose of 60 mg of elemental iron is preferred over a lower dose. If a woman is diagnosed with anaemia during pregnancy, her daily elemental iron should be increased to 120 mg until her Hb concentration rises to normal (Hb 110 g/L or higher). Thereafter, she can resume the standard daily antenatal iron dose to prevent recurrence of anaemia. In settings with a lower burden of anaemia (<20%) supplementation with 120 mg of elemental iron and 2800 mcg of folic acid once weekly is recommended if daily iron is not acceptable due to side-effects.

Compared to supplements without iron, or no treatment/placebo (no iron or placebo), a Cochrane systematic review[36] in 2024 of 14 trials concluded that iron supplementation during pregnancy may reduce the prevalence of maternal anaemia at term by:

$$RR = 0.30 \text{ [CI: } 0.20 - 0.47].$$

We modelled this probability distribution as a beta distribution with parameters $\text{Beta}(\alpha = 22.6, \beta = 47.2)$.

Maximum intervention coverage: We set the maximum feasible coverage to be the proportion of pregnancies in each country that received antenatal care from a clinical professional, as noted above (Figure S8).

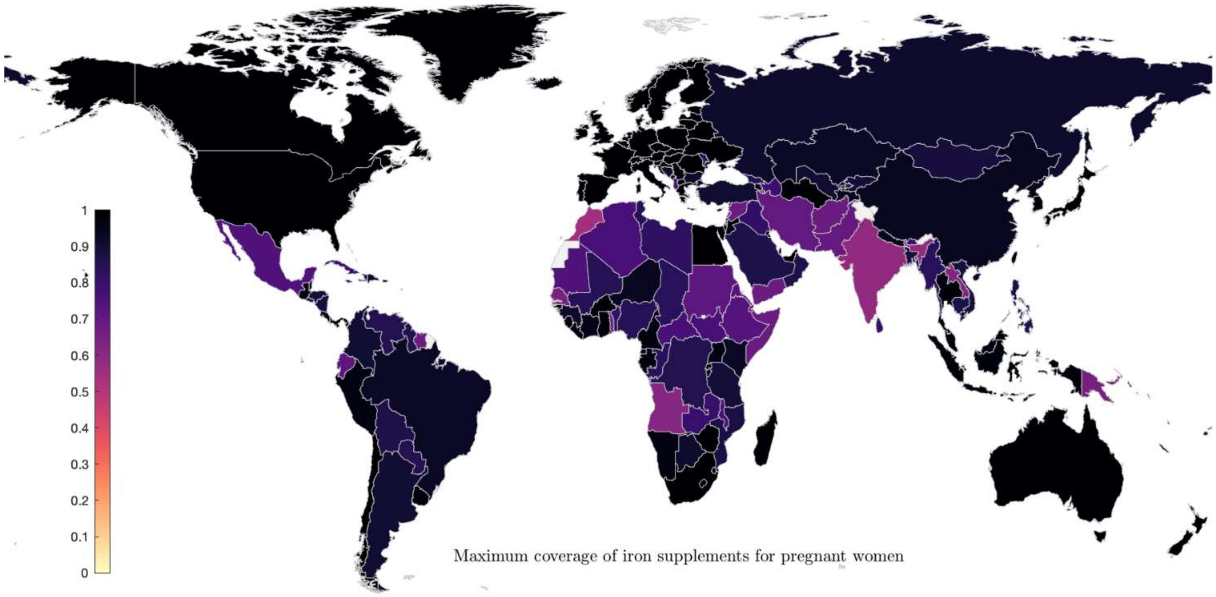

Figure S8: Estimated maximum feasible coverage for iron supplementation in pregnant women*, as a proportion, across all modelled countries. Data are based on the coverage of antenatal care in each country.*

Current intervention coverage: The DHS Program collects data on whether pregnant women received iron tablets or syrup during antenatal care (Table S1). We considered an individual to be currently covered by the intervention if they reported receiving *any* iron supplementation. While this category includes individuals who received lower than the WHO recommended dose, it also includes individuals who received the recommended dose but did not report or recall the specifics (Figure S9).

For LMIC countries without data, we interpolated from the set of most similar countries in the DHS dataset based on region and income grouping classification. In high income countries (as defined by OECD), we assumed that current iron supplementation coverage was 81% based on evidence from Denmark and Sweden.[37,38]

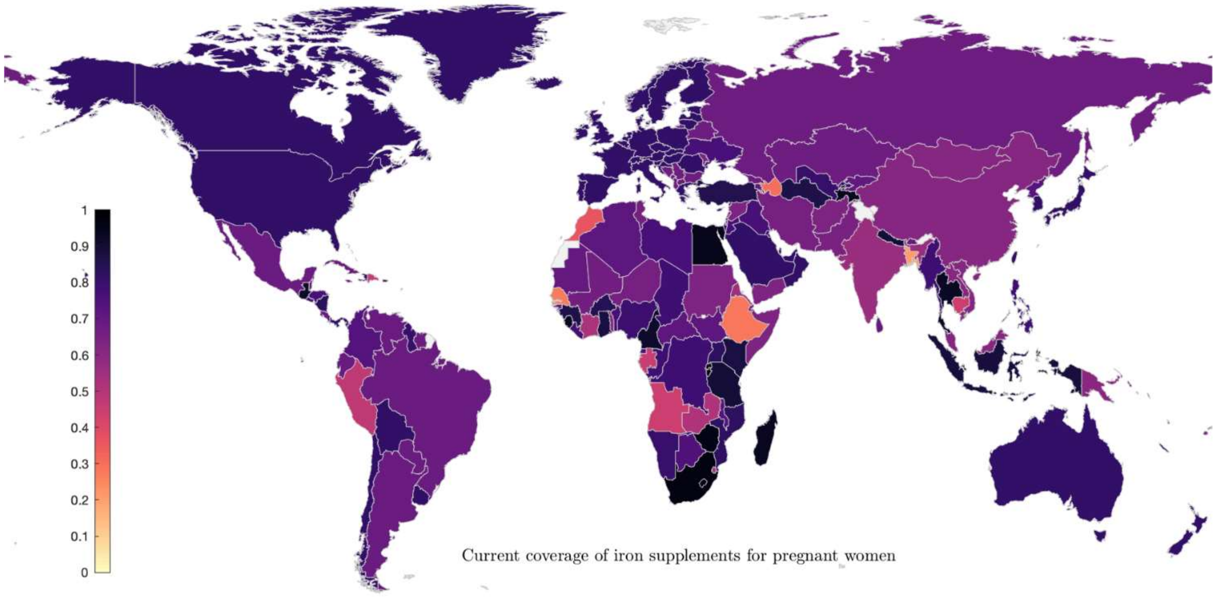

***Figure S9: Estimated current coverage for iron supplementation for pregnant women**, as a proportion, across all modelled countries. Data are based on the level of antenatal care available in each country.*

Indicator	Antenatal care content: Received iron tablets or syrup
Measure	Percent
Definition	Percentage of women with a live birth (or stillbirth) in the two (or three/five) years preceding the survey who received iron tablets or syrup during antenatal care
Denominator	Women who had a live birth (or stillbirth) in the two (or three/five) years preceding the survey
Group	Reproductive Health: Components of antenatal care
Type	Indicator
Decimals	1
Indicator ID	RH_ANCC_W_IRN

Table S1: DHS survey data used to estimate current coverage of iron supplementation for pregnant women.

2.3.4 Iron supplementation for non-pregnant women of reproductive age

According to recommendation in the WHO Guidelines,[39,40] daily iron supplementation (containing 30 – 60 mg elemental iron) is recommended as a public health intervention in menstruating adult women and adolescent girls where the prevalence of anaemia is greater than 40%. In settings with a lower prevalence of anaemia (20-40%) weekly dosing with 60 mg of iron and 2800 mcg of folic acid three times per week is recommended for 3 months on then 3 months off before recommencing supplementation. While the WHO guidelines are specific about the frequency of the supplements and the prevalence thresholds, we used the effect size for daily iron supplementation, regardless of their anaemia prevalence. This approach is a limitation as it may overestimate the effectiveness this intervention in settings where women receive weekly dosing, which has an RR of 0.65 according to a 2019 Cochrane systematic review.[41]

Maximum intervention coverage: There are limited data or proxy indicators to inform assumptions of maximum feasible coverage of iron supplementation in this population. We therefore assumed that the maximum coverage of iron supplementation for non-pregnant women of reproductive age would be as high, but no higher, than the maximum coverage for antenatal iron supplementation (Figure S10).

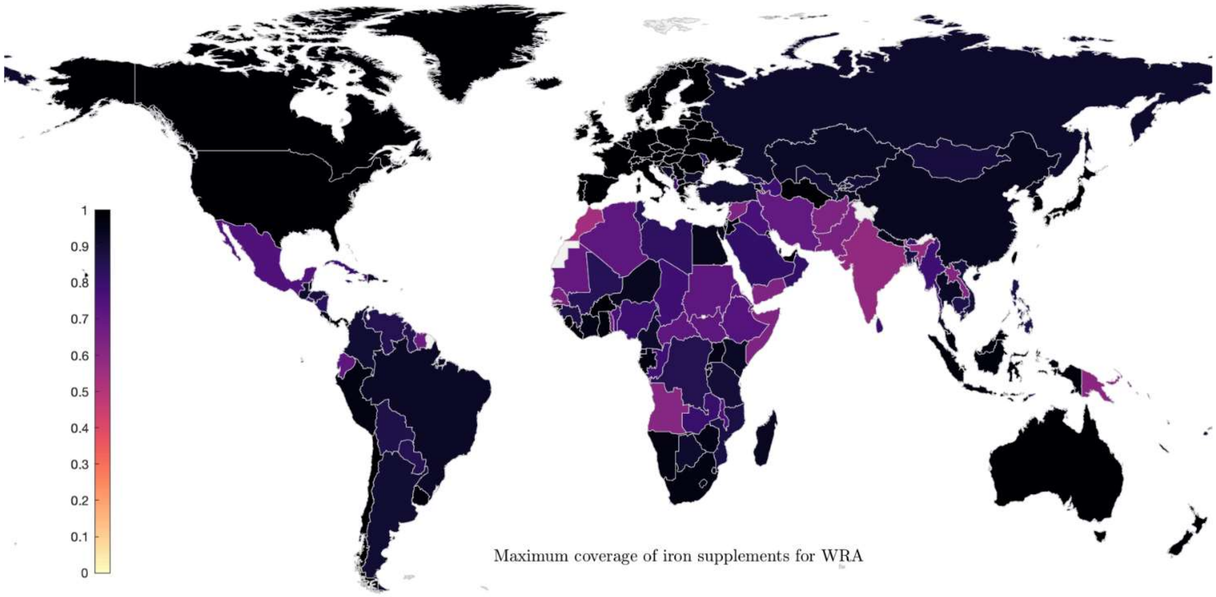

Figure S10: Estimated maximum achievable coverage for iron supplementation for women of reproductive age (WRA).

Current intervention coverage: There were no multi-country datasets available on the current coverage of iron supplementation (intermittent or daily) for non-pregnant women of reproductive age

Non-pregnant women are significantly less likely to take dietary supplements than pregnant women. According to data from the U.S. National Health and Nutrition Examination Survey (NHANES), rates of dietary supplement usage among non-pregnant and non-lactating women (45%) are lower than rates among pregnant or lactating women (77% and 70% respectively).[42] We therefore estimated current coverage of iron supplements for non-pregnant women of reproductive age to be 61% $(= 0.45/0.735)$ of the current coverage of antenatal iron supplementation (Figure S11).

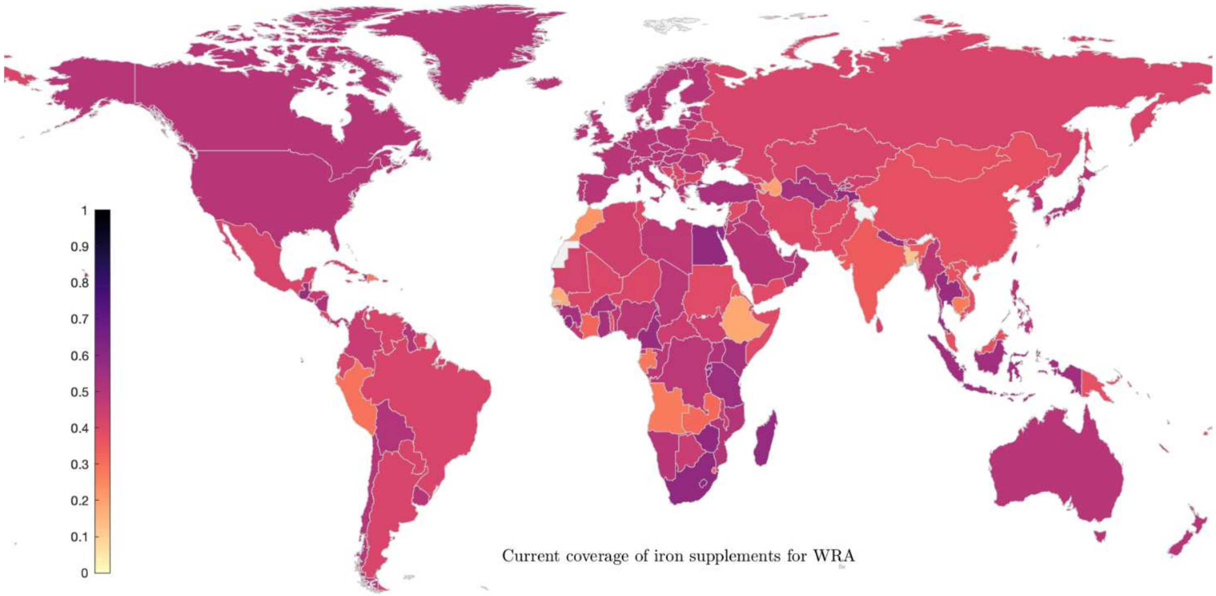

Figure S11: Estimated current coverage for iron supplementation for women of reproductive age (WRA).

SUPPORTING INFORMATION 3: COST AND COST-EFFECTIVENESS THRESHOLD ESTIMATION

For full details of our approach to estimating intervention costs, see Oliver et al.[43] Briefly, a formal health care sector perspective was adopted, which includes costs incurred by all payers, including government, patients and third-party payers. Data to estimate costs were drawn from secondary sources. Where country-specific data were not available, we used a population-weighted average of data from equivalent region and income classification sub-groups, based on WHO regions and World Bank income classifications.

All costs were reported in 2023 USD, with adjustments for inflation and currency conversions made as outlined by Turner and colleagues.[44]

3.1 Iron supplementation and IPTp-SP

Cost categories were defined in line with WHO Choosing Interventions that are Cost-Effective (WHO-CHOICE) methodology and included patient-level costs and program-level costs.[45] Patient-level costs are those incurred at the point of delivery of the intervention and include the commodity costs (e.g., cost of drugs and supplements); the costs associated with commodity supply chains; and the costs of the service delivery channels through which beneficiaries access commodities (e.g., health facility visits). Program-level costs capture the costs associated with delivering the interventions that are incurred at an ‘administrative level’ that is outside the point of delivery. Program-level costs include a combination of start-up and recurrent costs, such as training, administration, and community mobilisation. An ingredients-based approach was used to derive unit cost, where the quantities (q) and prices (p) of inputs comprising each cost of each component were determined and final costs calculated as $q \times p$.

Commodity quantities (i.e. number of tablets) were based on WHO-recommended dosing schedules for each intervention in each target group (Table S1). Commodity prices in LMICs were taken from the International Medical Products Guide. In high income countries, dose prices were taken from a combination of private market prices and government procurement pricing databases.

Table S1: Interventions, target populations and dosing schedules included in the analysis.

Intervention	Target population	Setting and dosing schedule
Iron and folic acid supplementation*	Menstruating women	*Where anaemia prevalence is 20-40%:* 60 mg of iron and 2800 mcg of folic acid three times per week for 3 months on then 3 months off before recommencing supplementation.[19]<br>*Where anaemia prevalence ≥40%:* 60 mg of elemental iron and 400 mcg of folic acid daily.[20]
	Pregnant women	*Where anaemia prevalence is <20%:* 60 mg of elemental iron and 400 mcg of folic acid daily or intermittent oral iron and folic acid supplementation with 120 mg of elemental iron and 2800 mcg of folic acid once weekly if daily iron is not acceptable due to side-effects.[18]<br>*Where anaemia prevalence ≥20%:* 60 mg of elemental iron and 400 mcg of folic acid daily.[18]

Intermittent preventive treatment of malaria in pregnancy (IPTp)	Pregnant women	*In malaria endemic settings:* Three doses of Sulfadoxine (1500 mg) and Pyrimethamine (75 mg).[22]

*In all settings, we assume a proportion of women are diagnosed with anaemia and are treated with daily iron (120 mg) and folic acid (400 mcg) for three months before commencing on the dosing schedule outlined here.

Commodity costs were inflated by region-specific mark-ups published by WHO-CHOICE to include the costs associated with transport of goods.

Quantities and prices of service delivery inputs were estimated as follows. Interventions targeting pregnant women were assumed to be delivered through a combination of antenatal care (ANC) visits (assuming approximately 2 minutes of a 10 minute visit) and community health worker visits (assuming a 5 minute visit). For interventions targeting non-pregnant women we assumed delivery through a combination of health centre visits in which treatment was the subject of the entire visit, community health worker visits and private pharmacies. The cost of each health facility visits was based on WHO-CHOICE outpatient visit unit costs.[46] The economic costs of community health worker visits were valued based using country-specific minimum wage estimates from the International Labour Organization. For iron supplementation, the per person cost of a point of care diagnostic test for anaemia was estimated to be 0.50 USD based on a costing analysis conducted in India and added to the service delivery cost.[47]

Program costs were drawn from a study by Baltussen et al, which modelled the start-up and ongoing costs of implementing a 10-year program of iron supplementation in four subregions based on WHO-CHOICE methodology in the estimation of resource use and the associated costs.[48]

3.2 Staple food fortification

Costs of wheat and maize flour fortification were estimated based on data from a comprehensive modelling study that estimated the total public and private sector costs associated with a national fortification program.[49] Total costs were divided by the population in the year of the study to derive a unit cost per person. Unit costs of rice fortification were drawn from a study in which private sector costs were estimated and reported as incremental cost to consumers per person per year in four countries with different industry structures, fortification methods, and macroeconomic contexts.[50]

3.3 Cost uncertainty

Upper and lower estimates of unit costs were derived based on an 'extreme scenario analysis',[51] in which lower estimates for each cost component (e.g., lower estimate of commodity costs, lower estimate of service delivery costs etc) were summed to give the lowest possible unit cost, and likewise for the higher estimates. This analysis will represent an overestimate of the true uncertainty as the value of individual cost components are not correlated in their variance. See Oliver et al[43] for more detail on how upper and lower estimates were calculated for each cost component.

3.4 Cost-effectiveness thresholds (CETs)

CETs were used to represent the point at which the costs of an intervention are considered an efficient use of resources, or not a good value investment for benefits gained. The value of these thresholds are sometimes based on explicit 'willingness to pay' thresholds, which represent the price that consumers, funders or healthcare agencies are willing to pay for each unit of benefit (e.g., a year of life in good health) gained.

However, country-specific willingness to pay thresholds are rarely explicitly stated. The WHO Commission on Macroeconomics and Health has previously suggested that an intervention may be considered very cost-effective when it costs less than one times the per capita GDP per DALY,[52] based on the conservative assumption that each DALY averted returns the equivalent of one GDP per capita into the economy. More recently, the Lancet Commission on Investing in Health called for a 'full income' approach, where the inherent value of good health is recognised beyond the contribution to national income and suggested that the value of a year of life is 2.3 times the per capita GDP in LMIC and 1.4 times GDP in high income countries.[53] While these approaches to setting a CET centre on quantifying the value of a year of life, they do not consider the reality that resources for health are finite and expenditure in one area can result in forgone benefits when those resources are not available to be invested elsewhere (opportunity costs). Recent studies have estimated CETs based on the opportunity costs of investing to avert one DALY, with thresholds generally much lower than those previously proposed (e.g. 0.24 times GDP in low-income countries).[54-56]

In the base case analysis, we set CETs at a level comparable to the opportunity costs of expenditure by drawing upper and lower estimates from a recent study where thresholds for 174 countries were estimated.[55] For countries where CETs were not reported, we used median thresholds reported for the relevant income classification. The results from this analysis might reasonably be considered targets that could be achieved within the constraints of domestic health budgets. In a separate analysis, we explored anaemia reduction targets that would be possible when the value of a year in good health is the basis for setting CETs (regardless of the opportunity costs of that investment), by using one GDP per capita as the lower estimate and estimates from a full income approach as the upper estimate. The results from this analysis may reflect targets that would require external financing to support anaemia reduction targets.

SUPPORTING INFORMATION 4: SIMULATION METHODS

4.1 Objective and approach

To determine what reduction in anaemia can be achieved within the bounds of investments that can be considered good value for money, we developed a globally uniform national-level health economic model. The model was based around the approach outlined in Disease Control Priorities, 3rd Edition (DCP3),[57] which models the economic costs and health consequences of a different packages of interventions. Formally, our analysis is known as a cohort component projection model (CCPM). The CCPM integrates the data outlined above on anaemia prevalence, severity, and intervention effectiveness, with estimates on intervention costs and country-specific CETs for DALYs averted. The method uses Monte Carlo simulations to account for uncertainty while projecting the reduction in anaemia prevalence that would result from increasing the coverage of a set of evidence-based interventions.

We proposed the anaemia reduction target for each country by identifying the combination of interventions that is both maximally effective in reducing anaemia prevalence, and good value for money, based on each country's CET. Mathematically, this involves finding a boundary solution to a constrained optimisation problem, where the objective function maximises the reduction in anaemia prevalence while the constraints reflect the country's CET. Operationally, we use an iterative, greedy search algorithm to approximate the optimal mix of interventions within the feasible cost-effectiveness space.

4.2 Modelling the effect of increases in intervention coverage

The model estimates the effect of anaemia-focused interventions on (1) the prevalence of mild, moderate, and severe anaemia, which we denote as $\{s_m, s_d, s_s\}$. These results allow us to also estimate the effect on (2) the overall burden of the disease, which we denote as B. Prevalence is the metric used by both the SDG and GNT targets, and current estimates exist for almost all countries (Figure S1). The burden of anaemia,

measured in DALYs, is based on the weighted sum of the prevalence of the three severity types, and their disability weights (w_m, w_d, and w_s for mild, moderate and severe anaemia respectively).

We begin with the estimates of current (2021) anaemia prevalence made by the GBD project,[4] which we denote P_1. We consider this prevalence to be the result of (1) the country's "underlying" prevalence P_0, and (2) the current levels of coverage for each of the interventions currently being applied to the population of women of reproductive age in that country, which we denote m_1^i for intervention i.

Note that because the effectiveness of each of our interventions is the same for the prevalence of the three different levels of severity, without loss of generality we can consider only overall prevalence in our models and assume that the distribution among severity types remains the same. An x% reduction in overall prevalence results in an x% reduction in burden.

To estimate the impact on anaemia prevalence of *increasing* the levels of intervention coverage from m_1^i to m_2^i, we calculate:

$$P_2 = P_1 \frac{1 - m_2^i(1 - R_i)}{1 - m_1^i(1 - R_i)}.$$

[Equation S1]

where R_i is the effectiveness of intervention i, as estimated in section **Supporting Information 2** above.

Given that $0 \geq m_2^i \geq m_1^i$, the fraction term on the right hand side of Equation 4 will be less than one, correctly predicting that $P_2 \leq P_1$ if $0 \leq R_i \leq 1$. We can relabel this term as:

$$\theta_i = \frac{1 - m_2^i(1 - R_i)}{1 - m_1^i(1 - R_i)}.$$

[Equation S2]

Effectively, Equation S1 takes the current prevalence of anaemia in a particular country and uses the denominator $1 - m_1^i(1 - R_i)$ to estimate what the prevalence would be in the absence of any application of intervention i. It then applies the numerator $1 - m_2^i(1 - R_i)$ to predict what the prevalence will be if the coverage of that intervention is increased to m_2^i.

We are considering increases in the coverage of multiple different interventions, not just one. If we accept the assumption that all interventions operate independently on anaemia, then we can repeatedly apply the technique shown in Equation 4 to the different interventions in turn:

$$P_2 = P_1 \prod_i \theta_i .$$

[Equation S3]

These equations can be directly applied for interventions that target all women of reproductive age – staple food fortification and universal iron supplementation. However, the other two interventions target only specific subsets of the population. Both iron supplementation and antenatal anti-malarials for pregnant women target the subset of pregnant women in the population, and the latter is only effective for the proportion of women who are malarial at the time of treatment.

To account for pregnancy, we combined fertility rates and stillbirth rates in each country. Fertility rates are defined by the number of live births each female is expected to deliver over her reproductive life. Stillbirth rates reflect the probability that each pregnancy may not be carried to term. We multiplied the probability of a stillbirth by the average duration of pregnancy prior to stillbirth (estimated as 0.65 years) and the fertility rate to obtain pregnancy duration leading to stillbirths. We then added this to the fertility rate multiplied by 0.75 years (the average duration of pregnancy) and multiplied the sum of these numbers by 34 reproductive years per woman. A limitation of this approach is that it assumes pregnancy is evenly distributed across reproductive age.

To estimate the consequent effect on the burden of disease in a given country, we calculate the change in overall prevalence, $\Delta(\boldsymbol{m}) = P_2 - P_1$ as a function of the set of intervention coverages $\boldsymbol{m}$. Given the original distribution of severities, we can calculate the benefit B in DALYs averted as:

$$B(\boldsymbol{m}) = \Delta(\boldsymbol{m})[s_m w_m + s_d w_d + s_s w_s].$$

[Equation S4]

4.3 Identifying the optimal package of interventions

Equation S3 allows us to estimate the prevalence of anaemia after the implementation of a package of interventions at enhanced coverage levels $\{m_2^1, m_2^2, m_2^3, m_2^4\}$. Based on our estimates of constant unit costs, the total additional cost C of this increased coverage would be:

$$C(\boldsymbol{m}) = N(m_2^1 - m_1^1)c_1 + N(m_2^2 - m_1^2)c_2 + N_P(m_2^3 - m_1^3)c_3 + N_P(m_2^4 - m_1^4)c_4,$$

[Equation S5]

where N and N_P are the populations of all women of reproductive age and pregnant women respectively, and c_x is the per-capita cost of delivering intervention x. See *Supporting Information 3* for details on cost estimation.

Identifying the optimal package of interventions, and thus the proposed target for each country, is a matter of comparing all possible values of $C(\boldsymbol{m})/B(\boldsymbol{m})$ for combinations of $\boldsymbol{m}$ where $C(\boldsymbol{m}) \leq T$, the CET threshold for that country. We have provided these interventions in order of cost-effectiveness by region in Table S2 below.

Table S2: League table ranking different anaemia reduction strategies by World Bank region. *The numbers and colour coding in each cell represent the order in which these interventions should be implemented, averaged across all 400 simulations and countries in that region-income combination.*

World Bank Region	Income Group	Fortification	Oral iron supplementation (Pregnant women)	Oral iron supplementation (WRA)	IPTp-SP
East Asia & Pacific	High income	1	2	3	4
	Upper middle income	1	2	3	4
	Lower middle income	1	3	2	4
Europe & Central Asia	High income	1	2	3	4
	Upper middle income	1	2	3	4
	Lower middle income	1	2	3	4
Latin America & Caribbean	High income	1	2	3	4
	Upper middle income	1	2	3	4
	Lower middle income	1	3	2	4
Middle East & North Africa	High income	2	1	3	4
	Upper middle income	1	2	3	4
	Lower middle income	1	2	3	4
	Low income	1	2	3	4
North America	High income	1	2	3	4
South Asia	Upper middle income	1	2	3	4
	Lower middle income	1	2	3	4
	Low income	1	2	3	4
Sub-Saharan Africa	High income	1	2	3	4
	Upper middle income	1	2	3	4
	Lower middle income	1	3	2	4
	Low income	1	2	3	4

Fortification: fortification with staple foods (wheat, rice, maize). WRA: Women of Reproductive Age. IPTp-SP: Intermittent Preventive Treatment with Sulfadoxine-Pyrimethamine.

4.4 Propagating parameter uncertainty through to results

Monte Carlo simulation is often used in economic analysis to sample from distributions of values. As it is computationally difficult to sample from some distributions, but comparably simple to generate pseudo-random numbers between 0 and 1, Monte Carlo simulation simply uses the latter as an input into the empirical cumulative distribution function of the former. In our simulation model, we propagated our uncertainty through to the results by using a single random sample from each input distribution per simulation. This method, known as probabilistic sensitivity analysis, is a means of expressing the potential variability in the final results (here, the final target for each country) due to uncertainty in the input parameters.

Relative rates for each intervention were all reported from meta-analyses that included uncertainty. Each individual country in our model contained separate and uncertain estimates for coverage and cost data for each intervention. We used the method of moments, from the ShinyPrior app developed by White & Blythe[58] to derive the distribution parameters used in the model. Input distributions were obtained by converting point estimates and confidence intervals to distribution parameters using their moments. We generally sampled from Gaussian distributions for normally distributed data with plausible tail values, Beta distributions for non-normally distributed values that were constrained between 0 and 1, and triangular distributions when parametric distributions did not adequately approximate our uncertainty. A full

description of each distribution is available in the code provided with the paper. If parameterisation for Beta distributions using the method of moments led to a distribution with different mean values, we first fit a triangular distribution based on the mean and 95% confidence intervals reported in the literature, then fit a Beta distribution to the triangular distribution using the R package fitdistrplus. We were unable to obtain uncertainty estimates for coverage data. We therefore added a perturbation to each point estimate equivalent to +/- 25%, distributed ~N(0, 0.127), to express uncertainty in these figures, limiting the resulting estimates to remain between [0, 1] inclusive.

4.5 Model assumptions and limitations

Rate of uptake: Our model predicts anaemia prevalence, following changes to interventions, several decades from the present time. Our primary interest is on the evaluation time, not the interim. We therefore assume that when this evaluation time arrives, any proposed interventions have had enough time to take full effect.

Changing demographics: Our focus is on women of reproductive age, but we further disaggregate this group into independent subgroups that either receive different treatments, and/or respond differently to a similar treatment. Many CCPMs include forecasts of population demographics, based on trends in all-cause mortality, fertility, and migration provided by datasets such as the World Population Prospects 2019. At present, we do not include these forecast changes. A simple method for including these dynamics is to use future projections of the population, rather than current projections.

Constant marginal costs: We assume marginal costs are constant with respect to both total coverage and expenditure. In reality, costs are likely to exhibit nonlinear relationships with coverage, with marginal costs being high at low and very high levels of coverage, e.g., when fixed costs are a large proportion of a program and because the program must focus on harder to reach population subgroups due to geographic remoteness or other challenges, respectively. However, empirical estimates of this relationship are rare and case-specific, and have not been undertaken for our interventions. This is a difficult feature to incorporate into a simulation model and we look forward to future research in this area.

4.6 Exploring structural uncertainty using scenario analyses

We examine the impact of structural assumptions on the estimated global anaemia reduction targets by conducting and evaluating several qualitatively different scenarios. The goal is to assess how markedly different modelling choices – such as removing our cost-effectiveness thresholds altogether – influence the results. The figures in this section illustrate the variability in global targets under these scenarios. Figure S12 presents the results of these scenarios, using violin plots to visualize the range of uncertainty around each target.

Figure S13 shows national-level targets under a scenario without any cost-effectiveness constraints, meaning interventions are applied up to their maximum feasible coverage, regardless of cost-effectiveness. Figure S14 explores a scenario where all interventions start from zero coverage, applying a cost-effectiveness threshold of 1 x GDP per capita.

These analyses demonstrate that cost-effectiveness constraints and existing intervention coverage play a significant role in determining achievable anaemia reduction targets. However, even the most optimistic scenarios fall well short of the 50% reduction goal set by global development frameworks. The results also indicate that including high-income countries increases the global target, while LMICs alone should aim for lower reductions, due to their economic and health system constraints.

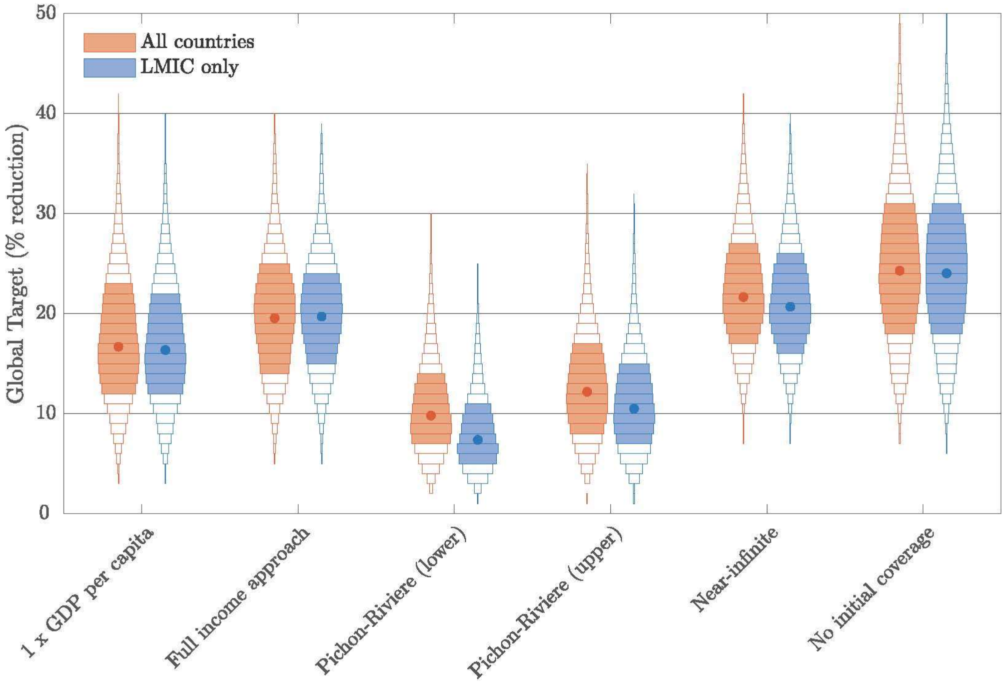

Figure S12: Sensitivity of global summary target to structural assumptions in the model. *Results show the global target, with uncertainty across Monte Carlo simulations shown using violin plots. Dots indicate the median target, and shaded areas indicate +/– 1 standard deviation. Five different scenarios are shown, summarised across all countries, and summarised across only countries classified as Lower or Middle Income Countries (LMIC). Five of the scenarios have different CET thresholds, and one scenario assumes zero initial coverage, with a CET threshold of 1 x GDP per capita.*

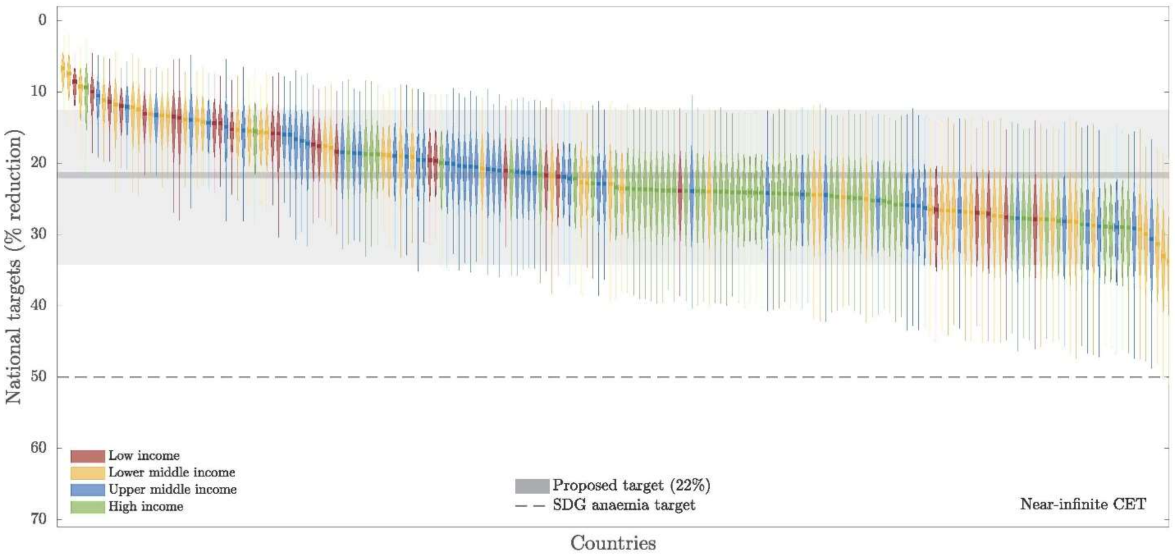

Figure S13: National anaemia reduction targets with no cost-effectiveness threshold (CET). *Bars show the recommended anaemia reduction targets for women of reproductive age in 191 countries, colour-coded by income level. Dots represent the mean target for each country, and vertical lines represent the effects of uncertainty in model parameters (e.g., intervention costs, effectiveness, coverage). Dashed line indicates the current Sustainable Development Goal (SDG) anaemia reduction target (50%). In this scenario, interventions are recommended, up to their maximum feasible coverage, regardless of their incremental cost-effectiveness ratio.*

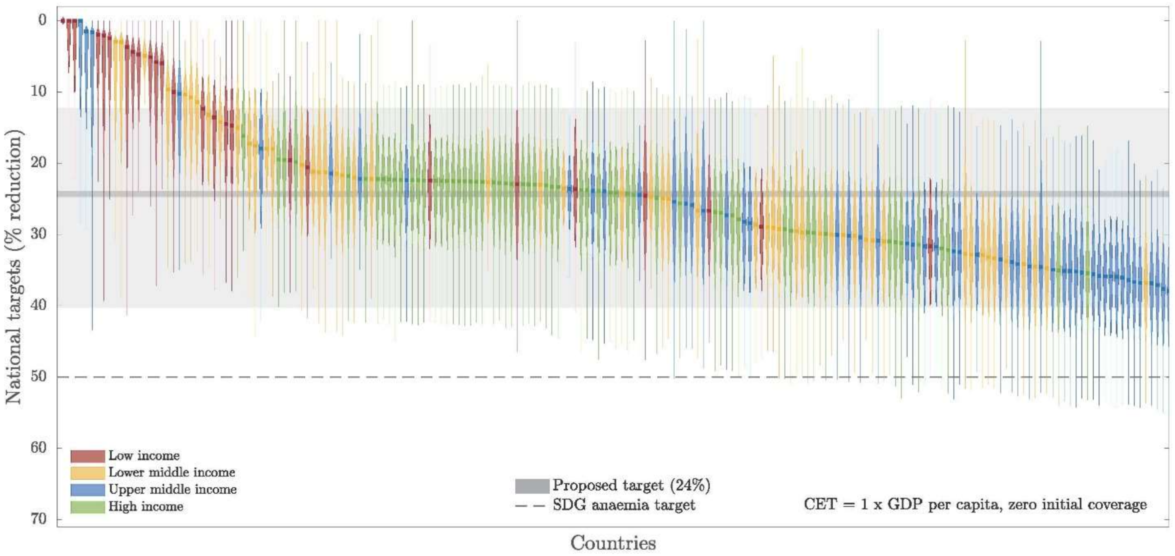

Figure S14: National anaemia reduction targets when we assumed that there was zero initial coverage for all interventions, and with a cost-effectiveness threshold (CET) set at 1 x GDP per capita. *Bars show the recommended anaemia reduction targets for women of reproductive age in 191 countries, colour-coded by income level. Dots represent the mean target for each country, and vertical lines represent the effects of uncertainty in model parameters (e.g., intervention costs, effectiveness, coverage). Dashed line indicates the current Sustainable Development Goal (SDG) anaemia reduction target (50%).*

REFERENCES CITED IN SUPPORTING INFORMATION 1 – 4

1. R Core Team. R: A language and environment for statistical computing. Vienna, Austria: R Foundation for Statistical Computing; 2022.
2. Arel-Bundock V, Enevoldsen N, Yetman C. countrycode: An R package to convert country names and country codes. *Journal of Open Source Software* 2018; **3**(28): 848.
3. Millard SP. EnvStats: an R package for environmental statistics: Springer Science & Business Media; 2013.
4. Gardner WM, Razo C, McHugh TA, et al. Prevalence, years lived with disability, and trends in anaemia burden by severity and cause, 1990–2021: findings from the Global Burden of Disease Study 2021. *The Lancet Haematology* 2023; **10**(9): e713-e34.
5. Kassebaum NJ, Jasrasaria R, Naghavi M, et al. A systematic analysis of global anemia burden from 1990 to 2010. *Blood* 2014; **123**(5): 615-24.
6. Vardell E. Global Health Observatory Data Repository. *Med Ref Serv Q* 2020; **39**(1): 67-74.
7. UN Inter-agency Group for Child Mortality Estimation. Child Mortality Estimates: Country-specific Stillbirth rate. Data and Analytics Section; Division of Data, Analytics, Planning and Monitoring, UNICEF; 2023.
8. Battle KE, Lucas TCD, Nguyen M, et al. Mapping the global endemicity and clinical burden of Plasmodium vivax, 2000-17: a spatial and temporal modelling study. *Lancet* 2019; **394**(10195): 332-43.
9. Weiss DJ, Lucas TCD, Nguyen M, et al. Mapping the global prevalence, incidence, and mortality of Plasmodium falciparum, 2000-17: a spatial and temporal modelling study. *Lancet* 2019; **394**(10195): 322-31.
10. Shekar M, Kakietek J, Dayton Eberwein J, Walters D. An Investment Framework for Nutrition: Reaching the Global Targets for Stunting, Anemia, Breastfeeding, and Wasting. Washington, DC: World Bank; 2017.
11. World Health Organization. e-Library of Evidence for Nutrition Actions (eLENA). 2024. https://www.who.int/tools/elena/interventions (accessed 28 January 2025.
12. World Health Organization. WHO policy brief for the implementation of intermittent preventive treatment of malaria in pregnancy using sulfadoxine-pyrimethamine (IPTp-SP): World Health Organization, 2014.
13. World Health Organization. Guideline: preventive chemotherapy to control soil-transmitted helminth infections in at-risk population groups. 2017.
14. Keats EC, Haider BA, Tam E, Bhutta ZA. Multiple-micronutrient supplementation for women during pregnancy. *Cochrane Database Syst Rev* 2019; **3**(3): CD004905.
15. Kilbourne AM, Neumann MS, Pincus HA, Bauer MS, Stall R. Implementing evidence-based interventions in health care: application of the replicating effective programs framework. *Implement Sci* 2007; **2**: 42.
16. Evans RE, Craig P, Hoddinott P, et al. When and how do 'effective' interventions need to be adapted and/or re-evaluated in new contexts? The need for guidance. *J Epidemiol Community Health* 2019; **73**(6): 481-2.
17. Milat A, Lee K, Conte K, et al. Intervention Scalability Assessment Tool: A decision support tool for health policy makers and implementers. *Health Res Policy Syst* 2020; **18**(1): 1.
18. Sutherland RL, Jackson JK, Lane C, et al. A systematic review of adaptations and effectiveness of scaled-up nutrition interventions. *Nutr Rev* 2022; **80**(4): 962-79.
19. von Klinggraeff L, Pfledderer CD, Burkart S, et al. Are the Risk of Generalizability Biases Generalizable? A Meta-Epidemiological Study. *Res Sq* 2024.
20. World Health Organization. Fortification of maize flour and corn meal with vitamins and minerals. 2016.
21. World Health Organization. Guideline: fortification of rice with vitamins and minerals as a public health strategy: World Health Organization; 2018.

22. World Health Organization. Guideline: Fortification of wheat flour with vitamins and minerals as a public health strategy. 2022.
23. Field MS, Mithra P, Pena-Rosas JP. Wheat flour fortification with iron and other micronutrients for reducing anaemia and improving iron status in populations. *Cochrane Database Syst Rev* 2021; **1**(1): CD011302.
24. Food Fortification Initiative. Global Progress. (accessed 03/02/2025 2025).
25. Food and Agriculture Organization of the United Nations (FAO). Food balances (2010-). *Food and Agriculture Organization of the United Nations* 2019.
26. World Health Organization. Report of the eighth meeting of the WHO Strategic and Technical Advisory Group of Experts for Maternal, Newborn, Child and Adolescent Health and Nutrition, 14-16 November 2023: World Health Organization; 2024.
27. Global Fortification Data Exchange. 2023. http://www.fortificationdata.org (accessed 10 July 2024.
28. Galetti V, Stoffel NU, Sieber C, Zeder C, Moretti D, Zimmermann MB. Threshold ferritin and hepcidin concentrations indicating early iron deficiency in young women based on upregulation of iron absorption. *eClinicalMedicine* 2021; **39**.
29. World Health Organization. WHO Guidelines for malaria. Geneva; 2022.
30. Kayentao K, Garner P, van Eijk AM, et al. Intermittent preventive therapy for malaria during pregnancy using 2 vs 3 or more doses of sulfadoxine-pyrimethamine and risk of low birth weight in Africa: systematic review and meta-analysis. *Jama* 2013; **309**(6): 594-604.
31. Snow RW, Sartorius B, Kyalo D, et al. The prevalence of Plasmodium falciparum in sub-Saharan Africa since 1900. *Nature* 2017; **550**(7677): 515-8.
32. Nafo Traoré F. Rolling back malaria: opportunities and challenges. *Transactions of The Royal Society of Tropical Medicine and Hygiene* 2005; **99**(6): 403-6.
33. Yaya S, Uthman OA, Amouzou A, Bishwajit G. Use of Intermittent Preventive Treatment among Pregnant Women in Sub-Saharan Africa: Evidence from Malaria Indicator Surveys. *Trop Med Infect Dis* 2018; **3**(1).
34. Mbonye AK, Bygbjerg I, Magnussen P. Intermittent preventive treatment of malaria in pregnancy: evaluation of a new delivery approach and the policy implications for malaria control in Uganda. *Health Policy* 2007; **81**(2-3): 228-41.
35. World Health Organization. WHO recommendations on antenatal care for a positive pregnancy experience: World Health Organization; 2016.
36. Finkelstein JL, Cuthbert A, Weeks J, et al. Daily oral iron supplementation during pregnancy. *Cochrane Database Syst Rev* 2024; **8**(8): CD004736.
37. Knudsen VK, Hansen HS, Ovesen L, Mikkelsen TB, Olsen SF. Iron supplement use among Danish pregnant women. *Public Health Nutr* 2007; **10**(10): 1104-10.
38. Wulff M, Ekström EC. Iron supplementation during pregnancy in Sweden: to what extent is the national recommendation followed? *Acta Obstet Gynecol Scand* 2003; **82**(7): 628-35.
39. World Health Organization. Guideline: intermittent iron and folic acid supplementation in menstruating women: World Health Organization; 2011.
40. World Health Organization. Daily iron supplementation in adult women and adolescent girls. *Geneva: World Health Organization* 2016; **33**.
41. Fernandez-Gaxiola AC, De-Regil LM. Intermittent iron supplementation for reducing anaemia and its associated impairments in adolescent and adult menstruating women. *Cochrane Database Syst Rev* 2019; **1**(1): CD009218.
42. Jun S, Gahche JJ, Potischman N, et al. Dietary Supplement Use and Its Micronutrient Contribution During Pregnancy and Lactation in the United States. *Obstet Gynecol* 2020; **135**(3): 623-33.
43. Oliver VL, Wang Y, Leung S, et al. Estimated unit costs of anaemia interventions for women of reproductive age in 193 United Nations member states: A costing study. *Lancet Haematology* 2025 (in press).

44. Turner HC, Lauer JA, Tran BX, Teerawattananon Y, Jit M. Adjusting for inflation and currency changes within health economic studies. *Value in Health* 2019; **22**(9): 1026-32.
45. Bertram MY, Lauer JA, Stenberg K, Edejer TTT. Methods for the Economic Evaluation of Health Care Interventions for Priority Setting in the Health System: An Update From WHO CHOICE. *Int J Health Policy Manag* 2021; **10**(11): 673-7.
46. Adam T, Evans DB, Murray CJ. Econometric estimation of country-specific hospital costs. *Cost effectiveness and resource allocation* 2003; **1**: 1-10.
47. Neogi SB, John D, Sharma J, et al. Cost-effectiveness of point-of-care devices for detection of anemia in community settings in India. *Clinical Epidemiology and Global Health* 2022; **14**.
48. Baltussen R, Knai C, Sharan M. Iron fortification and iron supplementation are cost-effective interventions to reduce iron deficiency in four subregions of the world. *J Nutr* 2004; **134**(10): 2678-84.
49. Fiedler JL, Sanghvi TG, Saunders MK. A review of the micronutrient intervention cost literature: program design and policy lessons. *The International journal of health planning and management* 2008; **23**(4): 373-97.
50. Alavi S, Bugusu B, Cramer G, et al. Rice fortification in developing countries: a critical review of the technical and economic feasibility. 2008.
51. Briggs AH. Handling uncertainty in cost-effectiveness models. *Pharmacoeconomics* 2000; **17**(5): 479-500.
52. World Health Organization. Macroeconomics and health : investing in health for economic development / report of the Commission on Macroeconomics and Health. Geneva: World Health Organization; 2001.
53. Jamison DT, Summers LH, Alleyne G, et al. Global health 2035: a world converging within a generation. *Lancet* 2013; **382**(9908): 1898-955.
54. Ochalek J, Lomas J, Claxton K. Estimating health opportunity costs in low-income and middle-income countries: a novel approach and evidence from cross-country data. *BMJ Glob Health* 2018; **3**(6): e000964.
55. Pichon-Riviere A, Drummond M, Palacios A, Garcia-Marti S, Augustovski F. Determining the efficiency path to universal health coverage: cost-effectiveness thresholds for 174 countries based on growth in life expectancy and health expenditures. *Lancet Glob Health* 2023; **11**(6): e833-e42.
56. Woods B, Revill P, Sculpher M, Claxton K. Country-Level Cost-Effectiveness Thresholds: Initial Estimates and the Need for Further Research. *Value Health* 2016; **19**(8): 929-35.
57. Jamison DT. Disease control priorities: improving health and reducing poverty. *The Lancet* 2018; **391**(10125): e11-e4.
58. White N, Blythe R. ShinyPrior: A tool for estimating probability distributions using published evidence. *OSF Preprints* 2023; **10**.

Supporting Information 5: CHEERS 2022 Checklist

Topic	No.	Item	Location where item is reported
**Title**			
	1	Identify the study as an economic evaluation and specify the interventions being compared.	NA – While this study uses cost-effectiveness methodology, the primary purpose is to describe a novel approach to global health target-setting
**Abstract**			
	2	Provide a structured summary that highlights context, key methods, results, and alternative analyses.	Abstract
**Introduction**			
**Background and objectives**	3	Give the context for the study, the study question, and its practical relevance for decision making in policy or practice.	Background section
**Methods**			
**Health economic analysis plan**	4	Indicate whether a health economic analysis plan was developed and where available.	Not developed

Topic	No.	Item	Location where item is reported
**Study population**	5	Describe characteristics of the study population (such as age range, demographics, socioeconomic, or clinical characteristics).	Methods – Overview
**Setting and location**	6	Provide relevant contextual information that may influence findings.	Methods – Overview
**Comparators**	7	Describe the interventions or strategies being compared and why chosen.	Methods – Data, selection of interventions.<br>Comparison done through league tables
**Perspective**	8	State the perspective(s) adopted by the study and why chosen.	Healthcare system approach.<br>Methods – data, intervention costs
**Time horizon**	9	State the time horizon for the study and why appropriate.	Methods - Overview
**Discount rate**	10	Report the discount rate(s) and reason chosen.	Methods - Overview
**Selection of outcomes**	11	Describe what outcomes were used as the measure(s) of benefit(s) and harm(s).	Methods – Overview<br>Supporting information 1: Anaemia prevalence data
**Measurement of outcomes**	12	Describe how outcomes used to capture benefit(s) and harm(s) were measured.	Methods, Data – intervention effectiveness<br>Methods, Data – coverage of anaemia interventions and interaction with treatment effectiveness.<br>Supporting information 1: Anaemia prevalence data<br>Supporting information 2: Intervention effectiveness

Topic	No.	Item	Location where item is reported
**Valuation of outcomes**	13	Describe the population and methods used to measure and value outcomes.	Methods – Data
**Measurement and valuation of resources and costs**	14	Describe how costs were valued.	Methods – Data, Intervention costs<br>Supporting methods 3.3 Cost estimation
**Currency, price date, and conversion**	15	Report the dates of the estimated resource quantities and unit costs, plus the currency and year of conversion.	Methods – Data, Intervention costs<br>Supporting methods 3.3 Cost estimation
**Rationale and description of model**	16	If modelling is used, describe in detail and why used. Report if the model is publicly available and where it can be accessed.	Methods – Overview<br>Methods - Setting National and Global Targets for Future Development Eras<br>Supporting information 3: simulation methods .
**Analytics and assumptions**	17	Describe any methods for analysing or statistically transforming data, any extrapolation methods, and approaches for validating any model used.	Supporting information 3.7: Model assumptions and limitations
**Characterising heterogeneity**	18	Describe any methods used for estimating how the results of the study vary for subgroups.	**NA**
**Characterising distributional effects**	19	Describe how impacts are distributed across different individuals or adjustments made to reflect priority populations.	**NA**

Topic	No.	Item	Location where item is reported
**Characterising uncertainty**	20	Describe methods to characterise any sources of uncertainty in the analysis.	Methods – uncertainty analysis<br>Supporting information 3.6: Propagating parameter uncertainty through to results
**Approach to engagement with patients and others affected by the study**	21	Describe any approaches to engage patients or service recipients, the general public, communities, or stakeholders (such as clinicians or payers) in the design of the study.	**NA**
**Results**			
**Study parameters**	22	Report all analytic inputs (such as values, ranges, references) including uncertainty or distributional assumptions.	Supporting information
**Summary of main results**	23	Report the mean values for the main categories of costs and outcomes of interest and summarise them in the most appropriate overall measure.	Results, Table 2
**Effect of uncertainty**	24	Describe how uncertainty about analytic judgments, inputs, or projections affect findings. Report the effect of choice of discount rate and time horizon, if applicable.	Impact of parameter uncertainty cited along with base case estimates in results section. Results from scenario analyses described in the results section and Supporting information

Topic	No.	Item	Location where item is reported
**Effect of engagement with patients and others affected by the study**	25	Report on any difference patient/service recipient, general public, community, or stakeholder involvement made to the approach or findings of the study	Not conducted for this study, however we discuss the importance of this as part of future directions in the Discussion
**Discussion**			
**Study findings, limitations, generalisability, and current knowledge**	26	Report key findings, limitations, ethical or equity considerations not captured, and how these could affect patients, policy, or practice.	Results<br>Discussion
**Other relevant information**			
**Source of funding**	27	Describe how the study was funded and any role of the funder in the identification, design, conduct, and reporting of the analysis	Role of the funding source section
**Conflicts of interest**	28	Report authors conflicts of interest according to journal or International Committee of Medical Journal Editors requirements.	Declaration of interests section

From: Husereau D, Drummond M, Augustovski F, et al. Consolidated Health Economic Evaluation Reporting Standards 2022 (CHEERS 2022) Explanation and Elaboration: A Report of the ISPOR CHEERS II Good Practices Task Force. Value Health 2022;25. doi:10.1016/j.jval.2021.10.008

Global Anaemia Reduction Target: National Reports

Links to national reports

Country	Country	Country	Country	Country
Afghanistan	Albania	Algeria	Andorra	Angola
Antigua_&_Barbuda	Argentina	Armenia	Australia	Austria
Azerbaijan	Bahamas	Bahrain	Bangladesh	Barbado
Belarus	Belgium	Belize	Benin	Bhutan
Bolivia	Bosnia_&_Herzegovina	Botswana	Brazil	Brunei
Bulgaria	Burkina_Faso	Burundi	Cambodia	Cameroo
Canada	Cape_Verde	Central_African_Republic	Chad	Chile
China	Colombia	Comoros	Congo_-_Brazzaville	Congo_-_Kinsha
Costa_Rica	Croatia	Cuba	Cyprus	Czechia
Côte_d’Ivoire	Denmark	Djibouti	Dominica	Dominic[illegible]blic
Ecuador	Egypt	El_Salvador	Equatorial_Guinea	Estonia
Eswatini	Ethiopia	Fiji	Finland	France
Gabon	Gambia	Georgia	Germany	Ghana
Greece	Grenada	Guatemala	Guinea-Bissau	Guinea
Guyana	Haiti	Honduras	Hungary	Iceland

Country	Country	Country	Country	Country
India	Indonesia	Iran	Iraq	Ireland
Israel	Italy	Jamaica	Japan	Jordan
Kazakhstan	Kenya	Kiribati	Kuwait	Kyrgyzst
Laos	Latvia	Lebanon	Lesotho	Liberia
Libya	Lithuania	Luxembourg	Madagascar	Malawi
Malaysia	Maldives	Mali	Malta	Marshall
Mauritania	Mauritius	Mexico	Micronesia_(Federated_States_of)	Moldova
Monaco	Mongolia	Montenegro	Morocco	Mozamb
Myanmar_(Burma)	Namibia	Nauru	Nepal	Netherla
New_Zealand	Nicaragua	Niger	Nigeria	North_M a
Norway	Oman	Pakistan	Palau	Panama
Papua_New_Guinea	Paraguay	Peru	Philippines	Poland
Portugal	Puerto_Rico	Qatar	Romania	Russia
Rwanda	Samoa	San_Marino	Saudi_Arabia	Senegal
Serbia	Seychelles	Sierra_Leone	Singapore	Slovakia
Slovenia	Solomon_Islands	Somalia	South_Africa	South_K
South_Sudan	Spain	Sri_Lanka	St.*Kitts*&_Nevis	St._Luci
St.*Vincent*&_Grenadines	Sudan	Suriname	Sweden	Switzerl

Country	Country	Country	Country	Country
Syria	São_Tomé_&_Príncipe	Tajikistan	Tanzania	Thailand
Timor-Leste	Togo	Tonga	Trinidad_&_Tobago	Tunisia
Turkey	Turkmenistan	Tuvalu	Uganda	Ukraine
United_Arab_Emirates	United_Kingdom	United_States	Uruguay	Uzbekis[illegible]
Vanuatu	Venezuela	Vietnam	Yemen	Zambia
Zimbabwe				

⬆ Back to Table of Contents

Anaemia Reduction in Afghanistan

National Target

For our baseline CET = 1 x GDP per capita, the national target for anaemia in **Afghanistan** should be a reduction in prevalence of **11%**. This target is estimated with a 95% uncertainty interval from **0%** to **28%**.

For CET based on Pichon-Riviere, the national target for anaemia in **Afghanistan** should be a reduction in prevalence of **0%**. This target is estimated with a 95% uncertainty interval from **0%** to **27%**.

For a near-infinite CET, the national target for anaemia in **Afghanistan** should be a reduction in prevalence of **16%**. This target is estimated with a 95% uncertainty interval from **8%** to **31%**.

Input parameters

- Among women of reproductive age in **Afghanistan**, the prevalence of overall anaemia is **24.0%**

    - **Mild anaemia:** 13.7%
    - **Moderate anaemia:** 9.6%
    - **Severe anaemia:** 0.8%
- The prevalence of malaria is **0.0%**.

Intervention coverage and costs

Iron supplementation (antenatal care)

- **Current coverage:** 43.8%
- **Maximum feasible coverage:** 61.0%
- **Estimated unit cost:** USD $2.24 (0.96 - 4.40)

Iron supplementation (all women of reproductive age)

- **Current coverage:** 18.1%
- **Maximum feasible coverage:** 61.0%
- **Estimated unit cost:** USD $2.14 (1.10 - 5.54)

Antenatal preventative IPTp-SP

- **Current coverage:** 0.0%
- **Maximum feasible coverage:** 61.0%
- **Estimated unit cost:** USD $1.16 (0.63 - 2.58)

Staple food supplementation

- **Current coverage:** 29.4%
- **Maximum feasible coverage:** 78.2%
- **Estimated unit cost:** USD $0.09 (0.01 - 0.28)

Cost-Effectiveness Analysis

The estimated cost per Year of Life Disabled (YLD) averted for each intervention in **Afghanistan** is given below, with interquartile ranges (IQR):

Iron supplementation (antenatal care)

- **Median Cost per YLD:** USD $893
- **IQR:** USD $577 – >USD 100k

Iron supplementation (all women of reproductive age)

- **Median Cost per YLD:** USD $2048
- **IQR:** USD $1427 – $3023

Antenatal preventative IPTp-SP

- **Median Cost per YLD:** >USD 100k
- **IQR:** >USD 100k

Staple food supplementation

- **Median Cost per YLD:** USD $268
- **IQR:** USD $172 – $419

Economic Considerations

Cost-effectiveness thresholds (CET, in USD per DALY averted) in **Afghanistan** is as follows:

- **Estimates from Pichon-Riviere et al. (Lower Bound):** USD $71
- **Estimates from Pichon-Riviere et al. (Upper Bound):** USD $231
- **CET equal to per capita GDP:** USD $356
- **CET equal to 2.3 (LMICs) or 1.7 (HICs) times per capita GDP:** USD $818

⬆ Back to Table of Contents

Anaemia Reduction in Albania

National Target

For our baseline CET = 1 x GDP per capita, the national target for anaemia in **Albania** should be a reduction in prevalence of **13%**. This target is estimated with a 95% uncertainty interval from **3%** to **28%**.

For CET based on Pichon-Riviere, the national target for anaemia in **Albania** should be a reduction in prevalence of **9%**. This target is estimated with a 95% uncertainty interval from **1%**

to **25%**.

For a near-infinite CET, the national target for anaemia in **Albania** should be a reduction in prevalence of **16%**. This target is estimated with a 95% uncertainty interval from **7%** to **29%**.

Input parameters

- Among women of reproductive age in **Albania**, the prevalence of overall anaemia is **28.9%**
  - **Mild anaemia:** 21.3%
  - **Moderate anaemia:** 7.4%
  - **Severe anaemia:** 0.2%
- There is no data on malaria prevalence; assume 0%.

Intervention coverage and costs

Iron supplementation (antenatal care)

- **Current coverage:** 70.0%
- **Maximum feasible coverage:** 89.1%
- **Estimated unit cost:** USD $10.70 (5.27 - 18.49)

Iron supplementation (all women of reproductive age)

- **Current coverage:** 28.9%
- **Maximum feasible coverage:** 89.1%
- **Estimated unit cost:** USD $7.90 (3.98 - 15.94)

Antenatal preventative IPTp-SP

- **Current coverage:** 0.0%
- **Maximum feasible coverage:** 89.1%
- **Estimated unit cost:** USD $8.27 (4.22 - 14.46)

Staple food supplementation

- **Current coverage:** 65.3%
- **Maximum feasible coverage:** 100.0%
- **Estimated unit cost:** USD $0.13 (0.04 - 0.25)

Cost-Effectiveness Analysis

The estimated cost per Year of Life Disabled (YLD) averted for each intervention in **Albania** is given below, with interquartile ranges (IQR):

Iron supplementation (antenatal care)

- **Median Cost per YLD:** USD $5299
- **IQR:** USD $3547 – >USD 100k

Iron supplementation (all women of reproductive age)

- **Median Cost per YLD:** USD $8664
- **IQR:** USD $6318 – $12094

Antenatal preventative IPTp-SP

- **Median Cost per YLD:** >USD 100k
- **IQR:** >USD 100k

Staple food supplementation

- **Median Cost per YLD:** USD $399
- **IQR:** USD $273 – $620

Economic Considerations

Cost-effectiveness thresholds (CET, in USD per DALY averted) in **Albania** is as follows:

- **Estimates from Pichon-Riviere et al. (Lower Bound):** USD $1674
- **Estimates from Pichon-Riviere et al. (Upper Bound):** USD $4853
- **CET equal to per capita GDP:** USD $8368
- **CET equal to 2.3 (LMICs) or 1.7 (HICs) times per capita GDP:** USD $19246

⬆ Back to Table of Contents

Anaemia Reduction in Algeria

National Target

For our baseline CET = 1 x GDP per capita, the national target for anaemia in **Algeria** should be a reduction in prevalence of **24%**. This target is estimated with a 95% uncertainty interval from **11%** to **42%**.

For CET based on Pichon-Riviere, the national target for anaemia in **Algeria** should be a reduction in prevalence of **22%**. This target is estimated with a 95% uncertainty interval from **9%** to **40%**.

For a near-infinite CET, the national target for anaemia in **Algeria** should be a reduction in prevalence of **25%**. This target is estimated with a 95% uncertainty interval from **12%** to **42%**.

Input parameters

- Among women of reproductive age in **Algeria**, the prevalence of overall anaemia is **36.1%**
  - **Mild anaemia:** 23.3%
  - **Moderate anaemia:** 12.1%
  - **Severe anaemia:** 0.6%
- The prevalence of malaria is **0.0%**.

Intervention coverage and costs

Iron supplementation (antenatal care)

- **Current coverage:** 82.3%
- **Maximum feasible coverage:** 87.3%
- **Estimated unit cost:** USD $4.75 (2.17 - 7.89)

Iron supplementation (all women of reproductive age)

- **Current coverage:** 33.9%
- **Maximum feasible coverage:** 82.3%
- **Estimated unit cost:** USD $3.66 (1.80 - 8.12)

Antenatal preventative IPTp-SP

- **Current coverage:** 38.2%

- **Maximum feasible coverage:** 70.0%
- **Estimated unit cost:** USD $2.94 (1.50 - 4.97)

Staple food supplementation

- **Current coverage:** 23.9%
- **Maximum feasible coverage:** 100.0%
- **Estimated unit cost:** USD $0.06 (0.02 - 0.20)

Cost-Effectiveness Analysis

The estimated cost per Year of Life Disabled (YLD) averted for each intervention in **Algeria** is given below, with interquartile ranges (IQR):

Iron supplementation (antenatal care)

- **Median Cost per YLD:** USD $1421
- **IQR:** USD $837 – >USD 100k

Iron supplementation (all women of reproductive age)

- **Median Cost per YLD:** USD $2295
- **IQR:** USD $1581 – $3293

Antenatal preventative IPTp-SP

- **Median Cost per YLD:** >USD 100k
- **IQR:** >USD 100k

Staple food supplementation

- **Median Cost per YLD:** USD $141
- **IQR:** USD $85 – $217

Economic Considerations

Cost-effectiveness thresholds (CET, in USD per DALY averted) in **Algeria** is as follows:

- **Estimates from Pichon-Riviere et al. (Lower Bound):** USD $1420

- **Estimates from Pichon-Riviere et al. (Upper Bound):** USD $2788
- **CET equal to per capita GDP:** USD $5260
- **CET equal to 2.3 (LMICs) or 1.7 (HICs) times per capita GDP:** USD $12098

⬆ Back to Table of Contents

Anaemia Reduction in Andorra

National Target

For our baseline CET = 1 x GDP per capita, the national target for anaemia in **Andorra** should be a reduction in prevalence of **17%**. This target is estimated with a 95% uncertainty interval from **6%** to **35%**.

For CET based on Pichon-Riviere, the national target for anaemia in **Andorra** should be a reduction in prevalence of **16%**. This target is estimated with a 95% uncertainty interval from **6%** to **34%**.

For a near-infinite CET, the national target for anaemia in **Andorra** should be a reduction in prevalence of **25%**. This target is estimated with a 95% uncertainty interval from **14%** to **41%**.

Input parameters

- Among women of reproductive age in **Andorra**, the prevalence of overall anaemia is **8.0%**
  - **Mild anaemia:** 6.5%
  - **Moderate anaemia:** 1.4%
  - **Severe anaemia:** 0.0%
- There is no data on malaria prevalence; assume 0%.

Intervention coverage and costs

Iron supplementation (antenatal care)

- **Current coverage:** 81.0%
- **Maximum feasible coverage:** 100.0%
- **Estimated unit cost:** USD $88.26 (33.25 - 140.59)

Iron supplementation (all women of reproductive age)

- **Current coverage:** 33.4%
- **Maximum feasible coverage:** 100.0%
- **Estimated unit cost:** USD $54.98 (19.60 - 79.52)

Antenatal preventative IPTp-SP

- **Current coverage:** 0.0%
- **Maximum feasible coverage:** 100.0%
- **Estimated unit cost:** USD $39.40 (19.77 - 61.69)

Staple food supplementation

- **Current coverage:** 33.7%
- **Maximum feasible coverage:** 100.0%
- **Estimated unit cost:** USD $0.23 (0.03 - 0.64)

Cost-Effectiveness Analysis

The estimated cost per Year of Life Disabled (YLD) averted for each intervention in **Andorra** is given below, with interquartile ranges (IQR):

Iron supplementation (antenatal care)

- **Median Cost per YLD:** >USD 100k
- **IQR:** >USD 100k

Iron supplementation (all women of reproductive age)

- **Median Cost per YLD:** >USD 100k
- **IQR:** >USD 100k

Antenatal preventative IPTp-SP

- **Median Cost per YLD:** >USD 100k
- **IQR:** >USD 100k

Staple food supplementation

- **Median Cost per YLD:** USD $4423

- **IQR:** USD $2632 – $6896

Economic Considerations

Cost-effectiveness thresholds (CET, in USD per DALY averted) in **Andorra** is as follows:

- **Estimates from Pichon-Riviere et al. (Lower Bound):** USD $12567
- **Estimates from Pichon-Riviere et al. (Upper Bound):** USD $31650
- **CET equal to per capita GDP:** USD $46545
- **CET equal to 2.3 (LMICs) or 1.7 (HICs) times per capita GDP:** USD $65163

⬆ Back to Table of Contents

Anaemia Reduction in Angola

National Target

For our baseline CET = 1 x GDP per capita, the national target for anaemia in **Angola** should be a reduction in prevalence of **25%**. This target is estimated with a 95% uncertainty interval from **8%** to **44%**.

For CET based on Pichon-Riviere, the national target for anaemia in **Angola** should be a reduction in prevalence of **0%**. This target is estimated with a 95% uncertainty interval from **0%** to **41%**.

For a near-infinite CET, the national target for anaemia in **Angola** should be a reduction in prevalence of **27%**. This target is estimated with a 95% uncertainty interval from **14%** to **46%**.

Input parameters

- Among women of reproductive age in **Angola**, the prevalence of overall anaemia is **52.7%**
    - **Mild anaemia:** 34.0%
    - **Moderate anaemia:** 18.0%
    - **Severe anaemia:** 0.7%
- The prevalence of malaria is **23.4%**.

Intervention coverage and costs

Iron supplementation (antenatal care)

- **Current coverage:** 74.4%
- **Maximum feasible coverage:** 81.3%
- **Estimated unit cost:** USD $4.93 (2.30 - 9.14)

Iron supplementation (all women of reproductive age)

- **Current coverage:** 30.7%
- **Maximum feasible coverage:** 81.3%
- **Estimated unit cost:** USD $4.46 (2.68 - 12.68)

Antenatal preventative IPTp-SP

- **Current coverage:** 36.8%
- **Maximum feasible coverage:** 81.3%
- **Estimated unit cost:** USD $3.09 (1.57 - 5.97)

Staple food supplementation

- **Current coverage:** 7.6%
- **Maximum feasible coverage:** 100.0%
- **Estimated unit cost:** USD $0.32 (0.09 - 1.23)

Cost-Effectiveness Analysis

The estimated cost per Year of Life Disabled (YLD) averted for each intervention in **Angola** is given below, with interquartile ranges (IQR):

Iron supplementation (antenatal care)

- **Median Cost per YLD:** USD $1107
- **IQR:** USD $634 – >USD 100k

Iron supplementation (all women of reproductive age)

- **Median Cost per YLD:** USD $2321
- **IQR:** USD $1590 – $3497

Antenatal preventative IPTp-SP

- **Median Cost per YLD:** USD $2819
- **IQR:** USD $2166 – $3795

Staple food supplementation

- **Median Cost per YLD:** USD $632
- **IQR:** USD $400 – $1007

Economic Considerations

Cost-effectiveness thresholds (CET, in USD per DALY averted) in **Angola** is as follows:

- **Estimates from Pichon-Riviere et al. (Lower Bound):** USD $254
- **Estimates from Pichon-Riviere et al. (Upper Bound):** USD $485
- **CET equal to per capita GDP:** USD $2310
- **CET equal to 2.3 (LMICs) or 1.7 (HICs) times per capita GDP:** USD $5312

⬆ Back to Table of Contents

Anaemia Reduction in Antigua & Barbuda

National Target

For our baseline CET = 1 x GDP per capita, the national target for anaemia in **Antigua & Barbuda** should be a reduction in prevalence of **21%**. This target is estimated with a 95% uncertainty interval from **8%** to **39%**.

For CET based on Pichon-Riviere, the national target for anaemia in **Antigua & Barbuda** should be a reduction in prevalence of **16%**. This target is estimated with a 95% uncertainty interval from **6%** to **33%**.

For a near-infinite CET, the national target for anaemia in **Antigua & Barbuda** should be a reduction in prevalence of **24%**. This target is estimated with a 95% uncertainty interval from **13%** to **40%**.

Input parameters

- Among women of reproductive age in **Antigua & Barbuda**, the prevalence of overall anaemia is **33.4%**
  - **Mild anaemia:** 21.2%
  - **Moderate anaemia:** 11.5%
  - **Severe anaemia:** 0.6%
- There is no data on malaria prevalence; assume 0%.

Intervention coverage and costs

Iron supplementation (antenatal care)

- **Current coverage:** NaN%
- **Maximum feasible coverage:** NaN%
- **Estimated unit cost:** USD $NaN (NaN - NaN)

Iron supplementation (all women of reproductive age)

- **Current coverage:** NaN%
- **Maximum feasible coverage:** NaN%
- **Estimated unit cost:** USD $NaN (NaN - NaN)

Antenatal preventative IPTp-SP

- **Current coverage:** NaN%
- **Maximum feasible coverage:** NaN%
- **Estimated unit cost:** USD $NaN (NaN - NaN)

Staple food supplementation

- **Current coverage:** NaN%
- **Maximum feasible coverage:** NaN%
- **Estimated unit cost:** USD $NaN (NaN - NaN)

Cost-Effectiveness Analysis

The estimated cost per Year of Life Disabled (YLD) averted for each intervention in **Antigua & Barbuda** is given below, with interquartile ranges (IQR):

Iron supplementation (antenatal care)

- **Median Cost per YLD:** USD $13163
- **IQR:** USD $8949 – $20728

Iron supplementation (all women of reproductive age)

- **Median Cost per YLD:** USD $18206
- **IQR:** USD $13189 – $26018

Antenatal preventative IPTp-SP

- **Median Cost per YLD:** >USD 100k
- **IQR:** >USD 100k

Staple food supplementation

- **Median Cost per YLD:** USD $462
- **IQR:** USD $295 – $715

Economic Considerations

Cost-effectiveness thresholds (CET, in USD per DALY averted) in **Antigua & Barbuda** is as follows:

- **Estimates from Pichon-Riviere et al. (Lower Bound):** USD $NaN
- **Estimates from Pichon-Riviere et al. (Upper Bound):** USD $NaN
- **CET equal to per capita GDP:** USD $NaN
- **CET equal to 2.3 (LMICs) or 1.7 (HICs) times per capita GDP:** USD $NaN

⬆ Back to Table of Contents

Anaemia Reduction in Argentina

National Target

For our baseline CET = 1 x GDP per capita, the national target for anaemia in **Argentina** should be a reduction in prevalence of **15%**. This target is estimated with a 95% uncertainty interval from **0%** to **32%**.

For CET based on Pichon-Riviere, the national target for anaemia in **Argentina** should be a reduction in prevalence of **15%**. This target is estimated with a 95% uncertainty interval from **0%** to **31%**.

For a near-infinite CET, the national target for anaemia in **Argentina** should be a reduction in prevalence of **19%**. This target is estimated with a 95% uncertainty interval from **9%** to **33%**.

Input parameters

- Among women of reproductive age in **Argentina**, the prevalence of overall anaemia is **17.6%**
  - **Mild anaemia:** 15.7%
  - **Moderate anaemia:** 1.9%
  - **Severe anaemia:** 0.0%
- The prevalence of malaria is **0.0%**.

Intervention coverage and costs

Iron supplementation (antenatal care)

- **Current coverage:** 88.3%
- **Maximum feasible coverage:** 90.0%
- **Estimated unit cost:** USD $17.60 (8.73 - 27.69)

Iron supplementation (all women of reproductive age)

- **Current coverage:** 36.4%
- **Maximum feasible coverage:** 90.0%
- **Estimated unit cost:** USD $9.00 (4.51 - 16.15)

Antenatal preventative IPTp-SP

- **Current coverage:** 0.1%
- **Maximum feasible coverage:** 90.0%
- **Estimated unit cost:** USD $12.84 (6.49 - 20.06)

Staple food supplementation

- **Current coverage:** 53.1%

- **Maximum feasible coverage:** 100.0%
- **Estimated unit cost:** USD $0.57 (0.29 - 1.19)

Cost-Effectiveness Analysis

The estimated cost per Year of Life Disabled (YLD) averted for each intervention in **Argentina** is given below, with interquartile ranges (IQR):

Iron supplementation (antenatal care)

- **Median Cost per YLD:** USD $40023
- **IQR:** USD $9672 – >USD 100k

Iron supplementation (all women of reproductive age)

- **Median Cost per YLD:** USD $15006
- **IQR:** USD $9811 – $23915

Antenatal preventative IPTp-SP

- **Median Cost per YLD:** >USD 100k
- **IQR:** >USD 100k

Staple food supplementation

- **Median Cost per YLD:** USD $2870
- **IQR:** USD $1777 – $5055

Economic Considerations

Cost-effectiveness thresholds (CET, in USD per DALY averted) in **Argentina** is as follows:

- **Estimates from Pichon-Riviere et al. (Lower Bound):** USD $9337
- **Estimates from Pichon-Riviere et al. (Upper Bound):** USD $12769
- **CET equal to per capita GDP:** USD $13731
- **CET equal to 2.3 (LMICs) or 1.7 (HICs) times per capita GDP:** USD $31580

⬆ Back to Table of Contents

Anaemia Reduction in Armenia

National Target

For our baseline CET = 1 x GDP per capita, the national target for anaemia in **Armenia** should be a reduction in prevalence of **18%**. This target is estimated with a 95% uncertainty interval from **4%** to **36%**.

For CET based on Pichon-Riviere, the national target for anaemia in **Armenia** should be a reduction in prevalence of **19%**. This target is estimated with a 95% uncertainty interval from **4%** to **36%**.

For a near-infinite CET, the national target for anaemia in **Armenia** should be a reduction in prevalence of **22%**. This target is estimated with a 95% uncertainty interval from **12%** to **35%**.

Input parameters

- Among women of reproductive age in **Armenia**, the prevalence of overall anaemia is **22.4%**
  - **Mild anaemia:** 14.3%
  - **Moderate anaemia:** 7.7%
  - **Severe anaemia:** 0.5%
- The prevalence of malaria is **0.0%**.

Intervention coverage and costs

Iron supplementation (antenatal care)

- **Current coverage:** 43.8%
- **Maximum feasible coverage:** 99.8%
- **Estimated unit cost:** USD $10.48 (5.15 - 18.02)

Iron supplementation (all women of reproductive age)

- **Current coverage:** 18.1%
- **Maximum feasible coverage:** 99.8%
- **Estimated unit cost:** USD $7.16 (3.61 - 14.67)

Antenatal preventative IPTp-SP

- **Current coverage:** 0.0%
- **Maximum feasible coverage:** 99.8%
- **Estimated unit cost:** USD $8.08 (4.12 - 14.12)

Staple food supplementation

- **Current coverage:** 51.1%
- **Maximum feasible coverage:** 83.5%
- **Estimated unit cost:** USD $0.33 (0.23 - 0.50)

Cost-Effectiveness Analysis

The estimated cost per Year of Life Disabled (YLD) averted for each intervention in **Armenia** is given below, with interquartile ranges (IQR):

Iron supplementation (antenatal care)

- **Median Cost per YLD:** USD $3990
- **IQR:** USD $3012 – $5535

Iron supplementation (all women of reproductive age)

- **Median Cost per YLD:** USD $7211
- **IQR:** USD $5107 – $10441

Antenatal preventative IPTp-SP

- **Median Cost per YLD:** >USD 100k
- **IQR:** >USD 100k

Staple food supplementation

- **Median Cost per YLD:** USD $884
- **IQR:** USD $626 – $1358

Economic Considerations

Cost-effectiveness thresholds (CET, in USD per DALY averted) in **Armenia** is as follows:

- **Estimates from Pichon-Riviere et al. (Lower Bound):** USD $6885
- **Estimates from Pichon-Riviere et al. (Upper Bound):** USD $9413
- **CET equal to per capita GDP:** USD $8716
- **CET equal to 2.3 (LMICs) or 1.7 (HICs) times per capita GDP:** USD $20046

⬆ Back to Table of Contents

Anaemia Reduction in Australia

National Target

For our baseline CET = 1 x GDP per capita, the national target for anaemia in **Australia** should be a reduction in prevalence of **20%**. This target is estimated with a 95% uncertainty interval from **8%** to **39%**.

For CET based on Pichon-Riviere, the national target for anaemia in **Australia** should be a reduction in prevalence of **20%**. This target is estimated with a 95% uncertainty interval from **8%** to **37%**.

For a near-infinite CET, the national target for anaemia in **Australia** should be a reduction in prevalence of **28%**. This target is estimated with a 95% uncertainty interval from **15%** to **44%**.

Input parameters

- Among women of reproductive age in **Australia**, the prevalence of overall anaemia is **8.9%**
  - **Mild anaemia:** 6.8%
  - **Moderate anaemia:** 2.0%
  - **Severe anaemia:** 0.1%
- There is no data on malaria prevalence; assume 0%.

Intervention coverage and costs

Iron supplementation (antenatal care)

- **Current coverage:** 81.0%
- **Maximum feasible coverage:** 100.0%

- **Estimated unit cost:** USD $109.33 (43.79 - 171.12)

Iron supplementation (all women of reproductive age)

- **Current coverage:** 33.4%
- **Maximum feasible coverage:** 100.0%
- **Estimated unit cost:** USD $65.71 (24.96 - 94.75)

Antenatal preventative IPTp-SP

- **Current coverage:** 0.0%
- **Maximum feasible coverage:** 100.0%
- **Estimated unit cost:** USD $54.59 (27.36 - 83.50)

Staple food supplementation

- **Current coverage:** 19.5%
- **Maximum feasible coverage:** 100.0%
- **Estimated unit cost:** USD $0.27 (0.12 - 0.52)

Cost-Effectiveness Analysis

The estimated cost per Year of Life Disabled (YLD) averted for each intervention in **Australia** is given below, with interquartile ranges (IQR):

Iron supplementation (antenatal care)

- **Median Cost per YLD:** >USD 100k
- **IQR:** USD $98457 – >USD 100k

Iron supplementation (all women of reproductive age)

- **Median Cost per YLD:** >USD 100k
- **IQR:** >USD 100k

Antenatal preventative IPTp-SP

- **Median Cost per YLD:** >USD 100k
- **IQR:** >USD 100k

Staple food supplementation

- **Median Cost per YLD:** USD $3305
- **IQR:** USD $2233 – $5052

Economic Considerations

Cost-effectiveness thresholds (CET, in USD per DALY averted) in **Australia** is as follows:

- **Estimates from Pichon-Riviere et al. (Lower Bound):** USD $49181
- **Estimates from Pichon-Riviere et al. (Upper Bound):** USD $58241
- **CET equal to per capita GDP:** USD $64712
- **CET equal to 2.3 (LMICs) or 1.7 (HICs) times per capita GDP:** USD $90596

⬆ Back to Table of Contents

Anaemia Reduction in Austria

National Target

For our baseline CET = 1 x GDP per capita, the national target for anaemia in **Austria** should be a reduction in prevalence of **17%**. This target is estimated with a 95% uncertainty interval from **6%** to **34%**.

For CET based on Pichon-Riviere, the national target for anaemia in **Austria** should be a reduction in prevalence of **17%**. This target is estimated with a 95% uncertainty interval from **6%** to **34%**.

For a near-infinite CET, the national target for anaemia in **Austria** should be a reduction in prevalence of **25%**. This target is estimated with a 95% uncertainty interval from **14%** to **41%**.

Input parameters

- Among women of reproductive age in **Austria**, the prevalence of overall anaemia is **7.9%**
  - **Mild anaemia:** 6.5%
  - **Moderate anaemia:** 1.4%
  - **Severe anaemia:** 0.0%
- There is no data on malaria prevalence; assume 0%.

Intervention coverage and costs

Iron supplementation (antenatal care)

- **Current coverage:** 81.0%
- **Maximum feasible coverage:** 100.0%
- **Estimated unit cost:** USD $57.37 (17.80 - 94.49)

Iron supplementation (all women of reproductive age)

- **Current coverage:** 33.4%
- **Maximum feasible coverage:** 100.0%
- **Estimated unit cost:** USD $35.94 (10.08 - 51.18)

Antenatal preventative IPTp-SP

- **Current coverage:** 0.0%
- **Maximum feasible coverage:** 100.0%
- **Estimated unit cost:** USD $16.27 (8.20 - 27.23)

Staple food supplementation

- **Current coverage:** 34.0%
- **Maximum feasible coverage:** 100.0%
- **Estimated unit cost:** USD $0.40 (0.27 - 0.62)

Cost-Effectiveness Analysis

The estimated cost per Year of Life Disabled (YLD) averted for each intervention in **Austria** is given below, with interquartile ranges (IQR):

Iron supplementation (antenatal care)

- **Median Cost per YLD:** >USD 100k
- **IQR:** USD $71109 – >USD 100k

Iron supplementation (all women of reproductive age)

- **Median Cost per YLD:** >USD 100k
- **IQR:** >USD 100k

Antenatal preventative IPTp-SP

- **Median Cost per YLD:** >USD 100k
- **IQR:** >USD 100k

Staple food supplementation

- **Median Cost per YLD:** USD $7189
- **IQR:** USD $4828 – $10859

Economic Considerations

Cost-effectiveness thresholds (CET, in USD per DALY averted) in **Austria** is as follows:

- **Estimates from Pichon-Riviere et al. (Lower Bound):** USD $44640
- **Estimates from Pichon-Riviere et al. (Upper Bound):** USD $52551
- **CET equal to per capita GDP:** USD $56506
- **CET equal to 2.3 (LMICs) or 1.7 (HICs) times per capita GDP:** USD $79108

⬆ Back to Table of Contents

Anaemia Reduction in Azerbaijan

National Target

For our baseline CET = 1 x GDP per capita, the national target for anaemia in **Azerbaijan** should be a reduction in prevalence of **19%**. This target is estimated with a 95% uncertainty interval from **4%** to **34%**.

For CET based on Pichon-Riviere, the national target for anaemia in **Azerbaijan** should be a reduction in prevalence of **9%**. This target is estimated with a 95% uncertainty interval from **2%** to **27%**.

For a near-infinite CET, the national target for anaemia in **Azerbaijan** should be a reduction in prevalence of **21%**. This target is estimated with a 95% uncertainty interval from **11%** to **34%**.

Input parameters

- Among women of reproductive age in **Azerbaijan**, the prevalence of overall anaemia is **35.6%**
  - **Mild anaemia:** 20.1%
  - **Moderate anaemia:** 14.4%
  - **Severe anaemia:** 1.1%
- The prevalence of malaria is **0.0%**.

Intervention coverage and costs

Iron supplementation (antenatal care)

- **Current coverage:** 27.2%
- **Maximum feasible coverage:** 79.7%
- **Estimated unit cost:** USD $16.54 (8.21 - 26.84)

Iron supplementation (all women of reproductive age)

- **Current coverage:** 11.2%
- **Maximum feasible coverage:** 79.7%
- **Estimated unit cost:** USD $9.27 (4.67 - 17.59)

Antenatal preventative IPTp-SP

- **Current coverage:** 0.0%
- **Maximum feasible coverage:** 79.7%
- **Estimated unit cost:** USD $12.41 (6.28 - 20.16)

Staple food supplementation

- **Current coverage:** 65.6%
- **Maximum feasible coverage:** 100.0%
- **Estimated unit cost:** USD $0.21 (0.13 - 0.32)

Cost-Effectiveness Analysis

The estimated cost per Year of Life Disabled (YLD) averted for each intervention in **Azerbaijan** is given below, with interquartile ranges (IQR):

Iron supplementation (antenatal care)

- **Median Cost per YLD:** USD $3570
- **IQR:** USD $2752 – $4849

Iron supplementation (all women of reproductive age)

- **Median Cost per YLD:** USD $4983
- **IQR:** USD $3589 – $6943

Antenatal preventative IPTp-SP

- **Median Cost per YLD:** >USD 100k
- **IQR:** >USD 100k

Staple food supplementation

- **Median Cost per YLD:** USD $264
- **IQR:** USD $183 – $377

Economic Considerations

Cost-effectiveness thresholds (CET, in USD per DALY averted) in **Azerbaijan** is as follows:

- **Estimates from Pichon-Riviere et al. (Lower Bound):** USD $2003
- **Estimates from Pichon-Riviere et al. (Upper Bound):** USD $2719
- **CET equal to per capita GDP:** USD $7155
- **CET equal to 2.3 (LMICs) or 1.7 (HICs) times per capita GDP:** USD $16457

⬆ Back to Table of Contents

Anaemia Reduction in Bahamas

National Target

For our baseline CET = 1 x GDP per capita, the national target for anaemia in **Bahamas** should be a reduction in prevalence of **27%**. This target is estimated with a 95% uncertainty interval from **12%** to **45%**.

For CET based on Pichon-Riviere, the national target for anaemia in **Bahamas** should be a reduction in prevalence of **22%**. This target is estimated with a 95% uncertainty interval from **9%** to **41%**.

For a near-infinite CET, the national target for anaemia in **Bahamas** should be a reduction in prevalence of **29%**. This target is estimated with a 95% uncertainty interval from **16%** to **45%**.

Input parameters

- Among women of reproductive age in **Bahamas**, the prevalence of overall anaemia is **35.6%**
  - **Mild anaemia:** 22.4%
  - **Moderate anaemia:** 12.5%
  - **Severe anaemia:** 0.7%
- There is no data on malaria prevalence; assume 0%.

Intervention coverage and costs

Iron supplementation (antenatal care)

- **Current coverage:** NaN%
- **Maximum feasible coverage:** NaN%
- **Estimated unit cost:** USD $85.42 (31.72 - 133.95)

Iron supplementation (all women of reproductive age)

- **Current coverage:** NaN%
- **Maximum feasible coverage:** NaN%
- **Estimated unit cost:** USD $53.08 (18.45 - 78.08)

Antenatal preventative IPTp-SP

- **Current coverage:** NaN%
- **Maximum feasible coverage:** NaN%
- **Estimated unit cost:** USD $34.51 (17.32 - 52.66)

Staple food supplementation

- **Current coverage:** NaN%
- **Maximum feasible coverage:** NaN%
- **Estimated unit cost:** USD $0.90 (0.48 - 1.87)

Cost-Effectiveness Analysis

The estimated cost per Year of Life Disabled (YLD) averted for each intervention in **Bahamas** is given below, with interquartile ranges (IQR):

Iron supplementation (antenatal care)

- **Median Cost per YLD:** USD $16860
- **IQR:** USD $11709 – $27553

Iron supplementation (all women of reproductive age)

- **Median Cost per YLD:** USD $25392
- **IQR:** USD $17535 – $36527

Antenatal preventative IPTp-SP

- **Median Cost per YLD:** >USD 100k
- **IQR:** >USD 100k

Staple food supplementation

- **Median Cost per YLD:** USD $1568
- **IQR:** USD $1081 – $2363

Economic Considerations

Cost-effectiveness thresholds (CET, in USD per DALY averted) in **Bahamas** is as follows:

- **Estimates from Pichon-Riviere et al. (Lower Bound):** USD $13900
- **Estimates from Pichon-Riviere et al. (Upper Bound):** USD $16332
- **CET equal to per capita GDP:** USD $34750

- **CET equal to 2.3 (LMICs) or 1.7 (HICs) times per capita GDP:** USD $48649

⬆ Back to Table of Contents

Anaemia Reduction in Bahrain

National Target

For our baseline CET = 1 x GDP per capita, the national target for anaemia in **Bahrain** should be a reduction in prevalence of **22%**. This target is estimated with a 95% uncertainty interval from **9%** to **40%**.

For CET based on Pichon-Riviere, the national target for anaemia in **Bahrain** should be a reduction in prevalence of **16%**. This target is estimated with a 95% uncertainty interval from **0%** to **34%**.

For a near-infinite CET, the national target for anaemia in **Bahrain** should be a reduction in prevalence of **25%**. This target is estimated with a 95% uncertainty interval from **14%** to **39%**.

Input parameters

- Among women of reproductive age in **Bahrain**, the prevalence of overall anaemia is **42.7%**
  - **Mild anaemia:** 30.4%
  - **Moderate anaemia:** 11.9%
  - **Severe anaemia:** 0.4%
- There is no data on malaria prevalence; assume 0%.

Intervention coverage and costs

Iron supplementation (antenatal care)

- **Current coverage:** 81.0%
- **Maximum feasible coverage:** 100.0%
- **Estimated unit cost:** USD $62.55 (20.36 - 99.53)

Iron supplementation (all women of reproductive age)

- **Current coverage:** 33.4%
- **Maximum feasible coverage:** 100.0%

- **Estimated unit cost:** USD $36.06 (12.48 - 67.88)

Antenatal preventative IPTp-SP

- **Current coverage:** 0.0%
- **Maximum feasible coverage:** 100.0%
- **Estimated unit cost:** USD $17.85 (8.99 - 28.39)

Staple food supplementation

- **Current coverage:** 37.6%
- **Maximum feasible coverage:** 100.0%
- **Estimated unit cost:** USD $2.69 (2.18 - 3.90)

Cost-Effectiveness Analysis

The estimated cost per Year of Life Disabled (YLD) averted for each intervention in **Bahrain** is given below, with interquartile ranges (IQR):

Iron supplementation (antenatal care)

- **Median Cost per YLD:** USD $12757
- **IQR:** USD $8414 – $19960

Iron supplementation (all women of reproductive age)

- **Median Cost per YLD:** USD $19686
- **IQR:** USD $13857 – $29357

Antenatal preventative IPTp-SP

- **Median Cost per YLD:** >USD 100k
- **IQR:** >USD 100k

Staple food supplementation

- **Median Cost per YLD:** USD $5169
- **IQR:** USD $3736 – $7712

Economic Considerations

Cost-effectiveness thresholds (CET, in USD per DALY averted) in **Bahrain** is as follows:

- **Estimates from Pichon-Riviere et al. (Lower Bound):** USD $8434
- **Estimates from Pichon-Riviere et al. (Upper Bound):** USD $9889
- **CET equal to per capita GDP:** USD $29084
- **CET equal to 2.3 (LMICs) or 1.7 (HICs) times per capita GDP:** USD $40718

⬆ Back to Table of Contents

Anaemia Reduction in Bangladesh

National Target

For our baseline CET = 1 x GDP per capita, the national target for anaemia in **Bangladesh** should be a reduction in prevalence of **12%**. This target is estimated with a 95% uncertainty interval from **1%** to **26%**.

For CET based on Pichon-Riviere, the national target for anaemia in **Bangladesh** should be a reduction in prevalence of **0%**. This target is estimated with a 95% uncertainty interval from **0%** to **12%**.

For a near-infinite CET, the national target for anaemia in **Bangladesh** should be a reduction in prevalence of **16%**. This target is estimated with a 95% uncertainty interval from **9%** to **26%**.

Input parameters

- Among women of reproductive age in **Bangladesh**, the prevalence of overall anaemia is **44.4%**
  - **Mild anaemia:** 29.3%
  - **Moderate anaemia:** 14.5%
  - **Severe anaemia:** 0.6%
- The prevalence of malaria is **0.0%**.

Intervention coverage and costs

Iron supplementation (antenatal care)

- **Current coverage:** 76.4%
- **Maximum feasible coverage:** 87.6%
- **Estimated unit cost:** USD $2.55 (1.18 - 4.96)

Iron supplementation (all women of reproductive age)

- **Current coverage:** 31.5%
- **Maximum feasible coverage:** 87.6%
- **Estimated unit cost:** USD $3.13 (2.07 - 10.72)

Antenatal preventative IPTp-SP

- **Current coverage:** 0.0%
- **Maximum feasible coverage:** 87.6%
- **Estimated unit cost:** USD $1.34 (0.73 - 2.67)

Staple food supplementation

- **Current coverage:** 0.3%
- **Maximum feasible coverage:** 32.0%
- **Estimated unit cost:** USD $0.70 (0.10 - 2.15)

Cost-Effectiveness Analysis

The estimated cost per Year of Life Disabled (YLD) averted for each intervention in **Bangladesh** is given below, with interquartile ranges (IQR):

Iron supplementation (antenatal care)

- **Median Cost per YLD:** USD $566
- **IQR:** USD $382 – $1280

Iron supplementation (all women of reproductive age)

- **Median Cost per YLD:** USD $2269
- **IQR:** USD $1579 – $3364

Antenatal preventative IPTp-SP

- **Median Cost per YLD:** >USD 100k
- **IQR:** >USD 100k

Staple food supplementation

- **Median Cost per YLD:** USD $1267
- **IQR:** USD $826 – $1973

Economic Considerations

Cost-effectiveness thresholds (CET, in USD per DALY averted) in **Bangladesh** is as follows:

- **Estimates from Pichon-Riviere et al. (Lower Bound):** USD $202
- **Estimates from Pichon-Riviere et al. (Upper Bound):** USD $430
- **CET equal to per capita GDP:** USD $2529
- **CET equal to 2.3 (LMICs) or 1.7 (HICs) times per capita GDP:** USD $5817

⬆ Back to Table of Contents

Anaemia Reduction in Barbados

National Target

For our baseline CET = 1 x GDP per capita, the national target for anaemia in **Barbados** should be a reduction in prevalence of **23%**. This target is estimated with a 95% uncertainty interval from **9%** to **40%**.

For CET based on Pichon-Riviere, the national target for anaemia in **Barbados** should be a reduction in prevalence of **18%**. This target is estimated with a 95% uncertainty interval from **7%** to **37%**.

For a near-infinite CET, the national target for anaemia in **Barbados** should be a reduction in prevalence of **26%**. This target is estimated with a 95% uncertainty interval from **14%** to **41%**.

Input parameters

- Among women of reproductive age in **Barbados**, the prevalence of overall anaemia is **30.3%**
  - **Mild anaemia:** 17.5%
  - **Moderate anaemia:** 11.9%

  - **Severe anaemia:** 1.0%
- There is no data on malaria prevalence; assume 0%.

Intervention coverage and costs

Iron supplementation (antenatal care)

- **Current coverage:** 81.0%
- **Maximum feasible coverage:** 100.0%
- **Estimated unit cost:** USD $65.95 (21.93 - 105.45)

Iron supplementation (all women of reproductive age)

- **Current coverage:** 33.4%
- **Maximum feasible coverage:** 100.0%
- **Estimated unit cost:** USD $39.76 (11.83 - 57.58)

Antenatal preventative IPTp-SP

- **Current coverage:** 0.0%
- **Maximum feasible coverage:** 100.0%
- **Estimated unit cost:** USD $19.85 (9.99 - 30.74)

Staple food supplementation

- **Current coverage:** 31.3%
- **Maximum feasible coverage:** 100.0%
- **Estimated unit cost:** USD $0.79 (0.49 - 1.43)

Cost-Effectiveness Analysis

The estimated cost per Year of Life Disabled (YLD) averted for each intervention in **Barbados** is given below, with interquartile ranges (IQR):

Iron supplementation (antenatal care)

- **Median Cost per YLD:** USD $13086
- **IQR:** USD $8833 – $21132

Iron supplementation (all women of reproductive age)

- **Median Cost per YLD:** USD $19180
- **IQR:** USD $13212 – $26423

Antenatal preventative IPTp-SP

- **Median Cost per YLD:** >USD 100k
- **IQR:** >USD 100k

Staple food supplementation

- **Median Cost per YLD:** USD $1530
- **IQR:** USD $1058 – $2258

Economic Considerations

Cost-effectiveness thresholds (CET, in USD per DALY averted) in **Barbados** is as follows:

- **Estimates from Pichon-Riviere et al. (Lower Bound):** USD $10429
- **Estimates from Pichon-Riviere et al. (Upper Bound):** USD $12243
- **CET equal to per capita GDP:** USD $22673
- **CET equal to 2.3 (LMICs) or 1.7 (HICs) times per capita GDP:** USD $31742

⬆ Back to Table of Contents

Anaemia Reduction in Belarus

National Target

For our baseline CET = 1 x GDP per capita, the national target for anaemia in **Belarus** should be a reduction in prevalence of **9%**. This target is estimated with a 95% uncertainty interval from **2%** to **23%**.

For CET based on Pichon-Riviere, the national target for anaemia in **Belarus** should be a reduction in prevalence of **9%**. This target is estimated with a 95% uncertainty interval from **2%** to **23%**.

For a near-infinite CET, the national target for anaemia in **Belarus** should be a reduction in prevalence of **22%**. This target is estimated with a 95% uncertainty interval from **12%** to **35%**.

Input parameters

- Among women of reproductive age in **Belarus**, the prevalence of overall anaemia is **20.2%**
  - **Mild anaemia:** 12.2%
  - **Moderate anaemia:** 7.5%
  - **Severe anaemia:** 0.6%
- There is no data on malaria prevalence; assume 0%.

Intervention coverage and costs

Iron supplementation (antenatal care)

- **Current coverage:** 54.0%
- **Maximum feasible coverage:** 100.0%
- **Estimated unit cost:** USD $83.37 (41.58 - 130.09)

Iron supplementation (all women of reproductive age)

- **Current coverage:** 22.2%
- **Maximum feasible coverage:** 100.0%
- **Estimated unit cost:** USD $35.36 (17.71 - 59.71)

Antenatal preventative IPTp-SP

- **Current coverage:** 0.0%
- **Maximum feasible coverage:** 100.0%
- **Estimated unit cost:** USD $62.53 (31.34 - 98.55)

Staple food supplementation

- **Current coverage:** 63.4%
- **Maximum feasible coverage:** 100.0%
- **Estimated unit cost:** USD $0.16 (0.06 - 0.32)

Cost-Effectiveness Analysis

The estimated cost per Year of Life Disabled (YLD) averted for each intervention in **Belarus** is given below, with interquartile ranges (IQR):

Iron supplementation (antenatal care)

- **Median Cost per YLD:** USD $29349
- **IQR:** USD $22220 – $39586

Iron supplementation (all women of reproductive age)

- **Median Cost per YLD:** USD $32632
- **IQR:** USD $23255 – $47132

Antenatal preventative IPTp-SP

- **Median Cost per YLD:** >USD 100k
- **IQR:** >USD 100k

Staple food supplementation

- **Median Cost per YLD:** USD $463
- **IQR:** USD $317 – $692

Economic Considerations

Cost-effectiveness thresholds (CET, in USD per DALY averted) in **Belarus** is as follows:

- **Estimates from Pichon-Riviere et al. (Lower Bound):** USD $3132
- **Estimates from Pichon-Riviere et al. (Upper Bound):** USD $4306
- **CET equal to per capita GDP:** USD $7829
- **CET equal to 2.3 (LMICs) or 1.7 (HICs) times per capita GDP:** USD $18007

⬆ Back to Table of Contents

Anaemia Reduction in Belgium

National Target

For our baseline CET = 1 x GDP per capita, the national target for anaemia in **Belgium** should be a reduction in prevalence of **16%**. This target is estimated with a 95% uncertainty interval from **6%** to **34%**.

For CET based on Pichon-Riviere, the national target for anaemia in **Belgium** should be a reduction in prevalence of **16%**. This target is estimated with a 95% uncertainty interval from **6%** to **33%**.

For a near-infinite CET, the national target for anaemia in **Belgium** should be a reduction in prevalence of **25%**. This target is estimated with a 95% uncertainty interval from **13%** to **40%**.

Input parameters

- Among women of reproductive age in **Belgium**, the prevalence of overall anaemia is **7.2%**
  - **Mild anaemia:** 5.4%
  - **Moderate anaemia:** 1.7%
  - **Severe anaemia:** 0.1%
- There is no data on malaria prevalence; assume 0%.

Intervention coverage and costs

Iron supplementation (antenatal care)

- **Current coverage:** 81.0%
- **Maximum feasible coverage:** 100.0%
- **Estimated unit cost:** USD $57.38 (17.80 - 94.57)

Iron supplementation (all women of reproductive age)

- **Current coverage:** 33.4%
- **Maximum feasible coverage:** 100.0%
- **Estimated unit cost:** USD $35.89 (10.06 - 51.02)

Antenatal preventative IPTp-SP

- **Current coverage:** 0.0%
- **Maximum feasible coverage:** 100.0%
- **Estimated unit cost:** USD $16.27 (8.20 - 27.23)

Staple food supplementation

- **Current coverage:** 38.4%
- **Maximum feasible coverage:** 100.0%
- **Estimated unit cost:** USD $0.09 (0.03 - 0.14)

Cost-Effectiveness Analysis

The estimated cost per Year of Life Disabled (YLD) averted for each intervention in **Belgium** is given below, with interquartile ranges (IQR):

Iron supplementation (antenatal care)

- **Median Cost per YLD:** USD $86575
- **IQR:** USD $58863 – >USD 100k

Iron supplementation (all women of reproductive age)

- **Median Cost per YLD:** >USD 100k
- **IQR:** USD $84340 – >USD 100k

Antenatal preventative IPTp-SP

- **Median Cost per YLD:** >USD 100k
- **IQR:** >USD 100k

Staple food supplementation

- **Median Cost per YLD:** USD $1132
- **IQR:** USD $762 – $1671

Economic Considerations

Cost-effectiveness thresholds (CET, in USD per DALY averted) in **Belgium** is as follows:

- **Estimates from Pichon-Riviere et al. (Lower Bound):** USD $43315
- **Estimates from Pichon-Riviere et al. (Upper Bound):** USD $50802
- **CET equal to per capita GDP:** USD $53475

- **CET equal to 2.3 (LMICs) or 1.7 (HICs) times per capita GDP:** USD $74865

⬆ Back to Table of Contents

Anaemia Reduction in Belize

National Target

For our baseline CET = 1 x GDP per capita, the national target for anaemia in **Belize** should be a reduction in prevalence of **19%**. This target is estimated with a 95% uncertainty interval from **8%** to **33%**.

For CET based on Pichon-Riviere, the national target for anaemia in **Belize** should be a reduction in prevalence of **17%**. This target is estimated with a 95% uncertainty interval from **6%** to **31%**.

For a near-infinite CET, the national target for anaemia in **Belize** should be a reduction in prevalence of **19%**. This target is estimated with a 95% uncertainty interval from **10%** to **31%**.

Input parameters

- Among women of reproductive age in **Belize**, the prevalence of overall anaemia is **40.5%**
  - **Mild anaemia:** 23.3%
  - **Moderate anaemia:** 16.1%
  - **Severe anaemia:** 1.2%
- The prevalence of malaria is **0.0%**.

Intervention coverage and costs

Iron supplementation (antenatal care)

- **Current coverage:** 88.3%
- **Maximum feasible coverage:** 93.0%
- **Estimated unit cost:** USD $9.84 (4.83 - 16.00)

Iron supplementation (all women of reproductive age)

- **Current coverage:** 36.4%
- **Maximum feasible coverage:** 93.0%

- **Estimated unit cost:** USD $5.80 (3.42 - 14.96)

Antenatal preventative IPTp-SP

- **Current coverage:** 0.1%
- **Maximum feasible coverage:** 93.0%
- **Estimated unit cost:** USD $6.92 (3.52 - 11.15)

Staple food supplementation

- **Current coverage:** 25.7%
- **Maximum feasible coverage:** 69.6%
- **Estimated unit cost:** USD $1.04 (0.69 - 1.83)

Cost-Effectiveness Analysis

The estimated cost per Year of Life Disabled (YLD) averted for each intervention in **Belize** is given below, with interquartile ranges (IQR):

Iron supplementation (antenatal care)

- **Median Cost per YLD:** USD $2276
- **IQR:** USD $1296 – >USD 100k

Iron supplementation (all women of reproductive age)

- **Median Cost per YLD:** USD $3100
- **IQR:** USD $2126 – $4369

Antenatal preventative IPTp-SP

- **Median Cost per YLD:** >USD 100k
- **IQR:** >USD 100k

Staple food supplementation

- **Median Cost per YLD:** USD $1265
- **IQR:** USD $910 – $1864

Economic Considerations

Cost-effectiveness thresholds (CET, in USD per DALY averted) in **Belize** is as follows:

- **Estimates from Pichon-Riviere et al. (Lower Bound):** USD $3275
- **Estimates from Pichon-Riviere et al. (Upper Bound):** USD $4553
- **CET equal to per capita GDP:** USD $7988
- **CET equal to 2.3 (LMICs) or 1.7 (HICs) times per capita GDP:** USD $18371

⬆ Back to Table of Contents

Anaemia Reduction in Benin

National Target

For our baseline CET = 1 x GDP per capita, the national target for anaemia in **Benin** should be a reduction in prevalence of **25%**. This target is estimated with a 95% uncertainty interval from **1%** to **44%**.

For CET based on Pichon-Riviere, the national target for anaemia in **Benin** should be a reduction in prevalence of **0%**. This target is estimated with a 95% uncertainty interval from **0%** to **29%**.

For a near-infinite CET, the national target for anaemia in **Benin** should be a reduction in prevalence of **27%**. This target is estimated with a 95% uncertainty interval from **14%** to **45%**.

Input parameters

- Among women of reproductive age in **Benin**, the prevalence of overall anaemia is **66.7%**
  - **Mild anaemia:** 36.4%
  - **Moderate anaemia:** 29.0%
  - **Severe anaemia:** 1.3%
- The prevalence of malaria is **35.8%**.

Intervention coverage and costs

Iron supplementation (antenatal care)

- **Current coverage:** 84.5%

- **Maximum feasible coverage:** 89.5%
- **Estimated unit cost:** USD $2.77 (1.26 - 5.33)

Iron supplementation (all women of reproductive age)

- **Current coverage:** 34.8%
- **Maximum feasible coverage:** 84.5%
- **Estimated unit cost:** USD $2.86 (1.83 - 9.85)

Antenatal preventative IPTp-SP

- **Current coverage:** 33.4%
- **Maximum feasible coverage:** 83.0%
- **Estimated unit cost:** USD $1.44 (0.75 - 2.75)

Staple food supplementation

- **Current coverage:** 3.8%
- **Maximum feasible coverage:** 89.2%
- **Estimated unit cost:** USD $0.54 (0.10 - 1.74)

Cost-Effectiveness Analysis

The estimated cost per Year of Life Disabled (YLD) averted for each intervention in **Benin** is given below, with interquartile ranges (IQR):

Iron supplementation (antenatal care)

- **Median Cost per YLD:** USD $418
- **IQR:** USD $241 – >USD 100k

Iron supplementation (all women of reproductive age)

- **Median Cost per YLD:** USD $1134
- **IQR:** USD $749 – $1631

Antenatal preventative IPTp-SP

- **Median Cost per YLD:** USD $640

- **IQR:** USD $479 – $856

Staple food supplementation

- **Median Cost per YLD:** USD $623
- **IQR:** USD $398 – $986

Economic Considerations

Cost-effectiveness thresholds (CET, in USD per DALY averted) in **Benin** is as follows:

- **Estimates from Pichon-Riviere et al. (Lower Bound):** USD $115
- **Estimates from Pichon-Riviere et al. (Upper Bound):** USD $230
- **CET equal to per capita GDP:** USD $1435
- **CET equal to 2.3 (LMICs) or 1.7 (HICs) times per capita GDP:** USD $3300

⬆ Back to Table of Contents

Anaemia Reduction in Bhutan

National Target

For our baseline CET = 1 x GDP per capita, the national target for anaemia in **Bhutan** should be a reduction in prevalence of **27%**. This target is estimated with a 95% uncertainty interval from **11%** to **46%**.

For CET based on Pichon-Riviere, the national target for anaemia in **Bhutan** should be a reduction in prevalence of **18%**. This target is estimated with a 95% uncertainty interval from **0%** to **41%**.

For a near-infinite CET, the national target for anaemia in **Bhutan** should be a reduction in prevalence of **27%**. This target is estimated with a 95% uncertainty interval from **14%** to **45%**.

Input parameters

- Among women of reproductive age in **Bhutan**, the prevalence of overall anaemia is **56.7%**
  - **Mild anaemia:** 34.8%
  - **Moderate anaemia:** 21.0%
  - **Severe anaemia:** 0.9%

- The prevalence of malaria is **0.0%**.

Intervention coverage and costs

Iron supplementation (antenatal care)

- **Current coverage:** 86.9%
- **Maximum feasible coverage:** 88.9%
- **Estimated unit cost:** USD $4.20 (2.04 - 7.56)

Iron supplementation (all women of reproductive age)

- **Current coverage:** 35.8%
- **Maximum feasible coverage:** 88.9%
- **Estimated unit cost:** USD $3.25 (2.10 - 10.91)

Antenatal preventative IPTp-SP

- **Current coverage:** 0.0%
- **Maximum feasible coverage:** 88.9%
- **Estimated unit cost:** USD $2.55 (1.33 - 4.46)

Staple food supplementation

- **Current coverage:** 1.1%
- **Maximum feasible coverage:** 88.0%
- **Estimated unit cost:** USD $0.67 (0.10 - 2.06)

Cost-Effectiveness Analysis

The estimated cost per Year of Life Disabled (YLD) averted for each intervention in **Bhutan** is given below, with interquartile ranges (IQR):

Iron supplementation (antenatal care)

- **Median Cost per YLD:** USD $783
- **IQR:** USD $433 – >USD 100k

Iron supplementation (all women of reproductive age)

- **Median Cost per YLD:** USD $1545
- **IQR:** USD $1003 – $2278

Antenatal preventative IPTp-SP

- **Median Cost per YLD:** >USD 100k
- **IQR:** >USD 100k

Staple food supplementation

- **Median Cost per YLD:** USD $785
- **IQR:** USD $498 – $1258

Economic Considerations

Cost-effectiveness thresholds (CET, in USD per DALY averted) in **Bhutan** is as follows:

- **Estimates from Pichon-Riviere et al. (Lower Bound):** USD $498
- **Estimates from Pichon-Riviere et al. (Upper Bound):** USD $961
- **CET equal to per capita GDP:** USD $3560
- **CET equal to 2.3 (LMICs) or 1.7 (HICs) times per capita GDP:** USD $8188

⬆ Back to Table of Contents

Anaemia Reduction in Bolivia

National Target

For our baseline CET = 1 x GDP per capita, the national target for anaemia in **Bolivia** should be a reduction in prevalence of **25%**. This target is estimated with a 95% uncertainty interval from **2%** to **44%**.

For CET based on Pichon-Riviere, the national target for anaemia in **Bolivia** should be a reduction in prevalence of **21%**. This target is estimated with a 95% uncertainty interval from **0%** to **41%**.

For a near-infinite CET, the national target for anaemia in **Bolivia** should be a reduction in prevalence of **27%**. This target is estimated with a 95% uncertainty interval from **15%** to **44%**.

Input parameters

- Among women of reproductive age in **Bolivia**, the prevalence of overall anaemia is **21.8%**
  - **Mild anaemia:** 11.3%
  - **Moderate anaemia:** 9.4%
  - **Severe anaemia:** 1.0%
- The prevalence of malaria is **0.0%**.

Intervention coverage and costs

Iron supplementation (antenatal care)

- **Current coverage:** 79.3%
- **Maximum feasible coverage:** 90.9%
- **Estimated unit cost:** USD $5.32 (2.53 - 8.97)

Iron supplementation (all women of reproductive age)

- **Current coverage:** 32.7%
- **Maximum feasible coverage:** 90.9%
- **Estimated unit cost:** USD $3.75 (1.88 - 8.28)

Antenatal preventative IPTp-SP

- **Current coverage:** 0.0%
- **Maximum feasible coverage:** 90.9%
- **Estimated unit cost:** USD $3.51 (1.82 - 6.02)

Staple food supplementation

- **Current coverage:** 15.4%
- **Maximum feasible coverage:** 100.0%
- **Estimated unit cost:** USD $0.51 (0.06 - 1.07)

Cost-Effectiveness Analysis

The estimated cost per Year of Life Disabled (YLD) averted for each intervention in **Bolivia** is given below, with interquartile ranges (IQR):

Iron supplementation (antenatal care)

- **Median Cost per YLD:** USD $1814
- **IQR:** USD $1181 – >USD 100k

Iron supplementation (all women of reproductive age)

- **Median Cost per YLD:** USD $2989
- **IQR:** USD $2157 – $4218

Antenatal preventative IPTp-SP

- **Median Cost per YLD:** >USD 100k
- **IQR:** >USD 100k

Staple food supplementation

- **Median Cost per YLD:** USD $1113
- **IQR:** USD $746 – $1660

Economic Considerations

Cost-effectiveness thresholds (CET, in USD per DALY averted) in **Bolivia** is as follows:

- **Estimates from Pichon-Riviere et al. (Lower Bound):** USD $999
- **Estimates from Pichon-Riviere et al. (Upper Bound):** USD $1962
- **CET equal to per capita GDP:** USD $3701
- **CET equal to 2.3 (LMICs) or 1.7 (HICs) times per capita GDP:** USD $8512

⬆ Back to Table of Contents

Anaemia Reduction in Bosnia & Herzegovina

National Target

For our baseline CET = 1 x GDP per capita, the national target for anaemia in **Bosnia & Herzegovina** should be a reduction in prevalence of **18%**. This target is estimated with a 95% uncertainty interval from **4%** to **35%**.

For CET based on Pichon-Riviere, the national target for anaemia in **Bosnia & Herzegovina** should be a reduction in prevalence of **16%**. This target is estimated with a 95% uncertainty interval from **4%** to **35%**.

For a near-infinite CET, the national target for anaemia in **Bosnia & Herzegovina** should be a reduction in prevalence of **22%**. This target is estimated with a 95% uncertainty interval from **12%** to **36%**.

Input parameters

- Among women of reproductive age in **Bosnia & Herzegovina**, the prevalence of overall anaemia is **23.5%**
  - **Mild anaemia:** 15.4%
  - **Moderate anaemia:** 7.7%
  - **Severe anaemia:** 0.4%
- There is no data on malaria prevalence; assume 0%.

Intervention coverage and costs

Iron supplementation (antenatal care)

- **Current coverage:** NaN%
- **Maximum feasible coverage:** NaN%
- **Estimated unit cost:** USD $NaN (NaN - NaN)

Iron supplementation (all women of reproductive age)

- **Current coverage:** NaN%
- **Maximum feasible coverage:** NaN%
- **Estimated unit cost:** USD $NaN (NaN - NaN)

Antenatal preventative IPTp-SP

- **Current coverage:** NaN%

- **Maximum feasible coverage:** NaN%
- **Estimated unit cost:** USD $NaN (NaN - NaN)

Staple food supplementation

- **Current coverage:** NaN%
- **Maximum feasible coverage:** NaN%
- **Estimated unit cost:** USD $NaN (NaN - NaN)

Cost-Effectiveness Analysis

The estimated cost per Year of Life Disabled (YLD) averted for each intervention in **Bosnia & Herzegovina** is given below, with interquartile ranges (IQR):

Iron supplementation (antenatal care)

- **Median Cost per YLD:** USD $3941
- **IQR:** USD $2935 – $5338

Iron supplementation (all women of reproductive age)

- **Median Cost per YLD:** USD $7667
- **IQR:** USD $5442 – $11403

Antenatal preventative IPTp-SP

- **Median Cost per YLD:** >USD 100k
- **IQR:** >USD 100k

Staple food supplementation

- **Median Cost per YLD:** USD $1618
- **IQR:** USD $1142 – $2357

Economic Considerations

Cost-effectiveness thresholds (CET, in USD per DALY averted) in **Bosnia & Herzegovina** is as follows:

- **Estimates from Pichon-Riviere et al. (Lower Bound):** USD $NaN
- **Estimates from Pichon-Riviere et al. (Upper Bound):** USD $NaN
- **CET equal to per capita GDP:** USD $NaN
- **CET equal to 2.3 (LMICs) or 1.7 (HICs) times per capita GDP:** USD $NaN

⬆ Back to Table of Contents

Anaemia Reduction in Botswana

National Target

For our baseline CET = 1 x GDP per capita, the national target for anaemia in **Botswana** should be a reduction in prevalence of **19%**. This target is estimated with a 95% uncertainty interval from **5%** to **34%**.

For CET based on Pichon-Riviere, the national target for anaemia in **Botswana** should be a reduction in prevalence of **17%**. This target is estimated with a 95% uncertainty interval from **0%** to **33%**.

For a near-infinite CET, the national target for anaemia in **Botswana** should be a reduction in prevalence of **20%**. This target is estimated with a 95% uncertainty interval from **10%** to **36%**.

Input parameters

- Among women of reproductive age in **Botswana**, the prevalence of overall anaemia is **34.6%**
  - **Mild anaemia:** 22.3%
  - **Moderate anaemia:** 11.7%
  - **Severe anaemia:** 0.6%
- The prevalence of malaria is **0.0%**.

Intervention coverage and costs

Iron supplementation (antenatal care)

- **Current coverage:** 90.2%
- **Maximum feasible coverage:** 91.9%
- **Estimated unit cost:** USD $9.53 (4.56 - 15.11)

Iron supplementation (all women of reproductive age)

- **Current coverage:** 37.2%
- **Maximum feasible coverage:** 91.9%
- **Estimated unit cost:** USD $6.31 (3.13 - 11.97)

Antenatal preventative IPTp-SP

- **Current coverage:** 27.8%
- **Maximum feasible coverage:** 91.9%
- **Estimated unit cost:** USD $6.54 (3.30 - 10.43)

Staple food supplementation

- **Current coverage:** 48.9%
- **Maximum feasible coverage:** 98.8%
- **Estimated unit cost:** USD $1.73 (1.34 - 2.61)

Cost-Effectiveness Analysis

The estimated cost per Year of Life Disabled (YLD) averted for each intervention in **Botswana** is given below, with interquartile ranges (IQR):

Iron supplementation (antenatal care)

- **Median Cost per YLD:** USD $4753
- **IQR:** USD $1752 – >USD 100k

Iron supplementation (all women of reproductive age)

- **Median Cost per YLD:** USD $3723
- **IQR:** USD $2634 – $5459

Antenatal preventative IPTp-SP

- **Median Cost per YLD:** >USD 100k
- **IQR:** >USD 100k

Staple food supplementation

- **Median Cost per YLD:** USD $2578
- **IQR:** USD $1789 – $3800

Economic Considerations

Cost-effectiveness thresholds (CET, in USD per DALY averted) in **Botswana** is as follows:

- **Estimates from Pichon-Riviere et al. (Lower Bound):** USD $3552
- **Estimates from Pichon-Riviere et al. (Upper Bound):** USD $4857
- **CET equal to per capita GDP:** USD $7250
- **CET equal to 2.3 (LMICs) or 1.7 (HICs) times per capita GDP:** USD $16675

⬆ Back to Table of Contents

Anaemia Reduction in Brazil

National Target

For our baseline CET = 1 x GDP per capita, the national target for anaemia in **Brazil** should be a reduction in prevalence of **24%**. This target is estimated with a 95% uncertainty interval from **12%** to **41%**.

For CET based on Pichon-Riviere, the national target for anaemia in **Brazil** should be a reduction in prevalence of **23%**. This target is estimated with a 95% uncertainty interval from **12%** to **39%**.

For a near-infinite CET, the national target for anaemia in **Brazil** should be a reduction in prevalence of **23%**. This target is estimated with a 95% uncertainty interval from **12%** to **39%**.

Input parameters

- Among women of reproductive age in **Brazil**, the prevalence of overall anaemia is **34.2%**
  - **Mild anaemia:** 20.6%
  - **Moderate anaemia:** 12.5%
  - **Severe anaemia:** 1.1%
- The prevalence of malaria is **0.0%**.

Intervention coverage and costs

Iron supplementation (antenatal care)

- **Current coverage:** 88.3%
- **Maximum feasible coverage:** 93.3%
- **Estimated unit cost:** USD $3.84 (1.82 - 7.00)

Iron supplementation (all women of reproductive age)

- **Current coverage:** 36.4%
- **Maximum feasible coverage:** 88.3%
- **Estimated unit cost:** USD $3.13 (1.58 - 7.60)

Antenatal preventative IPTp-SP

- **Current coverage:** 0.1%
- **Maximum feasible coverage:** 87.9%
- **Estimated unit cost:** USD $2.41 (1.27 - 4.46)

Staple food supplementation

- **Current coverage:** 34.4%
- **Maximum feasible coverage:** 100.0%
- **Estimated unit cost:** USD $0.91 (0.59 - 1.69)

Cost-Effectiveness Analysis

The estimated cost per Year of Life Disabled (YLD) averted for each intervention in **Brazil** is given below, with interquartile ranges (IQR):

Iron supplementation (antenatal care)

- **Median Cost per YLD:** USD $1365
- **IQR:** USD $701 – >USD 100k

Iron supplementation (all women of reproductive age)

- **Median Cost per YLD:** USD $1978
- **IQR:** USD $1364 – $2885

Antenatal preventative IPTp-SP

- **Median Cost per YLD:** >USD 100k
- **IQR:** >USD 100k

Staple food supplementation

- **Median Cost per YLD:** USD $1495
- **IQR:** USD $1065 – $2148

Economic Considerations

Cost-effectiveness thresholds (CET, in USD per DALY averted) in **Brazil** is as follows:

- **Estimates from Pichon-Riviere et al. (Lower Bound):** USD $7031
- **Estimates from Pichon-Riviere et al. (Upper Bound):** USD $9541
- **CET equal to per capita GDP:** USD $10044
- **CET equal to 2.3 (LMICs) or 1.7 (HICs) times per capita GDP:** USD $23100

⬆ Back to Table of Contents

Anaemia Reduction in Brunei

National Target

For our baseline CET = 1 x GDP per capita, the national target for anaemia in **Brunei** should be a reduction in prevalence of **22%**. This target is estimated with a 95% uncertainty interval from **9%** to **41%**.

For CET based on Pichon-Riviere, the national target for anaemia in **Brunei** should be a reduction in prevalence of **12%**. This target is estimated with a 95% uncertainty interval from **0%** to **39%**.

For a near-infinite CET, the national target for anaemia in **Brunei** should be a reduction in prevalence of **30%**. This target is estimated with a 95% uncertainty interval from **17%** to **47%**.

Input parameters

- Among women of reproductive age in **Brunei**, the prevalence of overall anaemia is **14.1%**
  - **Mild anaemia:** 11.1%
  - **Moderate anaemia:** 2.9%
  - **Severe anaemia:** 0.1%
- There is no data on malaria prevalence; assume 0%.

Intervention coverage and costs

Iron supplementation (antenatal care)

- **Current coverage:** NaN%
- **Maximum feasible coverage:** NaN%
- **Estimated unit cost:** USD $NaN (NaN - NaN)

Iron supplementation (all women of reproductive age)

- **Current coverage:** NaN%
- **Maximum feasible coverage:** NaN%
- **Estimated unit cost:** USD $NaN (NaN - NaN)

Antenatal preventative IPTp-SP

- **Current coverage:** NaN%
- **Maximum feasible coverage:** NaN%
- **Estimated unit cost:** USD $NaN (NaN - NaN)

Staple food supplementation

- **Current coverage:** NaN%
- **Maximum feasible coverage:** NaN%
- **Estimated unit cost:** USD $NaN (NaN - NaN)

Cost-Effectiveness Analysis

The estimated cost per Year of Life Disabled (YLD) averted for each intervention in **Brunei** is given below, with interquartile ranges (IQR):

Iron supplementation (antenatal care)

- **Median Cost per YLD:** USD $59118
- **IQR:** USD $38579 – >USD 100k

Iron supplementation (all women of reproductive age)

- **Median Cost per YLD:** USD $81753
- **IQR:** USD $53448 – >USD 100k

Antenatal preventative IPTp-SP

- **Median Cost per YLD:** >USD 100k
- **IQR:** >USD 100k

Staple food supplementation

- **Median Cost per YLD:** USD $5931
- **IQR:** USD $3843 – $9583

Economic Considerations

Cost-effectiveness thresholds (CET, in USD per DALY averted) in **Brunei** is as follows:

- **Estimates from Pichon-Riviere et al. (Lower Bound):** USD $NaN
- **Estimates from Pichon-Riviere et al. (Upper Bound):** USD $NaN
- **CET equal to per capita GDP:** USD $NaN
- **CET equal to 2.3 (LMICs) or 1.7 (HICs) times per capita GDP:** USD $NaN

⬆ Back to Table of Contents

Anaemia Reduction in Bulgaria

National Target

For our baseline CET = 1 x GDP per capita, the national target for anaemia in **Bulgaria** should be a reduction in prevalence of **21%**. This target is estimated with a 95% uncertainty interval from **7%** to **36%**.

For CET based on Pichon-Riviere, the national target for anaemia in **Bulgaria** should be a reduction in prevalence of **18%**. This target is estimated with a 95% uncertainty interval from **5%** to **34%**.

For a near-infinite CET, the national target for anaemia in **Bulgaria** should be a reduction in prevalence of **22%**. This target is estimated with a 95% uncertainty interval from **11%** to **35%**.

Input parameters

- Among women of reproductive age in **Bulgaria**, the prevalence of overall anaemia is **25.5%**
  - **Mild anaemia:** 16.5%
  - **Moderate anaemia:** 8.5%
  - **Severe anaemia:** 0.5%
- There is no data on malaria prevalence; assume 0%.

Intervention coverage and costs

Iron supplementation (antenatal care)

- **Current coverage:** 54.0%
- **Maximum feasible coverage:** 92.9%
- **Estimated unit cost:** USD $16.16 (7.99 - 27.03)

Iron supplementation (all women of reproductive age)

- **Current coverage:** 22.2%
- **Maximum feasible coverage:** 92.9%
- **Estimated unit cost:** USD $10.67 (5.37 - 20.44)

Antenatal preventative IPTp-SP

- **Current coverage:** 0.0%
- **Maximum feasible coverage:** 92.9%
- **Estimated unit cost:** USD $12.52 (6.34 - 21.23)

Staple food supplementation

- **Current coverage:** 58.5%
- **Maximum feasible coverage:** 100.0%
- **Estimated unit cost:** USD $0.50 (0.36 - 0.75)

Cost-Effectiveness Analysis

The estimated cost per Year of Life Disabled (YLD) averted for each intervention in **Bulgaria** is given below, with interquartile ranges (IQR):

Iron supplementation (antenatal care)

- **Median Cost per YLD:** USD $5156
- **IQR:** USD $3697 – $7134

Iron supplementation (all women of reproductive age)

- **Median Cost per YLD:** USD $9125
- **IQR:** USD $6183 – $13221

Antenatal preventative IPTp-SP

- **Median Cost per YLD:** >USD 100k
- **IQR:** >USD 100k

Staple food supplementation

- **Median Cost per YLD:** USD $1339
- **IQR:** USD $917 – $1970

Economic Considerations

Cost-effectiveness thresholds (CET, in USD per DALY averted) in **Bulgaria** is as follows:

- **Estimates from Pichon-Riviere et al. (Lower Bound):** USD $7741
- **Estimates from Pichon-Riviere et al. (Upper Bound):** USD $10742
- **CET equal to per capita GDP:** USD $15798

- **CET equal to 2.3 (LMICs) or 1.7 (HICs) times per capita GDP:** USD $36334

⬆ Back to Table of Contents

Anaemia Reduction in Burkina Faso

National Target

For our baseline CET = 1 x GDP per capita, the national target for anaemia in **Burkina Faso** should be a reduction in prevalence of **8%**. This target is estimated with a 95% uncertainty interval from **0%** to **21%**.

For CET based on Pichon-Riviere, the national target for anaemia in **Burkina Faso** should be a reduction in prevalence of **0%**. This target is estimated with a 95% uncertainty interval from **0%** to **8%**.

For a near-infinite CET, the national target for anaemia in **Burkina Faso** should be a reduction in prevalence of **15%**. This target is estimated with a 95% uncertainty interval from **8%** to **24%**.

Input parameters

- Among women of reproductive age in **Burkina Faso**, the prevalence of overall anaemia is **47.9%**
  - **Mild anaemia:** 23.9%
  - **Moderate anaemia:** 22.1%
  - **Severe anaemia:** 1.9%
- The prevalence of malaria is **35.5%**.

Intervention coverage and costs

Iron supplementation (antenatal care)

- **Current coverage:** 95.7%
- **Maximum feasible coverage:** 98.4%
- **Estimated unit cost:** USD $2.75 (1.20 - 5.08)

Iron supplementation (all women of reproductive age)

- **Current coverage:** 39.5%

- **Maximum feasible coverage:** 98.4%
- **Estimated unit cost:** USD $2.70 (1.81 - 9.47)

Antenatal preventative IPTp-SP

- **Current coverage:** 78.2%
- **Maximum feasible coverage:** 98.4%
- **Estimated unit cost:** USD $1.44 (0.75 - 2.75)

Staple food supplementation

- **Current coverage:** 0.9%
- **Maximum feasible coverage:** 28.8%
- **Estimated unit cost:** USD $0.43 (0.11 - 1.49)

Cost-Effectiveness Analysis

The estimated cost per Year of Life Disabled (YLD) averted for each intervention in **Burkina Faso** is given below, with interquartile ranges (IQR):

Iron supplementation (antenatal care)

- **Median Cost per YLD:** >USD 100k
- **IQR:** USD $305 – >USD 100k

Iron supplementation (all women of reproductive age)

- **Median Cost per YLD:** USD $1358
- **IQR:** USD $909 – $1998

Antenatal preventative IPTp-SP

- **Median Cost per YLD:** USD $710
- **IQR:** USD $520 – $1000

Staple food supplementation

- **Median Cost per YLD:** USD $676
- **IQR:** USD $425 – $1025

Economic Considerations

Cost-effectiveness thresholds (CET, in USD per DALY averted) in **Burkina Faso** is as follows:

- **Estimates from Pichon-Riviere et al. (Lower Bound):** USD $61
- **Estimates from Pichon-Riviere et al. (Upper Bound):** USD $201
- **CET equal to per capita GDP:** USD $874
- **CET equal to 2.3 (LMICs) or 1.7 (HICs) times per capita GDP:** USD $2010

⬆ Back to Table of Contents

Anaemia Reduction in Burundi

National Target

For our baseline CET = 1 x GDP per capita, the national target for anaemia in **Burundi** should be a reduction in prevalence of **0%**. This target is estimated with a 95% uncertainty interval from **0%** to **0%**.

For CET based on Pichon-Riviere, the national target for anaemia in **Burundi** should be a reduction in prevalence of **0%**. This target is estimated with a 95% uncertainty interval from **0%** to **0%**.

For a near-infinite CET, the national target for anaemia in **Burundi** should be a reduction in prevalence of **18%**. This target is estimated with a 95% uncertainty interval from **10%** to **29%**.

Input parameters

- Among women of reproductive age in **Burundi**, the prevalence of overall anaemia is **38.3%**
  - **Mild anaemia:** 24.1%
  - **Moderate anaemia:** 13.7%
  - **Severe anaemia:** 0.6%
- The prevalence of malaria is **29.4%**.

Intervention coverage and costs

Iron supplementation (antenatal care)

- **Current coverage:** 43.8%
- **Maximum feasible coverage:** 99.2%
- **Estimated unit cost:** USD $2.31 (0.96 - 4.36)

Iron supplementation (all women of reproductive age)

- **Current coverage:** 18.1%
- **Maximum feasible coverage:** 99.2%
- **Estimated unit cost:** USD $2.25 (1.14 - 5.44)

Antenatal preventative IPTp-SP

- **Current coverage:** 20.4%
- **Maximum feasible coverage:** 99.2%
- **Estimated unit cost:** USD $1.12 (0.59 - 2.34)

Staple food supplementation

- **Current coverage:** 2.0%
- **Maximum feasible coverage:** 54.6%
- **Estimated unit cost:** USD $0.38 (0.10 - 1.33)

Cost-Effectiveness Analysis

The estimated cost per Year of Life Disabled (YLD) averted for each intervention in **Burundi** is given below, with interquartile ranges (IQR):

Iron supplementation (antenatal care)

- **Median Cost per YLD:** USD $708
- **IQR:** USD $473 – >USD 100k

Iron supplementation (all women of reproductive age)

- **Median Cost per YLD:** USD $1508
- **IQR:** USD $1056 – $2182

Antenatal preventative IPTp-SP

- **Median Cost per YLD:** USD $1358
- **IQR:** USD $1020 – $1822

Staple food supplementation

- **Median Cost per YLD:** USD $1063
- **IQR:** USD $640 – $1662

Economic Considerations

Cost-effectiveness thresholds (CET, in USD per DALY averted) in **Burundi** is as follows:

- **Estimates from Pichon-Riviere et al. (Lower Bound):** USD $24
- **Estimates from Pichon-Riviere et al. (Upper Bound):** USD $78
- **CET equal to per capita GDP:** USD $200
- **CET equal to 2.3 (LMICs) or 1.7 (HICs) times per capita GDP:** USD $459

⬆ Back to Table of Contents

Anaemia Reduction in Cambodia

National Target

For our baseline CET = 1 x GDP per capita, the national target for anaemia in **Cambodia** should be a reduction in prevalence of **5%**. This target is estimated with a 95% uncertainty interval from **0%** to **16%**.

For CET based on Pichon-Riviere, the national target for anaemia in **Cambodia** should be a reduction in prevalence of **1%**. This target is estimated with a 95% uncertainty interval from **0%** to **15%**.

For a near-infinite CET, the national target for anaemia in **Cambodia** should be a reduction in prevalence of **8%**. This target is estimated with a 95% uncertainty interval from **2%** to **16%**.

Input parameters

- Among women of reproductive age in **Cambodia**, the prevalence of overall anaemia is **41.8%**
  - **Mild anaemia:** 23.8%

- **Moderate anaemia:** 17.0%
- **Severe anaemia:** 1.0%

- The prevalence of malaria is **0.0%**.

Intervention coverage and costs

Iron supplementation (antenatal care)

- **Current coverage:** 97.8%
- **Maximum feasible coverage:** 98.7%
- **Estimated unit cost:** USD $3.11 (1.49 - 5.95)

Iron supplementation (all women of reproductive age)

- **Current coverage:** 40.3%
- **Maximum feasible coverage:** 98.7%
- **Estimated unit cost:** USD $2.72 (1.90 - 10.10)

Antenatal preventative IPTp-SP

- **Current coverage:** 35.0%
- **Maximum feasible coverage:** 98.7%
- **Estimated unit cost:** USD $1.81 (0.98 - 3.48)

Staple food supplementation

- **Current coverage:** 0.4%
- **Maximum feasible coverage:** 2.7%
- **Estimated unit cost:** USD $0.74 (0.11 - 2.27)

Cost-Effectiveness Analysis

The estimated cost per Year of Life Disabled (YLD) averted for each intervention in **Cambodia** is given below, with interquartile ranges (IQR):

Iron supplementation (antenatal care)

- **Median Cost per YLD:** >USD 100k

- **IQR:** USD $530 – >USD 100k

Iron supplementation (all women of reproductive age)

- **Median Cost per YLD:** USD $1805
- **IQR:** USD $1221 – $2803

Antenatal preventative IPTp-SP

- **Median Cost per YLD:** >USD 100k
- **IQR:** >USD 100k

Staple food supplementation

- **Median Cost per YLD:** USD $1244
- **IQR:** USD $794 – $1986

Economic Considerations

Cost-effectiveness thresholds (CET, in USD per DALY averted) in **Cambodia** is as follows:

- **Estimates from Pichon-Riviere et al. (Lower Bound):** USD $506
- **Estimates from Pichon-Riviere et al. (Upper Bound):** USD $975
- **CET equal to per capita GDP:** USD $1875
- **CET equal to 2.3 (LMICs) or 1.7 (HICs) times per capita GDP:** USD $4313

⬆ Back to Table of Contents

Anaemia Reduction in Cameroon

National Target

For our baseline CET = 1 x GDP per capita, the national target for anaemia in **Cameroon** should be a reduction in prevalence of **16%**. This target is estimated with a 95% uncertainty interval from **1%** to **30%**.

For CET based on Pichon-Riviere, the national target for anaemia in **Cameroon** should be a reduction in prevalence of **0%**. This target is estimated with a 95% uncertainty interval from **0%** to **18%**.

For a near-infinite CET, the national target for anaemia in **Cameroon** should be a reduction in prevalence of **19%**. This target is estimated with a 95% uncertainty interval from **11%** to **30%**.

Input parameters

- Among women of reproductive age in **Cameroon**, the prevalence of overall anaemia is **37.6%**
  - **Mild anaemia:** 19.6%
  - **Moderate anaemia:** 16.6%
  - **Severe anaemia:** 1.4%
- The prevalence of malaria is **22.3%**.

Intervention coverage and costs

Iron supplementation (antenatal care)

- **Current coverage:** 78.6%
- **Maximum feasible coverage:** 88.7%
- **Estimated unit cost:** USD $3.21 (1.41 - 5.65)

Iron supplementation (all women of reproductive age)

- **Current coverage:** 32.4%
- **Maximum feasible coverage:** 88.7%
- **Estimated unit cost:** USD $2.85 (1.41 - 6.72)

Antenatal preventative IPTp-SP

- **Current coverage:** 65.8%
- **Maximum feasible coverage:** 88.7%
- **Estimated unit cost:** USD $1.81 (0.93 - 3.31)

Staple food supplementation

- **Current coverage:** 8.0%
- **Maximum feasible coverage:** 50.3%
- **Estimated unit cost:** USD $0.41 (0.09 - 1.36)

Cost-Effectiveness Analysis

The estimated cost per Year of Life Disabled (YLD) averted for each intervention in **Cameroon** is given below, with interquartile ranges (IQR):

Iron supplementation (antenatal care)

- **Median Cost per YLD:** USD $624
- **IQR:** USD $421 – >USD 100k

Iron supplementation (all women of reproductive age)

- **Median Cost per YLD:** USD $1407
- **IQR:** USD $991 – $2048

Antenatal preventative IPTp-SP

- **Median Cost per YLD:** USD $1755
- **IQR:** USD $1273 – $2399

Staple food supplementation

- **Median Cost per YLD:** USD $759
- **IQR:** USD $488 – $1171

Economic Considerations

Cost-effectiveness thresholds (CET, in USD per DALY averted) in **Cameroon** is as follows:

- **Estimates from Pichon-Riviere et al. (Lower Bound):** USD $184
- **Estimates from Pichon-Riviere et al. (Upper Bound):** USD $368
- **CET equal to per capita GDP:** USD $1674
- **CET equal to 2.3 (LMICs) or 1.7 (HICs) times per capita GDP:** USD $3849

⬆ Back to Table of Contents

Anaemia Reduction in Canada

National Target

For our baseline CET = 1 x GDP per capita, the national target for anaemia in **Canada** should be a reduction in prevalence of **14%**. This target is estimated with a 95% uncertainty interval from **5%** to **32%**.

For CET based on Pichon-Riviere, the national target for anaemia in **Canada** should be a reduction in prevalence of **14%**. This target is estimated with a 95% uncertainty interval from **4%** to **30%**.

For a near-infinite CET, the national target for anaemia in **Canada** should be a reduction in prevalence of **22%**. This target is estimated with a 95% uncertainty interval from **12%** to **37%**.

Input parameters

- Among women of reproductive age in **Canada**, the prevalence of overall anaemia is **12.3%**
  - **Mild anaemia:** 11.0%
  - **Moderate anaemia:** 1.3%
  - **Severe anaemia:** 0.0%
- There is no data on malaria prevalence; assume 0%.

Intervention coverage and costs

Iron supplementation (antenatal care)

- **Current coverage:** 81.0%
- **Maximum feasible coverage:** 100.0%
- **Estimated unit cost:** USD $99.85 (39.08 - 155.87)

Iron supplementation (all women of reproductive age)

- **Current coverage:** 33.4%
- **Maximum feasible coverage:** 100.0%
- **Estimated unit cost:** USD $57.29 (20.73 - 81.82)

Antenatal preventative IPTp-SP

- **Current coverage:** 0.0%

- **Maximum feasible coverage:** 100.0%
- **Estimated unit cost:** USD $47.13 (23.63 - 71.64)

Staple food supplementation

- **Current coverage:** 48.2%
- **Maximum feasible coverage:** 100.0%
- **Estimated unit cost:** USD $0.63 (0.42 - 1.08)

Cost-Effectiveness Analysis

The estimated cost per Year of Life Disabled (YLD) averted for each intervention in **Canada** is given below, with interquartile ranges (IQR):

Iron supplementation (antenatal care)

- **Median Cost per YLD:** >USD 100k
- **IQR:** USD $60189 – >USD 100k

Iron supplementation (all women of reproductive age)

- **Median Cost per YLD:** >USD 100k
- **IQR:** USD $85804 – >USD 100k

Antenatal preventative IPTp-SP

- **Median Cost per YLD:** >USD 100k
- **IQR:** >USD 100k

Staple food supplementation

- **Median Cost per YLD:** USD $5825
- **IQR:** USD $3643 – $9932

Economic Considerations

Cost-effectiveness thresholds (CET, in USD per DALY averted) in **Canada** is as follows:

- **Estimates from Pichon-Riviere et al. (Lower Bound):** USD $43765

- **Estimates from Pichon-Riviere et al. (Upper Bound):** USD $51237
- **CET equal to per capita GDP:** USD $53372
- **CET equal to 2.3 (LMICs) or 1.7 (HICs) times per capita GDP:** USD $74720

⬆ Back to Table of Contents

Anaemia Reduction in Cape Verde

National Target

For our baseline CET = 1 x GDP per capita, the national target for anaemia in **Cape Verde** should be a reduction in prevalence of **27%**. This target is estimated with a 95% uncertainty interval from **10%** to **45%**.

For CET based on Pichon-Riviere, the national target for anaemia in **Cape Verde** should be a reduction in prevalence of **22%**. This target is estimated with a 95% uncertainty interval from **0%** to **42%**.

For a near-infinite CET, the national target for anaemia in **Cape Verde** should be a reduction in prevalence of **27%**. This target is estimated with a 95% uncertainty interval from **14%** to **45%**.

Input parameters

- Among women of reproductive age in **Cape Verde**, the prevalence of overall anaemia is **42.2%**
  - **Mild anaemia:** 26.8%
  - **Moderate anaemia:** 14.7%
  - **Severe anaemia:** 0.7%
- The prevalence of malaria is **0.0%**.

Intervention coverage and costs

Iron supplementation (antenatal care)

- **Current coverage:** NaN%
- **Maximum feasible coverage:** NaN%
- **Estimated unit cost:** USD $NaN (NaN - NaN)

Iron supplementation (all women of reproductive age)

- **Current coverage:** NaN%
- **Maximum feasible coverage:** NaN%
- **Estimated unit cost:** USD $NaN (NaN - NaN)

Antenatal preventative IPTp-SP

- **Current coverage:** NaN%
- **Maximum feasible coverage:** NaN%
- **Estimated unit cost:** USD $NaN (NaN - NaN)

Staple food supplementation

- **Current coverage:** NaN%
- **Maximum feasible coverage:** NaN%
- **Estimated unit cost:** USD $NaN (NaN - NaN)

Cost-Effectiveness Analysis

The estimated cost per Year of Life Disabled (YLD) averted for each intervention in **Cape Verde** is given below, with interquartile ranges (IQR):

Iron supplementation (antenatal care)

- **Median Cost per YLD:** USD $1478
- **IQR:** USD $808 – >USD 100k

Iron supplementation (all women of reproductive age)

- **Median Cost per YLD:** USD $2499
- **IQR:** USD $1724 – $3808

Antenatal preventative IPTp-SP

- **Median Cost per YLD:** >USD 100k
- **IQR:** >USD 100k

Staple food supplementation

- **Median Cost per YLD:** USD $870
- **IQR:** USD $523 – $1337

Economic Considerations

Cost-effectiveness thresholds (CET, in USD per DALY averted) in **Cape Verde** is as follows:

- **Estimates from Pichon-Riviere et al. (Lower Bound):** USD $NaN
- **Estimates from Pichon-Riviere et al. (Upper Bound):** USD $NaN
- **CET equal to per capita GDP:** USD $NaN
- **CET equal to 2.3 (LMICs) or 1.7 (HICs) times per capita GDP:** USD $NaN

⬆ Back to Table of Contents

Anaemia Reduction in Central African Republic

National Target

For our baseline CET = 1 x GDP per capita, the national target for anaemia in **Central African Republic** should be a reduction in prevalence of **0%**. This target is estimated with a 95% uncertainty interval from **0%** to **14%**.

For CET based on Pichon-Riviere, the national target for anaemia in **Central African Republic** should be a reduction in prevalence of **0%**. This target is estimated with a 95% uncertainty interval from **0%** to **0%**.

For a near-infinite CET, the national target for anaemia in **Central African Republic** should be a reduction in prevalence of **14%**. This target is estimated with a 95% uncertainty interval from **7%** to **22%**.

Input parameters

- Among women of reproductive age in **Central African Republic**, the prevalence of overall anaemia is **38.4%**
  - **Mild anaemia:** 20.4%
  - **Moderate anaemia:** 16.6%
  - **Severe anaemia:** 1.4%
- The prevalence of malaria is **32.7%**.

Intervention coverage and costs

Iron supplementation (antenatal care)

- **Current coverage:** 77.9%
- **Maximum feasible coverage:** 82.9%
- **Estimated unit cost:** USD $2.63 (1.12 - 4.82)

Iron supplementation (all women of reproductive age)

- **Current coverage:** 32.1%
- **Maximum feasible coverage:** 77.9%
- **Estimated unit cost:** USD $2.71 (1.37 - 6.07)

Antenatal preventative IPTp-SP

- **Current coverage:** 53.1%
- **Maximum feasible coverage:** 66.8%
- **Estimated unit cost:** USD $1.40 (0.73 - 2.73)

Staple food supplementation

- **Current coverage:** 1.2%
- **Maximum feasible coverage:** 28.9%
- **Estimated unit cost:** USD $0.38 (0.11 - 1.34)

Cost-Effectiveness Analysis

The estimated cost per Year of Life Disabled (YLD) averted for each intervention in **Central African Republic** is given below, with interquartile ranges (IQR):

Iron supplementation (antenatal care)

- **Median Cost per YLD:** USD $592
- **IQR:** USD $382 – >USD 100k

Iron supplementation (all women of reproductive age)

- **Median Cost per YLD:** USD $1320
- **IQR:** USD $936 – $1857

Antenatal preventative IPTp-SP

- **Median Cost per YLD:** USD $1516
- **IQR:** USD $979 – >USD 100k

Staple food supplementation

- **Median Cost per YLD:** USD $759
- **IQR:** USD $475 – $1221

Economic Considerations

Cost-effectiveness thresholds (CET, in USD per DALY averted) in **Central African Republic** is as follows:

- **Estimates from Pichon-Riviere et al. (Lower Bound):** USD $45
- **Estimates from Pichon-Riviere et al. (Upper Bound):** USD $134
- **CET equal to per capita GDP:** USD $445
- **CET equal to 2.3 (LMICs) or 1.7 (HICs) times per capita GDP:** USD $1024

⬆ Back to Table of Contents

Anaemia Reduction in Chad

National Target

For our baseline CET = 1 x GDP per capita, the national target for anaemia in **Chad** should be a reduction in prevalence of **3%**. This target is estimated with a 95% uncertainty interval from **0%** to **14%**.

For CET based on Pichon-Riviere, the national target for anaemia in **Chad** should be a reduction in prevalence of **0%**. This target is estimated with a 95% uncertainty interval from **0%** to **0%**.

For a near-infinite CET, the national target for anaemia in **Chad** should be a reduction in prevalence of **9%**. This target is estimated with a 95% uncertainty interval from **5%** to **16%**.

Input parameters

- Among women of reproductive age in **Chad**, the prevalence of overall anaemia is **59.1%**
  - **Mild anaemia:** 31.5%
  - **Moderate anaemia:** 25.7%
  - **Severe anaemia:** 1.9%
- The prevalence of malaria is **14.1%**.

Intervention coverage and costs

Iron supplementation (antenatal care)

- **Current coverage:** 57.2%
- **Maximum feasible coverage:** 66.3%
- **Estimated unit cost:** USD $2.49 (1.10 - 4.89)

Iron supplementation (all women of reproductive age)

- **Current coverage:** 23.6%
- **Maximum feasible coverage:** 66.3%
- **Estimated unit cost:** USD $2.85 (1.86 - 9.56)

Antenatal preventative IPTp-SP

- **Current coverage:** 16.4%
- **Maximum feasible coverage:** 66.3%
- **Estimated unit cost:** USD $1.24 (0.65 - 2.52)

Staple food supplementation

- **Current coverage:** 0.0%
- **Maximum feasible coverage:** 6.5%
- **Estimated unit cost:** USD $0.45 (0.11 - 1.56)

Cost-Effectiveness Analysis

The estimated cost per Year of Life Disabled (YLD) averted for each intervention in **Chad** is given below, with interquartile ranges (IQR):

Iron supplementation (antenatal care)

- **Median Cost per YLD:** USD $328
- **IQR:** USD $222 – $592

Iron supplementation (all women of reproductive age)

- **Median Cost per YLD:** USD $1140
- **IQR:** USD $770 – $1734

Antenatal preventative IPTp-SP

- **Median Cost per YLD:** USD $1103
- **IQR:** USD $834 – $1503

Staple food supplementation

- **Median Cost per YLD:** USD $602
- **IQR:** USD $397 – $959

Economic Considerations

Cost-effectiveness thresholds (CET, in USD per DALY averted) in **Chad** is as follows:

- **Estimates from Pichon-Riviere et al. (Lower Bound):** USD $36
- **Estimates from Pichon-Riviere et al. (Upper Bound):** USD $108
- **CET equal to per capita GDP:** USD $719
- **CET equal to 2.3 (LMICs) or 1.7 (HICs) times per capita GDP:** USD $1655

⬆ Back to Table of Contents

Anaemia Reduction in Chile

National Target

For our baseline CET = 1 x GDP per capita, the national target for anaemia in **Chile** should be a reduction in prevalence of **7%**. This target is estimated with a 95% uncertainty interval from **0%** to **20%**.

For CET based on Pichon-Riviere, the national target for anaemia in **Chile** should be a reduction in prevalence of **7%**. This target is estimated with a 95% uncertainty interval from **0%** to **19%**.

For a near-infinite CET, the national target for anaemia in **Chile** should be a reduction in prevalence of **16%**. This target is estimated with a 95% uncertainty interval from **7%** to **29%**.

Input parameters

- Among women of reproductive age in **Chile**, the prevalence of overall anaemia is **8.1%**
    - **Mild anaemia:** 6.4%
    - **Moderate anaemia:** 1.7%
    - **Severe anaemia:** 0.1%
- There is no data on malaria prevalence; assume 0%.

Intervention coverage and costs

Iron supplementation (antenatal care)

- **Current coverage:** 81.0%
- **Maximum feasible coverage:** 100.0%
- **Estimated unit cost:** USD $56.88 (17.56 - 91.77)

Iron supplementation (all women of reproductive age)

- **Current coverage:** 33.4%
- **Maximum feasible coverage:** 100.0%
- **Estimated unit cost:** USD $33.71 (8.97 - 45.91)

Antenatal preventative IPTp-SP

- **Current coverage:** 0.0%
- **Maximum feasible coverage:** 100.0%
- **Estimated unit cost:** USD $14.81 (7.47 - 23.08)

Staple food supplementation

- **Current coverage:** 61.4%
- **Maximum feasible coverage:** 85.0%
- **Estimated unit cost:** USD $0.38 (0.23 - 0.64)

Cost-Effectiveness Analysis

The estimated cost per Year of Life Disabled (YLD) averted for each intervention in **Chile** is given below, with interquartile ranges (IQR):

Iron supplementation (antenatal care)

- **Median Cost per YLD:** USD $85481
- **IQR:** USD $53955 – >USD 100k

Iron supplementation (all women of reproductive age)

- **Median Cost per YLD:** >USD 100k
- **IQR:** USD $73759 – >USD 100k

Antenatal preventative IPTp-SP

- **Median Cost per YLD:** >USD 100k
- **IQR:** >USD 100k

Staple food supplementation

- **Median Cost per YLD:** USD $4620
- **IQR:** USD $3042 – $7384

Economic Considerations

Cost-effectiveness thresholds (CET, in USD per DALY averted) in **Chile** is as follows:

- **Estimates from Pichon-Riviere et al. (Lower Bound):** USD $11623
- **Estimates from Pichon-Riviere et al. (Upper Bound):** USD $13675
- **CET equal to per capita GDP:** USD $17093
- **CET equal to 2.3 (LMICs) or 1.7 (HICs) times per capita GDP:** USD $23931

⬆ Back to Table of Contents

Anaemia Reduction in China

National Target

For our baseline CET = 1 x GDP per capita, the national target for anaemia in **China** should be a reduction in prevalence of **29%**. This target is estimated with a 95% uncertainty interval from **12%** to **47%**.

For CET based on Pichon-Riviere, the national target for anaemia in **China** should be a reduction in prevalence of **24%**. This target is estimated with a 95% uncertainty interval from **0%** to **45%**.

For a near-infinite CET, the national target for anaemia in **China** should be a reduction in prevalence of **30%**. This target is estimated with a 95% uncertainty interval from **16%** to **48%**.

Input parameters

- Among women of reproductive age in **China**, the prevalence of overall anaemia is **15.8%**
  - **Mild anaemia:** 9.6%
  - **Moderate anaemia:** 5.7%
  - **Severe anaemia:** 0.5%
- The prevalence of malaria is **0.0%**.

Intervention coverage and costs

Iron supplementation (antenatal care)

- **Current coverage:** 84.2%
- **Maximum feasible coverage:** 93.0%
- **Estimated unit cost:** USD $9.15 (4.52 - 15.85)

Iron supplementation (all women of reproductive age)

- **Current coverage:** 34.7%
- **Maximum feasible coverage:** 93.0%
- **Estimated unit cost:** USD $5.66 (2.84 - 11.93)

Antenatal preventative IPTp-SP

- **Current coverage:** 0.0%
- **Maximum feasible coverage:** 93.0%
- **Estimated unit cost:** USD $6.70 (3.42 - 11.66)

Staple food supplementation

- **Current coverage:** 3.3%
- **Maximum feasible coverage:** 100.0%
- **Estimated unit cost:** USD $0.58 (0.14 - 1.56)

Cost-Effectiveness Analysis

The estimated cost per Year of Life Disabled (YLD) averted for each intervention in **China** is given below, with interquartile ranges (IQR):

Iron supplementation (antenatal care)

- **Median Cost per YLD:** USD $5493
- **IQR:** USD $3668 – >USD 100k

Iron supplementation (all women of reproductive age)

- **Median Cost per YLD:** USD $8227
- **IQR:** USD $5957 – $10831

Antenatal preventative IPTp-SP

- **Median Cost per YLD:** >USD 100k
- **IQR:** >USD 100k

Staple food supplementation

- **Median Cost per YLD:** USD $3236
- **IQR:** USD $2237 – $4679

Economic Considerations

Cost-effectiveness thresholds (CET, in USD per DALY averted) in **China** is as follows:

- **Estimates from Pichon-Riviere et al. (Lower Bound):** USD $4667
- **Estimates from Pichon-Riviere et al. (Upper Bound):** USD $6433
- **CET equal to per capita GDP:** USD $12614
- **CET equal to 2.3 (LMICs) or 1.7 (HICs) times per capita GDP:** USD $29012

⬆ Back to Table of Contents

Anaemia Reduction in Colombia

National Target

For our baseline CET = 1 x GDP per capita, the national target for anaemia in **Colombia** should be a reduction in prevalence of **1%**. This target is estimated with a 95% uncertainty interval from **0%** to **27%**.

For CET based on Pichon-Riviere, the national target for anaemia in **Colombia** should be a reduction in prevalence of **0%**. This target is estimated with a 95% uncertainty interval from **0%** to **24%**.

For a near-infinite CET, the national target for anaemia in **Colombia** should be a reduction in prevalence of **20%**. This target is estimated with a 95% uncertainty interval from **11%** to **32%**.

Input parameters

- Among women of reproductive age in **Colombia**, the prevalence of overall anaemia is **12.0%**
  - **Mild anaemia:** 8.2%
  - **Moderate anaemia:** 3.6%
  - **Severe anaemia:** 0.2%
- The prevalence of malaria is **0.1%**.

Intervention coverage and costs

Iron supplementation (antenatal care)

- **Current coverage:** 93.8%
- **Maximum feasible coverage:** 97.5%

- **Estimated unit cost:** USD $12.77 (6.30 - 20.36)

Iron supplementation (all women of reproductive age)

- **Current coverage:** 38.7%
- **Maximum feasible coverage:** 97.5%
- **Estimated unit cost:** USD $6.86 (3.43 - 12.78)

Antenatal preventative IPTp-SP

- **Current coverage:** 0.1%
- **Maximum feasible coverage:** 97.5%
- **Estimated unit cost:** USD $9.16 (4.65 - 14.51)

Staple food supplementation

- **Current coverage:** 7.4%
- **Maximum feasible coverage:** 58.5%
- **Estimated unit cost:** USD $1.22 (0.78 - 2.24)

Cost-Effectiveness Analysis

The estimated cost per Year of Life Disabled (YLD) averted for each intervention in **Colombia** is given below, with interquartile ranges (IQR):

Iron supplementation (antenatal care)

- **Median Cost per YLD:** USD $14117
- **IQR:** USD $7446 – >USD 100k

Iron supplementation (all women of reproductive age)

- **Median Cost per YLD:** USD $13266
- **IQR:** USD $9479 – $19014

Antenatal preventative IPTp-SP

- **Median Cost per YLD:** >USD 100k
- **IQR:** >USD 100k

Staple food supplementation

- **Median Cost per YLD:** USD $7731
- **IQR:** USD $5399 – $11447

Economic Considerations

Cost-effectiveness thresholds (CET, in USD per DALY averted) in **Colombia** is as follows:

- **Estimates from Pichon-Riviere et al. (Lower Bound):** USD $3839
- **Estimates from Pichon-Riviere et al. (Upper Bound):** USD $5235
- **CET equal to per capita GDP:** USD $6980
- **CET equal to 2.3 (LMICs) or 1.7 (HICs) times per capita GDP:** USD $16053

⬆ Back to Table of Contents

Anaemia Reduction in Comoros

National Target

For our baseline CET = 1 x GDP per capita, the national target for anaemia in **Comoros** should be a reduction in prevalence of **18%**. This target is estimated with a 95% uncertainty interval from **0%** to **41%**.

For CET based on Pichon-Riviere, the national target for anaemia in **Comoros** should be a reduction in prevalence of **0%**. This target is estimated with a 95% uncertainty interval from **0%** to **31%**.

For a near-infinite CET, the national target for anaemia in **Comoros** should be a reduction in prevalence of **27%**. This target is estimated with a 95% uncertainty interval from **15%** to **43%**.

Input parameters

- Among women of reproductive age in **Comoros**, the prevalence of overall anaemia is **38.4%**
  - **Mild anaemia:** 25.1%
  - **Moderate anaemia:** 12.7%
  - **Severe anaemia:** 0.6%
- The prevalence of malaria is **0.6%**.

Intervention coverage and costs

Iron supplementation (antenatal care)

- **Current coverage:** 78.7%
- **Maximum feasible coverage:** 92.0%
- **Estimated unit cost:** USD $2.82 (1.22 - 5.15)

Iron supplementation (all women of reproductive age)

- **Current coverage:** 32.4%
- **Maximum feasible coverage:** 92.0%
- **Estimated unit cost:** USD $2.79 (1.38 - 6.75)

Antenatal preventative IPTp-SP

- **Current coverage:** 27.8%
- **Maximum feasible coverage:** 92.0%
- **Estimated unit cost:** USD $1.53 (0.79 - 2.96)

Staple food supplementation

- **Current coverage:** 5.7%
- **Maximum feasible coverage:** 82.1%
- **Estimated unit cost:** USD $0.62 (0.09 - 1.91)

Cost-Effectiveness Analysis

The estimated cost per Year of Life Disabled (YLD) averted for each intervention in **Comoros** is given below, with interquartile ranges (IQR):

Iron supplementation (antenatal care)

- **Median Cost per YLD:** USD $662
- **IQR:** USD $430 – $1221

Iron supplementation (all women of reproductive age)

- **Median Cost per YLD:** USD $1756
- **IQR:** USD $1241 – $2595

Antenatal preventative IPTp-SP

- **Median Cost per YLD:** USD $34503
- **IQR:** USD $25091 – $45969

Staple food supplementation

- **Median Cost per YLD:** USD $1290
- **IQR:** USD $846 – $2089

Economic Considerations

Cost-effectiveness thresholds (CET, in USD per DALY averted) in **Comoros** is as follows:

- **Estimates from Pichon-Riviere et al. (Lower Bound):** USD $286
- **Estimates from Pichon-Riviere et al. (Upper Bound):** USD $540
- **CET equal to per capita GDP:** USD $1587
- **CET equal to 2.3 (LMICs) or 1.7 (HICs) times per capita GDP:** USD $3650

⬆ Back to Table of Contents

Anaemia Reduction in Congo - Brazzaville

National Target

For our baseline CET = 1 x GDP per capita, the national target for anaemia in **Congo - Brazzaville** should be a reduction in prevalence of **17%**. This target is estimated with a 95% uncertainty interval from **6%** to **33%**.

For CET based on Pichon-Riviere, the national target for anaemia in **Congo - Brazzaville** should be a reduction in prevalence of **10%**. This target is estimated with a 95% uncertainty interval from **0%** to **27%**.

For a near-infinite CET, the national target for anaemia in **Congo - Brazzaville** should be a reduction in prevalence of **20%**. This target is estimated with a 95% uncertainty interval from **9%** to **34%**.

Input parameters

- Among women of reproductive age in **Congo - Brazzaville**, the prevalence of overall anaemia is **48.3%**
  - **Mild anaemia:** 24.7%
  - **Moderate anaemia:** 21.9%
  - **Severe anaemia:** 1.7%
- There is no data on malaria prevalence; assume 0%.

Intervention coverage and costs

Iron supplementation (antenatal care)

- **Current coverage:** NaN%
- **Maximum feasible coverage:** NaN%
- **Estimated unit cost:** USD $NaN (NaN - NaN)

Iron supplementation (all women of reproductive age)

- **Current coverage:** NaN%
- **Maximum feasible coverage:** NaN%
- **Estimated unit cost:** USD $NaN (NaN - NaN)

Antenatal preventative IPTp-SP

- **Current coverage:** NaN%
- **Maximum feasible coverage:** NaN%
- **Estimated unit cost:** USD $NaN (NaN - NaN)

Staple food supplementation

- **Current coverage:** NaN%
- **Maximum feasible coverage:** NaN%
- **Estimated unit cost:** USD $NaN (NaN - NaN)

Cost-Effectiveness Analysis

The estimated cost per Year of Life Disabled (YLD) averted for each intervention in **Congo - Brazzaville** is given below, with interquartile ranges (IQR):

Iron supplementation (antenatal care)

- **Median Cost per YLD:** USD $1146
- **IQR:** USD $580 – >USD 100k

Iron supplementation (all women of reproductive age)

- **Median Cost per YLD:** USD $1845
- **IQR:** USD $1269 – $2669

Antenatal preventative IPTp-SP

- **Median Cost per YLD:** USD $3373
- **IQR:** USD $2601 – $4301

Staple food supplementation

- **Median Cost per YLD:** USD $291
- **IQR:** USD $181 – $453

Economic Considerations

Cost-effectiveness thresholds (CET, in USD per DALY averted) in **Congo - Brazzaville** is as follows:

- **Estimates from Pichon-Riviere et al. (Lower Bound):** USD $NaN
- **Estimates from Pichon-Riviere et al. (Upper Bound):** USD $NaN
- **CET equal to per capita GDP:** USD $NaN
- **CET equal to 2.3 (LMICs) or 1.7 (HICs) times per capita GDP:** USD $NaN

⬆ Back to Table of Contents

Anaemia Reduction in Congo - Kinshasa

National Target

For our baseline CET = 1 x GDP per capita, the national target for anaemia in **Congo - Kinshasa** should be a reduction in prevalence of **1%**. This target is estimated with a 95% uncertainty interval from **0%** to **43%**.

For CET based on Pichon-Riviere, the national target for anaemia in **Congo - Kinshasa** should be a reduction in prevalence of **0%**. This target is estimated with a 95% uncertainty interval from **0%** to **0%**.

For a near-infinite CET, the national target for anaemia in **Congo - Kinshasa** should be a reduction in prevalence of **27%**. This target is estimated with a 95% uncertainty interval from **14%** to **46%**.

Input parameters

- Among women of reproductive age in **Congo - Kinshasa**, the prevalence of overall anaemia is **46.3%**
  - **Mild anaemia:** 27.0%
  - **Moderate anaemia:** 18.3%
  - **Severe anaemia:** 1.0%
- There is no data on malaria prevalence; assume 0%.

Intervention coverage and costs

Iron supplementation (antenatal care)

- **Current coverage:** NaN%
- **Maximum feasible coverage:** NaN%
- **Estimated unit cost:** USD $NaN (NaN - NaN)

Iron supplementation (all women of reproductive age)

- **Current coverage:** NaN%
- **Maximum feasible coverage:** NaN%
- **Estimated unit cost:** USD $NaN (NaN - NaN)

Antenatal preventative IPTp-SP

- **Current coverage:** NaN%

- **Maximum feasible coverage:** NaN%
- **Estimated unit cost:** USD $NaN (NaN - NaN)

Staple food supplementation

- **Current coverage:** NaN%
- **Maximum feasible coverage:** NaN%
- **Estimated unit cost:** USD $NaN (NaN - NaN)

Cost-Effectiveness Analysis

The estimated cost per Year of Life Disabled (YLD) averted for each intervention in **Congo - Kinshasa** is given below, with interquartile ranges (IQR):

Iron supplementation (antenatal care)

- **Median Cost per YLD:** USD $624
- **IQR:** USD $398 – >USD 100k

Iron supplementation (all women of reproductive age)

- **Median Cost per YLD:** USD $1309
- **IQR:** USD $924 – $1872

Antenatal preventative IPTp-SP

- **Median Cost per YLD:** USD $1676
- **IQR:** USD $1065 – >USD 100k

Staple food supplementation

- **Median Cost per YLD:** USD $874
- **IQR:** USD $550 – $1336

Economic Considerations

Cost-effectiveness thresholds (CET, in USD per DALY averted) in **Congo - Kinshasa** is as follows:

- **Estimates from Pichon-Riviere et al. (Lower Bound):** USD $NaN
- **Estimates from Pichon-Riviere et al. (Upper Bound):** USD $NaN
- **CET equal to per capita GDP:** USD $NaN
- **CET equal to 2.3 (LMICs) or 1.7 (HICs) times per capita GDP:** USD $NaN

⬆ Back to Table of Contents

Anaemia Reduction in Costa Rica

National Target

For our baseline CET = 1 x GDP per capita, the national target for anaemia in **Costa Rica** should be a reduction in prevalence of **18%**. This target is estimated with a 95% uncertainty interval from **0%** to **34%**.

For CET based on Pichon-Riviere, the national target for anaemia in **Costa Rica** should be a reduction in prevalence of **16%**. This target is estimated with a 95% uncertainty interval from **0%** to **33%**.

For a near-infinite CET, the national target for anaemia in **Costa Rica** should be a reduction in prevalence of **22%**. This target is estimated with a 95% uncertainty interval from **12%** to **35%**.

Input parameters

- Among women of reproductive age in **Costa Rica**, the prevalence of overall anaemia is **18.3%**
  - **Mild anaemia:** 15.2%
  - **Moderate anaemia:** 3.0%
  - **Severe anaemia:** 0.1%
- The prevalence of malaria is **0.0%**.

Intervention coverage and costs

Iron supplementation (antenatal care)

- **Current coverage:** 88.3%
- **Maximum feasible coverage:** 94.0%
- **Estimated unit cost:** USD $16.48 (8.17 - 26.20)

Iron supplementation (all women of reproductive age)

- **Current coverage:** 36.4%
- **Maximum feasible coverage:** 94.0%
- **Estimated unit cost:** USD $8.48 (4.25 - 15.56)

Antenatal preventative IPTp-SP

- **Current coverage:** 0.1%
- **Maximum feasible coverage:** 94.0%
- **Estimated unit cost:** USD $12.03 (6.08 - 19.01)

Staple food supplementation

- **Current coverage:** 1.5%
- **Maximum feasible coverage:** 60.9%
- **Estimated unit cost:** USD $0.73 (0.34 - 1.63)

Cost-Effectiveness Analysis

The estimated cost per Year of Life Disabled (YLD) averted for each intervention in **Costa Rica** is given below, with interquartile ranges (IQR):

Iron supplementation (antenatal care)

- **Median Cost per YLD:** USD $20315
- **IQR:** USD $9617 – >USD 100k

Iron supplementation (all women of reproductive age)

- **Median Cost per YLD:** USD $17049
- **IQR:** USD $11191 – $27599

Antenatal preventative IPTp-SP

- **Median Cost per YLD:** >USD 100k
- **IQR:** >USD 100k

Staple food supplementation

- **Median Cost per YLD:** USD $5030
- **IQR:** USD $3216 – $8490

Economic Considerations

Cost-effectiveness thresholds (CET, in USD per DALY averted) in **Costa Rica** is as follows:

- **Estimates from Pichon-Riviere et al. (Lower Bound):** USD $2987
- **Estimates from Pichon-Riviere et al. (Upper Bound):** USD $12447
- **CET equal to per capita GDP:** USD $16595
- **CET equal to 2.3 (LMICs) or 1.7 (HICs) times per capita GDP:** USD $38169

⬆ Back to Table of Contents

Anaemia Reduction in Croatia

National Target

For our baseline CET = 1 x GDP per capita, the national target for anaemia in **Croatia** should be a reduction in prevalence of **18%**. This target is estimated with a 95% uncertainty interval from **6%** to **35%**.

For CET based on Pichon-Riviere, the national target for anaemia in **Croatia** should be a reduction in prevalence of **16%**. This target is estimated with a 95% uncertainty interval from **6%** to **33%**.

For a near-infinite CET, the national target for anaemia in **Croatia** should be a reduction in prevalence of **24%**. This target is estimated with a 95% uncertainty interval from **13%** to **40%**.

Input parameters

- Among women of reproductive age in **Croatia**, the prevalence of overall anaemia is **19.3%**
  - **Mild anaemia:** 13.0%
  - **Moderate anaemia:** 6.0%
  - **Severe anaemia:** 0.3%
- There is no data on malaria prevalence; assume 0%.

Intervention coverage and costs

Iron supplementation (antenatal care)

- **Current coverage:** 81.0%
- **Maximum feasible coverage:** 97.0%
- **Estimated unit cost:** USD $59.13 (18.71 - 92.89)

Iron supplementation (all women of reproductive age)

- **Current coverage:** 33.4%
- **Maximum feasible coverage:** 97.0%
- **Estimated unit cost:** USD $34.29 (11.45 - 64.57)

Antenatal preventative IPTp-SP

- **Current coverage:** 0.0%
- **Maximum feasible coverage:** 97.0%
- **Estimated unit cost:** USD $14.93 (7.53 - 23.21)

Staple food supplementation

- **Current coverage:** 35.6%
- **Maximum feasible coverage:** 100.0%
- **Estimated unit cost:** USD $0.28 (0.18 - 0.43)

Cost-Effectiveness Analysis

The estimated cost per Year of Life Disabled (YLD) averted for each intervention in **Croatia** is given below, with interquartile ranges (IQR):

Iron supplementation (antenatal care)

- **Median Cost per YLD:** USD $25863
- **IQR:** USD $16444 – $42833

Iron supplementation (all women of reproductive age)

- **Median Cost per YLD:** USD $37409
- **IQR:** USD $25779 – $55259

Antenatal preventative IPTp-SP

- **Median Cost per YLD:** >USD 100k
- **IQR:** >USD 100k

Staple food supplementation

- **Median Cost per YLD:** USD $1140
- **IQR:** USD $790 – $1670

Economic Considerations

Cost-effectiveness thresholds (CET, in USD per DALY averted) in **Croatia** is as follows:

- **Estimates from Pichon-Riviere et al. (Lower Bound):** USD $3863
- **Estimates from Pichon-Riviere et al. (Upper Bound):** USD $12232
- **CET equal to per capita GDP:** USD $21460
- **CET equal to 2.3 (LMICs) or 1.7 (HICs) times per capita GDP:** USD $30044

⬆ Back to Table of Contents

Anaemia Reduction in Cuba

National Target

For our baseline CET = 1 x GDP per capita, the national target for anaemia in **Cuba** should be a reduction in prevalence of **25%**. This target is estimated with a 95% uncertainty interval from **11%** to **42%**.

For CET based on Pichon-Riviere, the national target for anaemia in **Cuba** should be a reduction in prevalence of **25%**. This target is estimated with a 95% uncertainty interval from **11%** to **42%**.

For a near-infinite CET, the national target for anaemia in **Cuba** should be a reduction in prevalence of **25%**. This target is estimated with a 95% uncertainty interval from **13%** to **43%**.

Input parameters

- Among women of reproductive age in **Cuba**, the prevalence of overall anaemia is **30.2%**
  - **Mild anaemia:** 18.9%
  - **Moderate anaemia:** 10.7%
  - **Severe anaemia:** 0.6%
- There is no data on malaria prevalence; assume 0%.

Intervention coverage and costs

Iron supplementation (antenatal care)

- **Current coverage:** 88.3%
- **Maximum feasible coverage:** 93.3%
- **Estimated unit cost:** USD $8.85 (4.35 - 14.63)

Iron supplementation (all women of reproductive age)

- **Current coverage:** 36.4%
- **Maximum feasible coverage:** 88.3%
- **Estimated unit cost:** USD $5.62 (2.82 - 11.16)

Antenatal preventative IPTp-SP

- **Current coverage:** 0.1%
- **Maximum feasible coverage:** 79.0%
- **Estimated unit cost:** USD $6.25 (3.19 - 10.23)

Staple food supplementation

- **Current coverage:** 23.7%
- **Maximum feasible coverage:** 100.0%
- **Estimated unit cost:** USD $0.93 (0.50 - 1.93)

Cost-Effectiveness Analysis

The estimated cost per Year of Life Disabled (YLD) averted for each intervention in **Cuba** is given below, with interquartile ranges (IQR):

Iron supplementation (antenatal care)

- **Median Cost per YLD:** USD $3393
- **IQR:** USD $1769 – >USD 100k

Iron supplementation (all women of reproductive age)

- **Median Cost per YLD:** USD $3761
- **IQR:** USD $2615 – $5581

Antenatal preventative IPTp-SP

- **Median Cost per YLD:** >USD 100k
- **IQR:** >USD 100k

Staple food supplementation

- **Median Cost per YLD:** USD $2350
- **IQR:** USD $1568 – $3423

Economic Considerations

Cost-effectiveness thresholds (CET, in USD per DALY averted) in **Cuba** is as follows:

- **Estimates from Pichon-Riviere et al. (Lower Bound):** USD $1710
- **Estimates from Pichon-Riviere et al. (Upper Bound):** USD $11114
- **CET equal to per capita GDP:** USD $9500
- **CET equal to 2.3 (LMICs) or 1.7 (HICs) times per capita GDP:** USD $21849

⬆ Back to Table of Contents

Anaemia Reduction in Cyprus

National Target

For our baseline CET = 1 x GDP per capita, the national target for anaemia in **Cyprus** should be a reduction in prevalence of **16%**. This target is estimated with a 95% uncertainty interval from **6%** to **33%**.

For CET based on Pichon-Riviere, the national target for anaemia in **Cyprus** should be a reduction in prevalence of **16%**. This target is estimated with a 95% uncertainty interval from **6%** to **32%**.

For a near-infinite CET, the national target for anaemia in **Cyprus** should be a reduction in prevalence of **25%**. This target is estimated with a 95% uncertainty interval from **13%** to **40%**.

Input parameters

- Among women of reproductive age in **Cyprus**, the prevalence of overall anaemia is **8.4%**
  - **Mild anaemia:** 6.9%
  - **Moderate anaemia:** 1.5%
  - **Severe anaemia:** 0.0%
- There is no data on malaria prevalence; assume 0%.

Intervention coverage and costs

Iron supplementation (antenatal care)

- **Current coverage:** 81.0%
- **Maximum feasible coverage:** 100.0%
- **Estimated unit cost:** USD $116.37 (47.14 - 184.78)

Iron supplementation (all women of reproductive age)

- **Current coverage:** 33.4%
- **Maximum feasible coverage:** 100.0%
- **Estimated unit cost:** USD $74.75 (29.33 - 113.74)

Antenatal preventative IPTp-SP

- **Current coverage:** 0.0%
- **Maximum feasible coverage:** 100.0%
- **Estimated unit cost:** USD $60.06 (30.10 - 94.76)

Staple food supplementation

- **Current coverage:** 36.6%
- **Maximum feasible coverage:** 100.0%
- **Estimated unit cost:** USD $0.18 (0.07 - 0.34)

Cost-Effectiveness Analysis

The estimated cost per Year of Life Disabled (YLD) averted for each intervention in **Cyprus** is given below, with interquartile ranges (IQR):

Iron supplementation (antenatal care)

- **Median Cost per YLD:** >USD 100k
- **IQR:** >USD 100k

Iron supplementation (all women of reproductive age)

- **Median Cost per YLD:** >USD 100k
- **IQR:** >USD 100k

Antenatal preventative IPTp-SP

- **Median Cost per YLD:** >USD 100k
- **IQR:** >USD 100k

Staple food supplementation

- **Median Cost per YLD:** USD $2516
- **IQR:** USD $1583 – $3969

Economic Considerations

Cost-effectiveness thresholds (CET, in USD per DALY averted) in **Cyprus** is as follows:

- **Estimates from Pichon-Riviere et al. (Lower Bound):** USD $17004
- **Estimates from Pichon-Riviere et al. (Upper Bound):** USD $20127
- **CET equal to per capita GDP:** USD $34701

- **CET equal to 2.3 (LMICs) or 1.7 (HICs) times per capita GDP:** USD $48582

⬆ Back to Table of Contents

Anaemia Reduction in Czechia

National Target

For our baseline CET = 1 x GDP per capita, the national target for anaemia in **Czechia** should be a reduction in prevalence of **18%**. This target is estimated with a 95% uncertainty interval from **7%** to **36%**.

For CET based on Pichon-Riviere, the national target for anaemia in **Czechia** should be a reduction in prevalence of **16%**. This target is estimated with a 95% uncertainty interval from **6%** to **33%**.

For a near-infinite CET, the national target for anaemia in **Czechia** should be a reduction in prevalence of **24%**. This target is estimated with a 95% uncertainty interval from **13%** to **40%**.

Input parameters

- Among women of reproductive age in **Czechia**, the prevalence of overall anaemia is **19.4%**
  - **Mild anaemia:** 13.1%
  - **Moderate anaemia:** 6.0%
  - **Severe anaemia:** 0.3%
- There is no data on malaria prevalence; assume 0%.

Intervention coverage and costs

Iron supplementation (antenatal care)

- **Current coverage:** NaN%
- **Maximum feasible coverage:** NaN%
- **Estimated unit cost:** USD $71.30 (24.77 - 114.98)

Iron supplementation (all women of reproductive age)

- **Current coverage:** NaN%
- **Maximum feasible coverage:** NaN%

- **Estimated unit cost:** USD $44.51 (14.37 - 63.77)

Antenatal preventative IPTp-SP

- **Current coverage:** NaN%
- **Maximum feasible coverage:** NaN%
- **Estimated unit cost:** USD $26.66 (13.40 - 42.48)

Staple food supplementation

- **Current coverage:** NaN%
- **Maximum feasible coverage:** NaN%
- **Estimated unit cost:** USD $0.10 (0.01 - 0.23)

Cost-Effectiveness Analysis

The estimated cost per Year of Life Disabled (YLD) averted for each intervention in **Czechia** is given below, with interquartile ranges (IQR):

Iron supplementation (antenatal care)

- **Median Cost per YLD:** USD $30251
- **IQR:** USD $21039 – $46768

Iron supplementation (all women of reproductive age)

- **Median Cost per YLD:** USD $44807
- **IQR:** USD $32021 – $64107

Antenatal preventative IPTp-SP

- **Median Cost per YLD:** >USD 100k
- **IQR:** >USD 100k

Staple food supplementation

- **Median Cost per YLD:** USD $412
- **IQR:** USD $262 – $657

Economic Considerations

Cost-effectiveness thresholds (CET, in USD per DALY averted) in **Czechia** is as follows:

- **Estimates from Pichon-Riviere et al. (Lower Bound):** USD $17344
- **Estimates from Pichon-Riviere et al. (Upper Bound):** USD $20386
- **CET equal to per capita GDP:** USD $30427
- **CET equal to 2.3 (LMICs) or 1.7 (HICs) times per capita GDP:** USD $42598

⬆ Back to Table of Contents

Anaemia Reduction in Côte d'Ivoire

National Target

For our baseline CET = 1 x GDP per capita, the national target for anaemia in **Côte d'Ivoire** should be a reduction in prevalence of **25%**. This target is estimated with a 95% uncertainty interval from **11%** to **41%**.

For CET based on Pichon-Riviere, the national target for anaemia in **Côte d'Ivoire** should be a reduction in prevalence of **1%**. This target is estimated with a 95% uncertainty interval from **0%** to **32%**.

For a near-infinite CET, the national target for anaemia in **Côte d'Ivoire** should be a reduction in prevalence of **25%**. This target is estimated with a 95% uncertainty interval from **14%** to **41%**.

Input parameters

- Among women of reproductive age in **Côte d'Ivoire**, the prevalence of overall anaemia is **NaN%**
  - **Mild anaemia:** NaN%
  - **Moderate anaemia:** NaN%
  - **Severe anaemia:** NaN%
- There is no data on malaria prevalence; assume 0%.

Intervention coverage and costs

Iron supplementation (antenatal care)

- **Current coverage:** 86.4%
- **Maximum feasible coverage:** 95.4%
- **Estimated unit cost:** USD $NaN (NaN - NaN)

Iron supplementation (all women of reproductive age)

- **Current coverage:** 35.6%
- **Maximum feasible coverage:** 95.4%
- **Estimated unit cost:** USD $NaN (NaN - NaN)

Antenatal preventative IPTp-SP

- **Current coverage:** 38.2%
- **Maximum feasible coverage:** 95.4%
- **Estimated unit cost:** USD $NaN (NaN - NaN)

Staple food supplementation

- **Current coverage:** 3.1%
- **Maximum feasible coverage:** 72.0%
- **Estimated unit cost:** USD $NaN (NaN - NaN)

Cost-Effectiveness Analysis

The estimated cost per Year of Life Disabled (YLD) averted for each intervention in **Côte d'Ivoire** is given below, with interquartile ranges (IQR):

Iron supplementation (antenatal care)

- **Median Cost per YLD:** USD $437
- **IQR:** USD $273 – >USD 100k

Iron supplementation (all women of reproductive age)

- **Median Cost per YLD:** USD $961
- **IQR:** USD $660 – $1375

Antenatal preventative IPTp-SP

- **Median Cost per YLD:** USD $1133
- **IQR:** USD $883 – $1513

Staple food supplementation

- **Median Cost per YLD:** USD $803
- **IQR:** USD $507 – $1208

Economic Considerations

Cost-effectiveness thresholds (CET, in USD per DALY averted) in **Côte d'Ivoire** is as follows:

- **Estimates from Pichon-Riviere et al. (Lower Bound):** USD $NaN
- **Estimates from Pichon-Riviere et al. (Upper Bound):** USD $NaN
- **CET equal to per capita GDP:** USD $NaN
- **CET equal to 2.3 (LMICs) or 1.7 (HICs) times per capita GDP:** USD $NaN

⬆ Back to Table of Contents

Anaemia Reduction in Denmark

National Target

For our baseline CET = 1 x GDP per capita, the national target for anaemia in **Denmark** should be a reduction in prevalence of **17%**. This target is estimated with a 95% uncertainty interval from **6%** to **34%**.

For CET based on Pichon-Riviere, the national target for anaemia in **Denmark** should be a reduction in prevalence of **16%**. This target is estimated with a 95% uncertainty interval from **6%** to **34%**.

For a near-infinite CET, the national target for anaemia in **Denmark** should be a reduction in prevalence of **25%**. This target is estimated with a 95% uncertainty interval from **13%** to **40%**.

Input parameters

- Among women of reproductive age in **Denmark**, the prevalence of overall anaemia is **8.3%**
  - **Mild anaemia:** 6.9%
  - **Moderate anaemia:** 1.3%

    - **Severe anaemia:** 0.0%
- There is no data on malaria prevalence; assume 0%.

Intervention coverage and costs

Iron supplementation (antenatal care)

- **Current coverage:** 81.0%
- **Maximum feasible coverage:** 100.0%
- **Estimated unit cost:** USD $110.12 (44.18 - 172.62)

Iron supplementation (all women of reproductive age)

- **Current coverage:** 33.4%
- **Maximum feasible coverage:** 100.0%
- **Estimated unit cost:** USD $69.11 (28.92 - 119.69)

Antenatal preventative IPTp-SP

- **Current coverage:** 0.0%
- **Maximum feasible coverage:** 100.0%
- **Estimated unit cost:** USD $54.96 (27.55 - 86.21)

Staple food supplementation

- **Current coverage:** 34.9%
- **Maximum feasible coverage:** 100.0%
- **Estimated unit cost:** USD $0.31 (0.18 - 0.53)

Cost-Effectiveness Analysis

The estimated cost per Year of Life Disabled (YLD) averted for each intervention in **Denmark** is given below, with interquartile ranges (IQR):

Iron supplementation (antenatal care)

- **Median Cost per YLD:** >USD 100k
- **IQR:** >USD 100k

Iron supplementation (all women of reproductive age)

- **Median Cost per YLD:** >USD 100k
- **IQR:** >USD 100k

Antenatal preventative IPTp-SP

- **Median Cost per YLD:** >USD 100k
- **IQR:** >USD 100k

Staple food supplementation

- **Median Cost per YLD:** USD $5388
- **IQR:** USD $3589 – $8489

Economic Considerations

Cost-effectiveness thresholds (CET, in USD per DALY averted) in **Denmark** is as follows:

- **Estimates from Pichon-Riviere et al. (Lower Bound):** USD $50976
- **Estimates from Pichon-Riviere et al. (Upper Bound):** USD $59811
- **CET equal to per capita GDP:** USD $67967
- **CET equal to 2.3 (LMICs) or 1.7 (HICs) times per capita GDP:** USD $95154

⬆ Back to Table of Contents

Anaemia Reduction in Djibouti

National Target

For our baseline CET = 1 x GDP per capita, the national target for anaemia in **Djibouti** should be a reduction in prevalence of **9%**. This target is estimated with a 95% uncertainty interval from **1%** to **20%**.

For CET based on Pichon-Riviere, the national target for anaemia in **Djibouti** should be a reduction in prevalence of **0%**. This target is estimated with a 95% uncertainty interval from **0%** to **11%**.

For a near-infinite CET, the national target for anaemia in **Djibouti** should be a reduction in prevalence of **10%**. This target is estimated with a 95% uncertainty interval from **4%** to **20%**.

Input parameters

- Among women of reproductive age in **Djibouti**, the prevalence of overall anaemia is **37.3%**
  - **Mild anaemia:** 25.2%
  - **Moderate anaemia:** 11.7%
  - **Severe anaemia:** 0.5%
- The prevalence of malaria is **10.5%**.

Intervention coverage and costs

Iron supplementation (antenatal care)

- **Current coverage:** 58.4%
- **Maximum feasible coverage:** 76.8%
- **Estimated unit cost:** USD $2.78 (1.26 - 5.20)

Iron supplementation (all women of reproductive age)

- **Current coverage:** 24.1%
- **Maximum feasible coverage:** 76.8%
- **Estimated unit cost:** USD $3.47 (1.73 - 7.44)

Antenatal preventative IPTp-SP

- **Current coverage:** 0.0%
- **Maximum feasible coverage:** 76.8%
- **Estimated unit cost:** USD $1.62 (0.86 - 3.13)

Staple food supplementation

- **Current coverage:** 90.2%
- **Maximum feasible coverage:** 100.0%
- **Estimated unit cost:** USD $0.32 (0.05 - 0.97)

Cost-Effectiveness Analysis

The estimated cost per Year of Life Disabled (YLD) averted for each intervention in **Djibouti** is given below, with interquartile ranges (IQR):

Iron supplementation (antenatal care)

- **Median Cost per YLD:** USD $793
- **IQR:** USD $547 – $1237

Iron supplementation (all women of reproductive age)

- **Median Cost per YLD:** USD $2279
- **IQR:** USD $1605 – $3341

Antenatal preventative IPTp-SP

- **Median Cost per YLD:** USD $6333
- **IQR:** USD $4754 – $8354

Staple food supplementation

- **Median Cost per YLD:** USD $1009
- **IQR:** USD $478 – >USD 100k

Economic Considerations

Cost-effectiveness thresholds (CET, in USD per DALY averted) in **Djibouti** is as follows:

- **Estimates from Pichon-Riviere et al. (Lower Bound):** USD $252
- **Estimates from Pichon-Riviere et al. (Upper Bound):** USD $505
- **CET equal to per capita GDP:** USD $3606
- **CET equal to 2.3 (LMICs) or 1.7 (HICs) times per capita GDP:** USD $8295

⬆ Back to Table of Contents

Anaemia Reduction in Dominica

National Target

For our baseline CET = 1 x GDP per capita, the national target for anaemia in **Dominica** should be a reduction in prevalence of **24%**. This target is estimated with a 95% uncertainty interval from **11%** to **41%**.

For CET based on Pichon-Riviere, the national target for anaemia in **Dominica** should be a reduction in prevalence of **22%**. This target is estimated with a 95% uncertainty interval from **9%** to **39%**.

For a near-infinite CET, the national target for anaemia in **Dominica** should be a reduction in prevalence of **24%**. This target is estimated with a 95% uncertainty interval from **11%** to **41%**.

Input parameters

- Among women of reproductive age in **Dominica**, the prevalence of overall anaemia is **34.0%**
  - **Mild anaemia:** 18.5%
  - **Moderate anaemia:** 14.3%
  - **Severe anaemia:** 1.2%
- There is no data on malaria prevalence; assume 0%.

Intervention coverage and costs

Iron supplementation (antenatal care)

- **Current coverage:** 88.3%
- **Maximum feasible coverage:** 93.3%
- **Estimated unit cost:** USD $14.71 (7.26 - 23.34)

Iron supplementation (all women of reproductive age)

- **Current coverage:** 36.4%
- **Maximum feasible coverage:** 88.3%
- **Estimated unit cost:** USD $8.30 (4.18 - 15.44)

Antenatal preventative IPTp-SP

- **Current coverage:** 0.1%
- **Maximum feasible coverage:** 86.5%
- **Estimated unit cost:** USD $10.60 (5.36 - 16.75)

Staple food supplementation

- **Current coverage:** 27.5%
- **Maximum feasible coverage:** 100.0%
- **Estimated unit cost:** USD $0.44 (0.23 - 0.90)

Cost-Effectiveness Analysis

The estimated cost per Year of Life Disabled (YLD) averted for each intervention in **Dominica** is given below, with interquartile ranges (IQR):

Iron supplementation (antenatal care)

- **Median Cost per YLD:** USD $4012
- **IQR:** USD $2154 – >USD 100k

Iron supplementation (all women of reproductive age)

- **Median Cost per YLD:** USD $4032
- **IQR:** USD $2753 – $5893

Antenatal preventative IPTp-SP

- **Median Cost per YLD:** >USD 100k
- **IQR:** >USD 100k

Staple food supplementation

- **Median Cost per YLD:** USD $754
- **IQR:** USD $516 – $1110

Economic Considerations

Cost-effectiveness thresholds (CET, in USD per DALY averted) in **Dominica** is as follows:

- **Estimates from Pichon-Riviere et al. (Lower Bound):** USD $627
- **Estimates from Pichon-Riviere et al. (Upper Bound):** USD $5193
- **CET equal to per capita GDP:** USD $8954

- **CET equal to 2.3 (LMICs) or 1.7 (HICs) times per capita GDP:** USD $20594

⬆ Back to Table of Contents

Anaemia Reduction in Dominican Republic

National Target

For our baseline CET = 1 x GDP per capita, the national target for anaemia in **Dominican Republic** should be a reduction in prevalence of **14%**. This target is estimated with a 95% uncertainty interval from **7%** to **23%**.

For CET based on Pichon-Riviere, the national target for anaemia in **Dominican Republic** should be a reduction in prevalence of **13%**. This target is estimated with a 95% uncertainty interval from **5%** to **23%**.

For a near-infinite CET, the national target for anaemia in **Dominican Republic** should be a reduction in prevalence of **14%**. This target is estimated with a 95% uncertainty interval from **7%** to **22%**.

Input parameters

- Among women of reproductive age in **Dominican Republic**, the prevalence of overall anaemia is **33.6%**
  - **Mild anaemia:** 18.9%
  - **Moderate anaemia:** 13.6%
  - **Severe anaemia:** 1.0%
- The prevalence of malaria is **0.0%**.

Intervention coverage and costs

Iron supplementation (antenatal care)

- **Current coverage:** 94.7%
- **Maximum feasible coverage:** 99.5%
- **Estimated unit cost:** USD $9.81 (4.80 - 15.90)

Iron supplementation (all women of reproductive age)

- **Current coverage:** 39.0%
- **Maximum feasible coverage:** 99.5%
- **Estimated unit cost:** USD $5.50 (2.77 - 11.16)

Antenatal preventative IPTp-SP

- **Current coverage:** 0.1%
- **Maximum feasible coverage:** 99.5%
- **Estimated unit cost:** USD $6.87 (3.50 - 11.12)

Staple food supplementation

- **Current coverage:** 0.0%
- **Maximum feasible coverage:** 26.4%
- **Estimated unit cost:** USD $0.66 (0.15 - 1.87)

Cost-Effectiveness Analysis

The estimated cost per Year of Life Disabled (YLD) averted for each intervention in **Dominican Republic** is given below, with interquartile ranges (IQR):

Iron supplementation (antenatal care)

- **Median Cost per YLD:** USD $5201
- **IQR:** USD $1568 – >USD 100k

Iron supplementation (all women of reproductive age)

- **Median Cost per YLD:** USD $2976
- **IQR:** USD $2082 – $4298

Antenatal preventative IPTp-SP

- **Median Cost per YLD:** >USD 100k
- **IQR:** >USD 100k

Staple food supplementation

- **Median Cost per YLD:** USD $1274

- **IQR:** USD $838 – $1997

Economic Considerations

Cost-effectiveness thresholds (CET, in USD per DALY averted) in **Dominican Republic** is as follows:

- **Estimates from Pichon-Riviere et al. (Lower Bound):** USD $4501
- **Estimates from Pichon-Riviere et al. (Upper Bound):** USD $6108
- **CET equal to per capita GDP:** USD $10716
- **CET equal to 2.3 (LMICs) or 1.7 (HICs) times per capita GDP:** USD $24647

⬆ Back to Table of Contents

Anaemia Reduction in Ecuador

National Target

For our baseline CET = 1 x GDP per capita, the national target for anaemia in **Ecuador** should be a reduction in prevalence of **1%**. This target is estimated with a 95% uncertainty interval from **0%** to **39%**.

For CET based on Pichon-Riviere, the national target for anaemia in **Ecuador** should be a reduction in prevalence of **0%**. This target is estimated with a 95% uncertainty interval from **0%** to **37%**.

For a near-infinite CET, the national target for anaemia in **Ecuador** should be a reduction in prevalence of **26%**. This target is estimated with a 95% uncertainty interval from **14%** to **42%**.

Input parameters

- Among women of reproductive age in **Ecuador**, the prevalence of overall anaemia is **12.0%**
  - **Mild anaemia:** 8.3%
  - **Moderate anaemia:** 3.5%
  - **Severe anaemia:** 0.2%
- The prevalence of malaria is **0.0%**.

Intervention coverage and costs

Iron supplementation (antenatal care)

- **Current coverage:** 88.3%
- **Maximum feasible coverage:** 93.3%
- **Estimated unit cost:** USD $6.58 (3.19 - 11.23)

Iron supplementation (all women of reproductive age)

- **Current coverage:** 36.4%
- **Maximum feasible coverage:** 88.3%
- **Estimated unit cost:** USD $5.20 (2.62 - 10.84)

Antenatal preventative IPTp-SP

- **Current coverage:** 0.1%
- **Maximum feasible coverage:** 71.3%
- **Estimated unit cost:** USD $4.45 (2.29 - 7.65)

Staple food supplementation

- **Current coverage:** 20.7%
- **Maximum feasible coverage:** 100.0%
- **Estimated unit cost:** USD $1.18 (0.77 - 2.14)

Cost-Effectiveness Analysis

The estimated cost per Year of Life Disabled (YLD) averted for each intervention in **Ecuador** is given below, with interquartile ranges (IQR):

Iron supplementation (antenatal care)

- **Median Cost per YLD:** USD $7536
- **IQR:** USD $4250 – >USD 100k

Iron supplementation (all women of reproductive age)

- **Median Cost per YLD:** USD $11471
- **IQR:** USD $7986 – $16426

Antenatal preventative IPTp-SP

- **Median Cost per YLD:** >USD 100k
- **IQR:** >USD 100k

Staple food supplementation

- **Median Cost per YLD:** USD $7670
- **IQR:** USD $5542 – $11042

Economic Considerations

Cost-effectiveness thresholds (CET, in USD per DALY averted) in **Ecuador** is as follows:

- **Estimates from Pichon-Riviere et al. (Lower Bound):** USD $3659
- **Estimates from Pichon-Riviere et al. (Upper Bound):** USD $4965
- **CET equal to per capita GDP:** USD $6533
- **CET equal to 2.3 (LMICs) or 1.7 (HICs) times per capita GDP:** USD $15027

⬆ Back to Table of Contents

Anaemia Reduction in Egypt

National Target

For our baseline CET = 1 x GDP per capita, the national target for anaemia in **Egypt** should be a reduction in prevalence of **11%**. This target is estimated with a 95% uncertainty interval from **1%** to **28%**.

For CET based on Pichon-Riviere, the national target for anaemia in **Egypt** should be a reduction in prevalence of **5%**. This target is estimated with a 95% uncertainty interval from **0%** to **19%**.

For a near-infinite CET, the national target for anaemia in **Egypt** should be a reduction in prevalence of **16%**. This target is estimated with a 95% uncertainty interval from **8%** to **27%**.

Input parameters

- Among women of reproductive age in **Egypt**, the prevalence of overall anaemia is **26.0%**
  - **Mild anaemia:** 17.8%

- **Moderate anaemia:** 7.9%
- **Severe anaemia:** 0.3%

- There is no data on malaria prevalence; assume 0%.

Intervention coverage and costs

Iron supplementation (antenatal care)

- **Current coverage:** NaN%
- **Maximum feasible coverage:** NaN%
- **Estimated unit cost:** USD $4.91 (2.33 - 8.44)

Iron supplementation (all women of reproductive age)

- **Current coverage:** NaN%
- **Maximum feasible coverage:** NaN%
- **Estimated unit cost:** USD $3.29 (1.63 - 7.39)

Antenatal preventative IPTp-SP

- **Current coverage:** NaN%
- **Maximum feasible coverage:** NaN%
- **Estimated unit cost:** USD $3.21 (1.66 - 5.52)

Staple food supplementation

- **Current coverage:** NaN%
- **Maximum feasible coverage:** NaN%
- **Estimated unit cost:** USD $0.21 (0.05 - 0.72)

Cost-Effectiveness Analysis

The estimated cost per Year of Life Disabled (YLD) averted for each intervention in **Egypt** is given below, with interquartile ranges (IQR):

Iron supplementation (antenatal care)

- **Median Cost per YLD:** USD $1825

- **IQR:** USD $1312 – $2532

Iron supplementation (all women of reproductive age)

- **Median Cost per YLD:** USD $3550
- **IQR:** USD $2471 – $5070

Antenatal preventative IPTp-SP

- **Median Cost per YLD:** >USD 100k
- **IQR:** >USD 100k

Staple food supplementation

- **Median Cost per YLD:** USD $785
- **IQR:** USD $483 – $1313

Economic Considerations

Cost-effectiveness thresholds (CET, in USD per DALY averted) in **Egypt** is as follows:

- **Estimates from Pichon-Riviere et al. (Lower Bound):** USD $703
- **Estimates from Pichon-Riviere et al. (Upper Bound):** USD $1370
- **CET equal to per capita GDP:** USD $3513
- **CET equal to 2.3 (LMICs) or 1.7 (HICs) times per capita GDP:** USD $8079

⬆ Back to Table of Contents

Anaemia Reduction in El Salvador

National Target

For our baseline CET = 1 x GDP per capita, the national target for anaemia in **El Salvador** should be a reduction in prevalence of **21%**. This target is estimated with a 95% uncertainty interval from **0%** to **41%**.

For CET based on Pichon-Riviere, the national target for anaemia in **El Salvador** should be a reduction in prevalence of **1%**. This target is estimated with a 95% uncertainty interval from **0%** to **40%**.

For a near-infinite CET, the national target for anaemia in **El Salvador** should be a reduction in prevalence of **27%**. This target is estimated with a 95% uncertainty interval from **14%** to **44%**.

Input parameters

- Among women of reproductive age in **El Salvador**, the prevalence of overall anaemia is **9.3%**
  - **Mild anaemia:** 6.5%
  - **Moderate anaemia:** 2.6%
  - **Severe anaemia:** 0.1%
- The prevalence of malaria is **0.0%**.

Intervention coverage and costs

Iron supplementation (antenatal care)

- **Current coverage:** 74.1%
- **Maximum feasible coverage:** 86.9%
- **Estimated unit cost:** USD $2.57 (1.16 - 4.94)

Iron supplementation (all women of reproductive age)

- **Current coverage:** 30.5%
- **Maximum feasible coverage:** 86.9%
- **Estimated unit cost:** USD $3.51 (1.77 - 8.01)

Antenatal preventative IPTp-SP

- **Current coverage:** 0.0%
- **Maximum feasible coverage:** 86.9%
- **Estimated unit cost:** USD $1.46 (0.80 - 2.99)

Staple food supplementation

- **Current coverage:** 10.9%
- **Maximum feasible coverage:** 100.0%
- **Estimated unit cost:** USD $0.33 (0.10 - 1.19)

Cost-Effectiveness Analysis

The estimated cost per Year of Life Disabled (YLD) averted for each intervention in **El Salvador** is given below, with interquartile ranges (IQR):

Iron supplementation (antenatal care)

- **Median Cost per YLD:** USD $4218
- **IQR:** USD $2347 – >USD 100k

Iron supplementation (all women of reproductive age)

- **Median Cost per YLD:** USD $10423
- **IQR:** USD $7064 – $15425

Antenatal preventative IPTp-SP

- **Median Cost per YLD:** >USD 100k
- **IQR:** >USD 100k

Staple food supplementation

- **Median Cost per YLD:** USD $3469
- **IQR:** USD $2224 – $5591

Economic Considerations

Cost-effectiveness thresholds (CET, in USD per DALY averted) in **El Salvador** is as follows:

- **Estimates from Pichon-Riviere et al. (Lower Bound):** USD $1550
- **Estimates from Pichon-Riviere et al. (Upper Bound):** USD $3046
- **CET equal to per capita GDP:** USD $5344
- **CET equal to 2.3 (LMICs) or 1.7 (HICs) times per capita GDP:** USD $12292

⬆ Back to Table of Contents

Anaemia Reduction in Equatorial Guinea

National Target

For our baseline CET = 1 x GDP per capita, the national target for anaemia in **Equatorial Guinea** should be a reduction in prevalence of **22%**. This target is estimated with a 95% uncertainty interval from **9%** to **38%**.

For CET based on Pichon-Riviere, the national target for anaemia in **Equatorial Guinea** should be a reduction in prevalence of **13%**. This target is estimated with a 95% uncertainty interval from **0%** to **32%**.

For a near-infinite CET, the national target for anaemia in **Equatorial Guinea** should be a reduction in prevalence of **23%**. This target is estimated with a 95% uncertainty interval from **12%** to **37%**.

Input parameters

- Among women of reproductive age in **Equatorial Guinea**, the prevalence of overall anaemia is **47.4%**
  - **Mild anaemia:** 27.8%
  - **Moderate anaemia:** 18.5%
  - **Severe anaemia:** 1.1%
- The prevalence of malaria is **24.1%**.

Intervention coverage and costs

Iron supplementation (antenatal care)

- **Current coverage:** 90.2%
- **Maximum feasible coverage:** 94.6%
- **Estimated unit cost:** USD $13.92 (6.77 - 21.64)

Iron supplementation (all women of reproductive age)

- **Current coverage:** 37.2%
- **Maximum feasible coverage:** 94.6%
- **Estimated unit cost:** USD $9.09 (4.50 - 15.86)

Antenatal preventative IPTp-SP

- **Current coverage:** 27.8%
- **Maximum feasible coverage:** 94.6%
- **Estimated unit cost:** USD $9.96 (5.01 - 15.46)

Staple food supplementation

- **Current coverage:** 40.8%
- **Maximum feasible coverage:** 100.0%
- **Estimated unit cost:** USD $0.76 (0.11 - 2.33)

Cost-Effectiveness Analysis

The estimated cost per Year of Life Disabled (YLD) averted for each intervention in **Equatorial Guinea** is given below, with interquartile ranges (IQR):

Iron supplementation (antenatal care)

- **Median Cost per YLD:** USD $2869
- **IQR:** USD $1528 – >USD 100k

Iron supplementation (all women of reproductive age)

- **Median Cost per YLD:** USD $3310
- **IQR:** USD $2319 – $4796

Antenatal preventative IPTp-SP

- **Median Cost per YLD:** USD $9230
- **IQR:** USD $7025 – $11886

Staple food supplementation

- **Median Cost per YLD:** USD $1133
- **IQR:** USD $704 – $1716

Economic Considerations

Cost-effectiveness thresholds (CET, in USD per DALY averted) in **Equatorial Guinea** is as follows:

- **Estimates from Pichon-Riviere et al. (Lower Bound):** USD $1131
- **Estimates from Pichon-Riviere et al. (Upper Bound):** USD $1555
- **CET equal to per capita GDP:** USD $7067
- **CET equal to 2.3 (LMICs) or 1.7 (HICs) times per capita GDP:** USD $16253

⬆ Back to Table of Contents

Anaemia Reduction in Estonia

National Target

For our baseline CET = 1 x GDP per capita, the national target for anaemia in **Estonia** should be a reduction in prevalence of **24%**. This target is estimated with a 95% uncertainty interval from **9%** to **42%**.

For CET based on Pichon-Riviere, the national target for anaemia in **Estonia** should be a reduction in prevalence of **20%**. This target is estimated with a 95% uncertainty interval from **7%** to **39%**.

For a near-infinite CET, the national target for anaemia in **Estonia** should be a reduction in prevalence of **26%**. This target is estimated with a 95% uncertainty interval from **14%** to **43%**.

Input parameters

- Among women of reproductive age in **Estonia**, the prevalence of overall anaemia is **18.9%**
  - **Mild anaemia:** 11.7%
  - **Moderate anaemia:** 6.7%
  - **Severe anaemia:** 0.5%
- There is no data on malaria prevalence; assume 0%.

Intervention coverage and costs

Iron supplementation (antenatal care)

- **Current coverage:** 81.0%
- **Maximum feasible coverage:** 100.0%

- **Estimated unit cost:** USD $42.94 (10.54 - 69.47)

Iron supplementation (all women of reproductive age)

- **Current coverage:** 33.4%
- **Maximum feasible coverage:** 100.0%
- **Estimated unit cost:** USD $24.29 (6.62 - 49.39)

Antenatal preventative IPTp-SP

- **Current coverage:** 0.0%
- **Maximum feasible coverage:** 100.0%
- **Estimated unit cost:** USD $2.65 (1.39 - 4.79)

Staple food supplementation

- **Current coverage:** 27.9%
- **Maximum feasible coverage:** 100.0%
- **Estimated unit cost:** USD $0.81 (0.58 - 1.29)

Cost-Effectiveness Analysis

The estimated cost per Year of Life Disabled (YLD) averted for each intervention in **Estonia** is given below, with interquartile ranges (IQR):

Iron supplementation (antenatal care)

- **Median Cost per YLD:** USD $15770
- **IQR:** USD $10761 – $24169

Iron supplementation (all women of reproductive age)

- **Median Cost per YLD:** USD $24527
- **IQR:** USD $16793 – $36548

Antenatal preventative IPTp-SP

- **Median Cost per YLD:** >USD 100k
- **IQR:** >USD 100k

Staple food supplementation

- **Median Cost per YLD:** USD $3165
- **IQR:** USD $2227 – $4490

Economic Considerations

Cost-effectiveness thresholds (CET, in USD per DALY averted) in **Estonia** is as follows:

- **Estimates from Pichon-Riviere et al. (Lower Bound):** USD $14614
- **Estimates from Pichon-Riviere et al. (Upper Bound):** USD $17000
- **CET equal to per capita GDP:** USD $29824
- **CET equal to 2.3 (LMICs) or 1.7 (HICs) times per capita GDP:** USD $41753

⬆ Back to Table of Contents

Anaemia Reduction in Eswatini

National Target

For our baseline CET = 1 x GDP per capita, the national target for anaemia in **Eswatini** should be a reduction in prevalence of **23%**. This target is estimated with a 95% uncertainty interval from **9%** to **38%**.

For CET based on Pichon-Riviere, the national target for anaemia in **Eswatini** should be a reduction in prevalence of **18%**. This target is estimated with a 95% uncertainty interval from **0%** to **35%**.

For a near-infinite CET, the national target for anaemia in **Eswatini** should be a reduction in prevalence of **24%**. This target is estimated with a 95% uncertainty interval from **13%** to **38%**.

Input parameters

- Among women of reproductive age in **Eswatini**, the prevalence of overall anaemia is **28.7%**
    - **Mild anaemia:** 15.8%
    - **Moderate anaemia:** 11.9%
    - **Severe anaemia:** 1.0%
- There is no data on malaria prevalence; assume 0%.

Intervention coverage and costs

Iron supplementation (antenatal care)

- **Current coverage:** 84.8%
- **Maximum feasible coverage:** 97.1%
- **Estimated unit cost:** USD $4.31 (1.99 - 7.40)

Iron supplementation (all women of reproductive age)

- **Current coverage:** 35.0%
- **Maximum feasible coverage:** 97.1%
- **Estimated unit cost:** USD $3.60 (1.78 - 7.48)

Antenatal preventative IPTp-SP

- **Current coverage:** 0.0%
- **Maximum feasible coverage:** 97.1%
- **Estimated unit cost:** USD $2.71 (1.38 - 4.66)

Staple food supplementation

- **Current coverage:** 6.1%
- **Maximum feasible coverage:** 69.7%
- **Estimated unit cost:** USD $0.37 (0.10 - 1.29)

Cost-Effectiveness Analysis

The estimated cost per Year of Life Disabled (YLD) averted for each intervention in **Eswatini** is given below, with interquartile ranges (IQR):

Iron supplementation (antenatal care)

- **Median Cost per YLD:** USD $1109
- **IQR:** USD $721 – >USD 100k

Iron supplementation (all women of reproductive age)

- **Median Cost per YLD:** USD $2199
- **IQR:** USD $1559 – $3129

Antenatal preventative IPTp-SP

- **Median Cost per YLD:** >USD 100k
- **IQR:** >USD 100k

Staple food supplementation

- **Median Cost per YLD:** USD $896
- **IQR:** USD $562 – $1406

Economic Considerations

Cost-effectiveness thresholds (CET, in USD per DALY averted) in **Eswatini** is as follows:

- **Estimates from Pichon-Riviere et al. (Lower Bound):** USD $911
- **Estimates from Pichon-Riviere et al. (Upper Bound):** USD $1785
- **CET equal to per capita GDP:** USD $3797
- **CET equal to 2.3 (LMICs) or 1.7 (HICs) times per capita GDP:** USD $8734

⬆ Back to Table of Contents

Anaemia Reduction in Ethiopia

National Target

For our baseline CET = 1 x GDP per capita, the national target for anaemia in **Ethiopia** should be a reduction in prevalence of **5%**. This target is estimated with a 95% uncertainty interval from **0%** to **18%**.

For CET based on Pichon-Riviere, the national target for anaemia in **Ethiopia** should be a reduction in prevalence of **0%**. This target is estimated with a 95% uncertainty interval from **0%** to **0%**.

For a near-infinite CET, the national target for anaemia in **Ethiopia** should be a reduction in prevalence of **12%**. This target is estimated with a 95% uncertainty interval from **7%** to **21%**.

Input parameters

- Among women of reproductive age in **Ethiopia**, the prevalence of overall anaemia is **23.1%**
    - **Mild anaemia:** 12.7%
    - **Moderate anaemia:** 9.4%
    - **Severe anaemia:** 1.0%
- The prevalence of malaria is **1.9%**.

Intervention coverage and costs

Iron supplementation (antenatal care)

- **Current coverage:** 59.3%
- **Maximum feasible coverage:** 73.2%
- **Estimated unit cost:** USD $2.62 (1.09 - 4.62)

Iron supplementation (all women of reproductive age)

- **Current coverage:** 24.4%
- **Maximum feasible coverage:** 73.2%
- **Estimated unit cost:** USD $2.55 (1.25 - 6.08)

Antenatal preventative IPTp-SP

- **Current coverage:** 0.3%
- **Maximum feasible coverage:** 73.2%
- **Estimated unit cost:** USD $1.39 (0.72 - 2.65)

Staple food supplementation

- **Current coverage:** 1.3%
- **Maximum feasible coverage:** 18.6%
- **Estimated unit cost:** USD $0.25 (0.08 - 0.93)

Cost-Effectiveness Analysis

The estimated cost per Year of Life Disabled (YLD) averted for each intervention in **Ethiopia** is given below, with interquartile ranges (IQR):

Iron supplementation (antenatal care)

- **Median Cost per YLD:** USD $863
- **IQR:** USD $637 – $1214

Iron supplementation (all women of reproductive age)

- **Median Cost per YLD:** USD $2297
- **IQR:** USD $1629 – $3322

Antenatal preventative IPTp-SP

- **Median Cost per YLD:** USD $16071
- **IQR:** USD $12437 – $20373

Staple food supplementation

- **Median Cost per YLD:** USD $979
- **IQR:** USD $635 – $1499

Economic Considerations

Cost-effectiveness thresholds (CET, in USD per DALY averted) in **Ethiopia** is as follows:

- **Estimates from Pichon-Riviere et al. (Lower Bound):** USD $65
- **Estimates from Pichon-Riviere et al. (Upper Bound):** USD $181
- **CET equal to per capita GDP:** USD $1294
- **CET equal to 2.3 (LMICs) or 1.7 (HICs) times per capita GDP:** USD $2976

⬆ Back to Table of Contents

Anaemia Reduction in Fiji

National Target

For our baseline CET = 1 x GDP per capita, the national target for anaemia in **Fiji** should be a reduction in prevalence of **21%**. This target is estimated with a 95% uncertainty interval from **9%** to **36%**.

For CET based on Pichon-Riviere, the national target for anaemia in **Fiji** should be a reduction in prevalence of **15%**. This target is estimated with a 95% uncertainty interval from **0%** to **33%**.

For a near-infinite CET, the national target for anaemia in **Fiji** should be a reduction in prevalence of **21%**. This target is estimated with a 95% uncertainty interval from **10%** to **36%**.

Input parameters

- Among women of reproductive age in **Fiji**, the prevalence of overall anaemia is **37.5%**
  - **Mild anaemia:** 21.8%
  - **Moderate anaemia:** 14.7%
  - **Severe anaemia:** 0.9%
- There is no data on malaria prevalence; assume 0%.

Intervention coverage and costs

Iron supplementation (antenatal care)

- **Current coverage:** 84.2%
- **Maximum feasible coverage:** 89.0%
- **Estimated unit cost:** USD $7.17 (3.48 - 12.65)

Iron supplementation (all women of reproductive age)

- **Current coverage:** 34.7%
- **Maximum feasible coverage:** 89.0%
- **Estimated unit cost:** USD $4.89 (2.47 - 10.88)

Antenatal preventative IPTp-SP

- **Current coverage:** 0.0%
- **Maximum feasible coverage:** 89.0%
- **Estimated unit cost:** USD $5.14 (2.64 - 9.31)

Staple food supplementation

- **Current coverage:** 45.4%
- **Maximum feasible coverage:** 99.0%
- **Estimated unit cost:** USD $0.40 (0.08 - 1.12)

Cost-Effectiveness Analysis

The estimated cost per Year of Life Disabled (YLD) averted for each intervention in **Fiji** is given below, with interquartile ranges (IQR):

Iron supplementation (antenatal care)

- **Median Cost per YLD:** USD $1835
- **IQR:** USD $1142 – >USD 100k

Iron supplementation (all women of reproductive age)

- **Median Cost per YLD:** USD $2659
- **IQR:** USD $1829 – $3810

Antenatal preventative IPTp-SP

- **Median Cost per YLD:** >USD 100k
- **IQR:** >USD 100k

Staple food supplementation

- **Median Cost per YLD:** USD $698
- **IQR:** USD $441 – $1107

Economic Considerations

Cost-effectiveness thresholds (CET, in USD per DALY averted) in **Fiji** is as follows:

- **Estimates from Pichon-Riviere et al. (Lower Bound):** USD $1408
- **Estimates from Pichon-Riviere et al. (Upper Bound):** USD $1936
- **CET equal to per capita GDP:** USD $5868
- **CET equal to 2.3 (LMICs) or 1.7 (HICs) times per capita GDP:** USD $13497

⬆ Back to Table of Contents

Anaemia Reduction in Finland

National Target

For our baseline CET = 1 x GDP per capita, the national target for anaemia in **Finland** should be a reduction in prevalence of **16%**. This target is estimated with a 95% uncertainty interval from **6%** to **33%**.

For CET based on Pichon-Riviere, the national target for anaemia in **Finland** should be a reduction in prevalence of **16%**. This target is estimated with a 95% uncertainty interval from **6%** to **33%**.

For a near-infinite CET, the national target for anaemia in **Finland** should be a reduction in prevalence of **25%**. This target is estimated with a 95% uncertainty interval from **13%** to **40%**.

Input parameters

- Among women of reproductive age in **Finland**, the prevalence of overall anaemia is **7.3%**
  - **Mild anaemia:** 5.8%
  - **Moderate anaemia:** 1.5%
  - **Severe anaemia:** 0.0%
- There is no data on malaria prevalence; assume 0%.

Intervention coverage and costs

Iron supplementation (antenatal care)

- **Current coverage:** 81.0%
- **Maximum feasible coverage:** 100.0%
- **Estimated unit cost:** USD $59.68 (18.87 - 97.05)

Iron supplementation (all women of reproductive age)

- **Current coverage:** 33.4%
- **Maximum feasible coverage:** 100.0%
- **Estimated unit cost:** USD $38.23 (11.01 - 57.91)

Antenatal preventative IPTp-SP

- **Current coverage:** 0.0%
- **Maximum feasible coverage:** 100.0%
- **Estimated unit cost:** USD $16.27 (8.20 - 27.23)

Staple food supplementation

- **Current coverage:** 37.0%
- **Maximum feasible coverage:** 100.0%
- **Estimated unit cost:** USD $0.13 (0.01 - 0.32)

Cost-Effectiveness Analysis

The estimated cost per Year of Life Disabled (YLD) averted for each intervention in **Finland** is given below, with interquartile ranges (IQR):

Iron supplementation (antenatal care)

- **Median Cost per YLD:** >USD 100k
- **IQR:** USD $67243 – >USD 100k

Iron supplementation (all women of reproductive age)

- **Median Cost per YLD:** >USD 100k
- **IQR:** USD $97578 – >USD 100k

Antenatal preventative IPTp-SP

- **Median Cost per YLD:** >USD 100k
- **IQR:** >USD 100k

Staple food supplementation

- **Median Cost per YLD:** USD $2393
- **IQR:** USD $1516 – $3744

Economic Considerations

Cost-effectiveness thresholds (CET, in USD per DALY averted) in **Finland** is as follows:

- **Estimates from Pichon-Riviere et al. (Lower Bound):** USD $43542
- **Estimates from Pichon-Riviere et al. (Upper Bound):** USD $43542
- **CET equal to per capita GDP:** USD $53756
- **CET equal to 2.3 (LMICs) or 1.7 (HICs) times per capita GDP:** USD $75258

⬆ Back to Table of Contents

Anaemia Reduction in France

National Target

For our baseline CET = 1 x GDP per capita, the national target for anaemia in **France** should be a reduction in prevalence of **17%**. This target is estimated with a 95% uncertainty interval from **6%** to **35%**.

For CET based on Pichon-Riviere, the national target for anaemia in **France** should be a reduction in prevalence of **17%**. This target is estimated with a 95% uncertainty interval from **6%** to **34%**.

For a near-infinite CET, the national target for anaemia in **France** should be a reduction in prevalence of **25%**. This target is estimated with a 95% uncertainty interval from **13%** to **40%**.

Input parameters

- Among women of reproductive age in **France**, the prevalence of overall anaemia is **6.7%**
    - **Mild anaemia:** 4.9%
    - **Moderate anaemia:** 1.8%
    - **Severe anaemia:** 0.1%
- There is no data on malaria prevalence; assume 0%.

Intervention coverage and costs

Iron supplementation (antenatal care)

- **Current coverage:** 81.0%
- **Maximum feasible coverage:** 100.0%

- **Estimated unit cost:** USD $58.64 (18.43 - 96.45)

Iron supplementation (all women of reproductive age)

- **Current coverage:** 33.4%
- **Maximum feasible coverage:** 100.0%
- **Estimated unit cost:** USD $35.90 (10.07 - 51.05)

Antenatal preventative IPTp-SP

- **Current coverage:** 0.0%
- **Maximum feasible coverage:** 100.0%
- **Estimated unit cost:** USD $17.22 (8.68 - 28.65)

Staple food supplementation

- **Current coverage:** 34.8%
- **Maximum feasible coverage:** 100.0%
- **Estimated unit cost:** USD $0.33 (0.20 - 0.54)

Cost-Effectiveness Analysis

The estimated cost per Year of Life Disabled (YLD) averted for each intervention in **France** is given below, with interquartile ranges (IQR):

Iron supplementation (antenatal care)

- **Median Cost per YLD:** USD $84084
- **IQR:** USD $57870 – >USD 100k

Iron supplementation (all women of reproductive age)

- **Median Cost per YLD:** >USD 100k
- **IQR:** USD $83965 – >USD 100k

Antenatal preventative IPTp-SP

- **Median Cost per YLD:** >USD 100k
- **IQR:** >USD 100k

Staple food supplementation

- **Median Cost per YLD:** USD $4679
- **IQR:** USD $3286 – $6696

Economic Considerations

Cost-effectiveness thresholds (CET, in USD per DALY averted) in **France** is as follows:

- **Estimates from Pichon-Riviere et al. (Lower Bound):** USD $37347
- **Estimates from Pichon-Riviere et al. (Upper Bound):** USD $44016
- **CET equal to per capita GDP:** USD $44461
- **CET equal to 2.3 (LMICs) or 1.7 (HICs) times per capita GDP:** USD $62245

⬆ Back to Table of Contents

Anaemia Reduction in Gabon

National Target

For our baseline CET = 1 x GDP per capita, the national target for anaemia in **Gabon** should be a reduction in prevalence of **25%**. This target is estimated with a 95% uncertainty interval from **12%** to **42%**.

For CET based on Pichon-Riviere, the national target for anaemia in **Gabon** should be a reduction in prevalence of **22%**. This target is estimated with a 95% uncertainty interval from **8%** to **41%**.

For a near-infinite CET, the national target for anaemia in **Gabon** should be a reduction in prevalence of **25%**. This target is estimated with a 95% uncertainty interval from **13%** to **41%**.

Input parameters

- Among women of reproductive age in **Gabon**, the prevalence of overall anaemia is **57.0%**
  - **Mild anaemia:** 25.8%
  - **Moderate anaemia:** 28.5%
  - **Severe anaemia:** 2.7%
- The prevalence of malaria is **21.2%**.

Intervention coverage and costs

Iron supplementation (antenatal care)

- **Current coverage:** 92.5%
- **Maximum feasible coverage:** 96.8%
- **Estimated unit cost:** USD $10.39 (5.01 - 16.37)

Iron supplementation (all women of reproductive age)

- **Current coverage:** 38.1%
- **Maximum feasible coverage:** 96.8%
- **Estimated unit cost:** USD $6.34 (3.12 - 11.74)

Antenatal preventative IPTp-SP

- **Current coverage:** 50.7%
- **Maximum feasible coverage:** 96.8%
- **Estimated unit cost:** USD $7.32 (3.69 - 11.55)

Staple food supplementation

- **Current coverage:** 28.7%
- **Maximum feasible coverage:** 99.2%
- **Estimated unit cost:** USD $0.71 (0.36 - 1.49)

Cost-Effectiveness Analysis

The estimated cost per Year of Life Disabled (YLD) averted for each intervention in **Gabon** is given below, with interquartile ranges (IQR):

Iron supplementation (antenatal care)

- **Median Cost per YLD:** USD $1613
- **IQR:** USD $841 – >USD 100k

Iron supplementation (all women of reproductive age)

- **Median Cost per YLD:** USD $1570
- **IQR:** USD $1121 – $2267

Antenatal preventative IPTp-SP

- **Median Cost per YLD:** USD $4774
- **IQR:** USD $3628 – $6215

Staple food supplementation

- **Median Cost per YLD:** USD $519
- **IQR:** USD $369 – $813

Economic Considerations

Cost-effectiveness thresholds (CET, in USD per DALY averted) in **Gabon** is as follows:

- **Estimates from Pichon-Riviere et al. (Lower Bound):** USD $1431
- **Estimates from Pichon-Riviere et al. (Upper Bound):** USD $2021
- **CET equal to per capita GDP:** USD $8420
- **CET equal to 2.3 (LMICs) or 1.7 (HICs) times per capita GDP:** USD $19366

⬆ Back to Table of Contents

Anaemia Reduction in Gambia

National Target

For our baseline CET = 1 x GDP per capita, the national target for anaemia in **Gambia** should be a reduction in prevalence of **22%**. This target is estimated with a 95% uncertainty interval from **0%** to **43%**.

For CET based on Pichon-Riviere, the national target for anaemia in **Gambia** should be a reduction in prevalence of **0%**. This target is estimated with a 95% uncertainty interval from **0%** to **2%**.

For a near-infinite CET, the national target for anaemia in **Gambia** should be a reduction in prevalence of **28%**. This target is estimated with a 95% uncertainty interval from **15%** to **45%**.

Input parameters

- Among women of reproductive age in **Gambia**, the prevalence of overall anaemia is **57.0%**
  - **Mild anaemia:** 25.9%
  - **Moderate anaemia:** 28.4%
  - **Severe anaemia:** 2.7%
- The prevalence of malaria is **3.7%**.

Intervention coverage and costs

Iron supplementation (antenatal care)

- **Current coverage:** NaN%
- **Maximum feasible coverage:** NaN%
- **Estimated unit cost:** USD $2.82 (1.26 - 5.28)

Iron supplementation (all women of reproductive age)

- **Current coverage:** NaN%
- **Maximum feasible coverage:** NaN%
- **Estimated unit cost:** USD $2.67 (1.76 - 9.52)

Antenatal preventative IPTp-SP

- **Current coverage:** NaN%
- **Maximum feasible coverage:** NaN%
- **Estimated unit cost:** USD $1.46 (0.76 - 2.77)

Staple food supplementation

- **Current coverage:** NaN%
- **Maximum feasible coverage:** NaN%
- **Estimated unit cost:** USD $0.61 (0.09 - 1.88)

Cost-Effectiveness Analysis

The estimated cost per Year of Life Disabled (YLD) averted for each intervention in **Gambia** is given below, with interquartile ranges (IQR):

Iron supplementation (antenatal care)

- **Median Cost per YLD:** >USD 100k
- **IQR:** USD $272 – >USD 100k

Iron supplementation (all women of reproductive age)

- **Median Cost per YLD:** USD $1023
- **IQR:** USD $699 – $1512

Antenatal preventative IPTp-SP

- **Median Cost per YLD:** USD $2388
- **IQR:** USD $1829 – $3258

Staple food supplementation

- **Median Cost per YLD:** USD $597
- **IQR:** USD $391 – $902

Economic Considerations

Cost-effectiveness thresholds (CET, in USD per DALY averted) in **Gambia** is as follows:

- **Estimates from Pichon-Riviere et al. (Lower Bound):** USD $42
- **Estimates from Pichon-Riviere et al. (Upper Bound):** USD $135
- **CET equal to per capita GDP:** USD $844
- **CET equal to 2.3 (LMICs) or 1.7 (HICs) times per capita GDP:** USD $1941

⬆ Back to Table of Contents

Anaemia Reduction in Georgia

National Target

For our baseline CET = 1 x GDP per capita, the national target for anaemia in **Georgia** should be a reduction in prevalence of **19%**. This target is estimated with a 95% uncertainty interval from **6%** to **34%**.

For CET based on Pichon-Riviere, the national target for anaemia in **Georgia** should be a reduction in prevalence of **14%**. This target is estimated with a 95% uncertainty interval from **3%** to **32%**.

For a near-infinite CET, the national target for anaemia in **Georgia** should be a reduction in prevalence of **20%**. This target is estimated with a 95% uncertainty interval from **11%** to **35%**.

Input parameters

- Among women of reproductive age in **Georgia**, the prevalence of overall anaemia is **25.2%**
  - **Mild anaemia:** 14.0%
  - **Moderate anaemia:** 10.3%
  - **Severe anaemia:** 0.9%
- The prevalence of malaria is **0.0%**.

Intervention coverage and costs

Iron supplementation (antenatal care)

- **Current coverage:** 54.0%
- **Maximum feasible coverage:** 85.0%
- **Estimated unit cost:** USD $8.15 (4.07 - 14.86)

Iron supplementation (all women of reproductive age)

- **Current coverage:** 22.2%
- **Maximum feasible coverage:** 85.0%
- **Estimated unit cost:** USD $6.48 (3.75 - 16.94)

Antenatal preventative IPTp-SP

- **Current coverage:** 0.0%
- **Maximum feasible coverage:** 85.0%
- **Estimated unit cost:** USD $6.21 (3.19 - 11.21)

Staple food supplementation

- **Current coverage:** 60.4%
- **Maximum feasible coverage:** 100.0%
- **Estimated unit cost:** USD $0.47 (0.34 - 0.68)

Cost-Effectiveness Analysis

The estimated cost per Year of Life Disabled (YLD) averted for each intervention in **Georgia** is given below, with interquartile ranges (IQR):

Iron supplementation (antenatal care)

- **Median Cost per YLD:** USD $2281
- **IQR:** USD $1684 – $3138

Iron supplementation (all women of reproductive age)

- **Median Cost per YLD:** USD $5566
- **IQR:** USD $3848 – $8202

Antenatal preventative IPTp-SP

- **Median Cost per YLD:** >USD 100k
- **IQR:** >USD 100k

Staple food supplementation

- **Median Cost per YLD:** USD $1008
- **IQR:** USD $711 – $1481

Economic Considerations

Cost-effectiveness thresholds (CET, in USD per DALY averted) in **Georgia** is as follows:

- **Estimates from Pichon-Riviere et al. (Lower Bound):** USD $3492
- **Estimates from Pichon-Riviere et al. (Upper Bound):** USD $4791
- **CET equal to per capita GDP:** USD $8120

- **CET equal to 2.3 (LMICs) or 1.7 (HICs) times per capita GDP:** USD $18677

⬆ Back to Table of Contents

Anaemia Reduction in Germany

National Target

For our baseline CET = 1 x GDP per capita, the national target for anaemia in **Germany** should be a reduction in prevalence of **18%**. This target is estimated with a 95% uncertainty interval from **6%** to **36%**.

For CET based on Pichon-Riviere, the national target for anaemia in **Germany** should be a reduction in prevalence of **18%**. This target is estimated with a 95% uncertainty interval from **7%** to **36%**.

For a near-infinite CET, the national target for anaemia in **Germany** should be a reduction in prevalence of **25%**. This target is estimated with a 95% uncertainty interval from **13%** to **41%**.

Input parameters

- Among women of reproductive age in **Germany**, the prevalence of overall anaemia is **12.2%**
  - **Mild anaemia:** 11.2%
  - **Moderate anaemia:** 1.0%
  - **Severe anaemia:** 0.0%
- There is no data on malaria prevalence; assume 0%.

Intervention coverage and costs

Iron supplementation (antenatal care)

- **Current coverage:** 81.0%
- **Maximum feasible coverage:** 100.0%
- **Estimated unit cost:** USD $61.28 (19.54 - 100.88)

Iron supplementation (all women of reproductive age)

- **Current coverage:** 33.4%
- **Maximum feasible coverage:** 100.0%

- **Estimated unit cost:** USD $37.28 (10.63 - 55.13)

Antenatal preventative IPTp-SP

- **Current coverage:** 0.0%
- **Maximum feasible coverage:** 100.0%
- **Estimated unit cost:** USD $17.26 (8.70 - 28.71)

Staple food supplementation

- **Current coverage:** 34.9%
- **Maximum feasible coverage:** 100.0%
- **Estimated unit cost:** USD $0.34 (0.24 - 0.51)

Cost-Effectiveness Analysis

The estimated cost per Year of Life Disabled (YLD) averted for each intervention in **Germany** is given below, with interquartile ranges (IQR):

Iron supplementation (antenatal care)

- **Median Cost per YLD:** USD $63206
- **IQR:** USD $39686 – >USD 100k

Iron supplementation (all women of reproductive age)

- **Median Cost per YLD:** USD $90240
- **IQR:** USD $57761 – >USD 100k

Antenatal preventative IPTp-SP

- **Median Cost per YLD:** >USD 100k
- **IQR:** >USD 100k

Staple food supplementation

- **Median Cost per YLD:** USD $3688
- **IQR:** USD $2383 – $6325

Economic Considerations

Cost-effectiveness thresholds (CET, in USD per DALY averted) in **Germany** is as follows:

- **Estimates from Pichon-Riviere et al. (Lower Bound):** USD $45361
- **Estimates from Pichon-Riviere et al. (Upper Bound):** USD $53273
- **CET equal to per capita GDP:** USD $52746
- **CET equal to 2.3 (LMICs) or 1.7 (HICs) times per capita GDP:** USD $73844

⬆ Back to Table of Contents

Anaemia Reduction in Ghana

National Target

For our baseline CET = 1 x GDP per capita, the national target for anaemia in **Ghana** should be a reduction in prevalence of **21%**. This target is estimated with a 95% uncertainty interval from **9%** to **34%**.

For CET based on Pichon-Riviere, the national target for anaemia in **Ghana** should be a reduction in prevalence of **8%**. This target is estimated with a 95% uncertainty interval from **0%** to **29%**.

For a near-infinite CET, the national target for anaemia in **Ghana** should be a reduction in prevalence of **21%**. This target is estimated with a 95% uncertainty interval from **11%** to **33%**.

Input parameters

- Among women of reproductive age in **Ghana**, the prevalence of overall anaemia is **58.1%**
  - **Mild anaemia:** 32.4%
  - **Moderate anaemia:** 24.4%
  - **Severe anaemia:** 1.4%
- The prevalence of malaria is **17.6%**.

Intervention coverage and costs

Iron supplementation (antenatal care)

- **Current coverage:** 92.3%

- **Maximum feasible coverage:** 97.8%
- **Estimated unit cost:** USD $3.39 (1.51 - 6.34)

Iron supplementation (all women of reproductive age)

- **Current coverage:** 38.0%
- **Maximum feasible coverage:** 97.8%
- **Estimated unit cost:** USD $2.80 (1.37 - 6.69)

Antenatal preventative IPTp-SP

- **Current coverage:** 76.9%
- **Maximum feasible coverage:** 97.8%
- **Estimated unit cost:** USD $2.10 (1.08 - 4.13)

Staple food supplementation

- **Current coverage:** 4.6%
- **Maximum feasible coverage:** 58.8%
- **Estimated unit cost:** USD $0.50 (0.10 - 1.62)

Cost-Effectiveness Analysis

The estimated cost per Year of Life Disabled (YLD) averted for each intervention in **Ghana** is given below, with interquartile ranges (IQR):

Iron supplementation (antenatal care)

- **Median Cost per YLD:** USD $643
- **IQR:** USD $329 – >USD 100k

Iron supplementation (all women of reproductive age)

- **Median Cost per YLD:** USD $936
- **IQR:** USD $629 – $1348

Antenatal preventative IPTp-SP

- **Median Cost per YLD:** USD $1781

- **IQR:** USD $1273 – $2587

Staple food supplementation

- **Median Cost per YLD:** USD $542
- **IQR:** USD $355 – $853

Economic Considerations

Cost-effectiveness thresholds (CET, in USD per DALY averted) in **Ghana** is as follows:

- **Estimates from Pichon-Riviere et al. (Lower Bound):** USD $269
- **Estimates from Pichon-Riviere et al. (Upper Bound):** USD $515
- **CET equal to per capita GDP:** USD $2238
- **CET equal to 2.3 (LMICs) or 1.7 (HICs) times per capita GDP:** USD $5148

⬆ Back to Table of Contents

Anaemia Reduction in Greece

National Target

For our baseline CET = 1 x GDP per capita, the national target for anaemia in **Greece** should be a reduction in prevalence of **16%**. This target is estimated with a 95% uncertainty interval from **6%** to **34%**.

For CET based on Pichon-Riviere, the national target for anaemia in **Greece** should be a reduction in prevalence of **16%**. This target is estimated with a 95% uncertainty interval from **6%** to **32%**.

For a near-infinite CET, the national target for anaemia in **Greece** should be a reduction in prevalence of **24%**. This target is estimated with a 95% uncertainty interval from **13%** to **40%**.

Input parameters

- Among women of reproductive age in **Greece**, the prevalence of overall anaemia is **10.1%**
  - **Mild anaemia:** 8.1%
  - **Moderate anaemia:** 1.9%
  - **Severe anaemia:** 0.1%

- There is no data on malaria prevalence; assume 0%.

Intervention coverage and costs

Iron supplementation (antenatal care)

- **Current coverage:** 81.0%
- **Maximum feasible coverage:** 100.0%
- **Estimated unit cost:** USD $40.23 (9.36 - 63.22)

Iron supplementation (all women of reproductive age)

- **Current coverage:** 33.4%
- **Maximum feasible coverage:** 100.0%
- **Estimated unit cost:** USD $24.15 (6.16 - 49.33)

Antenatal preventative IPTp-SP

- **Current coverage:** 0.0%
- **Maximum feasible coverage:** 100.0%
- **Estimated unit cost:** USD $0.78 (0.46 - 1.75)

Staple food supplementation

- **Current coverage:** 37.2%
- **Maximum feasible coverage:** 100.0%
- **Estimated unit cost:** USD $0.14 (0.04 - 0.28)

Cost-Effectiveness Analysis

The estimated cost per Year of Life Disabled (YLD) averted for each intervention in **Greece** is given below, with interquartile ranges (IQR):

Iron supplementation (antenatal care)

- **Median Cost per YLD:** USD $52233
- **IQR:** USD $33828 – $85547

Iron supplementation (all women of reproductive age)

- **Median Cost per YLD:** USD $90920
- **IQR:** USD $57600 – >USD 100k

Antenatal preventative IPTp-SP

- **Median Cost per YLD:** >USD 100k
- **IQR:** >USD 100k

Staple food supplementation

- **Median Cost per YLD:** USD $1963
- **IQR:** USD $1274 – $3100

Economic Considerations

Cost-effectiveness thresholds (CET, in USD per DALY averted) in **Greece** is as follows:

- **Estimates from Pichon-Riviere et al. (Lower Bound):** USD $13564
- **Estimates from Pichon-Riviere et al. (Upper Bound):** USD $15863
- **CET equal to per capita GDP:** USD $22990
- **CET equal to 2.3 (LMICs) or 1.7 (HICs) times per capita GDP:** USD $32186

⬆ Back to Table of Contents

Anaemia Reduction in Grenada

National Target

For our baseline CET = 1 x GDP per capita, the national target for anaemia in **Grenada** should be a reduction in prevalence of **29%**. This target is estimated with a 95% uncertainty interval from **14%** to **48%**.

For CET based on Pichon-Riviere, the national target for anaemia in **Grenada** should be a reduction in prevalence of **27%**. This target is estimated with a 95% uncertainty interval from **10%** to **45%**.

For a near-infinite CET, the national target for anaemia in **Grenada** should be a reduction in prevalence of **29%**. This target is estimated with a 95% uncertainty interval from **15%** to **47%**.

Input parameters

- Among women of reproductive age in **Grenada**, the prevalence of overall anaemia is **32.7%**
    - **Mild anaemia:** 17.6%
    - **Moderate anaemia:** 13.8%
    - **Severe anaemia:** 1.3%
- There is no data on malaria prevalence; assume 0%.

Intervention coverage and costs

Iron supplementation (antenatal care)

- **Current coverage:** 88.3%
- **Maximum feasible coverage:** 93.3%
- **Estimated unit cost:** USD $12.66 (6.24 - 20.26)

Iron supplementation (all women of reproductive age)

- **Current coverage:** 36.4%
- **Maximum feasible coverage:** 88.3%
- **Estimated unit cost:** USD $7.20 (3.60 - 13.39)

Antenatal preventative IPTp-SP

- **Current coverage:** 0.1%
- **Maximum feasible coverage:** 86.5%
- **Estimated unit cost:** USD $9.15 (4.64 - 14.57)

Staple food supplementation

- **Current coverage:** 0.6%
- **Maximum feasible coverage:** 100.0%
- **Estimated unit cost:** USD $0.71 (0.35 - 1.53)

Cost-Effectiveness Analysis

The estimated cost per Year of Life Disabled (YLD) averted for each intervention in **Grenada** is given below, with interquartile ranges (IQR):

Iron supplementation (antenatal care)

- **Median Cost per YLD:** USD $3382
- **IQR:** USD $1922 – >USD 100k

Iron supplementation (all women of reproductive age)

- **Median Cost per YLD:** USD $3686
- **IQR:** USD $2478 – $5136

Antenatal preventative IPTp-SP

- **Median Cost per YLD:** >USD 100k
- **IQR:** >USD 100k

Staple food supplementation

- **Median Cost per YLD:** USD $1302
- **IQR:** USD $873 – $1881

Economic Considerations

Cost-effectiveness thresholds (CET, in USD per DALY averted) in **Grenada** is as follows:

- **Estimates from Pichon-Riviere et al. (Lower Bound):** USD $3453
- **Estimates from Pichon-Riviere et al. (Upper Bound):** USD $4813
- **CET equal to per capita GDP:** USD $10464
- **CET equal to 2.3 (LMICs) or 1.7 (HICs) times per capita GDP:** USD $24066

⬆ Back to Table of Contents

Anaemia Reduction in Guatemala

National Target

For our baseline CET = 1 x GDP per capita, the national target for anaemia in **Guatemala** should be a reduction in prevalence of **11%**. This target is estimated with a 95% uncertainty interval from **4%** to **20%**.

For CET based on Pichon-Riviere, the national target for anaemia in **Guatemala** should be a reduction in prevalence of **10%**. This target is estimated with a 95% uncertainty interval from **0%** to **19%**.

For a near-infinite CET, the national target for anaemia in **Guatemala** should be a reduction in prevalence of **11%**. This target is estimated with a 95% uncertainty interval from **5%** to **20%**.

Input parameters

- Among women of reproductive age in **Guatemala**, the prevalence of overall anaemia is **33.6%**
  - **Mild anaemia:** 17.4%
  - **Moderate anaemia:** 14.8%
  - **Severe anaemia:** 1.3%
- The prevalence of malaria is **0.0%**.

Intervention coverage and costs

Iron supplementation (antenatal care)

- **Current coverage:** 84.3%
- **Maximum feasible coverage:** 90.9%
- **Estimated unit cost:** USD $6.48 (3.14 - 10.91)

Iron supplementation (all women of reproductive age)

- **Current coverage:** 34.8%
- **Maximum feasible coverage:** 90.9%
- **Estimated unit cost:** USD $4.29 (2.17 - 9.23)

Antenatal preventative IPTp-SP

- **Current coverage:** 0.1%
- **Maximum feasible coverage:** 90.9%

- **Estimated unit cost:** USD $4.38 (2.26 - 7.39)

Staple food supplementation

- **Current coverage:** 8.5%
- **Maximum feasible coverage:** 19.4%
- **Estimated unit cost:** USD $2.08 (1.64 - 3.11)

Cost-Effectiveness Analysis

The estimated cost per Year of Life Disabled (YLD) averted for each intervention in **Guatemala** is given below, with interquartile ranges (IQR):

Iron supplementation (antenatal care)

- **Median Cost per YLD:** USD $1537
- **IQR:** USD $955 – >USD 100k

Iron supplementation (all women of reproductive age)

- **Median Cost per YLD:** USD $2165
- **IQR:** USD $1552 – $3190

Antenatal preventative IPTp-SP

- **Median Cost per YLD:** >USD 100k
- **IQR:** >USD 100k

Staple food supplementation

- **Median Cost per YLD:** USD $2917
- **IQR:** USD $2107 – $4168

Economic Considerations

Cost-effectiveness thresholds (CET, in USD per DALY averted) in **Guatemala** is as follows:

- **Estimates from Pichon-Riviere et al. (Lower Bound):** USD $2493
- **Estimates from Pichon-Riviere et al. (Upper Bound):** USD $3363

- **CET equal to per capita GDP:** USD $5798
- **CET equal to 2.3 (LMICs) or 1.7 (HICs) times per capita GDP:** USD $13334

⬆ Back to Table of Contents

Anaemia Reduction in Guinea-Bissau

National Target

For our baseline CET = 1 x GDP per capita, the national target for anaemia in **Guinea-Bissau** should be a reduction in prevalence of **14%**. This target is estimated with a 95% uncertainty interval from **0%** to **30%**.

For CET based on Pichon-Riviere, the national target for anaemia in **Guinea-Bissau** should be a reduction in prevalence of **1%**. This target is estimated with a 95% uncertainty interval from **0%** to **21%**.

For a near-infinite CET, the national target for anaemia in **Guinea-Bissau** should be a reduction in prevalence of **22%**. This target is estimated with a 95% uncertainty interval from **13%** to **32%**.

Input parameters

- Among women of reproductive age in **Guinea-Bissau**, the prevalence of overall anaemia is **56.5%**
  - **Mild anaemia:** 30.5%
  - **Moderate anaemia:** 24.3%
  - **Severe anaemia:** 1.7%
- The prevalence of malaria is **5.2%**.

Intervention coverage and costs

Iron supplementation (antenatal care)

- **Current coverage:** 77.9%
- **Maximum feasible coverage:** 100.0%
- **Estimated unit cost:** USD $2.96 (1.31 - 5.41)

Iron supplementation (all women of reproductive age)

- **Current coverage:** 32.1%
- **Maximum feasible coverage:** 100.0%
- **Estimated unit cost:** USD $2.82 (1.86 - 9.65)

Antenatal preventative IPTp-SP

- **Current coverage:** 53.1%
- **Maximum feasible coverage:** 100.0%
- **Estimated unit cost:** USD $1.62 (0.84 - 3.04)

Staple food supplementation

- **Current coverage:** 0.9%
- **Maximum feasible coverage:** 47.1%
- **Estimated unit cost:** USD $0.69 (0.10 - 2.10)

Cost-Effectiveness Analysis

The estimated cost per Year of Life Disabled (YLD) averted for each intervention in **Guinea-Bissau** is given below, with interquartile ranges (IQR):

Iron supplementation (antenatal care)

- **Median Cost per YLD:** USD $303
- **IQR:** USD $207 – $456

Iron supplementation (all women of reproductive age)

- **Median Cost per YLD:** USD $1123
- **IQR:** USD $744 – $1635

Antenatal preventative IPTp-SP

- **Median Cost per YLD:** USD $2829
- **IQR:** USD $2177 – $3864

Staple food supplementation

- **Median Cost per YLD:** USD $705
- **IQR:** USD $449 – $1097

Economic Considerations

Cost-effectiveness thresholds (CET, in USD per DALY averted) in **Guinea-Bissau** is as follows:

- **Estimates from Pichon-Riviere et al. (Lower Bound):** USD $101
- **Estimates from Pichon-Riviere et al. (Upper Bound):** USD $311
- **CET equal to per capita GDP:** USD $914
- **CET equal to 2.3 (LMICs) or 1.7 (HICs) times per capita GDP:** USD $2103

⬆ Back to Table of Contents

Anaemia Reduction in Guinea

National Target

For our baseline CET = 1 x GDP per capita, the national target for anaemia in **Guinea** should be a reduction in prevalence of **18%**. This target is estimated with a 95% uncertainty interval from **1%** to **32%**.

For CET based on Pichon-Riviere, the national target for anaemia in **Guinea** should be a reduction in prevalence of **0%**. This target is estimated with a 95% uncertainty interval from **0%** to **18%**.

For a near-infinite CET, the national target for anaemia in **Guinea** should be a reduction in prevalence of **20%**. This target is estimated with a 95% uncertainty interval from **11%** to **32%**.

Input parameters

- Among women of reproductive age in **Guinea**, the prevalence of overall anaemia is **46.8%**
  - **Mild anaemia:** 23.6%
  - **Moderate anaemia:** 21.7%
  - **Severe anaemia:** 1.5%
- The prevalence of malaria is **29.2%**.

Intervention coverage and costs

Iron supplementation (antenatal care)

- **Current coverage:** 80.6%
- **Maximum feasible coverage:** 86.0%
- **Estimated unit cost:** USD $2.72 (1.16 - 4.89)

Iron supplementation (all women of reproductive age)

- **Current coverage:** 33.2%
- **Maximum feasible coverage:** 86.0%
- **Estimated unit cost:** USD $2.59 (1.27 - 6.39)

Antenatal preventative IPTp-SP

- **Current coverage:** 72.5%
- **Maximum feasible coverage:** 86.0%
- **Estimated unit cost:** USD $1.46 (0.76 - 2.81)

Staple food supplementation

- **Current coverage:** 0.2%
- **Maximum feasible coverage:** 50.4%
- **Estimated unit cost:** USD $0.65 (0.10 - 2.01)

Cost-Effectiveness Analysis

The estimated cost per Year of Life Disabled (YLD) averted for each intervention in **Guinea** is given below, with interquartile ranges (IQR):

Iron supplementation (antenatal care)

- **Median Cost per YLD:** USD $513
- **IQR:** USD $312 – >USD 100k

Iron supplementation (all women of reproductive age)

- **Median Cost per YLD:** USD $1066
- **IQR:** USD $745 – $1561

Antenatal preventative IPTp-SP

- **Median Cost per YLD:** USD $1008
- **IQR:** USD $731 – $1579

Staple food supplementation

- **Median Cost per YLD:** USD $931
- **IQR:** USD $599 – $1526

Economic Considerations

Cost-effectiveness thresholds (CET, in USD per DALY averted) in **Guinea** is as follows:

- **Estimates from Pichon-Riviere et al. (Lower Bound):** USD $100
- **Estimates from Pichon-Riviere et al. (Upper Bound):** USD $300
- **CET equal to per capita GDP:** USD $1664
- **CET equal to 2.3 (LMICs) or 1.7 (HICs) times per capita GDP:** USD $3827

⬆ Back to Table of Contents

Anaemia Reduction in Guyana

National Target

For our baseline CET = 1 x GDP per capita, the national target for anaemia in **Guyana** should be a reduction in prevalence of **16%**. This target is estimated with a 95% uncertainty interval from **8%** to **27%**.

For CET based on Pichon-Riviere, the national target for anaemia in **Guyana** should be a reduction in prevalence of **16%**. This target is estimated with a 95% uncertainty interval from **8%** to **26%**.

For a near-infinite CET, the national target for anaemia in **Guyana** should be a reduction in prevalence of **16%**. This target is estimated with a 95% uncertainty interval from **8%** to **26%**.

Input parameters

- Among women of reproductive age in **Guyana**, the prevalence of overall anaemia is **37.9%**

- **Mild anaemia:** 21.8%
- **Moderate anaemia:** 15.1%
- **Severe anaemia:** 1.0%
- The prevalence of malaria is **0.9%**.

Intervention coverage and costs

Iron supplementation (antenatal care)

- **Current coverage:** 81.2%
- **Maximum feasible coverage:** 83.7%
- **Estimated unit cost:** USD $6.32 (3.12 - 11.05)

Iron supplementation (all women of reproductive age)

- **Current coverage:** 33.5%
- **Maximum feasible coverage:** 83.7%
- **Estimated unit cost:** USD $4.62 (2.79 - 13.24)

Antenatal preventative IPTp-SP

- **Current coverage:** 0.1%
- **Maximum feasible coverage:** 83.7%
- **Estimated unit cost:** USD $4.34 (2.24 - 7.42)

Staple food supplementation

- **Current coverage:** 0.0%
- **Maximum feasible coverage:** 36.1%
- **Estimated unit cost:** USD $0.55 (0.11 - 1.55)

Cost-Effectiveness Analysis

The estimated cost per Year of Life Disabled (YLD) averted for each intervention in **Guyana** is given below, with interquartile ranges (IQR):

Iron supplementation (antenatal care)

- **Median Cost per YLD:** USD $1756
- **IQR:** USD $1001 – >USD 100k

Iron supplementation (all women of reproductive age)

- **Median Cost per YLD:** USD $2903
- **IQR:** USD $2022 – $4264

Antenatal preventative IPTp-SP

- **Median Cost per YLD:** USD $66907
- **IQR:** USD $52781 – $88746

Staple food supplementation

- **Median Cost per YLD:** USD $950
- **IQR:** USD $615 – $1480

Economic Considerations

Cost-effectiveness thresholds (CET, in USD per DALY averted) in **Guyana** is as follows:

- **Estimates from Pichon-Riviere et al. (Lower Bound):** USD $7219
- **Estimates from Pichon-Riviere et al. (Upper Bound):** USD $9694
- **CET equal to per capita GDP:** USD $20626
- **CET equal to 2.3 (LMICs) or 1.7 (HICs) times per capita GDP:** USD $28877

⬆ Back to Table of Contents

Anaemia Reduction in Haiti

National Target

For our baseline CET = 1 x GDP per capita, the national target for anaemia in **Haiti** should be a reduction in prevalence of **22%**. This target is estimated with a 95% uncertainty interval from **7%** to **39%**.

For CET based on Pichon-Riviere, the national target for anaemia in **Haiti** should be a reduction in prevalence of **0%**. This target is estimated with a 95% uncertainty interval from **0%** to **31%**.

For a near-infinite CET, the national target for anaemia in **Haiti** should be a reduction in prevalence of **23%**. This target is estimated with a 95% uncertainty interval from **12%** to **40%**.

Input parameters

- Among women of reproductive age in **Haiti**, the prevalence of overall anaemia is **58.8%**
  - **Mild anaemia:** 32.1%
  - **Moderate anaemia:** 25.2%
  - **Severe anaemia:** 1.5%
- The prevalence of malaria is **0.3%**.

Intervention coverage and costs

Iron supplementation (antenatal care)

- **Current coverage:** 77.6%
- **Maximum feasible coverage:** 89.7%
- **Estimated unit cost:** USD $3.47 (1.64 - 6.54)

Iron supplementation (all women of reproductive age)

- **Current coverage:** 32.0%
- **Maximum feasible coverage:** 89.7%
- **Estimated unit cost:** USD $3.01 (1.55 - 7.26)

Antenatal preventative IPTp-SP

- **Current coverage:** 0.0%
- **Maximum feasible coverage:** 89.7%
- **Estimated unit cost:** USD $2.13 (1.13 - 4.10)

Staple food supplementation

- **Current coverage:** 9.1%
- **Maximum feasible coverage:** 79.3%
- **Estimated unit cost:** USD $0.52 (0.09 - 1.64)

Cost-Effectiveness Analysis

The estimated cost per Year of Life Disabled (YLD) averted for each intervention in **Haiti** is given below, with interquartile ranges (IQR):

Iron supplementation (antenatal care)

- **Median Cost per YLD:** USD $515
- **IQR:** USD $320 – >USD 100k

Iron supplementation (all women of reproductive age)

- **Median Cost per YLD:** USD $1018
- **IQR:** USD $721 – $1465

Antenatal preventative IPTp-SP

- **Median Cost per YLD:** USD $65683
- **IQR:** USD $49627 – $85988

Staple food supplementation

- **Median Cost per YLD:** USD $533
- **IQR:** USD $344 – $827

Economic Considerations

Cost-effectiveness thresholds (CET, in USD per DALY averted) in **Haiti** is as follows:

- **Estimates from Pichon-Riviere et al. (Lower Bound):** USD $102
- **Estimates from Pichon-Riviere et al. (Upper Bound):** USD $339
- **CET equal to per capita GDP:** USD $1693
- **CET equal to 2.3 (LMICs) or 1.7 (HICs) times per capita GDP:** USD $3894

⬆ Back to Table of Contents

Anaemia Reduction in Honduras

National Target

For our baseline CET = 1 x GDP per capita, the national target for anaemia in **Honduras** should be a reduction in prevalence of **4%**. This target is estimated with a 95% uncertainty interval from **0%** to **15%**.

For CET based on Pichon-Riviere, the national target for anaemia in **Honduras** should be a reduction in prevalence of **2%**. This target is estimated with a 95% uncertainty interval from **0%** to **7%**.

For a near-infinite CET, the national target for anaemia in **Honduras** should be a reduction in prevalence of **14%**. This target is estimated with a 95% uncertainty interval from **8%** to **24%**.

Input parameters

- Among women of reproductive age in **Honduras**, the prevalence of overall anaemia is **16.3%**
  - **Mild anaemia:** 10.7%
  - **Moderate anaemia:** 5.3%
  - **Severe anaemia:** 0.3%
- The prevalence of malaria is **0.0%**.

Intervention coverage and costs

Iron supplementation (antenatal care)

- **Current coverage:** 60.2%
- **Maximum feasible coverage:** 96.6%
- **Estimated unit cost:** USD $5.72 (2.82 - 10.13)

Iron supplementation (all women of reproductive age)

- **Current coverage:** 24.8%
- **Maximum feasible coverage:** 96.6%
- **Estimated unit cost:** USD $4.36 (2.67 - 12.52)

Antenatal preventative IPTp-SP

- **Current coverage:** 0.0%
- **Maximum feasible coverage:** 96.6%
- **Estimated unit cost:** USD $3.80 (1.97 - 6.57)

Staple food supplementation

- **Current coverage:** 7.3%
- **Maximum feasible coverage:** 19.8%
- **Estimated unit cost:** USD $0.34 (0.10 - 1.21)

Cost-Effectiveness Analysis

The estimated cost per Year of Life Disabled (YLD) averted for each intervention in **Honduras** is given below, with interquartile ranges (IQR):

Iron supplementation (antenatal care)

- **Median Cost per YLD:** USD $3257
- **IQR:** USD $2338 – $4449

Iron supplementation (all women of reproductive age)

- **Median Cost per YLD:** USD $8209
- **IQR:** USD $5725 – $12214

Antenatal preventative IPTp-SP

- **Median Cost per YLD:** >USD 100k
- **IQR:** >USD 100k

Staple food supplementation

- **Median Cost per YLD:** USD $1909
- **IQR:** USD $1250 – $2977

Economic Considerations

Cost-effectiveness thresholds (CET, in USD per DALY averted) in **Honduras** is as follows:

- **Estimates from Pichon-Riviere et al. (Lower Bound):** USD $1039
- **Estimates from Pichon-Riviere et al. (Upper Bound):** USD $2013
- **CET equal to per capita GDP:** USD $3247

- **CET equal to 2.3 (LMICs) or 1.7 (HICs) times per capita GDP:** USD $7469

⬆ Back to Table of Contents

Anaemia Reduction in Hungary

National Target

For our baseline CET = 1 x GDP per capita, the national target for anaemia in **Hungary** should be a reduction in prevalence of **18%**. This target is estimated with a 95% uncertainty interval from **6%** to **37%**.

For CET based on Pichon-Riviere, the national target for anaemia in **Hungary** should be a reduction in prevalence of **16%**. This target is estimated with a 95% uncertainty interval from **6%** to **33%**.

For a near-infinite CET, the national target for anaemia in **Hungary** should be a reduction in prevalence of **24%**. This target is estimated with a 95% uncertainty interval from **13%** to **40%**.

Input parameters

- Among women of reproductive age in **Hungary**, the prevalence of overall anaemia is **20.6%**
  - **Mild anaemia:** 13.6%
  - **Moderate anaemia:** 6.6%
  - **Severe anaemia:** 0.4%
- There is no data on malaria prevalence; assume 0%.

Intervention coverage and costs

Iron supplementation (antenatal care)

- **Current coverage:** 81.0%
- **Maximum feasible coverage:** 100.0%
- **Estimated unit cost:** USD $60.27 (19.32 - 97.72)

Iron supplementation (all women of reproductive age)

- **Current coverage:** 33.4%
- **Maximum feasible coverage:** 100.0%

- **Estimated unit cost:** USD $38.13 (11.12 - 55.18)

Antenatal preventative IPTp-SP

- **Current coverage:** 0.0%
- **Maximum feasible coverage:** 100.0%
- **Estimated unit cost:** USD $18.29 (9.21 - 30.02)

Staple food supplementation

- **Current coverage:** 38.1%
- **Maximum feasible coverage:** 100.0%
- **Estimated unit cost:** USD $0.08 (0.01 - 0.17)

Cost-Effectiveness Analysis

The estimated cost per Year of Life Disabled (YLD) averted for each intervention in **Hungary** is given below, with interquartile ranges (IQR):

Iron supplementation (antenatal care)

- **Median Cost per YLD:** USD $22269
- **IQR:** USD $14911 – $35841

Iron supplementation (all women of reproductive age)

- **Median Cost per YLD:** USD $31947
- **IQR:** USD $22605 – $48705

Antenatal preventative IPTp-SP

- **Median Cost per YLD:** >USD 100k
- **IQR:** >USD 100k

Staple food supplementation

- **Median Cost per YLD:** USD $269
- **IQR:** USD $173 – $441

Economic Considerations

Cost-effectiveness thresholds (CET, in USD per DALY averted) in **Hungary** is as follows:

- **Estimates from Pichon-Riviere et al. (Lower Bound):** USD $9745
- **Estimates from Pichon-Riviere et al. (Upper Bound):** USD $11517
- **CET equal to per capita GDP:** USD $22147
- **CET equal to 2.3 (LMICs) or 1.7 (HICs) times per capita GDP:** USD $31006

⬆ Back to Table of Contents

Anaemia Reduction in Iceland

National Target

For our baseline CET = 1 x GDP per capita, the national target for anaemia in **Iceland** should be a reduction in prevalence of **16%**. This target is estimated with a 95% uncertainty interval from **6%** to **34%**.

For CET based on Pichon-Riviere, the national target for anaemia in **Iceland** should be a reduction in prevalence of **16%**. This target is estimated with a 95% uncertainty interval from **6%** to **33%**.

For a near-infinite CET, the national target for anaemia in **Iceland** should be a reduction in prevalence of **25%**. This target is estimated with a 95% uncertainty interval from **14%** to **41%**.

Input parameters

- Among women of reproductive age in **Iceland**, the prevalence of overall anaemia is **6.7%**
  - **Mild anaemia:** 5.3%
  - **Moderate anaemia:** 1.3%
  - **Severe anaemia:** 0.0%
- There is no data on malaria prevalence; assume 0%.

Intervention coverage and costs

Iron supplementation (antenatal care)

- **Current coverage:** 81.0%

- **Maximum feasible coverage:** 100.0%
- **Estimated unit cost:** USD $114.78 (46.24 - 183.21)

Iron supplementation (all women of reproductive age)

- **Current coverage:** 33.4%
- **Maximum feasible coverage:** 100.0%
- **Estimated unit cost:** USD $71.83 (27.94 - 107.98)

Antenatal preventative IPTp-SP

- **Current coverage:** 0.0%
- **Maximum feasible coverage:** 100.0%
- **Estimated unit cost:** USD $58.02 (29.08 - 91.39)

Staple food supplementation

- **Current coverage:** 36.1%
- **Maximum feasible coverage:** 100.0%
- **Estimated unit cost:** USD $0.17 (0.02 - 0.43)

Cost-Effectiveness Analysis

The estimated cost per Year of Life Disabled (YLD) averted for each intervention in **Iceland** is given below, with interquartile ranges (IQR):

Iron supplementation (antenatal care)

- **Median Cost per YLD:** >USD 100k
- **IQR:** >USD 100k

Iron supplementation (all women of reproductive age)

- **Median Cost per YLD:** >USD 100k
- **IQR:** >USD 100k

Antenatal preventative IPTp-SP

- **Median Cost per YLD:** >USD 100k

- **IQR:** >USD 100k

Staple food supplementation

- **Median Cost per YLD:** USD $3304
- **IQR:** USD $1971 – $5531

Economic Considerations

Cost-effectiveness thresholds (CET, in USD per DALY averted) in **Iceland** is as follows:

- **Estimates from Pichon-Riviere et al. (Lower Bound):** USD $55168
- **Estimates from Pichon-Riviere et al. (Upper Bound):** USD $64625
- **CET equal to per capita GDP:** USD $78811
- **CET equal to 2.3 (LMICs) or 1.7 (HICs) times per capita GDP:** USD $110335

⬆ Back to Table of Contents

Anaemia Reduction in India

National Target

For our baseline CET = 1 x GDP per capita, the national target for anaemia in **India** should be a reduction in prevalence of **14%**. This target is estimated with a 95% uncertainty interval from **7%** to **24%**.

For CET based on Pichon-Riviere, the national target for anaemia in **India** should be a reduction in prevalence of **8%**. This target is estimated with a 95% uncertainty interval from **0%** to **21%**.

For a near-infinite CET, the national target for anaemia in **India** should be a reduction in prevalence of **14%**. This target is estimated with a 95% uncertainty interval from **8%** to **24%**.

Input parameters

- Among women of reproductive age in **India**, the prevalence of overall anaemia is **55.7%**
  - **Mild anaemia:** 28.3%
  - **Moderate anaemia:** 24.9%
  - **Severe anaemia:** 2.5%
- The prevalence of malaria is **0.1%**.

Intervention coverage and costs

Iron supplementation (antenatal care)

- **Current coverage:** 88.1%
- **Maximum feasible coverage:** 93.1%
- **Estimated unit cost:** USD $2.70 (1.24 - 5.03)

Iron supplementation (all women of reproductive age)

- **Current coverage:** 36.3%
- **Maximum feasible coverage:** 88.1%
- **Estimated unit cost:** USD $2.34 (1.17 - 5.71)

Antenatal preventative IPTp-SP

- **Current coverage:** 0.0%
- **Maximum feasible coverage:** 85.1%
- **Estimated unit cost:** USD $1.55 (0.83 - 2.94)

Staple food supplementation

- **Current coverage:** 0.1%
- **Maximum feasible coverage:** 30.0%
- **Estimated unit cost:** USD $0.41 (0.06 - 1.27)

Cost-Effectiveness Analysis

The estimated cost per Year of Life Disabled (YLD) averted for each intervention in **India** is given below, with interquartile ranges (IQR):

Iron supplementation (antenatal care)

- **Median Cost per YLD:** USD $445
- **IQR:** USD $262 – >USD 100k

Iron supplementation (all women of reproductive age)

- **Median Cost per YLD:** USD $817
- **IQR:** USD $590 – $1154

Antenatal preventative IPTp-SP

- **Median Cost per YLD:** >USD 100k
- **IQR:** >USD 100k

Staple food supplementation

- **Median Cost per YLD:** USD $479
- **IQR:** USD $317 – $720

Economic Considerations

Cost-effectiveness thresholds (CET, in USD per DALY averted) in **India** is as follows:

- **Estimates from Pichon-Riviere et al. (Lower Bound):** USD $298
- **Estimates from Pichon-Riviere et al. (Upper Bound):** USD $596
- **CET equal to per capita GDP:** USD $2485
- **CET equal to 2.3 (LMICs) or 1.7 (HICs) times per capita GDP:** USD $5715

⬆ Back to Table of Contents

Anaemia Reduction in Indonesia

National Target

For our baseline CET = 1 x GDP per capita, the national target for anaemia in **Indonesia** should be a reduction in prevalence of **9%**. This target is estimated with a 95% uncertainty interval from **1%** to **23%**.

For CET based on Pichon-Riviere, the national target for anaemia in **Indonesia** should be a reduction in prevalence of **0%**. This target is estimated with a 95% uncertainty interval from **0%** to **10%**.

For a near-infinite CET, the national target for anaemia in **Indonesia** should be a reduction in prevalence of **14%**. This target is estimated with a 95% uncertainty interval from **7%** to **22%**.

Input parameters

- Among women of reproductive age in **Indonesia**, the prevalence of overall anaemia is **28.1%**
  - **Mild anaemia:** 20.6%
  - **Moderate anaemia:** 7.2%
  - **Severe anaemia:** 0.3%
- The prevalence of malaria is **0.1%**.

Intervention coverage and costs

Iron supplementation (antenatal care)

- **Current coverage:** 86.2%
- **Maximum feasible coverage:** 97.5%
- **Estimated unit cost:** USD $3.68 (1.77 - 6.79)

Iron supplementation (all women of reproductive age)

- **Current coverage:** 35.5%
- **Maximum feasible coverage:** 97.5%
- **Estimated unit cost:** USD $3.07 (2.01 - 10.62)

Antenatal preventative IPTp-SP

- **Current coverage:** 0.0%
- **Maximum feasible coverage:** 97.5%
- **Estimated unit cost:** USD $2.16 (1.14 - 3.90)

Staple food supplementation

- **Current coverage:** 4.2%
- **Maximum feasible coverage:** 23.2%
- **Estimated unit cost:** USD $0.62 (0.10 - 1.93)

Cost-Effectiveness Analysis

The estimated cost per Year of Life Disabled (YLD) averted for each intervention in **Indonesia** is given below, with interquartile ranges (IQR):

Iron supplementation (antenatal care)

- **Median Cost per YLD:** USD $1750
- **IQR:** USD $1148 – >USD 100k

Iron supplementation (all women of reproductive age)

- **Median Cost per YLD:** USD $4595
- **IQR:** USD $3037 – $6981

Antenatal preventative IPTp-SP

- **Median Cost per YLD:** >USD 100k
- **IQR:** >USD 100k

Staple food supplementation

- **Median Cost per YLD:** USD $2442
- **IQR:** USD $1515 – $3786

Economic Considerations

Cost-effectiveness thresholds (CET, in USD per DALY averted) in **Indonesia** is as follows:

- **Estimates from Pichon-Riviere et al. (Lower Bound):** USD $988
- **Estimates from Pichon-Riviere et al. (Upper Bound):** USD $1334
- **CET equal to per capita GDP:** USD $4941
- **CET equal to 2.3 (LMICs) or 1.7 (HICs) times per capita GDP:** USD $11363

⬆ Back to Table of Contents

Anaemia Reduction in Iran

National Target

For our baseline CET = 1 x GDP per capita, the national target for anaemia in **Iran** should be a reduction in prevalence of **6%**. This target is estimated with a 95% uncertainty interval from **0%** to **19%**.

For CET based on Pichon-Riviere, the national target for anaemia in **Iran** should be a reduction in prevalence of **5%**. This target is estimated with a 95% uncertainty interval from **0%** to **21%**.

For a near-infinite CET, the national target for anaemia in **Iran** should be a reduction in prevalence of **12%**. This target is estimated with a 95% uncertainty interval from **5%** to **25%**.

Input parameters

- Among women of reproductive age in **Iran**, the prevalence of overall anaemia is **28.2%**
    - **Mild anaemia:** 16.4%
    - **Moderate anaemia:** 10.7%
    - **Severe anaemia:** 1.0%
- The prevalence of malaria is **0.0%**.

Intervention coverage and costs

Iron supplementation (antenatal care)

- **Current coverage:** NaN%
- **Maximum feasible coverage:** NaN%
- **Estimated unit cost:** USD $42.13 (20.91 - 64.97)

Iron supplementation (all women of reproductive age)

- **Current coverage:** NaN%
- **Maximum feasible coverage:** NaN%
- **Estimated unit cost:** USD $19.53 (9.76 - 32.83)

Antenatal preventative IPTp-SP

- **Current coverage:** NaN%
- **Maximum feasible coverage:** NaN%
- **Estimated unit cost:** USD $31.31 (15.71 - 48.50)

Staple food supplementation

- **Current coverage:** NaN%
- **Maximum feasible coverage:** NaN%
- **Estimated unit cost:** USD $0.19 (0.05 - 0.45)

Cost-Effectiveness Analysis

The estimated cost per Year of Life Disabled (YLD) averted for each intervention in **Iran** is given below, with interquartile ranges (IQR):

Iron supplementation (antenatal care)

- **Median Cost per YLD:** USD $11528
- **IQR:** USD $8144 – $17254

Iron supplementation (all women of reproductive age)

- **Median Cost per YLD:** USD $12565
- **IQR:** USD $8932 – $17340

Antenatal preventative IPTp-SP

- **Median Cost per YLD:** >USD 100k
- **IQR:** >USD 100k

Staple food supplementation

- **Median Cost per YLD:** USD $361
- **IQR:** USD $230 – $632

Economic Considerations

Cost-effectiveness thresholds (CET, in USD per DALY averted) in **Iran** is as follows:

- **Estimates from Pichon-Riviere et al. (Lower Bound):** USD $4412
- **Estimates from Pichon-Riviere et al. (Upper Bound):** USD $6033
- **CET equal to per capita GDP:** USD $4503
- **CET equal to 2.3 (LMICs) or 1.7 (HICs) times per capita GDP:** USD $10356

⬆ Back to Table of Contents

Anaemia Reduction in Iraq

National Target

For our baseline CET = 1 x GDP per capita, the national target for anaemia in **Iraq** should be a reduction in prevalence of **15%**. This target is estimated with a 95% uncertainty interval from **4%** to **28%**.

For CET based on Pichon-Riviere, the national target for anaemia in **Iraq** should be a reduction in prevalence of **10%**. This target is estimated with a 95% uncertainty interval from **2%** to **25%**.

For a near-infinite CET, the national target for anaemia in **Iraq** should be a reduction in prevalence of **15%**. This target is estimated with a 95% uncertainty interval from **6%** to **29%**.

Input parameters

- Among women of reproductive age in **Iraq**, the prevalence of overall anaemia is **30.8%**
  - **Mild anaemia:** 19.5%
  - **Moderate anaemia:** 10.7%
  - **Severe anaemia:** 0.6%
- The prevalence of malaria is **0.0%**.

Intervention coverage and costs

Iron supplementation (antenatal care)

- **Current coverage:** 80.9%
- **Maximum feasible coverage:** 85.9%
- **Estimated unit cost:** USD $4.92 (2.31 - 9.16)

Iron supplementation (all women of reproductive age)

- **Current coverage:** 33.4%
- **Maximum feasible coverage:** 80.9%
- **Estimated unit cost:** USD $4.11 (2.05 - 9.69)

Antenatal preventative IPTp-SP

- **Current coverage:** 0.0%
- **Maximum feasible coverage:** 68.0%
- **Estimated unit cost:** USD $3.41 (1.75 - 6.64)

Staple food supplementation

- **Current coverage:** 66.2%
- **Maximum feasible coverage:** 98.9%
- **Estimated unit cost:** USD $0.21 (0.03 - 0.56)

Cost-Effectiveness Analysis

The estimated cost per Year of Life Disabled (YLD) averted for each intervention in **Iraq** is given below, with interquartile ranges (IQR):

Iron supplementation (antenatal care)

- **Median Cost per YLD:** USD $1778
- **IQR:** USD $1033 – >USD 100k

Iron supplementation (all women of reproductive age)

- **Median Cost per YLD:** USD $3129
- **IQR:** USD $2173 – $4480

Antenatal preventative IPTp-SP

- **Median Cost per YLD:** >USD 100k
- **IQR:** >USD 100k

Staple food supplementation

- **Median Cost per YLD:** USD $422
- **IQR:** USD $262 – $639

Economic Considerations

Cost-effectiveness thresholds (CET, in USD per DALY averted) in **Iraq** is as follows:

- **Estimates from Pichon-Riviere et al. (Lower Bound):** USD $1543
- **Estimates from Pichon-Riviere et al. (Upper Bound):** USD $2095
- **CET equal to per capita GDP:** USD $5512
- **CET equal to 2.3 (LMICs) or 1.7 (HICs) times per capita GDP:** USD $12679

⬆ Back to Table of Contents

Anaemia Reduction in Ireland

National Target

For our baseline CET = 1 x GDP per capita, the national target for anaemia in **Ireland** should be a reduction in prevalence of **18%**. This target is estimated with a 95% uncertainty interval from **6%** to **37%**.

For CET based on Pichon-Riviere, the national target for anaemia in **Ireland** should be a reduction in prevalence of **17%**. This target is estimated with a 95% uncertainty interval from **6%** to **34%**.

For a near-infinite CET, the national target for anaemia in **Ireland** should be a reduction in prevalence of **25%**. This target is estimated with a 95% uncertainty interval from **14%** to **42%**.

Input parameters

- Among women of reproductive age in **Ireland**, the prevalence of overall anaemia is **7.6%**
    - **Mild anaemia:** 6.3%
    - **Moderate anaemia:** 1.3%
    - **Severe anaemia:** 0.0%
- There is no data on malaria prevalence; assume 0%.

Intervention coverage and costs

Iron supplementation (antenatal care)

- **Current coverage:** 81.0%
- **Maximum feasible coverage:** 100.0%

- **Estimated unit cost:** USD $59.83 (18.88 - 98.06)

Iron supplementation (all women of reproductive age)

- **Current coverage:** 33.4%
- **Maximum feasible coverage:** 100.0%
- **Estimated unit cost:** USD $37.71 (10.81 - 56.40)

Antenatal preventative IPTp-SP

- **Current coverage:** 0.0%
- **Maximum feasible coverage:** 100.0%
- **Estimated unit cost:** USD $16.27 (8.20 - 27.23)

Staple food supplementation

- **Current coverage:** 33.3%
- **Maximum feasible coverage:** 100.0%
- **Estimated unit cost:** USD $0.44 (0.30 - 0.70)

Cost-Effectiveness Analysis

The estimated cost per Year of Life Disabled (YLD) averted for each intervention in **Ireland** is given below, with interquartile ranges (IQR):

Iron supplementation (antenatal care)

- **Median Cost per YLD:** >USD 100k
- **IQR:** USD $73068 – >USD 100k

Iron supplementation (all women of reproductive age)

- **Median Cost per YLD:** >USD 100k
- **IQR:** >USD 100k

Antenatal preventative IPTp-SP

- **Median Cost per YLD:** >USD 100k
- **IQR:** >USD 100k

Staple food supplementation

- **Median Cost per YLD:** USD $7792
- **IQR:** USD $5307 – $12647

Economic Considerations

Cost-effectiveness thresholds (CET, in USD per DALY averted) in **Ireland** is as follows:

- **Estimates from Pichon-Riviere et al. (Lower Bound):** USD $52879
- **Estimates from Pichon-Riviere et al. (Upper Bound):** USD $62211
- **CET equal to per capita GDP:** USD $103685
- **CET equal to 2.3 (LMICs) or 1.7 (HICs) times per capita GDP:** USD $145159

⬆ Back to Table of Contents

Anaemia Reduction in Israel

National Target

For our baseline CET = 1 x GDP per capita, the national target for anaemia in **Israel** should be a reduction in prevalence of **16%**. This target is estimated with a 95% uncertainty interval from **6%** to **35%**.

For CET based on Pichon-Riviere, the national target for anaemia in **Israel** should be a reduction in prevalence of **16%**. This target is estimated with a 95% uncertainty interval from **6%** to **34%**.

For a near-infinite CET, the national target for anaemia in **Israel** should be a reduction in prevalence of **25%**. This target is estimated with a 95% uncertainty interval from **14%** to **41%**.

Input parameters

- Among women of reproductive age in **Israel**, the prevalence of overall anaemia is **9.7%**
  - **Mild anaemia:** 8.0%
  - **Moderate anaemia:** 1.7%
  - **Severe anaemia:** 0.0%
- There is no data on malaria prevalence; assume 0%.

Intervention coverage and costs

Iron supplementation (antenatal care)

- **Current coverage:** 81.0%
- **Maximum feasible coverage:** 100.0%
- **Estimated unit cost:** USD $88.24 (33.24 - 140.83)

Iron supplementation (all women of reproductive age)

- **Current coverage:** 33.4%
- **Maximum feasible coverage:** 100.0%
- **Estimated unit cost:** USD $55.30 (19.76 - 80.20)

Antenatal preventative IPTp-SP

- **Current coverage:** 0.0%
- **Maximum feasible coverage:** 100.0%
- **Estimated unit cost:** USD $39.61 (19.87 - 62.24)

Staple food supplementation

- **Current coverage:** 34.2%
- **Maximum feasible coverage:** 95.3%
- **Estimated unit cost:** USD $0.30 (0.13 - 0.62)

Cost-Effectiveness Analysis

The estimated cost per Year of Life Disabled (YLD) averted for each intervention in **Israel** is given below, with interquartile ranges (IQR):

Iron supplementation (antenatal care)

- **Median Cost per YLD:** >USD 100k
- **IQR:** USD $89039 – >USD 100k

Iron supplementation (all women of reproductive age)

- **Median Cost per YLD:** >USD 100k
- **IQR:** >USD 100k

Antenatal preventative IPTp-SP

- **Median Cost per YLD:** >USD 100k
- **IQR:** >USD 100k

Staple food supplementation

- **Median Cost per YLD:** USD $4480
- **IQR:** USD $2829 – $7301

Economic Considerations

Cost-effectiveness thresholds (CET, in USD per DALY averted) in **Israel** is as follows:

- **Estimates from Pichon-Riviere et al. (Lower Bound):** USD $30834
- **Estimates from Pichon-Riviere et al. (Upper Bound):** USD $36583
- **CET equal to per capita GDP:** USD $52262
- **CET equal to 2.3 (LMICs) or 1.7 (HICs) times per capita GDP:** USD $73166

⬆ Back to Table of Contents

Anaemia Reduction in Italy

National Target

For our baseline CET = 1 x GDP per capita, the national target for anaemia in **Italy** should be a reduction in prevalence of **16%**. This target is estimated with a 95% uncertainty interval from **6%** to **34%**.

For CET based on Pichon-Riviere, the national target for anaemia in **Italy** should be a reduction in prevalence of **16%**. This target is estimated with a 95% uncertainty interval from **6%** to **32%**.

For a near-infinite CET, the national target for anaemia in **Italy** should be a reduction in prevalence of **24%**. This target is estimated with a 95% uncertainty interval from **13%** to **40%**.

Input parameters

- Among women of reproductive age in **Italy**, the prevalence of overall anaemia is **8.4%**
  - **Mild anaemia:** 6.9%
  - **Moderate anaemia:** 1.5%
  - **Severe anaemia:** 0.0%
- There is no data on malaria prevalence; assume 0%.

Intervention coverage and costs

Iron supplementation (antenatal care)

- **Current coverage:** 81.0%
- **Maximum feasible coverage:** 100.0%
- **Estimated unit cost:** USD $54.76 (16.51 - 90.38)

Iron supplementation (all women of reproductive age)

- **Current coverage:** 33.4%
- **Maximum feasible coverage:** 100.0%
- **Estimated unit cost:** USD $36.08 (10.14 - 51.60)

Antenatal preventative IPTp-SP

- **Current coverage:** 0.0%
- **Maximum feasible coverage:** 100.0%
- **Estimated unit cost:** USD $14.32 (7.23 - 24.30)

Staple food supplementation

- **Current coverage:** 37.4%
- **Maximum feasible coverage:** 100.0%
- **Estimated unit cost:** USD $0.14 (0.06 - 0.25)

Cost-Effectiveness Analysis

The estimated cost per Year of Life Disabled (YLD) averted for each intervention in **Italy** is given below, with interquartile ranges (IQR):

Iron supplementation (antenatal care)

- **Median Cost per YLD:** USD $84849
- **IQR:** USD $58841 – >USD 100k

Iron supplementation (all women of reproductive age)

- **Median Cost per YLD:** >USD 100k
- **IQR:** USD $88326 – >USD 100k

Antenatal preventative IPTp-SP

- **Median Cost per YLD:** >USD 100k
- **IQR:** >USD 100k

Staple food supplementation

- **Median Cost per YLD:** USD $2239
- **IQR:** USD $1510 – $3389

Economic Considerations

Cost-effectiveness thresholds (CET, in USD per DALY averted) in **Italy** is as follows:

- **Estimates from Pichon-Riviere et al. (Lower Bound):** USD $25710
- **Estimates from Pichon-Riviere et al. (Upper Bound):** USD $29931
- **CET equal to per capita GDP:** USD $38373
- **CET equal to 2.3 (LMICs) or 1.7 (HICs) times per capita GDP:** USD $53722

⬆ Back to Table of Contents

Anaemia Reduction in Jamaica

National Target

For our baseline CET = 1 x GDP per capita, the national target for anaemia in **Jamaica** should be a reduction in prevalence of **21%**. This target is estimated with a 95% uncertainty interval from **9%** to **36%**.

For CET based on Pichon-Riviere, the national target for anaemia in **Jamaica** should be a reduction in prevalence of **19%**. This target is estimated with a 95% uncertainty interval from **7%** to **35%**.

For a near-infinite CET, the national target for anaemia in **Jamaica** should be a reduction in prevalence of **21%**. This target is estimated with a 95% uncertainty interval from **10%** to **36%**.

Input parameters

- Among women of reproductive age in **Jamaica**, the prevalence of overall anaemia is **35.5%**
  - **Mild anaemia:** 21.6%
  - **Moderate anaemia:** 13.1%
  - **Severe anaemia:** 0.8%
- There is no data on malaria prevalence; assume 0%.

Intervention coverage and costs

Iron supplementation (antenatal care)

- **Current coverage:** 88.3%
- **Maximum feasible coverage:** 93.3%
- **Estimated unit cost:** USD $9.55 (4.68 - 15.62)

Iron supplementation (all women of reproductive age)

- **Current coverage:** 36.4%
- **Maximum feasible coverage:** 88.3%
- **Estimated unit cost:** USD $5.57 (2.78 - 10.97)

Antenatal preventative IPTp-SP

- **Current coverage:** 0.1%
- **Maximum feasible coverage:** 86.5%
- **Estimated unit cost:** USD $6.81 (3.47 - 11.11)

Staple food supplementation

- **Current coverage:** 41.4%

- **Maximum feasible coverage:** 99.4%
- **Estimated unit cost:** USD $0.57 (0.30 - 1.16)

Cost-Effectiveness Analysis

The estimated cost per Year of Life Disabled (YLD) averted for each intervention in **Jamaica** is given below, with interquartile ranges (IQR):

Iron supplementation (antenatal care)

- **Median Cost per YLD:** USD $2728
- **IQR:** USD $1564 – >USD 100k

Iron supplementation (all women of reproductive age)

- **Median Cost per YLD:** USD $3156
- **IQR:** USD $2162 – $4455

Antenatal preventative IPTp-SP

- **Median Cost per YLD:** >USD 100k
- **IQR:** >USD 100k

Staple food supplementation

- **Median Cost per YLD:** USD $888
- **IQR:** USD $595 – $1356

Economic Considerations

Cost-effectiveness thresholds (CET, in USD per DALY averted) in **Jamaica** is as follows:

- **Estimates from Pichon-Riviere et al. (Lower Bound):** USD $2887
- **Estimates from Pichon-Riviere et al. (Upper Bound):** USD $3918
- **CET equal to per capita GDP:** USD $6874
- **CET equal to 2.3 (LMICs) or 1.7 (HICs) times per capita GDP:** USD $15811

⬆ Back to Table of Contents

Anaemia Reduction in Japan

National Target

For our baseline CET = 1 x GDP per capita, the national target for anaemia in **Japan** should be a reduction in prevalence of **22%**. This target is estimated with a 95% uncertainty interval from **6%** to **41%**.

For CET based on Pichon-Riviere, the national target for anaemia in **Japan** should be a reduction in prevalence of **21%**. This target is estimated with a 95% uncertainty interval from **7%** to **40%**.

For a near-infinite CET, the national target for anaemia in **Japan** should be a reduction in prevalence of **29%**. This target is estimated with a 95% uncertainty interval from **16%** to **46%**.

Input parameters

- Among women of reproductive age in **Japan**, the prevalence of overall anaemia is **15.8%**
    - **Mild anaemia:** 13.8%
    - **Moderate anaemia:** 1.9%
    - **Severe anaemia:** 0.0%
- There is no data on malaria prevalence; assume 0%.

Intervention coverage and costs

Iron supplementation (antenatal care)

- **Current coverage:** 81.0%
- **Maximum feasible coverage:** 100.0%
- **Estimated unit cost:** USD $75.91 (26.96 - 120.42)

Iron supplementation (all women of reproductive age)

- **Current coverage:** 33.4%
- **Maximum feasible coverage:** 100.0%
- **Estimated unit cost:** USD $47.05 (15.44 - 69.73)

Antenatal preventative IPTp-SP

- **Current coverage:** 0.0%
- **Maximum feasible coverage:** 100.0%
- **Estimated unit cost:** USD $27.75 (13.94 - 43.24)

Staple food supplementation

- **Current coverage:** 11.2%
- **Maximum feasible coverage:** 100.0%
- **Estimated unit cost:** USD $0.51 (0.08 - 1.50)

Cost-Effectiveness Analysis

The estimated cost per Year of Life Disabled (YLD) averted for each intervention in **Japan** is given below, with interquartile ranges (IQR):

Iron supplementation (antenatal care)

- **Median Cost per YLD:** USD $72757
- **IQR:** USD $48010 – >USD 100k

Iron supplementation (all women of reproductive age)

- **Median Cost per YLD:** >USD 100k
- **IQR:** USD $73535 – >USD 100k

Antenatal preventative IPTp-SP

- **Median Cost per YLD:** >USD 100k
- **IQR:** >USD 100k

Staple food supplementation

- **Median Cost per YLD:** USD $6743
- **IQR:** USD $4267 – $11281

Economic Considerations

Cost-effectiveness thresholds (CET, in USD per DALY averted) in **Japan** is as follows:

- **Estimates from Pichon-Riviere et al. (Lower Bound):** USD $27744
- **Estimates from Pichon-Riviere et al. (Upper Bound):** USD $32819
- **CET equal to per capita GDP:** USD $33834
- **CET equal to 2.3 (LMICs) or 1.7 (HICs) times per capita GDP:** USD $47368

⬆ Back to Table of Contents

Anaemia Reduction in Jordan

National Target

For our baseline CET = 1 x GDP per capita, the national target for anaemia in **Jordan** should be a reduction in prevalence of **12%**. This target is estimated with a 95% uncertainty interval from **3%** to **30%**.

For CET based on Pichon-Riviere, the national target for anaemia in **Jordan** should be a reduction in prevalence of **11%**. This target is estimated with a 95% uncertainty interval from **3%** to **27%**.

For a near-infinite CET, the national target for anaemia in **Jordan** should be a reduction in prevalence of **19%**. This target is estimated with a 95% uncertainty interval from **10%** to **33%**.

Input parameters

- Among women of reproductive age in **Jordan**, the prevalence of overall anaemia is **41.0%**
  - **Mild anaemia:** 23.8%
  - **Moderate anaemia:** 16.3%
  - **Severe anaemia:** 0.8%
- There is no data on malaria prevalence; assume 0%.

Intervention coverage and costs

Iron supplementation (antenatal care)

- **Current coverage:** 80.9%
- **Maximum feasible coverage:** 97.0%
- **Estimated unit cost:** USD $28.05 (13.91 - 44.02)

Iron supplementation (all women of reproductive age)

- **Current coverage:** 33.4%
- **Maximum feasible coverage:** 97.0%
- **Estimated unit cost:** USD $13.89 (6.93 - 24.20)

Antenatal preventative IPTp-SP

- **Current coverage:** 0.0%
- **Maximum feasible coverage:** 97.0%
- **Estimated unit cost:** USD $20.83 (10.47 - 32.79)

Staple food supplementation

- **Current coverage:** 59.6%
- **Maximum feasible coverage:** 100.0%
- **Estimated unit cost:** USD $0.25 (0.06 - 0.63)

Cost-Effectiveness Analysis

The estimated cost per Year of Life Disabled (YLD) averted for each intervention in **Jordan** is given below, with interquartile ranges (IQR):

Iron supplementation (antenatal care)

- **Median Cost per YLD:** USD $5083
- **IQR:** USD $3666 – $8300

Iron supplementation (all women of reproductive age)

- **Median Cost per YLD:** USD $6310
- **IQR:** USD $4503 – $8960

Antenatal preventative IPTp-SP

- **Median Cost per YLD:** >USD 100k
- **IQR:** >USD 100k

Staple food supplementation

- **Median Cost per YLD:** USD $387
- **IQR:** USD $246 – $586

Economic Considerations

Cost-effectiveness thresholds (CET, in USD per DALY averted) in **Jordan** is as follows:

- **Estimates from Pichon-Riviere et al. (Lower Bound):** USD $2286
- **Estimates from Pichon-Riviere et al. (Upper Bound):** USD $3137
- **CET equal to per capita GDP:** USD $4482
- **CET equal to 2.3 (LMICs) or 1.7 (HICs) times per capita GDP:** USD $10309

⬆ Back to Table of Contents

Anaemia Reduction in Kazakhstan

National Target

For our baseline CET = 1 x GDP per capita, the national target for anaemia in **Kazakhstan** should be a reduction in prevalence of **23%**. This target is estimated with a 95% uncertainty interval from **12%** to **38%**.

For CET based on Pichon-Riviere, the national target for anaemia in **Kazakhstan** should be a reduction in prevalence of **15%**. This target is estimated with a 95% uncertainty interval from **4%** to **34%**.

For a near-infinite CET, the national target for anaemia in **Kazakhstan** should be a reduction in prevalence of **23%**. This target is estimated with a 95% uncertainty interval from **13%** to **37%**.

Input parameters

- Among women of reproductive age in **Kazakhstan**, the prevalence of overall anaemia is **38.7%**
    - **Mild anaemia:** 22.6%
    - **Moderate anaemia:** 15.1%
    - **Severe anaemia:** 1.1%
- There is no data on malaria prevalence; assume 0%.

Intervention coverage and costs

Iron supplementation (antenatal care)

- **Current coverage:** 54.0%
- **Maximum feasible coverage:** 92.9%
- **Estimated unit cost:** USD $12.70 (6.30 - 21.68)

Iron supplementation (all women of reproductive age)

- **Current coverage:** 22.2%
- **Maximum feasible coverage:** 92.9%
- **Estimated unit cost:** USD $8.05 (4.56 - 19.71)

Antenatal preventative IPTp-SP

- **Current coverage:** 0.0%
- **Maximum feasible coverage:** 92.9%
- **Estimated unit cost:** USD $9.62 (4.89 - 16.51)

Staple food supplementation

- **Current coverage:** 55.3%
- **Maximum feasible coverage:** 100.0%
- **Estimated unit cost:** USD $0.13 (0.04 - 0.27)

Cost-Effectiveness Analysis

The estimated cost per Year of Life Disabled (YLD) averted for each intervention in **Kazakhstan** is given below, with interquartile ranges (IQR):

Iron supplementation (antenatal care)

- **Median Cost per YLD:** USD $2265
- **IQR:** USD $1677 – $3097

Iron supplementation (all women of reproductive age)

- **Median Cost per YLD:** USD $4396
- **IQR:** USD $3108 – $6331

Antenatal preventative IPTp-SP

- **Median Cost per YLD:** >USD 100k
- **IQR:** >USD 100k

Staple food supplementation

- **Median Cost per YLD:** USD $165
- **IQR:** USD $112 – $257

Economic Considerations

Cost-effectiveness thresholds (CET, in USD per DALY averted) in **Kazakhstan** is as follows:

- **Estimates from Pichon-Riviere et al. (Lower Bound):** USD $2496
- **Estimates from Pichon-Riviere et al. (Upper Bound):** USD $3416
- **CET equal to per capita GDP:** USD $13137
- **CET equal to 2.3 (LMICs) or 1.7 (HICs) times per capita GDP:** USD $30214

⬆ Back to Table of Contents

Anaemia Reduction in Kenya

National Target

For our baseline CET = 1 x GDP per capita, the national target for anaemia in **Kenya** should be a reduction in prevalence of **16%**. This target is estimated with a 95% uncertainty interval from **1%** to **33%**.

For CET based on Pichon-Riviere, the national target for anaemia in **Kenya** should be a reduction in prevalence of **0%**. This target is estimated with a 95% uncertainty interval from **0%** to **23%**.

For a near-infinite CET, the national target for anaemia in **Kenya** should be a reduction in prevalence of **23%**. This target is estimated with a 95% uncertainty interval from **13%** to **35%**.

Input parameters

- Among women of reproductive age in **Kenya**, the prevalence of overall anaemia is **24.1%**
  - **Mild anaemia:** 14.1%
  - **Moderate anaemia:** 8.1%
  - **Severe anaemia:** 1.9%
- The prevalence of malaria is **3.0%**.

Intervention coverage and costs

Iron supplementation (antenatal care)

- **Current coverage:** 70.5%
- **Maximum feasible coverage:** 92.8%
- **Estimated unit cost:** USD $3.10 (1.35 - 5.54)

Iron supplementation (all women of reproductive age)

- **Current coverage:** 29.1%
- **Maximum feasible coverage:** 92.8%
- **Estimated unit cost:** USD $2.83 (1.41 - 6.59)

Antenatal preventative IPTp-SP

- **Current coverage:** 19.3%
- **Maximum feasible coverage:** 92.8%
- **Estimated unit cost:** USD $1.75 (0.90 - 3.27)

Staple food supplementation

- **Current coverage:** 4.0%
- **Maximum feasible coverage:** 57.2%
- **Estimated unit cost:** USD $0.48 (0.10 - 1.25)

Cost-Effectiveness Analysis

The estimated cost per Year of Life Disabled (YLD) averted for each intervention in **Kenya** is given below, with interquartile ranges (IQR):

Iron supplementation (antenatal care)

- **Median Cost per YLD:** USD $827
- **IQR:** USD $610 – $1149

Iron supplementation (all women of reproductive age)

- **Median Cost per YLD:** USD $2256
- **IQR:** USD $1602 – $3012

Antenatal preventative IPTp-SP

- **Median Cost per YLD:** USD $16724
- **IQR:** USD $12834 – $20912

Staple food supplementation

- **Median Cost per YLD:** USD $1188
- **IQR:** USD $816 – $1748

Economic Considerations

Cost-effectiveness thresholds (CET, in USD per DALY averted) in **Kenya** is as follows:

- **Estimates from Pichon-Riviere et al. (Lower Bound):** USD $312
- **Estimates from Pichon-Riviere et al. (Upper Bound):** USD $624
- **CET equal to per capita GDP:** USD $1950
- **CET equal to 2.3 (LMICs) or 1.7 (HICs) times per capita GDP:** USD $4485

⬆ Back to Table of Contents

Anaemia Reduction in Kiribati

National Target

For our baseline CET = 1 x GDP per capita, the national target for anaemia in **Kiribati** should be a reduction in prevalence of **27%**. This target is estimated with a 95% uncertainty interval from **1%** to **47%**.

For CET based on Pichon-Riviere, the national target for anaemia in **Kiribati** should be a reduction in prevalence of **27%**. This target is estimated with a 95% uncertainty interval from **1%** to **46%**.

For a near-infinite CET, the national target for anaemia in **Kiribati** should be a reduction in prevalence of **29%**. This target is estimated with a 95% uncertainty interval from **14%** to **47%**.

Input parameters

- Among women of reproductive age in **Kiribati**, the prevalence of overall anaemia is **46.8%**
  - **Mild anaemia:** 26.6%
  - **Moderate anaemia:** 18.8%
  - **Severe anaemia:** 1.4%
- There is no data on malaria prevalence; assume 0%.

Intervention coverage and costs

Iron supplementation (antenatal care)

- **Current coverage:** 84.2%
- **Maximum feasible coverage:** 89.2%
- **Estimated unit cost:** USD $2.20 (1.00 - 4.37)

Iron supplementation (all women of reproductive age)

- **Current coverage:** 34.7%
- **Maximum feasible coverage:** 84.2%
- **Estimated unit cost:** USD $3.14 (1.59 - 7.43)

Antenatal preventative IPTp-SP

- **Current coverage:** 35.0%
- **Maximum feasible coverage:** 67.0%
- **Estimated unit cost:** USD $1.14 (0.65 - 2.48)

Staple food supplementation

- **Current coverage:** 3.2%

- **Maximum feasible coverage:** 100.0%
- **Estimated unit cost:** USD $0.54 (0.08 - 1.67)

Cost-Effectiveness Analysis

The estimated cost per Year of Life Disabled (YLD) averted for each intervention in **Kiribati** is given below, with interquartile ranges (IQR):

Iron supplementation (antenatal care)

- **Median Cost per YLD:** USD $494
- **IQR:** USD $282 – >USD 100k

Iron supplementation (all women of reproductive age)

- **Median Cost per YLD:** USD $1316
- **IQR:** USD $914 – $1963

Antenatal preventative IPTp-SP

- **Median Cost per YLD:** >USD 100k
- **IQR:** >USD 100k

Staple food supplementation

- **Median Cost per YLD:** USD $776
- **IQR:** USD $474 – $1227

Economic Considerations

Cost-effectiveness thresholds (CET, in USD per DALY averted) in **Kiribati** is as follows:

- **Estimates from Pichon-Riviere et al. (Lower Bound):** USD $1024
- **Estimates from Pichon-Riviere et al. (Upper Bound):** USD $2006
- **CET equal to per capita GDP:** USD $2090
- **CET equal to 2.3 (LMICs) or 1.7 (HICs) times per capita GDP:** USD $4807

⬆ Back to Table of Contents

Anaemia Reduction in Kuwait

National Target

For our baseline CET = 1 x GDP per capita, the national target for anaemia in **Kuwait** should be a reduction in prevalence of **17%**. This target is estimated with a 95% uncertainty interval from **4%** to **32%**.

For CET based on Pichon-Riviere, the national target for anaemia in **Kuwait** should be a reduction in prevalence of **11%**. This target is estimated with a 95% uncertainty interval from **2%** to **27%**.

For a near-infinite CET, the national target for anaemia in **Kuwait** should be a reduction in prevalence of **19%**. This target is estimated with a 95% uncertainty interval from **9%** to **32%**.

Input parameters

- Among women of reproductive age in **Kuwait**, the prevalence of overall anaemia is **27.1%**
  - **Mild anaemia:** 17.9%
  - **Moderate anaemia:** 8.7%
  - **Severe anaemia:** 0.5%
- There is no data on malaria prevalence; assume 0%.

Intervention coverage and costs

Iron supplementation (antenatal care)

- **Current coverage:** 81.0%
- **Maximum feasible coverage:** 100.0%
- **Estimated unit cost:** USD $60.85 (19.55 - 96.51)

Iron supplementation (all women of reproductive age)

- **Current coverage:** 33.4%
- **Maximum feasible coverage:** 100.0%
- **Estimated unit cost:** USD $36.44 (12.57 - 68.53)

Antenatal preventative IPTp-SP

- **Current coverage:** 0.0%
- **Maximum feasible coverage:** 100.0%
- **Estimated unit cost:** USD $16.65 (8.39 - 26.59)

Staple food supplementation

- **Current coverage:** 62.4%
- **Maximum feasible coverage:** 100.0%
- **Estimated unit cost:** USD $0.41 (0.10 - 1.09)

Cost-Effectiveness Analysis

The estimated cost per Year of Life Disabled (YLD) averted for each intervention in **Kuwait** is given below, with interquartile ranges (IQR):

Iron supplementation (antenatal care)

- **Median Cost per YLD:** USD $16992
- **IQR:** USD $11660 – $27192

Iron supplementation (all women of reproductive age)

- **Median Cost per YLD:** USD $28291
- **IQR:** USD $18899 – $41210

Antenatal preventative IPTp-SP

- **Median Cost per YLD:** >USD 100k
- **IQR:** >USD 100k

Staple food supplementation

- **Median Cost per YLD:** USD $863
- **IQR:** USD $525 – $1335

Economic Considerations

Cost-effectiveness thresholds (CET, in USD per DALY averted) in **Kuwait** is as follows:

- **Estimates from Pichon-Riviere et al. (Lower Bound):** USD $13137
- **Estimates from Pichon-Riviere et al. (Upper Bound):** USD $15389
- **CET equal to per capita GDP:** USD $37533
- **CET equal to 2.3 (LMICs) or 1.7 (HICs) times per capita GDP:** USD $52547

⬆ Back to Table of Contents

Anaemia Reduction in Kyrgyzstan

National Target

For our baseline CET = 1 x GDP per capita, the national target for anaemia in **Kyrgyzstan** should be a reduction in prevalence of **26%.** This target is estimated with a 95% uncertainty interval from **11%** to **47%**.

For CET based on Pichon-Riviere, the national target for anaemia in **Kyrgyzstan** should be a reduction in prevalence of **23%**. This target is estimated with a 95% uncertainty interval from **0%** to **42%**.

For a near-infinite CET, the national target for anaemia in **Kyrgyzstan** should be a reduction in prevalence of **34%**. This target is estimated with a 95% uncertainty interval from **19%** to **51%**.

Input parameters

- Among women of reproductive age in **Kyrgyzstan**, the prevalence of overall anaemia is **36.5%**
  - **Mild anaemia:** 19.5%
  - **Moderate anaemia:** 15.8%
  - **Severe anaemia:** 1.2%
- The prevalence of malaria is **0.0%**.

Intervention coverage and costs

Iron supplementation (antenatal care)

- **Current coverage:** NaN%
- **Maximum feasible coverage:** NaN%

- **Estimated unit cost:** USD $5.78 (2.81 - 10.89)

Iron supplementation (all women of reproductive age)

- **Current coverage:** NaN%
- **Maximum feasible coverage:** NaN%
- **Estimated unit cost:** USD $4.91 (2.49 - 11.23)

Antenatal preventative IPTp-SP

- **Current coverage:** NaN%
- **Maximum feasible coverage:** NaN%
- **Estimated unit cost:** USD $4.39 (2.27 - 8.44)

Staple food supplementation

- **Current coverage:** NaN%
- **Maximum feasible coverage:** NaN%
- **Estimated unit cost:** USD $0.10 (0.03 - 0.36)

Cost-Effectiveness Analysis

The estimated cost per Year of Life Disabled (YLD) averted for each intervention in **Kyrgyzstan** is given below, with interquartile ranges (IQR):

Iron supplementation (antenatal care)

- **Median Cost per YLD:** USD $1185
- **IQR:** USD $899 – $1600

Iron supplementation (all women of reproductive age)

- **Median Cost per YLD:** USD $2705
- **IQR:** USD $1960 – $3901

Antenatal preventative IPTp-SP

- **Median Cost per YLD:** >USD 100k
- **IQR:** >USD 100k

Staple food supplementation

- **Median Cost per YLD:** USD $229
- **IQR:** USD $150 – $343

Economic Considerations

Cost-effectiveness thresholds (CET, in USD per DALY averted) in **Kyrgyzstan** is as follows:

- **Estimates from Pichon-Riviere et al. (Lower Bound):** USD $335
- **Estimates from Pichon-Riviere et al. (Upper Bound):** USD $650
- **CET equal to per capita GDP:** USD $1970
- **CET equal to 2.3 (LMICs) or 1.7 (HICs) times per capita GDP:** USD $4531

⬆ Back to Table of Contents

Anaemia Reduction in Laos

National Target

For our baseline CET = 1 x GDP per capita, the national target for anaemia in **Laos** should be a reduction in prevalence of **9%**. This target is estimated with a 95% uncertainty interval from **0%** to **20%**.

For CET based on Pichon-Riviere, the national target for anaemia in **Laos** should be a reduction in prevalence of **0%**. This target is estimated with a 95% uncertainty interval from **0%** to **6%**.

For a near-infinite CET, the national target for anaemia in **Laos** should be a reduction in prevalence of **12%**. This target is estimated with a 95% uncertainty interval from **6%** to **20%**.

Input parameters

- Among women of reproductive age in **Laos**, the prevalence of overall anaemia is **48.2%**
  - **Mild anaemia:** 31.8%
  - **Moderate anaemia:** 16.0%
  - **Severe anaemia:** 0.4%
- The prevalence of malaria is **0.0%**.

Intervention coverage and costs

Iron supplementation (antenatal care)

- **Current coverage:** NaN%
- **Maximum feasible coverage:** NaN%
- **Estimated unit cost:** USD $2.71 (1.28 - 5.29)

Iron supplementation (all women of reproductive age)

- **Current coverage:** NaN%
- **Maximum feasible coverage:** NaN%
- **Estimated unit cost:** USD $2.45 (1.27 - 6.51)

Antenatal preventative IPTp-SP

- **Current coverage:** NaN%
- **Maximum feasible coverage:** NaN%
- **Estimated unit cost:** USD $1.51 (0.83 - 3.03)

Staple food supplementation

- **Current coverage:** NaN%
- **Maximum feasible coverage:** NaN%
- **Estimated unit cost:** USD $0.73 (0.11 - 2.24)

Cost-Effectiveness Analysis

The estimated cost per Year of Life Disabled (YLD) averted for each intervention in **Laos** is given below, with interquartile ranges (IQR):

Iron supplementation (antenatal care)

- **Median Cost per YLD:** USD $761
- **IQR:** USD $452 – >USD 100k

Iron supplementation (all women of reproductive age)

- **Median Cost per YLD:** USD $1585
- **IQR:** USD $1090 – $2234

Antenatal preventative IPTp-SP

- **Median Cost per YLD:** >USD 100k
- **IQR:** >USD 100k

Staple food supplementation

- **Median Cost per YLD:** USD $1396
- **IQR:** USD $877 – $2063

Economic Considerations

Cost-effectiveness thresholds (CET, in USD per DALY averted) in **Laos** is as follows:

- **Estimates from Pichon-Riviere et al. (Lower Bound):** USD $208
- **Estimates from Pichon-Riviere et al. (Upper Bound):** USD $394
- **CET equal to per capita GDP:** USD $2075
- **CET equal to 2.3 (LMICs) or 1.7 (HICs) times per capita GDP:** USD $4773

⬆ Back to Table of Contents

Anaemia Reduction in Latvia

National Target

For our baseline CET = 1 x GDP per capita, the national target for anaemia in **Latvia** should be a reduction in prevalence of **18%**. This target is estimated with a 95% uncertainty interval from **7%** to **36%**.

For CET based on Pichon-Riviere, the national target for anaemia in **Latvia** should be a reduction in prevalence of **16%**. This target is estimated with a 95% uncertainty interval from **6%** to **33%**.

For a near-infinite CET, the national target for anaemia in **Latvia** should be a reduction in prevalence of **24%**. This target is estimated with a 95% uncertainty interval from **14%** to **40%**.

Input parameters

- Among women of reproductive age in **Latvia**, the prevalence of overall anaemia is **20.8%**
  - **Mild anaemia:** 12.6%
  - **Moderate anaemia:** 7.6%
  - **Severe anaemia:** 0.6%
- There is no data on malaria prevalence; assume 0%.

Intervention coverage and costs

Iron supplementation (antenatal care)

- **Current coverage:** 81.0%
- **Maximum feasible coverage:** 100.0%
- **Estimated unit cost:** USD $62.64 (20.46 - 100.96)

Iron supplementation (all women of reproductive age)

- **Current coverage:** 33.4%
- **Maximum feasible coverage:** 100.0%
- **Estimated unit cost:** USD $40.08 (14.36 - 75.97)

Antenatal preventative IPTp-SP

- **Current coverage:** 0.0%
- **Maximum feasible coverage:** 100.0%
- **Estimated unit cost:** USD $19.47 (9.80 - 32.75)

Staple food supplementation

- **Current coverage:** 37.1%
- **Maximum feasible coverage:** 100.0%
- **Estimated unit cost:** USD $0.15 (0.05 - 0.30)

Cost-Effectiveness Analysis

The estimated cost per Year of Life Disabled (YLD) averted for each intervention in **Latvia** is given below, with interquartile ranges (IQR):

Iron supplementation (antenatal care)

- **Median Cost per YLD:** USD $20365
- **IQR:** USD $14209 – $32530

Iron supplementation (all women of reproductive age)

- **Median Cost per YLD:** USD $35689
- **IQR:** USD $25066 – $51761

Antenatal preventative IPTp-SP

- **Median Cost per YLD:** >USD 100k
- **IQR:** >USD 100k

Staple food supplementation

- **Median Cost per YLD:** USD $504
- **IQR:** USD $342 – $771

Economic Considerations

Cost-effectiveness thresholds (CET, in USD per DALY averted) in **Latvia** is as follows:

- **Estimates from Pichon-Riviere et al. (Lower Bound):** USD $10201
- **Estimates from Pichon-Riviere et al. (Upper Bound):** USD $12056
- **CET equal to per capita GDP:** USD $23184
- **CET equal to 2.3 (LMICs) or 1.7 (HICs) times per capita GDP:** USD $32458

⬆ Back to Table of Contents

Anaemia Reduction in Lebanon

National Target

For our baseline CET = 1 x GDP per capita, the national target for anaemia in **Lebanon** should be a reduction in prevalence of **6%**. This target is estimated with a 95% uncertainty interval from **0%** to **18%**.

For CET based on Pichon-Riviere, the national target for anaemia in **Lebanon** should be a reduction in prevalence of **5%**. This target is estimated with a 95% uncertainty interval from **0%** to **16%**.

For a near-infinite CET, the national target for anaemia in **Lebanon** should be a reduction in prevalence of **13%**. This target is estimated with a 95% uncertainty interval from **5%** to **25%**.

Input parameters

- Among women of reproductive age in **Lebanon**, the prevalence of overall anaemia is **24.1%**
  - **Mild anaemia:** 12.1%
  - **Moderate anaemia:** 10.7%
  - **Severe anaemia:** 1.3%
- There is no data on malaria prevalence; assume 0%.

Intervention coverage and costs

Iron supplementation (antenatal care)

- **Current coverage:** 58.4%
- **Maximum feasible coverage:** 76.8%
- **Estimated unit cost:** USD $85.57 (42.61 - 129.20)

Iron supplementation (all women of reproductive age)

- **Current coverage:** 24.1%
- **Maximum feasible coverage:** 76.8%
- **Estimated unit cost:** USD $56.51 (28.24 - 87.36)

Antenatal preventative IPTp-SP

- **Current coverage:** 0.0%
- **Maximum feasible coverage:** 76.8%
- **Estimated unit cost:** USD $63.62 (31.86 - 96.14)

Staple food supplementation

- **Current coverage:** 77.7%

- **Maximum feasible coverage:** 100.0%
- **Estimated unit cost:** USD $0.13 (0.02 - 0.39)

Cost-Effectiveness Analysis

The estimated cost per Year of Life Disabled (YLD) averted for each intervention in **Lebanon** is given below, with interquartile ranges (IQR):

Iron supplementation (antenatal care)

- **Median Cost per YLD:** USD $22031
- **IQR:** USD $15892 – $31775

Iron supplementation (all women of reproductive age)

- **Median Cost per YLD:** USD $33786
- **IQR:** USD $24409 – $47373

Antenatal preventative IPTp-SP

- **Median Cost per YLD:** >USD 100k
- **IQR:** >USD 100k

Staple food supplementation

- **Median Cost per YLD:** USD $266
- **IQR:** USD $162 – $481

Economic Considerations

Cost-effectiveness thresholds (CET, in USD per DALY averted) in **Lebanon** is as follows:

- **Estimates from Pichon-Riviere et al. (Lower Bound):** USD $2813
- **Estimates from Pichon-Riviere et al. (Upper Bound):** USD $3888
- **CET equal to per capita GDP:** USD $4136
- **CET equal to 2.3 (LMICs) or 1.7 (HICs) times per capita GDP:** USD $9513

⬆ Back to Table of Contents

Anaemia Reduction in Lesotho

National Target

For our baseline CET = 1 x GDP per capita, the national target for anaemia in **Lesotho** should be a reduction in prevalence of **15%**. This target is estimated with a 95% uncertainty interval from **0%** to **36%**.

For CET based on Pichon-Riviere, the national target for anaemia in **Lesotho** should be a reduction in prevalence of **1%**. This target is estimated with a 95% uncertainty interval from **0%** to **34%**.

For a near-infinite CET, the national target for anaemia in **Lesotho** should be a reduction in prevalence of **23%**. This target is estimated with a 95% uncertainty interval from **12%** to **39%**.

Input parameters

- Among women of reproductive age in **Lesotho**, the prevalence of overall anaemia is **28.7%**
    - **Mild anaemia:** 14.6%
    - **Moderate anaemia:** 12.8%
    - **Severe anaemia:** 1.3%
- There is no data on malaria prevalence; assume 0%.

Intervention coverage and costs

Iron supplementation (antenatal care)

- **Current coverage:** 76.9%
- **Maximum feasible coverage:** 96.0%
- **Estimated unit cost:** USD $2.96 (1.25 - 5.14)

Iron supplementation (all women of reproductive age)

- **Current coverage:** 31.7%
- **Maximum feasible coverage:** 96.0%
- **Estimated unit cost:** USD $2.50 (1.23 - 5.95)

Antenatal preventative IPTp-SP

- **Current coverage:** 38.2%
- **Maximum feasible coverage:** 96.0%
- **Estimated unit cost:** USD $1.62 (0.84 - 3.05)

Staple food supplementation

- **Current coverage:** 5.0%
- **Maximum feasible coverage:** 76.0%
- **Estimated unit cost:** USD $0.31 (0.11 - 1.17)

Cost-Effectiveness Analysis

The estimated cost per Year of Life Disabled (YLD) averted for each intervention in **Lesotho** is given below, with interquartile ranges (IQR):

Iron supplementation (antenatal care)

- **Median Cost per YLD:** >USD 100k
- **IQR:** USD $612 – >USD 100k

Iron supplementation (all women of reproductive age)

- **Median Cost per YLD:** USD $1610
- **IQR:** USD $1085 – $2333

Antenatal preventative IPTp-SP

- **Median Cost per YLD:** >USD 100k
- **IQR:** >USD 100k

Staple food supplementation

- **Median Cost per YLD:** USD $766
- **IQR:** USD $495 – $1158

Economic Considerations

Cost-effectiveness thresholds (CET, in USD per DALY averted) in **Lesotho** is as follows:

- **Estimates from Pichon-Riviere et al. (Lower Bound):** USD $307
- **Estimates from Pichon-Riviere et al. (Upper Bound):** USD $597
- **CET equal to per capita GDP:** USD $878
- **CET equal to 2.3 (LMICs) or 1.7 (HICs) times per capita GDP:** USD $2019

⬆ Back to Table of Contents

Anaemia Reduction in Liberia

National Target

For our baseline CET = 1 x GDP per capita, the national target for anaemia in **Liberia** should be a reduction in prevalence of **24%**. This target is estimated with a 95% uncertainty interval from **4%** to **40%**.

For CET based on Pichon-Riviere, the national target for anaemia in **Liberia** should be a reduction in prevalence of **10%**. This target is estimated with a 95% uncertainty interval from **0%** to **36%**.

For a near-infinite CET, the national target for anaemia in **Liberia** should be a reduction in prevalence of **24%**. This target is estimated with a 95% uncertainty interval from **13%** to **39%**.

Input parameters

- Among women of reproductive age in **Liberia**, the prevalence of overall anaemia is **71.9%**
  - **Mild anaemia:** 20.8%
  - **Moderate anaemia:** 43.7%
  - **Severe anaemia:** 7.5%
- The prevalence of malaria is **40.1%**.

Intervention coverage and costs

Iron supplementation (antenatal care)

- **Current coverage:** 93.2%
- **Maximum feasible coverage:** 97.8%

- **Estimated unit cost:** USD $2.06 (0.81 - 3.70)

Iron supplementation (all women of reproductive age)

- **Current coverage:** 38.4%
- **Maximum feasible coverage:** 97.8%
- **Estimated unit cost:** USD $1.81 (0.89 - 4.88)

Antenatal preventative IPTp-SP

- **Current coverage:** 79.5%
- **Maximum feasible coverage:** 97.8%
- **Estimated unit cost:** USD $0.88 (0.47 - 1.79)

Staple food supplementation

- **Current coverage:** 0.9%
- **Maximum feasible coverage:** 70.1%
- **Estimated unit cost:** USD $0.72 (0.10 - 2.19)

Cost-Effectiveness Analysis

The estimated cost per Year of Life Disabled (YLD) averted for each intervention in **Liberia** is given below, with interquartile ranges (IQR):

Iron supplementation (antenatal care)

- **Median Cost per YLD:** USD $208
- **IQR:** USD $92 – >USD 100k

Iron supplementation (all women of reproductive age)

- **Median Cost per YLD:** USD $308
- **IQR:** USD $204 – $458

Antenatal preventative IPTp-SP

- **Median Cost per YLD:** USD $219
- **IQR:** USD $155 – $350

Staple food supplementation

- **Median Cost per YLD:** USD $393
- **IQR:** USD $243 – $603

Economic Considerations

Cost-effectiveness thresholds (CET, in USD per DALY averted) in **Liberia** is as follows:

- **Estimates from Pichon-Riviere et al. (Lower Bound):** USD $88
- **Estimates from Pichon-Riviere et al. (Upper Bound):** USD $296
- **CET equal to per capita GDP:** USD $800
- **CET equal to 2.3 (LMICs) or 1.7 (HICs) times per capita GDP:** USD $1839

⬆ Back to Table of Contents

Anaemia Reduction in Libya

National Target

For our baseline CET = 1 x GDP per capita, the national target for anaemia in **Libya** should be a reduction in prevalence of **13%**. This target is estimated with a 95% uncertainty interval from **3%** to **27%**.

For CET based on Pichon-Riviere, the national target for anaemia in **Libya** should be a reduction in prevalence of **10%**. This target is estimated with a 95% uncertainty interval from **1%** to **26%**.

For a near-infinite CET, the national target for anaemia in **Libya** should be a reduction in prevalence of **14%**. This target is estimated with a 95% uncertainty interval from **5%** to **27%**.

Input parameters

- Among women of reproductive age in **Libya**, the prevalence of overall anaemia is **32.8%**
  - **Mild anaemia:** 20.3%
  - **Moderate anaemia:** 11.8%
  - **Severe anaemia:** 0.7%
- There is no data on malaria prevalence; assume 0%.

Intervention coverage and costs

Iron supplementation (antenatal care)

- **Current coverage:** 80.9%
- **Maximum feasible coverage:** 82.5%
- **Estimated unit cost:** USD $9.74 (4.82 - 16.93)

Iron supplementation (all women of reproductive age)

- **Current coverage:** 33.4%
- **Maximum feasible coverage:** 82.5%
- **Estimated unit cost:** USD $6.58 (3.71 - 16.66)

Antenatal preventative IPTp-SP

- **Current coverage:** 0.0%
- **Maximum feasible coverage:** 82.5%
- **Estimated unit cost:** USD $6.98 (3.54 - 12.00)

Staple food supplementation

- **Current coverage:** 70.4%
- **Maximum feasible coverage:** 100.0%
- **Estimated unit cost:** USD $0.15 (0.03 - 0.33)

Cost-Effectiveness Analysis

The estimated cost per Year of Life Disabled (YLD) averted for each intervention in **Libya** is given below, with interquartile ranges (IQR):

Iron supplementation (antenatal care)

- **Median Cost per YLD:** USD $4217
- **IQR:** USD $2034 – >USD 100k

Iron supplementation (all women of reproductive age)

- **Median Cost per YLD:** USD $4672
- **IQR:** USD $3262 – $7050

Antenatal preventative IPTp-SP

- **Median Cost per YLD:** >USD 100k
- **IQR:** >USD 100k

Staple food supplementation

- **Median Cost per YLD:** USD $219
- **IQR:** USD $137 – $345

Economic Considerations

Cost-effectiveness thresholds (CET, in USD per DALY averted) in **Libya** is as follows:

- **Estimates from Pichon-Riviere et al. (Lower Bound):** USD $806
- **Estimates from Pichon-Riviere et al. (Upper Bound):** USD $4251
- **CET equal to per capita GDP:** USD $7330
- **CET equal to 2.3 (LMICs) or 1.7 (HICs) times per capita GDP:** USD $16859

⬆ Back to Table of Contents

Anaemia Reduction in Lithuania

National Target

For our baseline CET = 1 x GDP per capita, the national target for anaemia in **Lithuania** should be a reduction in prevalence of **19%**. This target is estimated with a 95% uncertainty interval from **6%** to **37%**.

For CET based on Pichon-Riviere, the national target for anaemia in **Lithuania** should be a reduction in prevalence of **16%**. This target is estimated with a 95% uncertainty interval from **6%** to **35%**.

For a near-infinite CET, the national target for anaemia in **Lithuania** should be a reduction in prevalence of **24%**. This target is estimated with a 95% uncertainty interval from **13%** to **38%**.

Input parameters

- Among women of reproductive age in **Lithuania**, the prevalence of overall anaemia is **20.6%**
  - **Mild anaemia:** 12.6%
  - **Moderate anaemia:** 7.4%
  - **Severe anaemia:** 0.5%
- There is no data on malaria prevalence; assume 0%.

Intervention coverage and costs

Iron supplementation (antenatal care)

- **Current coverage:** 81.0%
- **Maximum feasible coverage:** 100.0%
- **Estimated unit cost:** USD $65.42 (21.69 - 104.48)

Iron supplementation (all women of reproductive age)

- **Current coverage:** 33.4%
- **Maximum feasible coverage:** 100.0%
- **Estimated unit cost:** USD $40.05 (11.96 - 58.43)

Antenatal preventative IPTp-SP

- **Current coverage:** 0.0%
- **Maximum feasible coverage:** 100.0%
- **Estimated unit cost:** USD $19.65 (9.89 - 30.58)

Staple food supplementation

- **Current coverage:** 37.9%
- **Maximum feasible coverage:** 100.0%
- **Estimated unit cost:** USD $0.12 (0.04 - 0.19)

Cost-Effectiveness Analysis

The estimated cost per Year of Life Disabled (YLD) averted for each intervention in **Lithuania** is given below, with interquartile ranges (IQR):

Iron supplementation (antenatal care)

- **Median Cost per YLD:** USD $21378
- **IQR:** USD $14746 – $34482

Iron supplementation (all women of reproductive age)

- **Median Cost per YLD:** USD $30130
- **IQR:** USD $21144 – $43031

Antenatal preventative IPTp-SP

- **Median Cost per YLD:** >USD 100k
- **IQR:** >USD 100k

Staple food supplementation

- **Median Cost per YLD:** USD $348
- **IQR:** USD $240 – $520

Economic Considerations

Cost-effectiveness thresholds (CET, in USD per DALY averted) in **Lithuania** is as follows:

- **Estimates from Pichon-Riviere et al. (Lower Bound):** USD $13280
- **Estimates from Pichon-Riviere et al. (Upper Bound):** USD $15449
- **CET equal to per capita GDP:** USD $27103
- **CET equal to 2.3 (LMICs) or 1.7 (HICs) times per capita GDP:** USD $37944

⬆ Back to Table of Contents

Anaemia Reduction in Luxembourg

National Target

For our baseline CET = 1 x GDP per capita, the national target for anaemia in **Luxembourg** should be a reduction in prevalence of **19%**. This target is estimated with a 95% uncertainty interval from **7%** to **38%**.

For CET based on Pichon-Riviere, the national target for anaemia in **Luxembourg** should be a reduction in prevalence of **16%**. This target is estimated with a 95% uncertainty interval from **6%** to **34%**.

For a near-infinite CET, the national target for anaemia in **Luxembourg** should be a reduction in prevalence of **25%**. This target is estimated with a 95% uncertainty interval from **13%** to **40%**.

Input parameters

- Among women of reproductive age in **Luxembourg**, the prevalence of overall anaemia is **7.6%**
    - **Mild anaemia:** 6.3%
    - **Moderate anaemia:** 1.3%
    - **Severe anaemia:** 0.0%
- There is no data on malaria prevalence; assume 0%.

Intervention coverage and costs

Iron supplementation (antenatal care)

- **Current coverage:** 81.0%
- **Maximum feasible coverage:** 100.0%
- **Estimated unit cost:** USD $61.13 (19.42 - 101.20)

Iron supplementation (all women of reproductive age)

- **Current coverage:** 33.4%
- **Maximum feasible coverage:** 100.0%
- **Estimated unit cost:** USD $36.92 (10.48 - 54.07)

Antenatal preventative IPTp-SP

- **Current coverage:** 0.0%
- **Maximum feasible coverage:** 100.0%

- **Estimated unit cost:** USD $17.07 (8.60 - 28.43)

Staple food supplementation

- **Current coverage:** 36.0%
- **Maximum feasible coverage:** 100.0%
- **Estimated unit cost:** USD $0.20 (0.07 - 0.43)

Cost-Effectiveness Analysis

The estimated cost per Year of Life Disabled (YLD) averted for each intervention in **Luxembourg** is given below, with interquartile ranges (IQR):

Iron supplementation (antenatal care)

- **Median Cost per YLD:** >USD 100k
- **IQR:** USD $78917 – >USD 100k

Iron supplementation (all women of reproductive age)

- **Median Cost per YLD:** >USD 100k
- **IQR:** >USD 100k

Antenatal preventative IPTp-SP

- **Median Cost per YLD:** >USD 100k
- **IQR:** >USD 100k

Staple food supplementation

- **Median Cost per YLD:** USD $3606
- **IQR:** USD $2276 – $5992

Economic Considerations

Cost-effectiveness thresholds (CET, in USD per DALY averted) in **Luxembourg** is as follows:

- **Estimates from Pichon-Riviere et al. (Lower Bound):** USD $53869
- **Estimates from Pichon-Riviere et al. (Upper Bound):** USD $62847

- **CET equal to per capita GDP:** USD $128259
- **CET equal to 2.3 (LMICs) or 1.7 (HICs) times per capita GDP:** USD $179563

⬆ Back to Table of Contents

Anaemia Reduction in Madagascar

National Target

For our baseline CET = 1 x GDP per capita, the national target for anaemia in **Madagascar** should be a reduction in prevalence of **1%**. This target is estimated with a 95% uncertainty interval from **0%** to **17%**.

For CET based on Pichon-Riviere, the national target for anaemia in **Madagascar** should be a reduction in prevalence of **0%**. This target is estimated with a 95% uncertainty interval from **0%** to **0%**.

For a near-infinite CET, the national target for anaemia in **Madagascar** should be a reduction in prevalence of **16%**. This target is estimated with a 95% uncertainty interval from **9%** to **25%**.

Input parameters

- Among women of reproductive age in **Madagascar**, the prevalence of overall anaemia is **36.1%**
  - **Mild anaemia:** 19.4%
  - **Moderate anaemia:** 15.4%
  - **Severe anaemia:** 1.3%
- The prevalence of malaria is **5.9%**.

Intervention coverage and costs

Iron supplementation (antenatal care)

- **Current coverage:** 72.9%
- **Maximum feasible coverage:** 87.3%
- **Estimated unit cost:** USD $2.19 (0.90 - 4.09)

Iron supplementation (all women of reproductive age)

- **Current coverage:** 30.1%
- **Maximum feasible coverage:** 87.3%
- **Estimated unit cost:** USD $1.99 (1.00 - 4.50)

Antenatal preventative IPTp-SP

- **Current coverage:** 40.2%
- **Maximum feasible coverage:** 87.3%
- **Estimated unit cost:** USD $1.13 (0.59 - 2.27)

Staple food supplementation

- **Current coverage:** 0.8%
- **Maximum feasible coverage:** 26.3%
- **Estimated unit cost:** USD $0.69 (0.10 - 2.11)

Cost-Effectiveness Analysis

The estimated cost per Year of Life Disabled (YLD) averted for each intervention in **Madagascar** is given below, with interquartile ranges (IQR):

Iron supplementation (antenatal care)

- **Median Cost per YLD:** USD $416
- **IQR:** USD $286 – $658

Iron supplementation (all women of reproductive age)

- **Median Cost per YLD:** USD $1051
- **IQR:** USD $733 – $1501

Antenatal preventative IPTp-SP

- **Median Cost per YLD:** USD $3616
- **IQR:** USD $2726 – $4941

Staple food supplementation

- **Median Cost per YLD:** USD $1281

- **IQR:** USD $801 – $2078

Economic Considerations

Cost-effectiveness thresholds (CET, in USD per DALY averted) in **Madagascar** is as follows:

- **Estimates from Pichon-Riviere et al. (Lower Bound):** USD $32
- **Estimates from Pichon-Riviere et al. (Upper Bound):** USD $95
- **CET equal to per capita GDP:** USD $529
- **CET equal to 2.3 (LMICs) or 1.7 (HICs) times per capita GDP:** USD $1216

⬆ Back to Table of Contents

Anaemia Reduction in Malawi

National Target

For our baseline CET = 1 x GDP per capita, the national target for anaemia in **Malawi** should be a reduction in prevalence of **4%**. This target is estimated with a 95% uncertainty interval from **0%** to **17%**.

For CET based on Pichon-Riviere, the national target for anaemia in **Malawi** should be a reduction in prevalence of **0%**. This target is estimated with a 95% uncertainty interval from **0%** to **1%**.

For a near-infinite CET, the national target for anaemia in **Malawi** should be a reduction in prevalence of **12%**. This target is estimated with a 95% uncertainty interval from **6%** to **20%**.

Input parameters

- Among women of reproductive age in **Malawi**, the prevalence of overall anaemia is **59.7%**
  - **Mild anaemia:** 42.6%
  - **Moderate anaemia:** 16.8%
  - **Severe anaemia:** 0.3%
- The prevalence of malaria is **18.5%**.

Intervention coverage and costs

Iron supplementation (antenatal care)

- **Current coverage:** 89.1%
- **Maximum feasible coverage:** 98.0%
- **Estimated unit cost:** USD $2.85 (1.22 - 5.40)

Iron supplementation (all women of reproductive age)

- **Current coverage:** 36.7%
- **Maximum feasible coverage:** 98.0%
- **Estimated unit cost:** USD $2.84 (1.44 - 6.47)

Antenatal preventative IPTp-SP

- **Current coverage:** 75.8%
- **Maximum feasible coverage:** 98.0%
- **Estimated unit cost:** USD $1.59 (0.82 - 3.30)

Staple food supplementation

- **Current coverage:** 0.7%
- **Maximum feasible coverage:** 17.8%
- **Estimated unit cost:** USD $0.31 (0.12 - 1.30)

Cost-Effectiveness Analysis

The estimated cost per Year of Life Disabled (YLD) averted for each intervention in **Malawi** is given below, with interquartile ranges (IQR):

Iron supplementation (antenatal care)

- **Median Cost per YLD:** USD $824
- **IQR:** USD $369 – >USD 100k

Iron supplementation (all women of reproductive age)

- **Median Cost per YLD:** USD $1283
- **IQR:** USD $888 – $1901

Antenatal preventative IPTp-SP

- **Median Cost per YLD:** USD $2293
- **IQR:** USD $1397 – >USD 100k

Staple food supplementation

- **Median Cost per YLD:** USD $641
- **IQR:** USD $389 – $1008

Economic Considerations

Cost-effectiveness thresholds (CET, in USD per DALY averted) in **Malawi** is as follows:

- **Estimates from Pichon-Riviere et al. (Lower Bound):** USD $47
- **Estimates from Pichon-Riviere et al. (Upper Bound):** USD $155
- **CET equal to per capita GDP:** USD $673
- **CET equal to 2.3 (LMICs) or 1.7 (HICs) times per capita GDP:** USD $1548

⬆ Back to Table of Contents

Anaemia Reduction in Malaysia

National Target

For our baseline CET = 1 x GDP per capita, the national target for anaemia in **Malaysia** should be a reduction in prevalence of **28%**. This target is estimated with a 95% uncertainty interval from **15%** to **47%**.

For CET based on Pichon-Riviere, the national target for anaemia in **Malaysia** should be a reduction in prevalence of **25%**. This target is estimated with a 95% uncertainty interval from **10%** to **45%**.

For a near-infinite CET, the national target for anaemia in **Malaysia** should be a reduction in prevalence of **28%**. This target is estimated with a 95% uncertainty interval from **15%** to **47%**.

Input parameters

- Among women of reproductive age in **Malaysia**, the prevalence of overall anaemia is **64.7%**
  - **Mild anaemia:** 51.0%

- Moderate anaemia: 13.5%
- Severe anaemia: 0.2%

- The prevalence of malaria is **0.0%**.

Intervention coverage and costs

Iron supplementation (antenatal care)

- **Current coverage:** 84.2%
- **Maximum feasible coverage:** 97.0%
- **Estimated unit cost:** USD $10.80 (5.39 - 18.56)

Iron supplementation (all women of reproductive age)

- **Current coverage:** 34.7%
- **Maximum feasible coverage:** 97.0%
- **Estimated unit cost:** USD $6.56 (3.77 - 16.86)

Antenatal preventative IPTp-SP

- **Current coverage:** 0.0%
- **Maximum feasible coverage:** 97.0%
- **Estimated unit cost:** USD $7.83 (3.99 - 13.35)

Staple food supplementation

- **Current coverage:** 14.8%
- **Maximum feasible coverage:** 100.0%
- **Estimated unit cost:** USD $0.72 (0.24 - 1.84)

Cost-Effectiveness Analysis

The estimated cost per Year of Life Disabled (YLD) averted for each intervention in **Malaysia** is given below, with interquartile ranges (IQR):

Iron supplementation (antenatal care)

- **Median Cost per YLD:** USD $2056

- **IQR:** USD $1311 – $4844

Iron supplementation (all women of reproductive age)

- **Median Cost per YLD:** USD $3576
- **IQR:** USD $2356 – $5379

Antenatal preventative IPTp-SP

- **Median Cost per YLD:** >USD 100k
- **IQR:** >USD 100k

Staple food supplementation

- **Median Cost per YLD:** USD $1118
- **IQR:** USD $689 – $1683

Economic Considerations

Cost-effectiveness thresholds (CET, in USD per DALY averted) in **Malaysia** is as follows:

- **Estimates from Pichon-Riviere et al. (Lower Bound):** USD $3262
- **Estimates from Pichon-Riviere et al. (Upper Bound):** USD $4426
- **CET equal to per capita GDP:** USD $11649
- **CET equal to 2.3 (LMICs) or 1.7 (HICs) times per capita GDP:** USD $26792

⬆ Back to Table of Contents

Anaemia Reduction in Maldives

National Target

For our baseline CET = 1 x GDP per capita, the national target for anaemia in **Maldives** should be a reduction in prevalence of **29%**. This target is estimated with a 95% uncertainty interval from **15%** to **47%**.

For CET based on Pichon-Riviere, the national target for anaemia in **Maldives** should be a reduction in prevalence of **28%**. This target is estimated with a 95% uncertainty interval from **15%** to **46%**.

For a near-infinite CET, the national target for anaemia in **Maldives** should be a reduction in prevalence of **29%**. This target is estimated with a 95% uncertainty interval from **15%** to **46%**.

Input parameters

- Among women of reproductive age in **Maldives**, the prevalence of overall anaemia is **60.2%**
    - **Mild anaemia:** 29.3%
    - **Moderate anaemia:** 29.0%
    - **Severe anaemia:** 2.0%
- There is no data on malaria prevalence; assume 0%.

Intervention coverage and costs

Iron supplementation (antenatal care)

- **Current coverage:** 90.8%
- **Maximum feasible coverage:** 98.8%
- **Estimated unit cost:** USD $9.89 (4.90 - 16.49)

Iron supplementation (all women of reproductive age)

- **Current coverage:** 37.4%
- **Maximum feasible coverage:** 98.8%
- **Estimated unit cost:** USD $6.22 (3.56 - 15.61)

Antenatal preventative IPTp-SP

- **Current coverage:** 0.0%
- **Maximum feasible coverage:** 98.8%
- **Estimated unit cost:** USD $6.85 (3.48 - 11.20)

Staple food supplementation

- **Current coverage:** 4.3%
- **Maximum feasible coverage:** 100.0%
- **Estimated unit cost:** USD $0.48 (0.07 - 1.41)

Cost-Effectiveness Analysis

The estimated cost per Year of Life Disabled (YLD) averted for each intervention in **Maldives** is given below, with interquartile ranges (IQR):

Iron supplementation (antenatal care)

- **Median Cost per YLD:** USD $1499
- **IQR:** USD $809 – >USD 100k

Iron supplementation (all women of reproductive age)

- **Median Cost per YLD:** USD $1946
- **IQR:** USD $1348 – $2766

Antenatal preventative IPTp-SP

- **Median Cost per YLD:** >USD 100k
- **IQR:** >USD 100k

Staple food supplementation

- **Median Cost per YLD:** USD $570
- **IQR:** USD $363 – $876

Economic Considerations

Cost-effectiveness thresholds (CET, in USD per DALY averted) in **Maldives** is as follows:

- **Estimates from Pichon-Riviere et al. (Lower Bound):** USD $7474
- **Estimates from Pichon-Riviere et al. (Upper Bound):** USD $10261
- **CET equal to per capita GDP:** USD $12667
- **CET equal to 2.3 (LMICs) or 1.7 (HICs) times per capita GDP:** USD $29135

⬆ Back to Table of Contents

Anaemia Reduction in Mali

National Target

For our baseline CET = 1 x GDP per capita, the national target for anaemia in **Mali** should be a reduction in prevalence of **18%**. This target is estimated with a 95% uncertainty interval from **1%** to **33%**.

For CET based on Pichon-Riviere, the national target for anaemia in **Mali** should be a reduction in prevalence of **0%**. This target is estimated with a 95% uncertainty interval from **0%** to **14%**.

For a near-infinite CET, the national target for anaemia in **Mali** should be a reduction in prevalence of **22%**. This target is estimated with a 95% uncertainty interval from **13%** to **36%**.

Input parameters

- Among women of reproductive age in **Mali**, the prevalence of overall anaemia is **59.6%**
    - **Mild anaemia:** 26.8%
    - **Moderate anaemia:** 29.8%
    - **Severe anaemia:** 3.1%
- The prevalence of malaria is **20.9%**.

Intervention coverage and costs

Iron supplementation (antenatal care)

- **Current coverage:** 78.4%
- **Maximum feasible coverage:** 84.2%
- **Estimated unit cost:** USD $2.57 (1.14 - 4.97)

Iron supplementation (all women of reproductive age)

- **Current coverage:** 32.3%
- **Maximum feasible coverage:** 84.2%
- **Estimated unit cost:** USD $2.75 (1.80 - 9.57)

Antenatal preventative IPTp-SP

- **Current coverage:** 54.7%
- **Maximum feasible coverage:** 84.2%
- **Estimated unit cost:** USD $1.29 (0.68 - 2.55)

Staple food supplementation

- **Current coverage:** 3.8%
- **Maximum feasible coverage:** 61.7%
- **Estimated unit cost:** USD $0.49 (0.10 - 1.61)

Cost-Effectiveness Analysis

The estimated cost per Year of Life Disabled (YLD) averted for each intervention in **Mali** is given below, with interquartile ranges (IQR):

Iron supplementation (antenatal care)

- **Median Cost per YLD:** USD $329
- **IQR:** USD $209 – >USD 100k

Iron supplementation (all women of reproductive age)

- **Median Cost per YLD:** USD $989
- **IQR:** USD $667 – $1446

Antenatal preventative IPTp-SP

- **Median Cost per YLD:** USD $714
- **IQR:** USD $521 – $933

Staple food supplementation

- **Median Cost per YLD:** USD $548
- **IQR:** USD $343 – $854

Economic Considerations

Cost-effectiveness thresholds (CET, in USD per DALY averted) in **Mali** is as follows:

- **Estimates from Pichon-Riviere et al. (Lower Bound):** USD $45
- **Estimates from Pichon-Riviere et al. (Upper Bound):** USD $144
- **CET equal to per capita GDP:** USD $897
- **CET equal to 2.3 (LMICs) or 1.7 (HICs) times per capita GDP:** USD $2064

⬆ Back to Table of Contents

Anaemia Reduction in Malta

National Target

For our baseline CET = 1 x GDP per capita, the national target for anaemia in **Malta** should be a reduction in prevalence of **16%**. This target is estimated with a 95% uncertainty interval from **6%** to **34%**.

For CET based on Pichon-Riviere, the national target for anaemia in **Malta** should be a reduction in prevalence of **16%**. This target is estimated with a 95% uncertainty interval from **6%** to **33%**.

For a near-infinite CET, the national target for anaemia in **Malta** should be a reduction in prevalence of **25%**. This target is estimated with a 95% uncertainty interval from **13%** to **40%**.

Input parameters

- Among women of reproductive age in **Malta**, the prevalence of overall anaemia is **9.2%**
  - **Mild anaemia:** 7.5%
  - **Moderate anaemia:** 1.6%
  - **Severe anaemia:** 0.0%
- There is no data on malaria prevalence; assume 0%.

Intervention coverage and costs

Iron supplementation (antenatal care)

- **Current coverage:** 81.0%
- **Maximum feasible coverage:** 100.0%
- **Estimated unit cost:** USD $135.19 (56.86 - 211.44)

Iron supplementation (all women of reproductive age)

- **Current coverage:** 33.4%
- **Maximum feasible coverage:** 100.0%
- **Estimated unit cost:** USD $88.43 (38.26 - 151.76)

Antenatal preventative IPTp-SP

- **Current coverage:** 0.0%
- **Maximum feasible coverage:** 100.0%
- **Estimated unit cost:** USD $75.50 (37.82 - 119.79)

Staple food supplementation

- **Current coverage:** 35.9%
- **Maximum feasible coverage:** 100.0%
- **Estimated unit cost:** USD $0.24 (0.12 - 0.42)

Cost-Effectiveness Analysis

The estimated cost per Year of Life Disabled (YLD) averted for each intervention in **Malta** is given below, with interquartile ranges (IQR):

Iron supplementation (antenatal care)

- **Median Cost per YLD:** >USD 100k
- **IQR:** >USD 100k

Iron supplementation (all women of reproductive age)

- **Median Cost per YLD:** >USD 100k
- **IQR:** >USD 100k

Antenatal preventative IPTp-SP

- **Median Cost per YLD:** >USD 100k
- **IQR:** >USD 100k

Staple food supplementation

- **Median Cost per YLD:** USD $3629
- **IQR:** USD $2365 – $5896

Economic Considerations

Cost-effectiveness thresholds (CET, in USD per DALY averted) in **Malta** is as follows:

- **Estimates from Pichon-Riviere et al. (Lower Bound):** USD $23487
- **Estimates from Pichon-Riviere et al. (Upper Bound):** USD $27654
- **CET equal to per capita GDP:** USD $37882
- **CET equal to 2.3 (LMICs) or 1.7 (HICs) times per capita GDP:** USD $53035

⬆ Back to Table of Contents

Anaemia Reduction in Marshall Islands

National Target

For our baseline CET = 1 x GDP per capita, the national target for anaemia in **Marshall Islands** should be a reduction in prevalence of **27%**. This target is estimated with a 95% uncertainty interval from **13%** to **45%**.

For CET based on Pichon-Riviere, the national target for anaemia in **Marshall Islands** should be a reduction in prevalence of **25%**. This target is estimated with a 95% uncertainty interval from **11%** to **43%**.

For a near-infinite CET, the national target for anaemia in **Marshall Islands** should be a reduction in prevalence of **26%**. This target is estimated with a 95% uncertainty interval from **14%** to **44%**.

Input parameters

- Among women of reproductive age in **Marshall Islands**, the prevalence of overall anaemia is **35.6%**
    - **Mild anaemia:** 18.4%
    - **Moderate anaemia:** 15.6%
    - **Severe anaemia:** 1.6%
- There is no data on malaria prevalence; assume 0%.

Intervention coverage and costs

Iron supplementation (antenatal care)

- **Current coverage:** 84.2%

- **Maximum feasible coverage:** 85.4%
- **Estimated unit cost:** USD $8.21 (4.04 - 14.37)

Iron supplementation (all women of reproductive age)

- **Current coverage:** 34.7%
- **Maximum feasible coverage:** 85.4%
- **Estimated unit cost:** USD $5.64 (2.83 - 11.80)

Antenatal preventative IPTp-SP

- **Current coverage:** 0.0%
- **Maximum feasible coverage:** 85.4%
- **Estimated unit cost:** USD $6.01 (3.08 - 10.61)

Staple food supplementation

- **Current coverage:** 15.5%
- **Maximum feasible coverage:** 100.0%
- **Estimated unit cost:** USD $0.32 (0.04 - 0.91)

Cost-Effectiveness Analysis

The estimated cost per Year of Life Disabled (YLD) averted for each intervention in **Marshall Islands** is given below, with interquartile ranges (IQR):

Iron supplementation (antenatal care)

- **Median Cost per YLD:** USD $2654
- **IQR:** USD $1192 – >USD 100k

Iron supplementation (all women of reproductive age)

- **Median Cost per YLD:** USD $2599
- **IQR:** USD $1799 – $3744

Antenatal preventative IPTp-SP

- **Median Cost per YLD:** >USD 100k

- **IQR:** >USD 100k

Staple food supplementation

- **Median Cost per YLD:** USD $468
- **IQR:** USD $299 – $732

Economic Considerations

Cost-effectiveness thresholds (CET, in USD per DALY averted) in **Marshall Islands** is as follows:

- **Estimates from Pichon-Riviere et al. (Lower Bound):** USD $4193
- **Estimates from Pichon-Riviere et al. (Upper Bound):** USD $3922
- **CET equal to per capita GDP:** USD $6763
- **CET equal to 2.3 (LMICs) or 1.7 (HICs) times per capita GDP:** USD $15554

⬆ Back to Table of Contents

Anaemia Reduction in Mauritania

National Target

For our baseline CET = 1 x GDP per capita, the national target for anaemia in **Mauritania** should be a reduction in prevalence of **15%**. This target is estimated with a 95% uncertainty interval from **6%** to **29%**.

For CET based on Pichon-Riviere, the national target for anaemia in **Mauritania** should be a reduction in prevalence of **10%**. This target is estimated with a 95% uncertainty interval from **0%** to **26%**.

For a near-infinite CET, the national target for anaemia in **Mauritania** should be a reduction in prevalence of **19%**. This target is estimated with a 95% uncertainty interval from **10%** to **32%**.

Input parameters

- Among women of reproductive age in **Mauritania**, the prevalence of overall anaemia is **50.9%**
  - **Mild anaemia:** 30.6%
  - **Moderate anaemia:** 19.2%

- Severe anaemia: 1.0%

- The prevalence of malaria is **5.2%**.

Intervention coverage and costs

Iron supplementation (antenatal care)

- **Current coverage:** 82.3%
- **Maximum feasible coverage:** 85.2%
- **Estimated unit cost:** USD $8.24 (3.92 - 13.22)

Iron supplementation (all women of reproductive age)

- **Current coverage:** 33.9%
- **Maximum feasible coverage:** 85.2%
- **Estimated unit cost:** USD $8.99 (4.47 - 16.15)

Antenatal preventative IPTp-SP

- **Current coverage:** 22.9%
- **Maximum feasible coverage:** 85.2%
- **Estimated unit cost:** USD $5.59 (2.83 - 9.04)

Staple food supplementation

- **Current coverage:** 15.9%
- **Maximum feasible coverage:** 65.5%
- **Estimated unit cost:** USD $0.28 (0.04 - 0.87)

Cost-Effectiveness Analysis

The estimated cost per Year of Life Disabled (YLD) averted for each intervention in **Mauritania** is given below, with interquartile ranges (IQR):

Iron supplementation (antenatal care)

- **Median Cost per YLD:** USD $1390
- **IQR:** USD $853 – >USD 100k

Iron supplementation (all women of reproductive age)

- **Median Cost per YLD:** USD $3239
- **IQR:** USD $2301 – $4606

Antenatal preventative IPTp-SP

- **Median Cost per YLD:** USD $10296
- **IQR:** USD $7719 – $13442

Staple food supplementation

- **Median Cost per YLD:** USD $392
- **IQR:** USD $241 – $614

Economic Considerations

Cost-effectiveness thresholds (CET, in USD per DALY averted) in **Mauritania** is as follows:

- **Estimates from Pichon-Riviere et al. (Lower Bound):** USD $236
- **Estimates from Pichon-Riviere et al. (Upper Bound):** USD $473
- **CET equal to per capita GDP:** USD $2149
- **CET equal to 2.3 (LMICs) or 1.7 (HICs) times per capita GDP:** USD $4944

⬆ Back to Table of Contents

Anaemia Reduction in Mauritius

National Target

For our baseline CET = 1 x GDP per capita, the national target for anaemia in **Mauritius** should be a reduction in prevalence of **25%**. This target is estimated with a 95% uncertainty interval from **12%** to **41%**.

For CET based on Pichon-Riviere, the national target for anaemia in **Mauritius** should be a reduction in prevalence of **22%**. This target is estimated with a 95% uncertainty interval from **9%** to **39%**.

For a near-infinite CET, the national target for anaemia in **Mauritius** should be a reduction in prevalence of **25%**. This target is estimated with a 95% uncertainty interval from **13%** to **41%**.

Input parameters

- Among women of reproductive age in **Mauritius**, the prevalence of overall anaemia is **35.1%**
  - **Mild anaemia:** 22.7%
  - **Moderate anaemia:** 11.9%
  - **Severe anaemia:** 0.6%
- There is no data on malaria prevalence; assume 0%.

Intervention coverage and costs

Iron supplementation (antenatal care)

- **Current coverage:** 90.2%
- **Maximum feasible coverage:** 94.6%
- **Estimated unit cost:** USD $10.63 (5.15 - 16.97)

Iron supplementation (all women of reproductive age)

- **Current coverage:** 37.2%
- **Maximum feasible coverage:** 94.6%
- **Estimated unit cost:** USD $6.38 (3.62 - 15.56)

Antenatal preventative IPTp-SP

- **Current coverage:** 27.8%
- **Maximum feasible coverage:** 94.6%
- **Estimated unit cost:** USD $7.36 (3.71 - 11.66)

Staple food supplementation

- **Current coverage:** 26.5%
- **Maximum feasible coverage:** 97.2%
- **Estimated unit cost:** USD $0.38 (0.10 - 0.97)

Cost-Effectiveness Analysis

The estimated cost per Year of Life Disabled (YLD) averted for each intervention in **Mauritius** is given below, with interquartile ranges (IQR):

Iron supplementation (antenatal care)

- **Median Cost per YLD:** USD $3463
- **IQR:** USD $1934 – >USD 100k

Iron supplementation (all women of reproductive age)

- **Median Cost per YLD:** USD $4499
- **IQR:** USD $3098 – $6613

Antenatal preventative IPTp-SP

- **Median Cost per YLD:** >USD 100k
- **IQR:** >USD 100k

Staple food supplementation

- **Median Cost per YLD:** USD $775
- **IQR:** USD $529 – $1225

Economic Considerations

Cost-effectiveness thresholds (CET, in USD per DALY averted) in **Mauritius** is as follows:

- **Estimates from Pichon-Riviere et al. (Lower Bound):** USD $4795
- **Estimates from Pichon-Riviere et al. (Upper Bound):** USD $5708
- **CET equal to per capita GDP:** USD $11417
- **CET equal to 2.3 (LMICs) or 1.7 (HICs) times per capita GDP:** USD $26259

⬆ Back to Table of Contents

Anaemia Reduction in Mexico

National Target

For our baseline CET = 1 x GDP per capita, the national target for anaemia in **Mexico** should be a reduction in prevalence of **8%**. This target is estimated with a 95% uncertainty interval from **0%** to **28%**.

For CET based on Pichon-Riviere, the national target for anaemia in **Mexico** should be a reduction in prevalence of **0%**. This target is estimated with a 95% uncertainty interval from **0%** to **16%**.

For a near-infinite CET, the national target for anaemia in **Mexico** should be a reduction in prevalence of **19%**. This target is estimated with a 95% uncertainty interval from **10%** to **31%**.

Input parameters

- Among women of reproductive age in **Mexico**, the prevalence of overall anaemia is **10.8%**
  - **Mild anaemia:** 7.0%
  - **Moderate anaemia:** 3.6%
  - **Severe anaemia:** 0.3%
- The prevalence of malaria is **0.0%**.

Intervention coverage and costs

Iron supplementation (antenatal care)

- **Current coverage:** 88.3%
- **Maximum feasible coverage:** 93.3%
- **Estimated unit cost:** USD $17.79 (8.80 - 28.00)

Iron supplementation (all women of reproductive age)

- **Current coverage:** 36.4%
- **Maximum feasible coverage:** 88.3%
- **Estimated unit cost:** USD $8.98 (4.51 - 16.48)

Antenatal preventative IPTp-SP

- **Current coverage:** 0.1%
- **Maximum feasible coverage:** 73.9%
- **Estimated unit cost:** USD $12.91 (6.52 - 20.28)

Staple food supplementation

- **Current coverage:** 0.6%
- **Maximum feasible coverage:** 50.8%
- **Estimated unit cost:** USD $3.09 (1.72 - 3.17)

Cost-Effectiveness Analysis

The estimated cost per Year of Life Disabled (YLD) averted for each intervention in **Mexico** is given below, with interquartile ranges (IQR):

Iron supplementation (antenatal care)

- **Median Cost per YLD:** USD $19252
- **IQR:** USD $11271 – >USD 100k

Iron supplementation (all women of reproductive age)

- **Median Cost per YLD:** USD $19225
- **IQR:** USD $14368 – $26004

Antenatal preventative IPTp-SP

- **Median Cost per YLD:** >USD 100k
- **IQR:** >USD 100k

Staple food supplementation

- **Median Cost per YLD:** USD $15961
- **IQR:** USD $12130 – $22104

Economic Considerations

Cost-effectiveness thresholds (CET, in USD per DALY averted) in **Mexico** is as follows:

- **Estimates from Pichon-Riviere et al. (Lower Bound):** USD $5292
- **Estimates from Pichon-Riviere et al. (Upper Bound):** USD $7242
- **CET equal to per capita GDP:** USD $13926

- **CET equal to 2.3 (LMICs) or 1.7 (HICs) times per capita GDP:** USD $32030

⬆ Back to Table of Contents

Anaemia Reduction in Micronesia (Federated States of)

National Target

For our baseline CET = 1 x GDP per capita, the national target for anaemia in **Micronesia (Federated States of)** should be a reduction in prevalence of **27%**. This target is estimated with a 95% uncertainty interval from **9%** to **46%**.

For CET based on Pichon-Riviere, the national target for anaemia in **Micronesia (Federated States of)** should be a reduction in prevalence of **27%**. This target is estimated with a 95% uncertainty interval from **7%** to **45%**.

For a near-infinite CET, the national target for anaemia in **Micronesia (Federated States of)** should be a reduction in prevalence of **28%**. This target is estimated with a 95% uncertainty interval from **14%** to **46%**.

Input parameters

- Among women of reproductive age in **Micronesia (Federated States of)**, the prevalence of overall anaemia is **40.4%**
    - **Mild anaemia:** 23.5%
    - **Moderate anaemia:** 15.8%
    - **Severe anaemia:** 1.1%
- There is no data on malaria prevalence; assume 0%.

Intervention coverage and costs

Iron supplementation (antenatal care)

- **Current coverage:** NaN%
- **Maximum feasible coverage:** NaN%
- **Estimated unit cost:** USD $NaN (NaN - NaN)

Iron supplementation (all women of reproductive age)

- **Current coverage:** NaN%
- **Maximum feasible coverage:** NaN%
- **Estimated unit cost:** USD $NaN (NaN - NaN)

Antenatal preventative IPTp-SP

- **Current coverage:** NaN%
- **Maximum feasible coverage:** NaN%
- **Estimated unit cost:** USD $NaN (NaN - NaN)

Staple food supplementation

- **Current coverage:** NaN%
- **Maximum feasible coverage:** NaN%
- **Estimated unit cost:** USD $NaN (NaN - NaN)

Cost-Effectiveness Analysis

The estimated cost per Year of Life Disabled (YLD) averted for each intervention in **Micronesia (Federated States of)** is given below, with interquartile ranges (IQR):

Iron supplementation (antenatal care)

- **Median Cost per YLD:** USD $1439
- **IQR:** USD $886 – >USD 100k

Iron supplementation (all women of reproductive age)

- **Median Cost per YLD:** USD $2265
- **IQR:** USD $1581 – $3140

Antenatal preventative IPTp-SP

- **Median Cost per YLD:** >USD 100k
- **IQR:** >USD 100k

Staple food supplementation

- **Median Cost per YLD:** USD $1181

- **IQR:** USD $737 – $1805

Economic Considerations

Cost-effectiveness thresholds (CET, in USD per DALY averted) in **Micronesia (Federated States of)** is as follows:

- **Estimates from Pichon-Riviere et al. (Lower Bound):** USD $NaN
- **Estimates from Pichon-Riviere et al. (Upper Bound):** USD $NaN
- **CET equal to per capita GDP:** USD $NaN
- **CET equal to 2.3 (LMICs) or 1.7 (HICs) times per capita GDP:** USD $NaN

⬆ Back to Table of Contents

Anaemia Reduction in Moldova

National Target

For our baseline CET = 1 x GDP per capita, the national target for anaemia in **Moldova** should be a reduction in prevalence of **26%**. This target is estimated with a 95% uncertainty interval from **1%** to **45%**.

For CET based on Pichon-Riviere, the national target for anaemia in **Moldova** should be a reduction in prevalence of **21%**. This target is estimated with a 95% uncertainty interval from **0%** to **41%**.

For a near-infinite CET, the national target for anaemia in **Moldova** should be a reduction in prevalence of **31%**. This target is estimated with a 95% uncertainty interval from **17%** to **49%**.

Input parameters

- Among women of reproductive age in **Moldova**, the prevalence of overall anaemia is **24.8%**
  - **Mild anaemia:** 15.7%
  - **Moderate anaemia:** 8.7%
  - **Severe anaemia:** 0.4%
- There is no data on malaria prevalence; assume 0%.

Intervention coverage and costs

Iron supplementation (antenatal care)

- **Current coverage:** 58.1%
- **Maximum feasible coverage:** 83.8%
- **Estimated unit cost:** USD $NaN (NaN - NaN)

Iron supplementation (all women of reproductive age)

- **Current coverage:** 24.0%
- **Maximum feasible coverage:** 83.8%
- **Estimated unit cost:** USD $NaN (NaN - NaN)

Antenatal preventative IPTp-SP

- **Current coverage:** 0.0%
- **Maximum feasible coverage:** 83.8%
- **Estimated unit cost:** USD $NaN (NaN - NaN)

Staple food supplementation

- **Current coverage:** 4.3%
- **Maximum feasible coverage:** 100.0%
- **Estimated unit cost:** USD $NaN (NaN - NaN)

Cost-Effectiveness Analysis

The estimated cost per Year of Life Disabled (YLD) averted for each intervention in **Moldova** is given below, with interquartile ranges (IQR):

Iron supplementation (antenatal care)

- **Median Cost per YLD:** USD $3315
- **IQR:** USD $2409 – $4572

Iron supplementation (all women of reproductive age)

- **Median Cost per YLD:** USD $8233
- **IQR:** USD $5787 – $11562

Antenatal preventative IPTp-SP

- **Median Cost per YLD:** >USD 100k
- **IQR:** >USD 100k

Staple food supplementation

- **Median Cost per YLD:** USD $2694
- **IQR:** USD $1968 – $3794

Economic Considerations

Cost-effectiveness thresholds (CET, in USD per DALY averted) in **Moldova** is as follows:

- **Estimates from Pichon-Riviere et al. (Lower Bound):** USD $NaN
- **Estimates from Pichon-Riviere et al. (Upper Bound):** USD $NaN
- **CET equal to per capita GDP:** USD $NaN
- **CET equal to 2.3 (LMICs) or 1.7 (HICs) times per capita GDP:** USD $NaN

⬆ Back to Table of Contents

Anaemia Reduction in Monaco

National Target

For our baseline CET = 1 x GDP per capita, the national target for anaemia in **Monaco** should be a reduction in prevalence of **22%**. This target is estimated with a 95% uncertainty interval from **8%** to **40%**.

For CET based on Pichon-Riviere, the national target for anaemia in **Monaco** should be a reduction in prevalence of **19%**. This target is estimated with a 95% uncertainty interval from **7%** to **37%**.

For a near-infinite CET, the national target for anaemia in **Monaco** should be a reduction in prevalence of **25%**. This target is estimated with a 95% uncertainty interval from **13%** to **40%**.

Input parameters

- Among women of reproductive age in **Monaco**, the prevalence of overall anaemia is **6.5%**

- **Mild anaemia:** 5.4%
  - **Moderate anaemia:** 1.0%
  - **Severe anaemia:** 0.0%
- There is no data on malaria prevalence; assume 0%.

Intervention coverage and costs

Iron supplementation (antenatal care)

- **Current coverage:** 81.0%
- **Maximum feasible coverage:** 100.0%
- **Estimated unit cost:** USD $57.38 (17.80 - 94.65)

Iron supplementation (all women of reproductive age)

- **Current coverage:** 33.4%
- **Maximum feasible coverage:** 100.0%
- **Estimated unit cost:** USD $35.83 (10.04 - 50.86)

Antenatal preventative IPTp-SP

- **Current coverage:** 0.0%
- **Maximum feasible coverage:** 100.0%
- **Estimated unit cost:** USD $16.27 (8.20 - 27.23)

Staple food supplementation

- **Current coverage:** 35.7%
- **Maximum feasible coverage:** 100.0%
- **Estimated unit cost:** USD $0.25 (0.14 - 0.40)

Cost-Effectiveness Analysis

The estimated cost per Year of Life Disabled (YLD) averted for each intervention in **Monaco** is given below, with interquartile ranges (IQR):

Iron supplementation (antenatal care)

- **Median Cost per YLD:** >USD 100k
- **IQR:** USD $96671 – >USD 100k

Iron supplementation (all women of reproductive age)

- **Median Cost per YLD:** >USD 100k
- **IQR:** >USD 100k

Antenatal preventative IPTp-SP

- **Median Cost per YLD:** >USD 100k
- **IQR:** >USD 100k

Staple food supplementation

- **Median Cost per YLD:** USD $6652
- **IQR:** USD $4585 – $10407

Economic Considerations

Cost-effectiveness thresholds (CET, in USD per DALY averted) in **Monaco** is as follows:

- **Estimates from Pichon-Riviere et al. (Lower Bound):** USD $110797
- **Estimates from Pichon-Riviere et al. (Upper Bound):** USD $163786
- **CET equal to per capita GDP:** USD $240862
- **CET equal to 2.3 (LMICs) or 1.7 (HICs) times per capita GDP:** USD $337207

⬆ Back to Table of Contents

Anaemia Reduction in Mongolia

National Target

For our baseline CET = 1 x GDP per capita, the national target for anaemia in **Mongolia** should be a reduction in prevalence of **30%**. This target is estimated with a 95% uncertainty interval from **15%** to **49%**.

For CET based on Pichon-Riviere, the national target for anaemia in **Mongolia** should be a reduction in prevalence of **27%**. This target is estimated with a 95% uncertainty interval from

11% to **46%**.

For a near-infinite CET, the national target for anaemia in **Mongolia** should be a reduction in prevalence of **30%**. This target is estimated with a 95% uncertainty interval from **16%** to **48%**.

Input parameters

- Among women of reproductive age in **Mongolia**, the prevalence of overall anaemia is **28.1%**
  - **Mild anaemia:** 11.2%
  - **Moderate anaemia:** 14.1%
  - **Severe anaemia:** 2.8%
- There is no data on malaria prevalence; assume 0%.

Intervention coverage and costs

Iron supplementation (antenatal care)

- **Current coverage:** 84.2%
- **Maximum feasible coverage:** 88.0%
- **Estimated unit cost:** USD $4.24 (2.03 - 7.49)

Iron supplementation (all women of reproductive age)

- **Current coverage:** 34.7%
- **Maximum feasible coverage:** 88.0%
- **Estimated unit cost:** USD $3.05 (1.55 - 7.37)

Antenatal preventative IPTp-SP

- **Current coverage:** 35.0%
- **Maximum feasible coverage:** 88.0%
- **Estimated unit cost:** USD $2.67 (1.41 - 4.77)

Staple food supplementation

- **Current coverage:** 0.5%
- **Maximum feasible coverage:** 100.0%
- **Estimated unit cost:** USD $0.09 (0.01 - 0.29)

Cost-Effectiveness Analysis

The estimated cost per Year of Life Disabled (YLD) averted for each intervention in **Mongolia** is given below, with interquartile ranges (IQR):

Iron supplementation (antenatal care)

- **Median Cost per YLD:** USD $1089
- **IQR:** USD $579 – >USD 100k

Iron supplementation (all women of reproductive age)

- **Median Cost per YLD:** USD $1522
- **IQR:** USD $1076 – $2172

Antenatal preventative IPTp-SP

- **Median Cost per YLD:** >USD 100k
- **IQR:** >USD 100k

Staple food supplementation

- **Median Cost per YLD:** USD $162
- **IQR:** USD $103 – $237

Economic Considerations

Cost-effectiveness thresholds (CET, in USD per DALY averted) in **Mongolia** is as follows:

- **Estimates from Pichon-Riviere et al. (Lower Bound):** USD $2652
- **Estimates from Pichon-Riviere et al. (Upper Bound):** USD $1672
- **CET equal to per capita GDP:** USD $5765
- **CET equal to 2.3 (LMICs) or 1.7 (HICs) times per capita GDP:** USD $13259

⬆ Back to Table of Contents

Anaemia Reduction in Montenegro

National Target

For our baseline CET = 1 x GDP per capita, the national target for anaemia in **Montenegro** should be a reduction in prevalence of **15%**. This target is estimated with a 95% uncertainty interval from **2%** to **33%**.

For CET based on Pichon-Riviere, the national target for anaemia in **Montenegro** should be a reduction in prevalence of **11%.** This target is estimated with a 95% uncertainty interval from **2%** to **31%**.

For a near-infinite CET, the national target for anaemia in **Montenegro** should be a reduction in prevalence of **21%**. This target is estimated with a 95% uncertainty interval from **10%** to **33%**.

Input parameters

- Among women of reproductive age in **Montenegro**, the prevalence of overall anaemia is **21.7%**
    - **Mild anaemia:** 14.4%
    - **Moderate anaemia:** 6.9%
    - **Severe anaemia:** 0.4%
- There is no data on malaria prevalence; assume 0%.

Intervention coverage and costs

Iron supplementation (antenatal care)

- **Current coverage:** 54.0%
- **Maximum feasible coverage:** 94.0%
- **Estimated unit cost:** USD $18.45 (9.13 - 30.96)

Iron supplementation (all women of reproductive age)

- **Current coverage:** 22.2%
- **Maximum feasible coverage:** 94.0%
- **Estimated unit cost:** USD $11.48 (5.77 - 22.12)

Antenatal preventative IPTp-SP

- **Current coverage:** 0.0%

- **Maximum feasible coverage:** 94.0%
- **Estimated unit cost:** USD $14.37 (7.27 - 24.54)

Staple food supplementation

- **Current coverage:** 66.5%
- **Maximum feasible coverage:** 100.0%
- **Estimated unit cost:** USD $0.16 (0.08 - 0.25)

Cost-Effectiveness Analysis

The estimated cost per Year of Life Disabled (YLD) averted for each intervention in **Montenegro** is given below, with interquartile ranges (IQR):

Iron supplementation (antenatal care)

- **Median Cost per YLD:** USD $7422
- **IQR:** USD $5362 – $9974

Iron supplementation (all women of reproductive age)

- **Median Cost per YLD:** USD $12378
- **IQR:** USD $8662 – $17678

Antenatal preventative IPTp-SP

- **Median Cost per YLD:** >USD 100k
- **IQR:** >USD 100k

Staple food supplementation

- **Median Cost per YLD:** USD $449
- **IQR:** USD $308 – $700

Economic Considerations

Cost-effectiveness thresholds (CET, in USD per DALY averted) in **Montenegro** is as follows:

- **Estimates from Pichon-Riviere et al. (Lower Bound):** USD $5528

- **Estimates from Pichon-Riviere et al. (Upper Bound):** USD $9854
- **CET equal to per capita GDP:** USD $12017
- **CET equal to 2.3 (LMICs) or 1.7 (HICs) times per capita GDP:** USD $27639

⬆ Back to Table of Contents

Anaemia Reduction in Morocco

National Target

For our baseline CET = 1 x GDP per capita, the national target for anaemia in **Morocco** should be a reduction in prevalence of **21%**. This target is estimated with a 95% uncertainty interval from **10%** to **39%**.

For CET based on Pichon-Riviere, the national target for anaemia in **Morocco** should be a reduction in prevalence of **19%**. This target is estimated with a 95% uncertainty interval from **8%** to **36%**.

For a near-infinite CET, the national target for anaemia in **Morocco** should be a reduction in prevalence of **22%**. This target is estimated with a 95% uncertainty interval from **12%** to **39%**.

Input parameters

- Among women of reproductive age in **Morocco**, the prevalence of overall anaemia is **34.9%**
    - **Mild anaemia:** 21.8%
    - **Moderate anaemia:** 12.3%
    - **Severe anaemia:** 0.7%
- The prevalence of malaria is **0.0%**.

Intervention coverage and costs

Iron supplementation (antenatal care)

- **Current coverage:** 47.7%
- **Maximum feasible coverage:** 67.8%
- **Estimated unit cost:** USD $4.17 (1.95 - 7.28)

Iron supplementation (all women of reproductive age)

- **Current coverage:** 19.7%
- **Maximum feasible coverage:** 67.8%
- **Estimated unit cost:** USD $3.70 (1.85 - 8.32)

Antenatal preventative IPTp-SP

- **Current coverage:** 0.0%
- **Maximum feasible coverage:** 67.8%
- **Estimated unit cost:** USD $2.57 (1.33 - 4.55)

Staple food supplementation

- **Current coverage:** 27.7%
- **Maximum feasible coverage:** 99.5%
- **Estimated unit cost:** USD $0.09 (0.03 - 0.32)

Cost-Effectiveness Analysis

The estimated cost per Year of Life Disabled (YLD) averted for each intervention in **Morocco** is given below, with interquartile ranges (IQR):

Iron supplementation (antenatal care)

- **Median Cost per YLD:** USD $1221
- **IQR:** USD $825 – >USD 100k

Iron supplementation (all women of reproductive age)

- **Median Cost per YLD:** USD $2431
- **IQR:** USD $1685 – $3621

Antenatal preventative IPTp-SP

- **Median Cost per YLD:** >USD 100k
- **IQR:** >USD 100k

Staple food supplementation

- **Median Cost per YLD:** USD $214

- **IQR:** USD $132 – $347

Economic Considerations

Cost-effectiveness thresholds (CET, in USD per DALY averted) in **Morocco** is as follows:

- **Estimates from Pichon-Riviere et al. (Lower Bound):** USD $881
- **Estimates from Pichon-Riviere et al. (Upper Bound):** USD $1726
- **CET equal to per capita GDP:** USD $3672
- **CET equal to 2.3 (LMICs) or 1.7 (HICs) times per capita GDP:** USD $8446

⬆ Back to Table of Contents

Anaemia Reduction in Mozambique

National Target

For our baseline CET = 1 x GDP per capita, the national target for anaemia in **Mozambique** should be a reduction in prevalence of **20%**. This target is estimated with a 95% uncertainty interval from **2%** to **34%**.

For CET based on Pichon-Riviere, the national target for anaemia in **Mozambique** should be a reduction in prevalence of **2%**. This target is estimated with a 95% uncertainty interval from **0%** to **30%**.

For a near-infinite CET, the national target for anaemia in **Mozambique** should be a reduction in prevalence of **22%**. This target is estimated with a 95% uncertainty interval from **13%** to **36%**.

Input parameters

- Among women of reproductive age in **Mozambique**, the prevalence of overall anaemia is **57.3%**
  - **Mild anaemia:** 12.8%
  - **Moderate anaemia:** 29.5%
  - **Severe anaemia:** 14.9%
- The prevalence of malaria is **28.6%**.

Intervention coverage and costs

Iron supplementation (antenatal care)

- **Current coverage:** 77.9%
- **Maximum feasible coverage:** 87.0%
- **Estimated unit cost:** USD $2.48 (1.09 - 4.87)

Iron supplementation (all women of reproductive age)

- **Current coverage:** 32.1%
- **Maximum feasible coverage:** 87.0%
- **Estimated unit cost:** USD $2.65 (1.79 - 8.28)

Antenatal preventative IPTp-SP

- **Current coverage:** 44.9%
- **Maximum feasible coverage:** 87.0%
- **Estimated unit cost:** USD $1.24 (0.65 - 2.52)

Staple food supplementation

- **Current coverage:** 2.8%
- **Maximum feasible coverage:** 61.0%
- **Estimated unit cost:** USD $0.40 (0.10 - 1.38)

Cost-Effectiveness Analysis

The estimated cost per Year of Life Disabled (YLD) averted for each intervention in **Mozambique** is given below, with interquartile ranges (IQR):

Iron supplementation (antenatal care)

- **Median Cost per YLD:** USD $157
- **IQR:** USD $108 – >USD 100k

Iron supplementation (all women of reproductive age)

- **Median Cost per YLD:** USD $523
- **IQR:** USD $368 – $735

Antenatal preventative IPTp-SP

- **Median Cost per YLD:** USD $340
- **IQR:** USD $263 – $441

Staple food supplementation

- **Median Cost per YLD:** USD $259
- **IQR:** USD $164 – $383

Economic Considerations

Cost-effectiveness thresholds (CET, in USD per DALY averted) in **Mozambique** is as follows:

- **Estimates from Pichon-Riviere et al. (Lower Bound):** USD $67
- **Estimates from Pichon-Riviere et al. (Upper Bound):** USD $219
- **CET equal to per capita GDP:** USD $608
- **CET equal to 2.3 (LMICs) or 1.7 (HICs) times per capita GDP:** USD $1399

⬆ Back to Table of Contents

Anaemia Reduction in Myanmar (Burma)

National Target

For our baseline CET = 1 x GDP per capita, the national target for anaemia in **Myanmar (Burma)** should be a reduction in prevalence of **20%**. This target is estimated with a 95% uncertainty interval from **0%** to **46%**.

For CET based on Pichon-Riviere, the national target for anaemia in **Myanmar (Burma)** should be a reduction in prevalence of **0%**. This target is estimated with a 95% uncertainty interval from **0%** to **36%**.

For a near-infinite CET, the national target for anaemia in **Myanmar (Burma)** should be a reduction in prevalence of **29%**. This target is estimated with a 95% uncertainty interval from **15%** to **48%**.

Input parameters

- Among women of reproductive age in **Myanmar (Burma)**, the prevalence of overall anaemia is **57.5%**
    - **Mild anaemia:** 36.8%
    - **Moderate anaemia:** 20.1%
    - **Severe anaemia:** 0.6%
- There is no data on malaria prevalence; assume 0%.

Intervention coverage and costs

Iron supplementation (antenatal care)

- **Current coverage:** NaN%
- **Maximum feasible coverage:** NaN%
- **Estimated unit cost:** USD $NaN (NaN - NaN)

Iron supplementation (all women of reproductive age)

- **Current coverage:** NaN%
- **Maximum feasible coverage:** NaN%
- **Estimated unit cost:** USD $NaN (NaN - NaN)

Antenatal preventative IPTp-SP

- **Current coverage:** NaN%
- **Maximum feasible coverage:** NaN%
- **Estimated unit cost:** USD $NaN (NaN - NaN)

Staple food supplementation

- **Current coverage:** NaN%
- **Maximum feasible coverage:** NaN%
- **Estimated unit cost:** USD $NaN (NaN - NaN)

Cost-Effectiveness Analysis

The estimated cost per Year of Life Disabled (YLD) averted for each intervention in **Myanmar (Burma)** is given below, with interquartile ranges (IQR):

Iron supplementation (antenatal care)

- **Median Cost per YLD:** USD $661
- **IQR:** USD $374 – >USD 100k

Iron supplementation (all women of reproductive age)

- **Median Cost per YLD:** USD $1672
- **IQR:** USD $1170 – $2458

Antenatal preventative IPTp-SP

- **Median Cost per YLD:** >USD 100k
- **IQR:** >USD 100k

Staple food supplementation

- **Median Cost per YLD:** USD $1039
- **IQR:** USD $638 – $1581

Economic Considerations

Cost-effectiveness thresholds (CET, in USD per DALY averted) in **Myanmar (Burma)** is as follows:

- **Estimates from Pichon-Riviere et al. (Lower Bound):** USD $NaN
- **Estimates from Pichon-Riviere et al. (Upper Bound):** USD $NaN
- **CET equal to per capita GDP:** USD $NaN
- **CET equal to 2.3 (LMICs) or 1.7 (HICs) times per capita GDP:** USD $NaN

⬆ Back to Table of Contents

Anaemia Reduction in Namibia

National Target

For our baseline CET = 1 x GDP per capita, the national target for anaemia in **Namibia** should be a reduction in prevalence of **17%**. This target is estimated with a 95% uncertainty interval from **0%** to **34%**.

For CET based on Pichon-Riviere, the national target for anaemia in **Namibia** should be a reduction in prevalence of **10%**. This target is estimated with a 95% uncertainty interval from **0%** to **32%**.

For a near-infinite CET, the national target for anaemia in **Namibia** should be a reduction in prevalence of **21%**. This target is estimated with a 95% uncertainty interval from **11%** to **35%**.

Input parameters

- Among women of reproductive age in **Namibia**, the prevalence of overall anaemia is **30.3%**
  - **Mild anaemia:** 21.9%
  - **Moderate anaemia:** 8.2%
  - **Severe anaemia:** 0.2%
- The prevalence of malaria is **0.5%**.

Intervention coverage and costs

Iron supplementation (antenatal care)

- **Current coverage:** 88.4%
- **Maximum feasible coverage:** 96.6%
- **Estimated unit cost:** USD $5.33 (2.45 - 8.79)

Iron supplementation (all women of reproductive age)

- **Current coverage:** 36.4%
- **Maximum feasible coverage:** 96.6%
- **Estimated unit cost:** USD $4.18 (2.06 - 8.75)

Antenatal preventative IPTp-SP

- **Current coverage:** 4.9%
- **Maximum feasible coverage:** 96.6%
- **Estimated unit cost:** USD $3.40 (1.73 - 5.75)

Staple food supplementation

- **Current coverage:** 44.3%

- **Maximum feasible coverage:** 91.7%
- **Estimated unit cost:** USD $1.38 (1.08 - 2.06)

Cost-Effectiveness Analysis

The estimated cost per Year of Life Disabled (YLD) averted for each intervention in **Namibia** is given below, with interquartile ranges (IQR):

Iron supplementation (antenatal care)

- **Median Cost per YLD:** USD $2178
- **IQR:** USD $1375 – >USD 100k

Iron supplementation (all women of reproductive age)

- **Median Cost per YLD:** USD $3931
- **IQR:** USD $2723 – $5734

Antenatal preventative IPTp-SP

- **Median Cost per YLD:** >USD 100k
- **IQR:** >USD 100k

Staple food supplementation

- **Median Cost per YLD:** USD $3397
- **IQR:** USD $2399 – $4940

Economic Considerations

Cost-effectiveness thresholds (CET, in USD per DALY averted) in **Namibia** is as follows:

- **Estimates from Pichon-Riviere et al. (Lower Bound):** USD $2419
- **Estimates from Pichon-Riviere et al. (Upper Bound):** USD $3273
- **CET equal to per capita GDP:** USD $4743
- **CET equal to 2.3 (LMICs) or 1.7 (HICs) times per capita GDP:** USD $10908

⬆ Back to Table of Contents

Anaemia Reduction in Nauru

National Target

For our baseline CET = 1 x GDP per capita, the national target for anaemia in **Nauru** should be a reduction in prevalence of **24%**. This target is estimated with a 95% uncertainty interval from **10%** to **44%**.

For CET based on Pichon-Riviere, the national target for anaemia in **Nauru** should be a reduction in prevalence of **22%**. This target is estimated with a 95% uncertainty interval from **9%** to **41%**.

For a near-infinite CET, the national target for anaemia in **Nauru** should be a reduction in prevalence of **29%**. This target is estimated with a 95% uncertainty interval from **17%** to **46%**.

Input parameters

- Among women of reproductive age in **Nauru**, the prevalence of overall anaemia is **42.7%**
  - **Mild anaemia:** 26.5%
  - **Moderate anaemia:** 15.3%
  - **Severe anaemia:** 0.9%
- There is no data on malaria prevalence; assume 0%.

Intervention coverage and costs

Iron supplementation (antenatal care)

- **Current coverage:** 81.0%
- **Maximum feasible coverage:** 100.0%
- **Estimated unit cost:** USD $46.99 (12.58 - 76.20)

Iron supplementation (all women of reproductive age)

- **Current coverage:** 33.4%
- **Maximum feasible coverage:** 100.0%
- **Estimated unit cost:** USD $28.22 (8.56 - 56.10)

Antenatal preventative IPTp-SP

- **Current coverage:** 0.0%
- **Maximum feasible coverage:** 100.0%
- **Estimated unit cost:** USD $6.18 (3.16 - 10.89)

Staple food supplementation

- **Current coverage:** 13.8%
- **Maximum feasible coverage:** 100.0%
- **Estimated unit cost:** USD $0.50 (0.11 - 1.39)

Cost-Effectiveness Analysis

The estimated cost per Year of Life Disabled (YLD) averted for each intervention in **Nauru** is given below, with interquartile ranges (IQR):

Iron supplementation (antenatal care)

- **Median Cost per YLD:** USD $7412
- **IQR:** USD $5198 – $11415

Iron supplementation (all women of reproductive age)

- **Median Cost per YLD:** USD $12653
- **IQR:** USD $8685 – $18154

Antenatal preventative IPTp-SP

- **Median Cost per YLD:** >USD 100k
- **IQR:** >USD 100k

Staple food supplementation

- **Median Cost per YLD:** USD $792
- **IQR:** USD $518 – $1244

Economic Considerations

Cost-effectiveness thresholds (CET, in USD per DALY averted) in **Nauru** is as follows:

- **Estimates from Pichon-Riviere et al. (Lower Bound):** USD $6151
- **Estimates from Pichon-Riviere et al. (Upper Bound):** USD $8201
- **CET equal to per capita GDP:** USD $12060
- **CET equal to 2.3 (LMICs) or 1.7 (HICs) times per capita GDP:** USD $16884

⬆ Back to Table of Contents

Anaemia Reduction in Nepal

National Target

For our baseline CET = 1 x GDP per capita, the national target for anaemia in **Nepal** should be a reduction in prevalence of **10%**. This target is estimated with a 95% uncertainty interval from **0%** to **24%**.

For CET based on Pichon-Riviere, the national target for anaemia in **Nepal** should be a reduction in prevalence of **0%**. This target is estimated with a 95% uncertainty interval from **0%** to **15%**.

For a near-infinite CET, the national target for anaemia in **Nepal** should be a reduction in prevalence of **16%**. This target is estimated with a 95% uncertainty interval from **8%** to **26%**.

Input parameters

- Among women of reproductive age in **Nepal**, the prevalence of overall anaemia is **50.5%**
  - **Mild anaemia:** 33.7%
  - **Moderate anaemia:** 16.4%
  - **Severe anaemia:** 0.4%
- The prevalence of malaria is **0.0%**.

Intervention coverage and costs

Iron supplementation (antenatal care)

- **Current coverage:** 96.1%
- **Maximum feasible coverage:** 100.0%
- **Estimated unit cost:** USD $2.65 (1.25 - 5.18)

Iron supplementation (all women of reproductive age)

- **Current coverage:** 39.6%
- **Maximum feasible coverage:** 96.1%
- **Estimated unit cost:** USD $2.50 (1.74 - 9.65)

Antenatal preventative IPTp-SP

- **Current coverage:** 0.0%
- **Maximum feasible coverage:** 94.3%
- **Estimated unit cost:** USD $1.40 (0.76 - 2.74)

Staple food supplementation

- **Current coverage:** 0.9%
- **Maximum feasible coverage:** 36.0%
- **Estimated unit cost:** USD $0.49 (0.09 - 1.56)

Cost-Effectiveness Analysis

The estimated cost per Year of Life Disabled (YLD) averted for each intervention in **Nepal** is given below, with interquartile ranges (IQR):

Iron supplementation (antenatal care)

- **Median Cost per YLD:** >USD 100k
- **IQR:** USD $461 – >USD 100k

Iron supplementation (all women of reproductive age)

- **Median Cost per YLD:** USD $1818
- **IQR:** USD $1237 – $2836

Antenatal preventative IPTp-SP

- **Median Cost per YLD:** >USD 100k
- **IQR:** >USD 100k

Staple food supplementation

- **Median Cost per YLD:** USD $841
- **IQR:** USD $543 – $1314

Economic Considerations

Cost-effectiveness thresholds (CET, in USD per DALY averted) in **Nepal** is as follows:

- **Estimates from Pichon-Riviere et al. (Lower Bound):** USD $238
- **Estimates from Pichon-Riviere et al. (Upper Bound):** USD $463
- **CET equal to per capita GDP:** USD $1324
- **CET equal to 2.3 (LMICs) or 1.7 (HICs) times per capita GDP:** USD $3045

⬆ Back to Table of Contents

Anaemia Reduction in Netherlands

National Target

For our baseline CET = 1 x GDP per capita, the national target for anaemia in **Netherlands** should be a reduction in prevalence of **16%**. This target is estimated with a 95% uncertainty interval from **6%** to **36%**.

For CET based on Pichon-Riviere, the national target for anaemia in **Netherlands** should be a reduction in prevalence of **16%**. This target is estimated with a 95% uncertainty interval from **6%** to **33%**.

For a near-infinite CET, the national target for anaemia in **Netherlands** should be a reduction in prevalence of **24%**. This target is estimated with a 95% uncertainty interval from **13%** to **40%**.

Input parameters

- Among women of reproductive age in **Netherlands**, the prevalence of overall anaemia is **7.9%**
    - **Mild anaemia:** 6.5%
    - **Moderate anaemia:** 1.4%
    - **Severe anaemia:** 0.0%
- There is no data on malaria prevalence; assume 0%.

Intervention coverage and costs

Iron supplementation (antenatal care)

- **Current coverage:** 81.0%
- **Maximum feasible coverage:** 100.0%
- **Estimated unit cost:** USD $57.37 (17.80 - 94.48)

Iron supplementation (all women of reproductive age)

- **Current coverage:** 33.4%
- **Maximum feasible coverage:** 100.0%
- **Estimated unit cost:** USD $35.95 (10.09 - 51.19)

Antenatal preventative IPTp-SP

- **Current coverage:** 0.0%
- **Maximum feasible coverage:** 100.0%
- **Estimated unit cost:** USD $16.27 (8.20 - 27.23)

Staple food supplementation

- **Current coverage:** 37.8%
- **Maximum feasible coverage:** 100.0%
- **Estimated unit cost:** USD $0.15 (0.09 - 0.19)

Cost-Effectiveness Analysis

The estimated cost per Year of Life Disabled (YLD) averted for each intervention in **Netherlands** is given below, with interquartile ranges (IQR):

Iron supplementation (antenatal care)

- **Median Cost per YLD:** >USD 100k
- **IQR:** USD $67048 – >USD 100k

Iron supplementation (all women of reproductive age)

- **Median Cost per YLD:** >USD 100k
- **IQR:** >USD 100k

Antenatal preventative IPTp-SP

- **Median Cost per YLD:** >USD 100k
- **IQR:** >USD 100k

Staple food supplementation

- **Median Cost per YLD:** USD $2287
- **IQR:** USD $1510 – $3646

Economic Considerations

Cost-effectiveness thresholds (CET, in USD per DALY averted) in **Netherlands** is as follows:

- **Estimates from Pichon-Riviere et al. (Lower Bound):** USD $47528
- **Estimates from Pichon-Riviere et al. (Upper Bound):** USD $56283
- **CET equal to per capita GDP:** USD $62537
- **CET equal to 2.3 (LMICs) or 1.7 (HICs) times per capita GDP:** USD $87551

⬆ Back to Table of Contents

Anaemia Reduction in New Zealand

National Target

For our baseline CET = 1 x GDP per capita, the national target for anaemia in **New Zealand** should be a reduction in prevalence of **20%**. This target is estimated with a 95% uncertainty interval from **8%** to **39%**.

For CET based on Pichon-Riviere, the national target for anaemia in **New Zealand** should be a reduction in prevalence of **20%**. This target is estimated with a 95% uncertainty interval from **8%** to **38%**.

For a near-infinite CET, the national target for anaemia in **New Zealand** should be a reduction in prevalence of **28%**. This target is estimated with a 95% uncertainty interval from **16%** to **45%**.

Input parameters

- Among women of reproductive age in **New Zealand**, the prevalence of overall anaemia is **8.8%**
  - **Mild anaemia:** 6.4%
  - **Moderate anaemia:** 2.2%
  - **Severe anaemia:** 0.1%
- There is no data on malaria prevalence; assume 0%.

Intervention coverage and costs

Iron supplementation (antenatal care)

- **Current coverage:** 81.0%
- **Maximum feasible coverage:** 100.0%
- **Estimated unit cost:** USD $86.81 (32.53 - 137.34)

Iron supplementation (all women of reproductive age)

- **Current coverage:** 33.4%
- **Maximum feasible coverage:** 100.0%
- **Estimated unit cost:** USD $53.19 (18.70 - 75.95)

Antenatal preventative IPTp-SP

- **Current coverage:** 0.0%
- **Maximum feasible coverage:** 100.0%
- **Estimated unit cost:** USD $37.69 (18.91 - 58.15)

Staple food supplementation

- **Current coverage:** 18.4%
- **Maximum feasible coverage:** 100.0%
- **Estimated unit cost:** USD $0.35 (0.18 - 0.68)

Cost-Effectiveness Analysis

The estimated cost per Year of Life Disabled (YLD) averted for each intervention in **New Zealand** is given below, with interquartile ranges (IQR):

Iron supplementation (antenatal care)

- **Median Cost per YLD:** >USD 100k
- **IQR:** USD $68994 – >USD 100k

Iron supplementation (all women of reproductive age)

- **Median Cost per YLD:** >USD 100k
- **IQR:** USD $98398 – >USD 100k

Antenatal preventative IPTp-SP

- **Median Cost per YLD:** >USD 100k
- **IQR:** >USD 100k

Staple food supplementation

- **Median Cost per YLD:** USD $3972
- **IQR:** USD $2760 – $5840

Economic Considerations

Cost-effectiveness thresholds (CET, in USD per DALY averted) in **New Zealand** is as follows:

- **Estimates from Pichon-Riviere et al. (Lower Bound):** USD $35911
- **Estimates from Pichon-Riviere et al. (Upper Bound):** USD $42219
- **CET equal to per capita GDP:** USD $48528
- **CET equal to 2.3 (LMICs) or 1.7 (HICs) times per capita GDP:** USD $67939

⬆ Back to Table of Contents

Anaemia Reduction in Nicaragua

National Target

For our baseline CET = 1 x GDP per capita, the national target for anaemia in **Nicaragua** should be a reduction in prevalence of **19%**. This target is estimated with a 95% uncertainty interval from **0%** to **45%**.

For CET based on Pichon-Riviere, the national target for anaemia in **Nicaragua** should be a reduction in prevalence of **0%**. This target is estimated with a 95% uncertainty interval from **0%** to **41%**.

For a near-infinite CET, the national target for anaemia in **Nicaragua** should be a reduction in prevalence of **28%**. This target is estimated with a 95% uncertainty interval from **15%** to **46%**.

Input parameters

- Among women of reproductive age in **Nicaragua**, the prevalence of overall anaemia is **15.9%**
    - **Mild anaemia:** 9.9%
    - **Moderate anaemia:** 5.5%
    - **Severe anaemia:** 0.4%
- The prevalence of malaria is **0.1%**.

Intervention coverage and costs

Iron supplementation (antenatal care)

- **Current coverage:** 79.1%
- **Maximum feasible coverage:** 84.1%
- **Estimated unit cost:** USD $4.28 (2.07 - 7.68)

Iron supplementation (all women of reproductive age)

- **Current coverage:** 32.6%
- **Maximum feasible coverage:** 79.1%
- **Estimated unit cost:** USD $3.10 (1.56 - 7.22)

Antenatal preventative IPTp-SP

- **Current coverage:** 0.0%
- **Maximum feasible coverage:** 70.4%

- **Estimated unit cost:** USD $2.79 (1.46 - 4.96)

Staple food supplementation

- **Current coverage:** 3.2%
- **Maximum feasible coverage:** 100.0%
- **Estimated unit cost:** USD $0.46 (0.11 - 1.55)

Cost-Effectiveness Analysis

The estimated cost per Year of Life Disabled (YLD) averted for each intervention in **Nicaragua** is given below, with interquartile ranges (IQR):

Iron supplementation (antenatal care)

- **Median Cost per YLD:** USD $2943
- **IQR:** USD $1778 – >USD 100k

Iron supplementation (all women of reproductive age)

- **Median Cost per YLD:** USD $4581
- **IQR:** USD $3145 – $6710

Antenatal preventative IPTp-SP

- **Median Cost per YLD:** >USD 100k
- **IQR:** >USD 100k

Staple food supplementation

- **Median Cost per YLD:** USD $2287
- **IQR:** USD $1499 – $3669

Economic Considerations

Cost-effectiveness thresholds (CET, in USD per DALY averted) in **Nicaragua** is as follows:

- **Estimates from Pichon-Riviere et al. (Lower Bound):** USD $860
- **Estimates from Pichon-Riviere et al. (Upper Bound):** USD $1695

- **CET equal to per capita GDP:** USD $2530
- **CET equal to 2.3 (LMICs) or 1.7 (HICs) times per capita GDP:** USD $5820

⬆ Back to Table of Contents

Anaemia Reduction in Niger

National Target

For our baseline CET = 1 x GDP per capita, the national target for anaemia in **Niger** should be a reduction in prevalence of **2%**. This target is estimated with a 95% uncertainty interval from **0%** to **37%**.

For CET based on Pichon-Riviere, the national target for anaemia in **Niger** should be a reduction in prevalence of **0%**. This target is estimated with a 95% uncertainty interval from **0%** to **2%**.

For a near-infinite CET, the national target for anaemia in **Niger** should be a reduction in prevalence of **28%**. This target is estimated with a 95% uncertainty interval from **16%** to **45%**.

Input parameters

- Among women of reproductive age in **Niger**, the prevalence of overall anaemia is **51.8%**
  - **Mild anaemia:** 28.9%
  - **Moderate anaemia:** 21.6%
  - **Severe anaemia:** 1.3%
- The prevalence of malaria is **23.6%**.

Intervention coverage and costs

Iron supplementation (antenatal care)

- **Current coverage:** 83.4%
- **Maximum feasible coverage:** 93.8%
- **Estimated unit cost:** USD $2.33 (1.00 - 4.50)

Iron supplementation (all women of reproductive age)

- **Current coverage:** 34.4%

- **Maximum feasible coverage:** 93.8%
- **Estimated unit cost:** USD $2.51 (1.72 - 8.59)

Antenatal preventative IPTp-SP

- **Current coverage:** 55.8%
- **Maximum feasible coverage:** 93.8%
- **Estimated unit cost:** USD $1.12 (0.59 - 2.29)

Staple food supplementation

- **Current coverage:** 0.5%
- **Maximum feasible coverage:** 80.8%
- **Estimated unit cost:** USD $0.64 (0.10 - 1.97)

Cost-Effectiveness Analysis

The estimated cost per Year of Life Disabled (YLD) averted for each intervention in **Niger** is given below, with interquartile ranges (IQR):

Iron supplementation (antenatal care)

- **Median Cost per YLD:** USD $352
- **IQR:** USD $235 – >USD 100k

Iron supplementation (all women of reproductive age)

- **Median Cost per YLD:** USD $1239
- **IQR:** USD $861 – $1827

Antenatal preventative IPTp-SP

- **Median Cost per YLD:** USD $714
- **IQR:** USD $523 – $957

Staple food supplementation

- **Median Cost per YLD:** USD $1077
- **IQR:** USD $653 – $1771

Economic Considerations

Cost-effectiveness thresholds (CET, in USD per DALY averted) in **Niger** is as follows:

- **Estimates from Pichon-Riviere et al. (Lower Bound):** USD $49
- **Estimates from Pichon-Riviere et al. (Upper Bound):** USD $155
- **CET equal to per capita GDP:** USD $618
- **CET equal to 2.3 (LMICs) or 1.7 (HICs) times per capita GDP:** USD $1422

⬆ Back to Table of Contents

Anaemia Reduction in Nigeria

National Target

For our baseline CET = 1 x GDP per capita, the national target for anaemia in **Nigeria** should be a reduction in prevalence of **17%**. This target is estimated with a 95% uncertainty interval from **5%** to **29%**.

For CET based on Pichon-Riviere, the national target for anaemia in **Nigeria** should be a reduction in prevalence of **0%**. This target is estimated with a 95% uncertainty interval from **0%** to **20%**.

For a near-infinite CET, the national target for anaemia in **Nigeria** should be a reduction in prevalence of **18%**. This target is estimated with a 95% uncertainty interval from **10%** to **29%**.

Input parameters

- Among women of reproductive age in **Nigeria**, the prevalence of overall anaemia is **59.3%**
  - **Mild anaemia:** 29.6%
  - **Moderate anaemia:** 27.6%
  - **Severe anaemia:** 2.1%
- The prevalence of malaria is **28.2%**.

Intervention coverage and costs

Iron supplementation (antenatal care)

- **Current coverage:** 70.2%
- **Maximum feasible coverage:** 75.2%
- **Estimated unit cost:** USD $3.26 (1.49 - 6.03)

Iron supplementation (all women of reproductive age)

- **Current coverage:** 28.9%
- **Maximum feasible coverage:** 70.2%
- **Estimated unit cost:** USD $3.17 (2.01 - 10.00)

Antenatal preventative IPTp-SP

- **Current coverage:** 43.7%
- **Maximum feasible coverage:** 63.0%
- **Estimated unit cost:** USD $1.82 (0.94 - 3.36)

Staple food supplementation

- **Current coverage:** 5.1%
- **Maximum feasible coverage:** 52.4%
- **Estimated unit cost:** USD $0.41 (0.09 - 1.38)

Cost-Effectiveness Analysis

The estimated cost per Year of Life Disabled (YLD) averted for each intervention in **Nigeria** is given below, with interquartile ranges (IQR):

Iron supplementation (antenatal care)

- **Median Cost per YLD:** USD $507
- **IQR:** USD $313 – >USD 100k

Iron supplementation (all women of reproductive age)

- **Median Cost per YLD:** USD $1305
- **IQR:** USD $908 – $1924

Antenatal preventative IPTp-SP

- **Median Cost per YLD:** USD $1032
- **IQR:** USD $811 – $1335

Staple food supplementation

- **Median Cost per YLD:** USD $493
- **IQR:** USD $336 – $794

Economic Considerations

Cost-effectiveness thresholds (CET, in USD per DALY averted) in **Nigeria** is as follows:

- **Estimates from Pichon-Riviere et al. (Lower Bound):** USD $146
- **Estimates from Pichon-Riviere et al. (Upper Bound):** USD $276
- **CET equal to per capita GDP:** USD $1621
- **CET equal to 2.3 (LMICs) or 1.7 (HICs) times per capita GDP:** USD $3729

⬆ Back to Table of Contents

Anaemia Reduction in North Macedonia

National Target

For our baseline CET = 1 x GDP per capita, the national target for anaemia in **North Macedonia** should be a reduction in prevalence of **18%**. This target is estimated with a 95% uncertainty interval from **4%** to **34%**.

For CET based on Pichon-Riviere, the national target for anaemia in **North Macedonia** should be a reduction in prevalence of **13%**. This target is estimated with a 95% uncertainty interval from **3%** to **33%**.

For a near-infinite CET, the national target for anaemia in **North Macedonia** should be a reduction in prevalence of **21%**. This target is estimated with a 95% uncertainty interval from **11%** to **34%**.

Input parameters

- Among women of reproductive age in **North Macedonia**, the prevalence of overall anaemia is **20.4%**

    - **Mild anaemia:** 11.5%
    - **Moderate anaemia:** 8.1%
    - **Severe anaemia:** 0.8%
- There is no data on malaria prevalence; assume 0%.

Intervention coverage and costs

Iron supplementation (antenatal care)

- **Current coverage:** 54.0%
- **Maximum feasible coverage:** 96.0%
- **Estimated unit cost:** USD $11.35 (5.58 - 19.22)

Iron supplementation (all women of reproductive age)

- **Current coverage:** 22.2%
- **Maximum feasible coverage:** 96.0%
- **Estimated unit cost:** USD $7.47 (3.76 - 15.00)

Antenatal preventative IPTp-SP

- **Current coverage:** 0.0%
- **Maximum feasible coverage:** 96.0%
- **Estimated unit cost:** USD $8.66 (4.41 - 14.90)

Staple food supplementation

- **Current coverage:** 64.6%
- **Maximum feasible coverage:** 100.0%
- **Estimated unit cost:** USD $0.25 (0.16 - 0.38)

Cost-Effectiveness Analysis

The estimated cost per Year of Life Disabled (YLD) averted for each intervention in **North Macedonia** is given below, with interquartile ranges (IQR):

Iron supplementation (antenatal care)

- **Median Cost per YLD:** USD $3784
- **IQR:** USD $2832 – $4996

Iron supplementation (all women of reproductive age)

- **Median Cost per YLD:** USD $6856
- **IQR:** USD $4947 – $9505

Antenatal preventative IPTp-SP

- **Median Cost per YLD:** >USD 100k
- **IQR:** >USD 100k

Staple food supplementation

- **Median Cost per YLD:** USD $579
- **IQR:** USD $403 – $871

Economic Considerations

Cost-effectiveness thresholds (CET, in USD per DALY averted) in **North Macedonia** is as follows:

- **Estimates from Pichon-Riviere et al. (Lower Bound):** USD $4236
- **Estimates from Pichon-Riviere et al. (Upper Bound):** USD $5865
- **CET equal to per capita GDP:** USD $8146
- **CET equal to 2.3 (LMICs) or 1.7 (HICs) times per capita GDP:** USD $18737

⬆ Back to Table of Contents

Anaemia Reduction in Norway

National Target

For our baseline CET = 1 x GDP per capita, the national target for anaemia in **Norway** should be a reduction in prevalence of **16%**. This target is estimated with a 95% uncertainty interval from **6%** to **33%**.

For CET based on Pichon-Riviere, the national target for anaemia in **Norway** should be a reduction in prevalence of **16%**. This target is estimated with a 95% uncertainty interval from **6%** to **33%**.

For a near-infinite CET, the national target for anaemia in **Norway** should be a reduction in prevalence of **24%**. This target is estimated with a 95% uncertainty interval from **13%** to **40%**.

Input parameters

- Among women of reproductive age in **Norway**, the prevalence of overall anaemia is **6.9%**
    - **Mild anaemia:** 5.6%
    - **Moderate anaemia:** 1.3%
    - **Severe anaemia:** 0.0%
- There is no data on malaria prevalence; assume 0%.

Intervention coverage and costs

Iron supplementation (antenatal care)

- **Current coverage:** 81.0%
- **Maximum feasible coverage:** 100.0%
- **Estimated unit cost:** USD $131.66 (54.94 - 207.30)

Iron supplementation (all women of reproductive age)

- **Current coverage:** 33.4%
- **Maximum feasible coverage:** 100.0%
- **Estimated unit cost:** USD $83.08 (33.66 - 123.07)

Antenatal preventative IPTp-SP

- **Current coverage:** 0.0%
- **Maximum feasible coverage:** 100.0%
- **Estimated unit cost:** USD $72.83 (36.48 - 113.35)

Staple food supplementation

- **Current coverage:** 37.5%

- **Maximum feasible coverage:** 100.0%
- **Estimated unit cost:** USD $0.11 (0.01 - 0.25)

Cost-Effectiveness Analysis

The estimated cost per Year of Life Disabled (YLD) averted for each intervention in **Norway** is given below, with interquartile ranges (IQR):

Iron supplementation (antenatal care)

- **Median Cost per YLD:** >USD 100k
- **IQR:** >USD 100k

Iron supplementation (all women of reproductive age)

- **Median Cost per YLD:** >USD 100k
- **IQR:** >USD 100k

Antenatal preventative IPTp-SP

- **Median Cost per YLD:** >USD 100k
- **IQR:** >USD 100k

Staple food supplementation

- **Median Cost per YLD:** USD $2011
- **IQR:** USD $1228 – $3268

Economic Considerations

Cost-effectiveness thresholds (CET, in USD per DALY averted) in **Norway** is as follows:

- **Estimates from Pichon-Riviere et al. (Lower Bound):** USD $71249
- **Estimates from Pichon-Riviere et al. (Upper Bound):** USD $83564
- **CET equal to per capita GDP:** USD $87962
- **CET equal to 2.3 (LMICs) or 1.7 (HICs) times per capita GDP:** USD $123146

⬆ Back to Table of Contents

Anaemia Reduction in Oman

National Target

For our baseline CET = 1 x GDP per capita, the national target for anaemia in **Oman** should be a reduction in prevalence of **6%**. This target is estimated with a 95% uncertainty interval from **0%** to **21%**.

For CET based on Pichon-Riviere, the national target for anaemia in **Oman** should be a reduction in prevalence of **2%**. This target is estimated with a 95% uncertainty interval from **0%** to **14%**.

For a near-infinite CET, the national target for anaemia in **Oman** should be a reduction in prevalence of **10%**. This target is estimated with a 95% uncertainty interval from **3%** to **22%**.

Input parameters

- Among women of reproductive age in **Oman**, the prevalence of overall anaemia is **33.0%**
  - **Mild anaemia:** 24.2%
  - **Moderate anaemia:** 8.5%
  - **Severe anaemia:** 0.3%
- The prevalence of malaria is **0.0%**.

Intervention coverage and costs

Iron supplementation (antenatal care)

- **Current coverage:** 81.0%
- **Maximum feasible coverage:** 86.0%
- **Estimated unit cost:** USD $59.03 (18.50 - 95.39)

Iron supplementation (all women of reproductive age)

- **Current coverage:** 33.4%
- **Maximum feasible coverage:** 81.0%
- **Estimated unit cost:** USD $37.56 (10.71 - 55.21)

Antenatal preventative IPTp-SP

- **Current coverage:** 0.0%
- **Maximum feasible coverage:** 74.0%
- **Estimated unit cost:** USD $15.05 (7.59 - 24.18)

Staple food supplementation

- **Current coverage:** 81.9%
- **Maximum feasible coverage:** 89.6%
- **Estimated unit cost:** USD $0.66 (0.28 - 1.51)

Cost-Effectiveness Analysis

The estimated cost per Year of Life Disabled (YLD) averted for each intervention in **Oman** is given below, with interquartile ranges (IQR):

Iron supplementation (antenatal care)

- **Median Cost per YLD:** USD $25207
- **IQR:** USD $13718 – >USD 100k

Iron supplementation (all women of reproductive age)

- **Median Cost per YLD:** USD $25986
- **IQR:** USD $17962 – $37681

Antenatal preventative IPTp-SP

- **Median Cost per YLD:** >USD 100k
- **IQR:** >USD 100k

Staple food supplementation

- **Median Cost per YLD:** USD $2736
- **IQR:** USD $1474 – >USD 100k

Economic Considerations

Cost-effectiveness thresholds (CET, in USD per DALY averted) in **Oman** is as follows:

- **Estimates from Pichon-Riviere et al. (Lower Bound):** USD $6057
- **Estimates from Pichon-Riviere et al. (Upper Bound):** USD $7222
- **CET equal to per capita GDP:** USD $23295
- **CET equal to 2.3 (LMICs) or 1.7 (HICs) times per capita GDP:** USD $32613

⬆ Back to Table of Contents

Anaemia Reduction in Pakistan

National Target

For our baseline CET = 1 x GDP per capita, the national target for anaemia in **Pakistan** should be a reduction in prevalence of **6%**. This target is estimated with a 95% uncertainty interval from **1%** to **14%**.

For CET based on Pichon-Riviere, the national target for anaemia in **Pakistan** should be a reduction in prevalence of **2%**. This target is estimated with a 95% uncertainty interval from **0%** to **7%**.

For a near-infinite CET, the national target for anaemia in **Pakistan** should be a reduction in prevalence of **7%**. This target is estimated with a 95% uncertainty interval from **3%** to **14%**.

Input parameters

- Among women of reproductive age in **Pakistan**, the prevalence of overall anaemia is **56.4%**
  - **Mild anaemia:** 28.1%
  - **Moderate anaemia:** 25.8%
  - **Severe anaemia:** 2.6%
- The prevalence of malaria is **0.1%**.

Intervention coverage and costs

Iron supplementation (antenatal care)

- **Current coverage:** 60.4%
- **Maximum feasible coverage:** 86.9%
- **Estimated unit cost:** USD $2.55 (1.19 - 5.11)

Iron supplementation (all women of reproductive age)

- **Current coverage:** 24.9%
- **Maximum feasible coverage:** 86.9%
- **Estimated unit cost:** USD $2.58 (1.75 - 9.84)

Antenatal preventative IPTp-SP

- **Current coverage:** 0.0%
- **Maximum feasible coverage:** 86.9%
- **Estimated unit cost:** USD $1.33 (0.72 - 2.69)

Staple food supplementation

- **Current coverage:** 23.0%
- **Maximum feasible coverage:** 30.6%
- **Estimated unit cost:** USD $0.13 (0.03 - 0.39)

Cost-Effectiveness Analysis

The estimated cost per Year of Life Disabled (YLD) averted for each intervention in **Pakistan** is given below, with interquartile ranges (IQR):

Iron supplementation (antenatal care)

- **Median Cost per YLD:** USD $409
- **IQR:** USD $256 – >USD 100k

Iron supplementation (all women of reproductive age)

- **Median Cost per YLD:** USD $1197
- **IQR:** USD $779 – $1852

Antenatal preventative IPTp-SP

- **Median Cost per YLD:** >USD 100k
- **IQR:** USD $89573 – >USD 100k

Staple food supplementation

- **Median Cost per YLD:** USD $142
- **IQR:** USD $92 – $230

Economic Considerations

Cost-effectiveness thresholds (CET, in USD per DALY averted) in **Pakistan** is as follows:

- **Estimates from Pichon-Riviere et al. (Lower Bound):** USD $141
- **Estimates from Pichon-Riviere et al. (Upper Bound):** USD $281
- **CET equal to per capita GDP:** USD $1407
- **CET equal to 2.3 (LMICs) or 1.7 (HICs) times per capita GDP:** USD $3236

⬆ Back to Table of Contents

Anaemia Reduction in Palau

National Target

For our baseline CET = 1 x GDP per capita, the national target for anaemia in **Palau** should be a reduction in prevalence of **26%**. This target is estimated with a 95% uncertainty interval from **12%** to **46%**.

For CET based on Pichon-Riviere, the national target for anaemia in **Palau** should be a reduction in prevalence of **24%**. This target is estimated with a 95% uncertainty interval from **10%** to **42%**.

For a near-infinite CET, the national target for anaemia in **Palau** should be a reduction in prevalence of **26%**. This target is estimated with a 95% uncertainty interval from **14%** to **44%**.

Input parameters

- Among women of reproductive age in **Palau**, the prevalence of overall anaemia is **37.2%**
  - **Mild anaemia:** 23.2%
  - **Moderate anaemia:** 13.2%
  - **Severe anaemia:** 0.8%
- There is no data on malaria prevalence; assume 0%.

Intervention coverage and costs

Iron supplementation (antenatal care)

- **Current coverage:** 84.2%
- **Maximum feasible coverage:** 85.4%
- **Estimated unit cost:** USD $23.00 (11.43 - 36.57)

Iron supplementation (all women of reproductive age)

- **Current coverage:** 34.7%
- **Maximum feasible coverage:** 85.4%
- **Estimated unit cost:** USD $12.88 (6.47 - 23.06)

Antenatal preventative IPTp-SP

- **Current coverage:** 0.0%
- **Maximum feasible coverage:** 85.4%
- **Estimated unit cost:** USD $17.00 (8.58 - 27.11)

Staple food supplementation

- **Current coverage:** 15.5%
- **Maximum feasible coverage:** 100.0%
- **Estimated unit cost:** USD $0.67 (0.10 - 2.04)

Cost-Effectiveness Analysis

The estimated cost per Year of Life Disabled (YLD) averted for each intervention in **Palau** is given below, with interquartile ranges (IQR):

Iron supplementation (antenatal care)

- **Median Cost per YLD:** USD $8540
- **IQR:** USD $3897 – >USD 100k

Iron supplementation (all women of reproductive age)

- **Median Cost per YLD:** USD $6807
- **IQR:** USD $4878 – $9940

Antenatal preventative IPTp-SP

- **Median Cost per YLD:** >USD 100k
- **IQR:** >USD 100k

Staple food supplementation

- **Median Cost per YLD:** USD $1653
- **IQR:** USD $1044 – $2605

Economic Considerations

Cost-effectiveness thresholds (CET, in USD per DALY averted) in **Palau** is as follows:

- **Estimates from Pichon-Riviere et al. (Lower Bound):** USD $1457
- **Estimates from Pichon-Riviere et al. (Upper Bound):** USD $8448
- **CET equal to per capita GDP:** USD $14565
- **CET equal to 2.3 (LMICs) or 1.7 (HICs) times per capita GDP:** USD $33500

⬆ Back to Table of Contents

Anaemia Reduction in Panama

National Target

For our baseline CET = 1 x GDP per capita, the national target for anaemia in **Panama** should be a reduction in prevalence of **10%**. This target is estimated with a 95% uncertainty interval from **0%** to **28%**.

For CET based on Pichon-Riviere, the national target for anaemia in **Panama** should be a reduction in prevalence of **10%**. This target is estimated with a 95% uncertainty interval from **0%** to **24%**.

For a near-infinite CET, the national target for anaemia in **Panama** should be a reduction in prevalence of **19%**. This target is estimated with a 95% uncertainty interval from **10%** to **32%**.

Input parameters

- Among women of reproductive age in **Panama**, the prevalence of overall anaemia is **23.2%**

- Mild anaemia: 19.1%
  - **Moderate anaemia:** 4.0%
  - **Severe anaemia:** 0.1%
- The prevalence of malaria is **0.0%**.

Intervention coverage and costs

Iron supplementation (antenatal care)

- **Current coverage:** 81.0%
- **Maximum feasible coverage:** 100.0%
- **Estimated unit cost:** USD $55.72 (16.74 - 90.75)

Iron supplementation (all women of reproductive age)

- **Current coverage:** 33.4%
- **Maximum feasible coverage:** 100.0%
- **Estimated unit cost:** USD $32.53 (8.27 - 45.75)

Antenatal preventative IPTp-SP

- **Current coverage:** 0.0%
- **Maximum feasible coverage:** 100.0%
- **Estimated unit cost:** USD $11.94 (6.04 - 18.70)

Staple food supplementation

- **Current coverage:** 45.1%
- **Maximum feasible coverage:** 80.6%
- **Estimated unit cost:** USD $1.03 (0.50 - 2.28)

Cost-Effectiveness Analysis

The estimated cost per Year of Life Disabled (YLD) averted for each intervention in **Panama** is given below, with interquartile ranges (IQR):

Iron supplementation (antenatal care)

- **Median Cost per YLD:** USD $30314
- **IQR:** USD $19546 – $59208

Iron supplementation (all women of reproductive age)

- **Median Cost per YLD:** USD $40071
- **IQR:** USD $26689 – $64247

Antenatal preventative IPTp-SP

- **Median Cost per YLD:** >USD 100k
- **IQR:** >USD 100k

Staple food supplementation

- **Median Cost per YLD:** USD $5347
- **IQR:** USD $3484 – $8641

Economic Considerations

Cost-effectiveness thresholds (CET, in USD per DALY averted) in **Panama** is as follows:

- **Estimates from Pichon-Riviere et al. (Lower Bound):** USD $10077
- **Estimates from Pichon-Riviere et al. (Upper Bound):** USD $11944
- **CET equal to per capita GDP:** USD $18662
- **CET equal to 2.3 (LMICs) or 1.7 (HICs) times per capita GDP:** USD $26126

⬆ Back to Table of Contents

Anaemia Reduction in Papua New Guinea

National Target

For our baseline CET = 1 x GDP per capita, the national target for anaemia in **Papua New Guinea** should be a reduction in prevalence of **24%**. This target is estimated with a 95% uncertainty interval from **10%** to **42%**.

For CET based on Pichon-Riviere, the national target for anaemia in **Papua New Guinea** should be a reduction in prevalence of **1%**. This target is estimated with a 95% uncertainty interval from

0% to **38%**.

For a near-infinite CET, the national target for anaemia in **Papua New Guinea** should be a reduction in prevalence of **25%**. This target is estimated with a 95% uncertainty interval from **12%** to **43%**.

Input parameters

- Among women of reproductive age in **Papua New Guinea**, the prevalence of overall anaemia is **53.8%**
  - **Mild anaemia:** 35.1%
  - **Moderate anaemia:** 18.0%
  - **Severe anaemia:** 0.7%
- The prevalence of malaria is **3.6%**.

Intervention coverage and costs

Iron supplementation (antenatal care)

- **Current coverage:** 68.7%
- **Maximum feasible coverage:** 74.2%
- **Estimated unit cost:** USD $3.80 (1.87 - 7.14)

Iron supplementation (all women of reproductive age)

- **Current coverage:** 28.3%
- **Maximum feasible coverage:** 74.2%
- **Estimated unit cost:** USD $3.91 (2.49 - 11.19)

Antenatal preventative IPTp-SP

- **Current coverage:** 35.0%
- **Maximum feasible coverage:** 74.2%
- **Estimated unit cost:** USD $2.31 (1.23 - 4.24)

Staple food supplementation

- **Current coverage:** 13.9%

- **Maximum feasible coverage:** 95.2%
- **Estimated unit cost:** USD $0.37 (0.06 - 1.14)

Cost-Effectiveness Analysis

The estimated cost per Year of Life Disabled (YLD) averted for each intervention in **Papua New Guinea** is given below, with interquartile ranges (IQR):

Iron supplementation (antenatal care)

- **Median Cost per YLD:** USD $888
- **IQR:** USD $501 – >USD 100k

Iron supplementation (all women of reproductive age)

- **Median Cost per YLD:** USD $2075
- **IQR:** USD $1346 – $3020

Antenatal preventative IPTp-SP

- **Median Cost per YLD:** USD $8290
- **IQR:** USD $6249 – $11884

Staple food supplementation

- **Median Cost per YLD:** USD $557
- **IQR:** USD $343 – $872

Economic Considerations

Cost-effectiveness thresholds (CET, in USD per DALY averted) in **Papua New Guinea** is as follows:

- **Estimates from Pichon-Riviere et al. (Lower Bound):** USD $240
- **Estimates from Pichon-Riviere et al. (Upper Bound):** USD $479
- **CET equal to per capita GDP:** USD $2994
- **CET equal to 2.3 (LMICs) or 1.7 (HICs) times per capita GDP:** USD $6887

⬆ Back to Table of Contents

Anaemia Reduction in Paraguay

National Target

For our baseline CET = 1 x GDP per capita, the national target for anaemia in **Paraguay** should be a reduction in prevalence of **12%**. This target is estimated with a 95% uncertainty interval from **1%** to **22%**.

For CET based on Pichon-Riviere, the national target for anaemia in **Paraguay** should be a reduction in prevalence of **10%**. This target is estimated with a 95% uncertainty interval from **0%** to **20%**.

For a near-infinite CET, the national target for anaemia in **Paraguay** should be a reduction in prevalence of **13%**. This target is estimated with a 95% uncertainty interval from **6%** to **21%**.

Input parameters

- Among women of reproductive age in **Paraguay**, the prevalence of overall anaemia is **29.3%**
    - **Mild anaemia:** 18.0%
    - **Moderate anaemia:** 10.7%
    - **Severe anaemia:** 0.7%
- The prevalence of malaria is **0.0%**.

Intervention coverage and costs

Iron supplementation (antenatal care)

- **Current coverage:** 88.3%
- **Maximum feasible coverage:** 93.3%
- **Estimated unit cost:** USD $5.65 (2.71 - 9.60)

Iron supplementation (all women of reproductive age)

- **Current coverage:** 36.4%
- **Maximum feasible coverage:** 88.3%
- **Estimated unit cost:** USD $3.74 (1.89 - 8.43)

Antenatal preventative IPTp-SP

- **Current coverage:** 0.1%
- **Maximum feasible coverage:** 85.7%
- **Estimated unit cost:** USD $3.73 (1.93 - 6.40)

Staple food supplementation

- **Current coverage:** 8.1%
- **Maximum feasible coverage:** 28.7%
- **Estimated unit cost:** USD $1.96 (1.56 - 2.87)

Cost-Effectiveness Analysis

The estimated cost per Year of Life Disabled (YLD) averted for each intervention in **Paraguay** is given below, with interquartile ranges (IQR):

Iron supplementation (antenatal care)

- **Median Cost per YLD:** USD $1935
- **IQR:** USD $1095 – >USD 100k

Iron supplementation (all women of reproductive age)

- **Median Cost per YLD:** USD $2678
- **IQR:** USD $1832 – $3939

Antenatal preventative IPTp-SP

- **Median Cost per YLD:** >USD 100k
- **IQR:** >USD 100k

Staple food supplementation

- **Median Cost per YLD:** USD $3960
- **IQR:** USD $2786 – $5577

Economic Considerations

Cost-effectiveness thresholds (CET, in USD per DALY averted) in **Paraguay** is as follows:

- **Estimates from Pichon-Riviere et al. (Lower Bound):** USD $3130
- **Estimates from Pichon-Riviere et al. (Upper Bound):** USD $4257
- **CET equal to per capita GDP:** USD $6260
- **CET equal to 2.3 (LMICs) or 1.7 (HICs) times per capita GDP:** USD $14399

⬆ Back to Table of Contents

Anaemia Reduction in Peru

National Target

For our baseline CET = 1 x GDP per capita, the national target for anaemia in **Peru** should be a reduction in prevalence of **11%**. This target is estimated with a 95% uncertainty interval from **0%** to **24%**.

For CET based on Pichon-Riviere, the national target for anaemia in **Peru** should be a reduction in prevalence of **2%**. This target is estimated with a 95% uncertainty interval from **0%** to **19%**.

For a near-infinite CET, the national target for anaemia in **Peru** should be a reduction in prevalence of **15%**. This target is estimated with a 95% uncertainty interval from **8%** to **24%**.

Input parameters

- Among women of reproductive age in **Peru**, the prevalence of overall anaemia is **26.6%**
    - **Mild anaemia:** 20.2%
    - **Moderate anaemia:** 6.2%
    - **Severe anaemia:** 0.1%
- The prevalence of malaria is **0.0%**.

Intervention coverage and costs

Iron supplementation (antenatal care)

- **Current coverage:** 87.3%
- **Maximum feasible coverage:** 95.9%
- **Estimated unit cost:** USD $10.32 (5.04 - 16.54)

Iron supplementation (all women of reproductive age)

- **Current coverage:** 36.0%
- **Maximum feasible coverage:** 95.9%
- **Estimated unit cost:** USD $5.61 (2.82 - 11.15)

Antenatal preventative IPTp-SP

- **Current coverage:** 0.1%
- **Maximum feasible coverage:** 95.9%
- **Estimated unit cost:** USD $7.27 (3.70 - 11.67)

Staple food supplementation

- **Current coverage:** 0.0%
- **Maximum feasible coverage:** 26.4%
- **Estimated unit cost:** USD $0.96 (0.37 - 2.38)

Cost-Effectiveness Analysis

The estimated cost per Year of Life Disabled (YLD) averted for each intervention in **Peru** is given below, with interquartile ranges (IQR):

Iron supplementation (antenatal care)

- **Median Cost per YLD:** USD $5737
- **IQR:** USD $3382 – >USD 100k

Iron supplementation (all women of reproductive age)

- **Median Cost per YLD:** USD $7058
- **IQR:** USD $4849 – $10492

Antenatal preventative IPTp-SP

- **Median Cost per YLD:** >USD 100k
- **IQR:** >USD 100k

Staple food supplementation

- **Median Cost per YLD:** USD $4273
- **IQR:** USD $2877 – $6457

Economic Considerations

Cost-effectiveness thresholds (CET, in USD per DALY averted) in **Peru** is as follows:

- **Estimates from Pichon-Riviere et al. (Lower Bound):** USD $2882
- **Estimates from Pichon-Riviere et al. (Upper Bound):** USD $3895
- **CET equal to per capita GDP:** USD $7790
- **CET equal to 2.3 (LMICs) or 1.7 (HICs) times per capita GDP:** USD $17917

⬆ Back to Table of Contents

Anaemia Reduction in Philippines

National Target

For our baseline CET = 1 x GDP per capita, the national target for anaemia in **Philippines** should be a reduction in prevalence of **14%**. This target is estimated with a 95% uncertainty interval from **5%** to **23%**.

For CET based on Pichon-Riviere, the national target for anaemia in **Philippines** should be a reduction in prevalence of **6%**. This target is estimated with a 95% uncertainty interval from **0%** to **20%**.

For a near-infinite CET, the national target for anaemia in **Philippines** should be a reduction in prevalence of **14%**. This target is estimated with a 95% uncertainty interval from **7%** to **23%**.

Input parameters

- Among women of reproductive age in **Philippines**, the prevalence of overall anaemia is **30.1%**
  - **Mild anaemia:** 15.2%
  - **Moderate anaemia:** 13.1%
  - **Severe anaemia:** 1.8%
- The prevalence of malaria is **0.0%**.

Intervention coverage and costs

Iron supplementation (antenatal care)

- **Current coverage:** 86.2%
- **Maximum feasible coverage:** 91.2%
- **Estimated unit cost:** USD $3.92 (1.87 - 7.02)

Iron supplementation (all women of reproductive age)

- **Current coverage:** 35.5%
- **Maximum feasible coverage:** 86.2%
- **Estimated unit cost:** USD $3.01 (1.53 - 7.30)

Antenatal preventative IPTp-SP

- **Current coverage:** 35.0%
- **Maximum feasible coverage:** 85.9%
- **Estimated unit cost:** USD $2.43 (1.29 - 4.40)

Staple food supplementation

- **Current coverage:** 10.8%
- **Maximum feasible coverage:** 37.7%
- **Estimated unit cost:** USD $0.59 (0.10 - 1.84)

Cost-Effectiveness Analysis

The estimated cost per Year of Life Disabled (YLD) averted for each intervention in **Philippines** is given below, with interquartile ranges (IQR):

Iron supplementation (antenatal care)

- **Median Cost per YLD:** USD $1039
- **IQR:** USD $621 – >USD 100k

Iron supplementation (all women of reproductive age)

- **Median Cost per YLD:** USD $1719
- **IQR:** USD $1181 – $2525

Antenatal preventative IPTp-SP

- **Median Cost per YLD:** >USD 100k
- **IQR:** >USD 100k

Staple food supplementation

- **Median Cost per YLD:** USD $1211
- **IQR:** USD $775 – $1794

Economic Considerations

Cost-effectiveness thresholds (CET, in USD per DALY averted) in **Philippines** is as follows:

- **Estimates from Pichon-Riviere et al. (Lower Bound):** USD $596
- **Estimates from Pichon-Riviere et al. (Upper Bound):** USD $1192
- **CET equal to per capita GDP:** USD $3726
- **CET equal to 2.3 (LMICs) or 1.7 (HICs) times per capita GDP:** USD $8569

⬆ Back to Table of Contents

Anaemia Reduction in Poland

National Target

For our baseline CET = 1 x GDP per capita, the national target for anaemia in **Poland** should be a reduction in prevalence of **18%**. This target is estimated with a 95% uncertainty interval from **7%** to **37%**.

For CET based on Pichon-Riviere, the national target for anaemia in **Poland** should be a reduction in prevalence of **16%**. This target is estimated with a 95% uncertainty interval from **6%** to **32%**.

For a near-infinite CET, the national target for anaemia in **Poland** should be a reduction in prevalence of **24%**. This target is estimated with a 95% uncertainty interval from **12%** to **39%**.

Input parameters

- Among women of reproductive age in **Poland**, the prevalence of overall anaemia is **22.8%**
  - **Mild anaemia:** 15.8%
  - **Moderate anaemia:** 6.6%
  - **Severe anaemia:** 0.4%
- There is no data on malaria prevalence; assume 0%.

Intervention coverage and costs

Iron supplementation (antenatal care)

- **Current coverage:** 81.0%
- **Maximum feasible coverage:** 100.0%
- **Estimated unit cost:** USD $61.04 (19.40 - 100.03)

Iron supplementation (all women of reproductive age)

- **Current coverage:** 33.4%
- **Maximum feasible coverage:** 100.0%
- **Estimated unit cost:** USD $37.20 (10.61 - 53.96)

Antenatal preventative IPTp-SP

- **Current coverage:** 0.0%
- **Maximum feasible coverage:** 100.0%
- **Estimated unit cost:** USD $16.69 (8.41 - 27.08)

Staple food supplementation

- **Current coverage:** 38.7%
- **Maximum feasible coverage:** 100.0%
- **Estimated unit cost:** USD $0.06 (0.00 - 0.10)

Cost-Effectiveness Analysis

The estimated cost per Year of Life Disabled (YLD) averted for each intervention in **Poland** is given below, with interquartile ranges (IQR):

Iron supplementation (antenatal care)

- **Median Cost per YLD:** USD $22500
- **IQR:** USD $14862 – $35000

Iron supplementation (all women of reproductive age)

- **Median Cost per YLD:** USD $30459
- **IQR:** USD $21602 – $44173

Antenatal preventative IPTp-SP

- **Median Cost per YLD:** >USD 100k
- **IQR:** >USD 100k

Staple food supplementation

- **Median Cost per YLD:** USD $174
- **IQR:** USD $111 – $268

Economic Considerations

Cost-effectiveness thresholds (CET, in USD per DALY averted) in **Poland** is as follows:

- **Estimates from Pichon-Riviere et al. (Lower Bound):** USD $9951
- **Estimates from Pichon-Riviere et al. (Upper Bound):** USD $11720
- **CET equal to per capita GDP:** USD $22113
- **CET equal to 2.3 (LMICs) or 1.7 (HICs) times per capita GDP:** USD $30958

⬆ Back to Table of Contents

Anaemia Reduction in Portugal

National Target

For our baseline CET = 1 x GDP per capita, the national target for anaemia in **Portugal** should be a reduction in prevalence of **17%**. This target is estimated with a 95% uncertainty interval from **0%** to **34%**.

For CET based on Pichon-Riviere, the national target for anaemia in **Portugal** should be a reduction in prevalence of **17%**. This target is estimated with a 95% uncertainty interval from **0%** to **33%**.

For a near-infinite CET, the national target for anaemia in **Portugal** should be a reduction in prevalence of **26%**. This target is estimated with a 95% uncertainty interval from **14%** to **42%**.

Input parameters

- Among women of reproductive age in **Portugal**, the prevalence of overall anaemia is **9.3%**
  - **Mild anaemia:** 7.5%
  - **Moderate anaemia:** 1.7%
  - **Severe anaemia:** 0.0%
- There is no data on malaria prevalence; assume 0%.

Intervention coverage and costs

Iron supplementation (antenatal care)

- **Current coverage:** 81.0%
- **Maximum feasible coverage:** 100.0%
- **Estimated unit cost:** USD $57.36 (17.81 - 94.32)

Iron supplementation (all women of reproductive age)

- **Current coverage:** 33.4%
- **Maximum feasible coverage:** 100.0%
- **Estimated unit cost:** USD $36.05 (10.13 - 51.50)

Antenatal preventative IPTp-SP

- **Current coverage:** 0.0%
- **Maximum feasible coverage:** 100.0%
- **Estimated unit cost:** USD $16.27 (8.20 - 27.23)

Staple food supplementation

- **Current coverage:** 32.1%
- **Maximum feasible coverage:** 100.0%
- **Estimated unit cost:** USD $0.51 (0.34 - 0.84)

Cost-Effectiveness Analysis

The estimated cost per Year of Life Disabled (YLD) averted for each intervention in **Portugal** is given below, with interquartile ranges (IQR):

Iron supplementation (antenatal care)

- **Median Cost per YLD:** USD $88580
- **IQR:** USD $58339 – >USD 100k

Iron supplementation (all women of reproductive age)

- **Median Cost per YLD:** >USD 100k
- **IQR:** USD $79737 – >USD 100k

Antenatal preventative IPTp-SP

- **Median Cost per YLD:** >USD 100k
- **IQR:** >USD 100k

Staple food supplementation

- **Median Cost per YLD:** USD $7622
- **IQR:** USD $4902 – $12012

Economic Considerations

Cost-effectiveness thresholds (CET, in USD per DALY averted) in **Portugal** is as follows:

- **Estimates from Pichon-Riviere et al. (Lower Bound):** USD $19365
- **Estimates from Pichon-Riviere et al. (Upper Bound):** USD $22911
- **CET equal to per capita GDP:** USD $27275

- **CET equal to 2.3 (LMICs) or 1.7 (HICs) times per capita GDP:** USD $38185

⬆ Back to Table of Contents

Anaemia Reduction in Puerto Rico

National Target

For our baseline CET = 1 x GDP per capita, the national target for anaemia in **Puerto Rico** should be a reduction in prevalence of **19%**. This target is estimated with a 95% uncertainty interval from **7%** to **38%**.

For CET based on Pichon-Riviere, the national target for anaemia in **Puerto Rico** should be a reduction in prevalence of **17%**. This target is estimated with a 95% uncertainty interval from **7%** to **37%**.

For a near-infinite CET, the national target for anaemia in **Puerto Rico** should be a reduction in prevalence of **25%**. This target is estimated with a 95% uncertainty interval from **14%** to **41%**.

Input parameters

- Among women of reproductive age in **Puerto Rico**, the prevalence of overall anaemia is **25.2%**
  - **Mild anaemia:** 17.1%
  - **Moderate anaemia:** 7.7%
  - **Severe anaemia:** 0.4%
- There is no data on malaria prevalence; assume 0%.

Intervention coverage and costs

Iron supplementation (antenatal care)

- **Current coverage:** 81.0%
- **Maximum feasible coverage:** 100.0%
- **Estimated unit cost:** USD $107.05 (42.42 - 167.69)

Iron supplementation (all women of reproductive age)

- **Current coverage:** 33.4%

- **Maximum feasible coverage:** 100.0%
- **Estimated unit cost:** USD $64.54 (24.26 - 94.17)

Antenatal preventative IPTp-SP

- **Current coverage:** 0.0%
- **Maximum feasible coverage:** 100.0%
- **Estimated unit cost:** USD $50.58 (25.35 - 76.83)

Staple food supplementation

- **Current coverage:** 24.8%
- **Maximum feasible coverage:** 89.6%
- **Estimated unit cost:** USD $0.73 (0.19 - 2.02)

Cost-Effectiveness Analysis

The estimated cost per Year of Life Disabled (YLD) averted for each intervention in **Puerto Rico** is given below, with interquartile ranges (IQR):

Iron supplementation (antenatal care)

- **Median Cost per YLD:** >USD 100k
- **IQR:** >USD 100k

Iron supplementation (all women of reproductive age)

- **Median Cost per YLD:** USD $50367
- **IQR:** USD $35599 – $70531

Antenatal preventative IPTp-SP

- **Median Cost per YLD:** >USD 100k
- **IQR:** >USD 100k

Staple food supplementation

- **Median Cost per YLD:** USD $2701
- **IQR:** USD $1724 – $4445

Economic Considerations

Cost-effectiveness thresholds (CET, in USD per DALY averted) in **Puerto Rico** is as follows:

- **Estimates from Pichon-Riviere et al. (Lower Bound):** USD $26113
- **Estimates from Pichon-Riviere et al. (Upper Bound):** USD $25010
- **CET equal to per capita GDP:** USD $36779
- **CET equal to 2.3 (LMICs) or 1.7 (HICs) times per capita GDP:** USD $51491

⬆ Back to Table of Contents

Anaemia Reduction in Qatar

National Target

For our baseline CET = 1 x GDP per capita, the national target for anaemia in **Qatar** should be a reduction in prevalence of **28%**. This target is estimated with a 95% uncertainty interval from **13%** to **45%**.

For CET based on Pichon-Riviere, the national target for anaemia in **Qatar** should be a reduction in prevalence of **21%**. This target is estimated with a 95% uncertainty interval from **0%** to **40%**.

For a near-infinite CET, the national target for anaemia in **Qatar** should be a reduction in prevalence of **28%**. This target is estimated with a 95% uncertainty interval from **16%** to **45%**.

Input parameters

- Among women of reproductive age in **Qatar**, the prevalence of overall anaemia is **30.5%**
  - **Mild anaemia:** 21.7%
  - **Moderate anaemia:** 8.5%
  - **Severe anaemia:** 0.3%
- There is no data on malaria prevalence; assume 0%.

Intervention coverage and costs

Iron supplementation (antenatal care)

- **Current coverage:** 81.0%

- **Maximum feasible coverage:** 100.0%
- **Estimated unit cost:** USD $100.10 (39.01 - 157.29)

Iron supplementation (all women of reproductive age)

- **Current coverage:** 33.4%
- **Maximum feasible coverage:** 100.0%
- **Estimated unit cost:** USD $63.02 (23.46 - 93.12)

Antenatal preventative IPTp-SP

- **Current coverage:** 0.0%
- **Maximum feasible coverage:** 100.0%
- **Estimated unit cost:** USD $45.81 (22.97 - 70.32)

Staple food supplementation

- **Current coverage:** 16.7%
- **Maximum feasible coverage:** 100.0%
- **Estimated unit cost:** USD $2.69 (2.18 - 3.90)

Cost-Effectiveness Analysis

The estimated cost per Year of Life Disabled (YLD) averted for each intervention in **Qatar** is given below, with interquartile ranges (IQR):

Iron supplementation (antenatal care)

- **Median Cost per YLD:** USD $29044
- **IQR:** USD $20140 – $46626

Iron supplementation (all women of reproductive age)

- **Median Cost per YLD:** USD $43522
- **IQR:** USD $30450 – $63791

Antenatal preventative IPTp-SP

- **Median Cost per YLD:** >USD 100k

- **IQR:** >USD 100k

Staple food supplementation

- **Median Cost per YLD:** USD $10840
- **IQR:** USD $7781 – $15829

Economic Considerations

Cost-effectiveness thresholds (CET, in USD per DALY averted) in **Qatar** is as follows:

- **Estimates from Pichon-Riviere et al. (Lower Bound):** USD $19286
- **Estimates from Pichon-Riviere et al. (Upper Bound):** USD $22792
- **CET equal to per capita GDP:** USD $87661
- **CET equal to 2.3 (LMICs) or 1.7 (HICs) times per capita GDP:** USD $122726

⬆ Back to Table of Contents

Anaemia Reduction in Romania

National Target

For our baseline CET = 1 x GDP per capita, the national target for anaemia in **Romania** should be a reduction in prevalence of **16%**. This target is estimated with a 95% uncertainty interval from **6%** to **33%**.

For CET based on Pichon-Riviere, the national target for anaemia in **Romania** should be a reduction in prevalence of **14%**. This target is estimated with a 95% uncertainty interval from **5%** to **29%**.

For a near-infinite CET, the national target for anaemia in **Romania** should be a reduction in prevalence of **23%**. This target is estimated with a 95% uncertainty interval from **12%** to **37%**.

Input parameters

- Among women of reproductive age in **Romania**, the prevalence of overall anaemia is **23.0%**
    - **Mild anaemia:** 15.2%
    - **Moderate anaemia:** 7.3%
    - **Severe anaemia:** 0.4%

- There is no data on malaria prevalence; assume 0%.

Intervention coverage and costs

Iron supplementation (antenatal care)

- **Current coverage:** 81.0%
- **Maximum feasible coverage:** 100.0%
- **Estimated unit cost:** USD $57.17 (17.48 - 97.35)

Iron supplementation (all women of reproductive age)

- **Current coverage:** 33.4%
- **Maximum feasible coverage:** 100.0%
- **Estimated unit cost:** USD $36.32 (10.15 - 56.22)

Antenatal preventative IPTp-SP

- **Current coverage:** 0.0%
- **Maximum feasible coverage:** 100.0%
- **Estimated unit cost:** USD $14.11 (7.12 - 26.57)

Staple food supplementation

- **Current coverage:** 24.0%
- **Maximum feasible coverage:** 77.5%
- **Estimated unit cost:** USD $0.60 (0.45 - 0.89)

Cost-Effectiveness Analysis

The estimated cost per Year of Life Disabled (YLD) averted for each intervention in **Romania** is given below, with interquartile ranges (IQR):

Iron supplementation (antenatal care)

- **Median Cost per YLD:** USD $20040
- **IQR:** USD $13535 – $31992

Iron supplementation (all women of reproductive age)

- **Median Cost per YLD:** USD $28946
- **IQR:** USD $20018 – $41784

Antenatal preventative IPTp-SP

- **Median Cost per YLD:** >USD 100k
- **IQR:** >USD 100k

Staple food supplementation

- **Median Cost per YLD:** USD $2081
- **IQR:** USD $1480 – $2997

Economic Considerations

Cost-effectiveness thresholds (CET, in USD per DALY averted) in **Romania** is as follows:

- **Estimates from Pichon-Riviere et al. (Lower Bound):** USD $7184
- **Estimates from Pichon-Riviere et al. (Upper Bound):** USD $8473
- **CET equal to per capita GDP:** USD $18419
- **CET equal to 2.3 (LMICs) or 1.7 (HICs) times per capita GDP:** USD $25787

⬆ Back to Table of Contents

Anaemia Reduction in Russia

National Target

For our baseline CET = 1 x GDP per capita, the national target for anaemia in **Russia** should be a reduction in prevalence of **17%**. This target is estimated with a 95% uncertainty interval from **3%** to **33%**.

For CET based on Pichon-Riviere, the national target for anaemia in **Russia** should be a reduction in prevalence of **10%**. This target is estimated with a 95% uncertainty interval from **2%** to **28%**.

For a near-infinite CET, the national target for anaemia in **Russia** should be a reduction in prevalence of **20%**. This target is estimated with a 95% uncertainty interval from **10%** to **33%**.

Input parameters

- Among women of reproductive age in **Russia**, the prevalence of overall anaemia is **21.8%**
  - **Mild anaemia:** 13.5%
  - **Moderate anaemia:** 7.6%
  - **Severe anaemia:** 0.6%
- There is no data on malaria prevalence; assume 0%.

Intervention coverage and costs

Iron supplementation (antenatal care)

- **Current coverage:** NaN%
- **Maximum feasible coverage:** NaN%
- **Estimated unit cost:** USD $NaN (NaN - NaN)

Iron supplementation (all women of reproductive age)

- **Current coverage:** NaN%
- **Maximum feasible coverage:** NaN%
- **Estimated unit cost:** USD $NaN (NaN - NaN)

Antenatal preventative IPTp-SP

- **Current coverage:** NaN%
- **Maximum feasible coverage:** NaN%
- **Estimated unit cost:** USD $NaN (NaN - NaN)

Staple food supplementation

- **Current coverage:** NaN%
- **Maximum feasible coverage:** NaN%
- **Estimated unit cost:** USD $NaN (NaN - NaN)

Cost-Effectiveness Analysis

The estimated cost per Year of Life Disabled (YLD) averted for each intervention in **Russia** is given below, with interquartile ranges (IQR):

Iron supplementation (antenatal care)

- **Median Cost per YLD:** USD $6579
- **IQR:** USD $4822 – $9084

Iron supplementation (all women of reproductive age)

- **Median Cost per YLD:** USD $11437
- **IQR:** USD $8095 – $16115

Antenatal preventative IPTp-SP

- **Median Cost per YLD:** >USD 100k
- **IQR:** >USD 100k

Staple food supplementation

- **Median Cost per YLD:** USD $237
- **IQR:** USD $158 – $366

Economic Considerations

Cost-effectiveness thresholds (CET, in USD per DALY averted) in **Russia** is as follows:

- **Estimates from Pichon-Riviere et al. (Lower Bound):** USD $NaN
- **Estimates from Pichon-Riviere et al. (Upper Bound):** USD $NaN
- **CET equal to per capita GDP:** USD $NaN
- **CET equal to 2.3 (LMICs) or 1.7 (HICs) times per capita GDP:** USD $NaN

⬆ Back to Table of Contents

Anaemia Reduction in Rwanda

National Target

For our baseline CET = 1 x GDP per capita, the national target for anaemia in **Rwanda** should be a reduction in prevalence of **0%**. This target is estimated with a 95% uncertainty interval from **0%** to **22%**.

For CET based on Pichon-Riviere, the national target for anaemia in **Rwanda** should be a reduction in prevalence of **0%**. This target is estimated with a 95% uncertainty interval from **0%** to **0%**.

For a near-infinite CET, the national target for anaemia in **Rwanda** should be a reduction in prevalence of **19%**. This target is estimated with a 95% uncertainty interval from **10%** to **30%**.

Input parameters

- Among women of reproductive age in **Rwanda**, the prevalence of overall anaemia is **18.5%**
  - **Mild anaemia:** 11.3%
  - **Moderate anaemia:** 6.7%
  - **Severe anaemia:** 0.5%
- The prevalence of malaria is **2.5%**.

Intervention coverage and costs

Iron supplementation (antenatal care)

- **Current coverage:** 82.3%
- **Maximum feasible coverage:** 97.5%
- **Estimated unit cost:** USD $2.44 (0.98 - 4.27)

Iron supplementation (all women of reproductive age)

- **Current coverage:** 33.9%
- **Maximum feasible coverage:** 97.5%
- **Estimated unit cost:** USD $2.27 (1.13 - 5.33)

Antenatal preventative IPTp-SP

- **Current coverage:** 17.2%
- **Maximum feasible coverage:** 97.5%
- **Estimated unit cost:** USD $1.23 (0.64 - 2.42)

Staple food supplementation

- **Current coverage:** 0.4%
- **Maximum feasible coverage:** 49.4%
- **Estimated unit cost:** USD $0.41 (0.10 - 1.41)

Cost-Effectiveness Analysis

The estimated cost per Year of Life Disabled (YLD) averted for each intervention in **Rwanda** is given below, with interquartile ranges (IQR):

Iron supplementation (antenatal care)

- **Median Cost per YLD:** USD $1400
- **IQR:** USD $819 – >USD 100k

Iron supplementation (all women of reproductive age)

- **Median Cost per YLD:** USD $2769
- **IQR:** USD $1889 – $4016

Antenatal preventative IPTp-SP

- **Median Cost per YLD:** USD $19446
- **IQR:** USD $14815 – $25920

Staple food supplementation

- **Median Cost per YLD:** USD $1848
- **IQR:** USD $1195 – $2972

Economic Considerations

Cost-effectiveness thresholds (CET, in USD per DALY averted) in **Rwanda** is as follows:

- **Estimates from Pichon-Riviere et al. (Lower Bound):** USD $100
- **Estimates from Pichon-Riviere et al. (Upper Bound):** USD $310
- **CET equal to per capita GDP:** USD $1000

- **CET equal to 2.3 (LMICs) or 1.7 (HICs) times per capita GDP:** USD $2301

⬆ Back to Table of Contents

Anaemia Reduction in Samoa

National Target

For our baseline CET = 1 x GDP per capita, the national target for anaemia in **Samoa** should be a reduction in prevalence of **28%**. This target is estimated with a 95% uncertainty interval from **13%** to **48%**.

For CET based on Pichon-Riviere, the national target for anaemia in **Samoa** should be a reduction in prevalence of **26%**. This target is estimated with a 95% uncertainty interval from **1%** to **45%**.

For a near-infinite CET, the national target for anaemia in **Samoa** should be a reduction in prevalence of **29%**. This target is estimated with a 95% uncertainty interval from **15%** to **47%**.

Input parameters

- Among women of reproductive age in **Samoa**, the prevalence of overall anaemia is **27.2%**
  - **Mild anaemia:** 13.2%
  - **Moderate anaemia:** 12.4%
  - **Severe anaemia:** 1.6%
- There is no data on malaria prevalence; assume 0%.

Intervention coverage and costs

Iron supplementation (antenatal care)

- **Current coverage:** 84.2%
- **Maximum feasible coverage:** 89.2%
- **Estimated unit cost:** USD $5.31 (2.61 - 9.28)

Iron supplementation (all women of reproductive age)

- **Current coverage:** 34.7%
- **Maximum feasible coverage:** 84.2%

- **Estimated unit cost:** USD $4.29 (2.16 - 9.07)

Antenatal preventative IPTp-SP

- **Current coverage:** 35.0%
- **Maximum feasible coverage:** 70.0%
- **Estimated unit cost:** USD $3.55 (1.85 - 6.09)

Staple food supplementation

- **Current coverage:** 2.2%
- **Maximum feasible coverage:** 100.0%
- **Estimated unit cost:** USD $0.37 (0.05 - 1.14)

Cost-Effectiveness Analysis

The estimated cost per Year of Life Disabled (YLD) averted for each intervention in **Samoa** is given below, with interquartile ranges (IQR):

Iron supplementation (antenatal care)

- **Median Cost per YLD:** USD $1612
- **IQR:** USD $927 – >USD 100k

Iron supplementation (all women of reproductive age)

- **Median Cost per YLD:** USD $2538
- **IQR:** USD $1727 – $3536

Antenatal preventative IPTp-SP

- **Median Cost per YLD:** >USD 100k
- **IQR:** >USD 100k

Staple food supplementation

- **Median Cost per YLD:** USD $832
- **IQR:** USD $527 – $1284

Economic Considerations

Cost-effectiveness thresholds (CET, in USD per DALY averted) in **Samoa** is as follows:

- **Estimates from Pichon-Riviere et al. (Lower Bound):** USD $1863
- **Estimates from Pichon-Riviere et al. (Upper Bound):** USD $2525
- **CET equal to per capita GDP:** USD $4139
- **CET equal to 2.3 (LMICs) or 1.7 (HICs) times per capita GDP:** USD $9520

⬆ Back to Table of Contents

Anaemia Reduction in San Marino

National Target

For our baseline CET = 1 x GDP per capita, the national target for anaemia in **San Marino** should be a reduction in prevalence of **16%**. This target is estimated with a 95% uncertainty interval from **6%** to **34%**.

For CET based on Pichon-Riviere, the national target for anaemia in **San Marino** should be a reduction in prevalence of **16%**. This target is estimated with a 95% uncertainty interval from **6%** to **33%**.

For a near-infinite CET, the national target for anaemia in **San Marino** should be a reduction in prevalence of **25%**. This target is estimated with a 95% uncertainty interval from **13%** to **41%**.

Input parameters

- Among women of reproductive age in **San Marino**, the prevalence of overall anaemia is **7.8%**
    - **Mild anaemia:** 6.3%
    - **Moderate anaemia:** 1.4%
    - **Severe anaemia:** 0.0%
- There is no data on malaria prevalence; assume 0%.

Intervention coverage and costs

Iron supplementation (antenatal care)

- **Current coverage:** 81.0%

- **Maximum feasible coverage:** 100.0%
- **Estimated unit cost:** USD $100.82 (39.43 - 158.62)

Iron supplementation (all women of reproductive age)

- **Current coverage:** 33.4%
- **Maximum feasible coverage:** 100.0%
- **Estimated unit cost:** USD $63.40 (23.61 - 95.23)

Antenatal preventative IPTp-SP

- **Current coverage:** 0.0%
- **Maximum feasible coverage:** 100.0%
- **Estimated unit cost:** USD $47.04 (23.58 - 73.07)

Staple food supplementation

- **Current coverage:** 35.7%
- **Maximum feasible coverage:** 100.0%
- **Estimated unit cost:** USD $0.25 (0.14 - 0.40)

Cost-Effectiveness Analysis

The estimated cost per Year of Life Disabled (YLD) averted for each intervention in **San Marino** is given below, with interquartile ranges (IQR):

Iron supplementation (antenatal care)

- **Median Cost per YLD:** >USD 100k
- **IQR:** >USD 100k

Iron supplementation (all women of reproductive age)

- **Median Cost per YLD:** >USD 100k
- **IQR:** >USD 100k

Antenatal preventative IPTp-SP

- **Median Cost per YLD:** >USD 100k

- **IQR:** >USD 100k

Staple food supplementation

- **Median Cost per YLD:** USD $3873
- **IQR:** USD $2605 – $5972

Economic Considerations

Cost-effectiveness thresholds (CET, in USD per DALY averted) in **San Marino** is as follows:

- **Estimates from Pichon-Riviere et al. (Lower Bound):** USD $24742
- **Estimates from Pichon-Riviere et al. (Upper Bound):** USD $37388
- **CET equal to per capita GDP:** USD $54982
- **CET equal to 2.3 (LMICs) or 1.7 (HICs) times per capita GDP:** USD $76975

⬆ Back to Table of Contents

Anaemia Reduction in Saudi Arabia

National Target

For our baseline CET = 1 x GDP per capita, the national target for anaemia in **Saudi Arabia** should be a reduction in prevalence of **18%**. This target is estimated with a 95% uncertainty interval from **7%** to **34%**.

For CET based on Pichon-Riviere, the national target for anaemia in **Saudi Arabia** should be a reduction in prevalence of **14%**. This target is estimated with a 95% uncertainty interval from **5%** to **31%**.

For a near-infinite CET, the national target for anaemia in **Saudi Arabia** should be a reduction in prevalence of **19%**. This target is estimated with a 95% uncertainty interval from **9%** to **34%**.

Input parameters

- Among women of reproductive age in **Saudi Arabia**, the prevalence of overall anaemia is **33.0%**
  - **Mild anaemia:** 21.9%
  - **Moderate anaemia:** 10.6%

- Severe anaemia: 0.5%

- The prevalence of malaria is **0.0%**.

Intervention coverage and costs

Iron supplementation (antenatal care)

- **Current coverage:** 81.0%
- **Maximum feasible coverage:** 86.0%
- **Estimated unit cost:** USD $58.34 (18.20 - 93.81)

Iron supplementation (all women of reproductive age)

- **Current coverage:** 33.4%
- **Maximum feasible coverage:** 81.0%
- **Estimated unit cost:** USD $36.95 (10.37 - 54.81)

Antenatal preventative IPTp-SP

- **Current coverage:** 0.0%
- **Maximum feasible coverage:** 80.0%
- **Estimated unit cost:** USD $14.61 (7.37 - 23.53)

Staple food supplementation

- **Current coverage:** 48.5%
- **Maximum feasible coverage:** 100.0%
- **Estimated unit cost:** USD $0.69 (0.40 - 1.31)

Cost-Effectiveness Analysis

The estimated cost per Year of Life Disabled (YLD) averted for each intervention in **Saudi Arabia** is given below, with interquartile ranges (IQR):

Iron supplementation (antenatal care)

- **Median Cost per YLD:** USD $17458
- **IQR:** USD $10103 – >USD 100k

Iron supplementation (all women of reproductive age)

- **Median Cost per YLD:** USD $20463
- **IQR:** USD $13861 – $28941

Antenatal preventative IPTp-SP

- **Median Cost per YLD:** >USD 100k
- **IQR:** >USD 100k

Staple food supplementation

- **Median Cost per YLD:** USD $1270
- **IQR:** USD $864 – $1920

Economic Considerations

Cost-effectiveness thresholds (CET, in USD per DALY averted) in **Saudi Arabia** is as follows:

- **Estimates from Pichon-Riviere et al. (Lower Bound):** USD $11269
- **Estimates from Pichon-Riviere et al. (Upper Bound):** USD $13292
- **CET equal to per capita GDP:** USD $28895
- **CET equal to 2.3 (LMICs) or 1.7 (HICs) times per capita GDP:** USD $40453

⬆ Back to Table of Contents

Anaemia Reduction in Senegal

National Target

For our baseline CET = 1 x GDP per capita, the national target for anaemia in **Senegal** should be a reduction in prevalence of **19%**. This target is estimated with a 95% uncertainty interval from **7%** to **32%**.

For CET based on Pichon-Riviere, the national target for anaemia in **Senegal** should be a reduction in prevalence of **10%**. This target is estimated with a 95% uncertainty interval from **0%** to **26%**.

For a near-infinite CET, the national target for anaemia in **Senegal** should be a reduction in prevalence of **20%**. This target is estimated with a 95% uncertainty interval from **10%** to **32%**.

Input parameters

- Among women of reproductive age in **Senegal**, the prevalence of overall anaemia is **67.7%**
  - **Mild anaemia:** 35.6%
  - **Moderate anaemia:** 30.4%
  - **Severe anaemia:** 1.7%
- The prevalence of malaria is **4.2%**.

Intervention coverage and costs

Iron supplementation (antenatal care)

- **Current coverage:** 97.0%
- **Maximum feasible coverage:** 97.9%
- **Estimated unit cost:** USD $2.66 (1.14 - 4.88)

Iron supplementation (all women of reproductive age)

- **Current coverage:** 40.0%
- **Maximum feasible coverage:** 97.9%
- **Estimated unit cost:** USD $2.75 (1.35 - 6.76)

Antenatal preventative IPTp-SP

- **Current coverage:** 64.4%
- **Maximum feasible coverage:** 97.9%
- **Estimated unit cost:** USD $1.39 (0.73 - 2.72)

Staple food supplementation

- **Current coverage:** 8.5%
- **Maximum feasible coverage:** 57.1%
- **Estimated unit cost:** USD $0.52 (0.09 - 1.63)

Cost-Effectiveness Analysis

The estimated cost per Year of Life Disabled (YLD) averted for each intervention in **Senegal** is given below, with interquartile ranges (IQR):

Iron supplementation (antenatal care)

- **Median Cost per YLD:** >USD 100k
- **IQR:** USD $231 – >USD 100k

Iron supplementation (all women of reproductive age)

- **Median Cost per YLD:** USD $786
- **IQR:** USD $550 – $1156

Antenatal preventative IPTp-SP

- **Median Cost per YLD:** USD $2871
- **IQR:** USD $1722 – >USD 100k

Staple food supplementation

- **Median Cost per YLD:** USD $526
- **IQR:** USD $335 – $835

Economic Considerations

Cost-effectiveness thresholds (CET, in USD per DALY averted) in **Senegal** is as follows:

- **Estimates from Pichon-Riviere et al. (Lower Bound):** USD $279
- **Estimates from Pichon-Riviere et al. (Upper Bound):** USD $524
- **CET equal to per capita GDP:** USD $1746
- **CET equal to 2.3 (LMICs) or 1.7 (HICs) times per capita GDP:** USD $4016

⬆ Back to Table of Contents

Anaemia Reduction in Serbia

National Target

For our baseline CET = 1 x GDP per capita, the national target for anaemia in **Serbia** should be a reduction in prevalence of **17%**. This target is estimated with a 95% uncertainty interval from **4%** to **34%**.

For CET based on Pichon-Riviere, the national target for anaemia in **Serbia** should be a reduction in prevalence of **13%**. This target is estimated with a 95% uncertainty interval from **3%** to **33%**.

For a near-infinite CET, the national target for anaemia in **Serbia** should be a reduction in prevalence of **21%**. This target is estimated with a 95% uncertainty interval from **12%** to **35%**.

Input parameters

- Among women of reproductive age in **Serbia**, the prevalence of overall anaemia is **22.1%**
  - **Mild anaemia:** 14.6%
  - **Moderate anaemia:** 7.1%
  - **Severe anaemia:** 0.4%
- There is no data on malaria prevalence; assume 0%.

Intervention coverage and costs

Iron supplementation (antenatal care)

- **Current coverage:** 54.0%
- **Maximum feasible coverage:** 97.0%
- **Estimated unit cost:** USD $13.77 (6.80 - 28.18)

Iron supplementation (all women of reproductive age)

- **Current coverage:** 22.2%
- **Maximum feasible coverage:** 97.0%
- **Estimated unit cost:** USD $8.62 (4.35 - 22.08)

Antenatal preventative IPTp-SP

- **Current coverage:** 0.0%
- **Maximum feasible coverage:** 97.0%
- **Estimated unit cost:** USD $10.57 (5.36 - 23.00)

Staple food supplementation

- **Current coverage:** 38.5%
- **Maximum feasible coverage:** 72.7%
- **Estimated unit cost:** USD $0.60 (0.45 - 0.87)

Cost-Effectiveness Analysis

The estimated cost per Year of Life Disabled (YLD) averted for each intervention in **Serbia** is given below, with interquartile ranges (IQR):

Iron supplementation (antenatal care)

- **Median Cost per YLD:** USD $6041
- **IQR:** USD $4283 – $8506

Iron supplementation (all women of reproductive age)

- **Median Cost per YLD:** USD $10758
- **IQR:** USD $7288 – $15649

Antenatal preventative IPTp-SP

- **Median Cost per YLD:** >USD 100k
- **IQR:** >USD 100k

Staple food supplementation

- **Median Cost per YLD:** USD $1878
- **IQR:** USD $1385 – $2799

Economic Considerations

Cost-effectiveness thresholds (CET, in USD per DALY averted) in **Serbia** is as follows:

- **Estimates from Pichon-Riviere et al. (Lower Bound):** USD $6930
- **Estimates from Pichon-Riviere et al. (Upper Bound):** USD $9543
- **CET equal to per capita GDP:** USD $11361

- **CET equal to 2.3 (LMICs) or 1.7 (HICs) times per capita GDP:** USD $26130

⬆ Back to Table of Contents

Anaemia Reduction in Seychelles

National Target

For our baseline CET = 1 x GDP per capita, the national target for anaemia in **Seychelles** should be a reduction in prevalence of **25%**. This target is estimated with a 95% uncertainty interval from **9%** to **43%**.

For CET based on Pichon-Riviere, the national target for anaemia in **Seychelles** should be a reduction in prevalence of **20%**. This target is estimated with a 95% uncertainty interval from **7%** to **39%**.

For a near-infinite CET, the national target for anaemia in **Seychelles** should be a reduction in prevalence of **28%**. This target is estimated with a 95% uncertainty interval from **16%** to **45%**.

Input parameters

- Among women of reproductive age in **Seychelles**, the prevalence of overall anaemia is **35.1%**
    - **Mild anaemia:** 22.5%
    - **Moderate anaemia:** 12.0%
    - **Severe anaemia:** 0.6%
- There is no data on malaria prevalence; assume 0%.

Intervention coverage and costs

Iron supplementation (antenatal care)

- **Current coverage:** 81.0%
- **Maximum feasible coverage:** 100.0%
- **Estimated unit cost:** USD $56.86 (17.54 - 91.67)

Iron supplementation (all women of reproductive age)

- **Current coverage:** 33.4%

- **Maximum feasible coverage:** 100.0%
- **Estimated unit cost:** USD $33.69 (8.96 - 45.74)

Antenatal preventative IPTp-SP

- **Current coverage:** 0.0%
- **Maximum feasible coverage:** 100.0%
- **Estimated unit cost:** USD $14.73 (7.43 - 22.86)

Staple food supplementation

- **Current coverage:** 18.1%
- **Maximum feasible coverage:** 100.0%
- **Estimated unit cost:** USD $0.74 (0.36 - 1.62)

Cost-Effectiveness Analysis

The estimated cost per Year of Life Disabled (YLD) averted for each intervention in **Seychelles** is given below, with interquartile ranges (IQR):

Iron supplementation (antenatal care)

- **Median Cost per YLD:** USD $11891
- **IQR:** USD $7832 – $19200

Iron supplementation (all women of reproductive age)

- **Median Cost per YLD:** USD $15818
- **IQR:** USD $10676 – $22212

Antenatal preventative IPTp-SP

- **Median Cost per YLD:** >USD 100k
- **IQR:** >USD 100k

Staple food supplementation

- **Median Cost per YLD:** USD $1640
- **IQR:** USD $1096 – $2457

Economic Considerations

Cost-effectiveness thresholds (CET, in USD per DALY averted) in **Seychelles** is as follows:

- **Estimates from Pichon-Riviere et al. (Lower Bound):** USD $5900
- **Estimates from Pichon-Riviere et al. (Upper Bound):** USD $6973
- **CET equal to per capita GDP:** USD $17879
- **CET equal to 2.3 (LMICs) or 1.7 (HICs) times per capita GDP:** USD $25031

⬆ Back to Table of Contents

Anaemia Reduction in Sierra Leone

National Target

For our baseline CET = 1 x GDP per capita, the national target for anaemia in **Sierra Leone** should be a reduction in prevalence of **0%**. This target is estimated with a 95% uncertainty interval from **0%** to **23%**.

For CET based on Pichon-Riviere, the national target for anaemia in **Sierra Leone** should be a reduction in prevalence of **0%**. This target is estimated with a 95% uncertainty interval from **0%** to **0%**.

For a near-infinite CET, the national target for anaemia in **Sierra Leone** should be a reduction in prevalence of **20%**. This target is estimated with a 95% uncertainty interval from **11%** to **31%**.

Input parameters

- Among women of reproductive age in **Sierra Leone**, the prevalence of overall anaemia is **43.8%**
  - **Mild anaemia:** 21.9%
  - **Moderate anaemia:** 20.3%
  - **Severe anaemia:** 1.6%
- The prevalence of malaria is **33.4%**.

Intervention coverage and costs

Iron supplementation (antenatal care)

- **Current coverage:** 96.0%
- **Maximum feasible coverage:** 98.2%
- **Estimated unit cost:** USD $2.43 (1.03 - 4.75)

Iron supplementation (all women of reproductive age)

- **Current coverage:** 39.6%
- **Maximum feasible coverage:** 98.2%
- **Estimated unit cost:** USD $2.32 (1.63 - 9.13)

Antenatal preventative IPTp-SP

- **Current coverage:** 73.0%
- **Maximum feasible coverage:** 98.2%
- **Estimated unit cost:** USD $1.19 (0.62 - 2.58)

Staple food supplementation

- **Current coverage:** 0.9%
- **Maximum feasible coverage:** 51.7%
- **Estimated unit cost:** USD $0.71 (0.10 - 2.17)

Cost-Effectiveness Analysis

The estimated cost per Year of Life Disabled (YLD) averted for each intervention in **Sierra Leone** is given below, with interquartile ranges (IQR):

Iron supplementation (antenatal care)

- **Median Cost per YLD:** >USD 100k
- **IQR:** USD $323 – >USD 100k

Iron supplementation (all women of reproductive age)

- **Median Cost per YLD:** USD $1378
- **IQR:** USD $911 – $1972

Antenatal preventative IPTp-SP

- **Median Cost per YLD:** USD $822
- **IQR:** USD $608 – $1144

Staple food supplementation

- **Median Cost per YLD:** USD $1003
- **IQR:** USD $639 – $1537

Economic Considerations

Cost-effectiveness thresholds (CET, in USD per DALY averted) in **Sierra Leone** is as follows:

- **Estimates from Pichon-Riviere et al. (Lower Bound):** USD $43
- **Estimates from Pichon-Riviere et al. (Upper Bound):** USD $134
- **CET equal to per capita GDP:** USD $433
- **CET equal to 2.3 (LMICs) or 1.7 (HICs) times per capita GDP:** USD $997

⬆ Back to Table of Contents

Anaemia Reduction in Singapore

National Target

For our baseline CET = 1 x GDP per capita, the national target for anaemia in **Singapore** should be a reduction in prevalence of **25%**. This target is estimated with a 95% uncertainty interval from **9%** to **45%**.

For CET based on Pichon-Riviere, the national target for anaemia in **Singapore** should be a reduction in prevalence of **21%**. This target is estimated with a 95% uncertainty interval from **8%** to **40%**.

For a near-infinite CET, the national target for anaemia in **Singapore** should be a reduction in prevalence of **29%**. This target is estimated with a 95% uncertainty interval from **16%** to **46%**.

Input parameters

- Among women of reproductive age in **Singapore**, the prevalence of overall anaemia is **18.4%**

    - **Mild anaemia:** 17.0%
    - **Moderate anaemia:** 1.4%
    - **Severe anaemia:** 0.0%
- There is no data on malaria prevalence; assume 0%.

Intervention coverage and costs

Iron supplementation (antenatal care)

- **Current coverage:** 81.0%
- **Maximum feasible coverage:** 100.0%
- **Estimated unit cost:** USD $98.02 (38.00 - 153.88)

Iron supplementation (all women of reproductive age)

- **Current coverage:** 33.4%
- **Maximum feasible coverage:** 100.0%
- **Estimated unit cost:** USD $60.52 (22.19 - 89.65)

Antenatal preventative IPTp-SP

- **Current coverage:** 0.0%
- **Maximum feasible coverage:** 100.0%
- **Estimated unit cost:** USD $44.29 (22.21 - 68.05)

Staple food supplementation

- **Current coverage:** 12.5%
- **Maximum feasible coverage:** 100.0%
- **Estimated unit cost:** USD $0.48 (0.10 - 1.35)

Cost-Effectiveness Analysis

The estimated cost per Year of Life Disabled (YLD) averted for each intervention in **Singapore** is given below, with interquartile ranges (IQR):

Iron supplementation (antenatal care)

- **Median Cost per YLD:** USD $67733
- **IQR:** USD $41009 – >USD 100k

Iron supplementation (all women of reproductive age)

- **Median Cost per YLD:** USD $98720
- **IQR:** USD $61184 – >USD 100k

Antenatal preventative IPTp-SP

- **Median Cost per YLD:** >USD 100k
- **IQR:** >USD 100k

Staple food supplementation

- **Median Cost per YLD:** USD $3390
- **IQR:** USD $1982 – $5850

Economic Considerations

Cost-effectiveness thresholds (CET, in USD per DALY averted) in **Singapore** is as follows:

- **Estimates from Pichon-Riviere et al. (Lower Bound):** USD $25420
- **Estimates from Pichon-Riviere et al. (Upper Bound):** USD $30504
- **CET equal to per capita GDP:** USD $84734
- **CET equal to 2.3 (LMICs) or 1.7 (HICs) times per capita GDP:** USD $118628

⬆ Back to Table of Contents

Anaemia Reduction in Slovakia

National Target

For our baseline CET = 1 x GDP per capita, the national target for anaemia in **Slovakia** should be a reduction in prevalence of **20%**. This target is estimated with a 95% uncertainty interval from **7%** to **38%**.

For CET based on Pichon-Riviere, the national target for anaemia in **Slovakia** should be a reduction in prevalence of **16%**. This target is estimated with a 95% uncertainty interval from

6% to **34%**.

For a near-infinite CET, the national target for anaemia in **Slovakia** should be a reduction in prevalence of **24%**. This target is estimated with a 95% uncertainty interval from **13%** to **40%**.

Input parameters

- Among women of reproductive age in **Slovakia**, the prevalence of overall anaemia is **21.3%**
  - **Mild anaemia:** 14.3%
  - **Moderate anaemia:** 6.6%
  - **Severe anaemia:** 0.4%
- There is no data on malaria prevalence; assume 0%.

Intervention coverage and costs

Iron supplementation (antenatal care)

- **Current coverage:** NaN%
- **Maximum feasible coverage:** NaN%
- **Estimated unit cost:** USD $41.02 (9.86 - 63.35)

Iron supplementation (all women of reproductive age)

- **Current coverage:** NaN%
- **Maximum feasible coverage:** NaN%
- **Estimated unit cost:** USD $25.70 (6.71 - 51.96)

Antenatal preventative IPTp-SP

- **Current coverage:** NaN%
- **Maximum feasible coverage:** NaN%
- **Estimated unit cost:** USD $1.58 (0.86 - 3.01)

Staple food supplementation

- **Current coverage:** NaN%
- **Maximum feasible coverage:** NaN%
- **Estimated unit cost:** USD $0.07 (0.01 - 0.12)

Cost-Effectiveness Analysis

The estimated cost per Year of Life Disabled (YLD) averted for each intervention in **Slovakia** is given below, with interquartile ranges (IQR):

Iron supplementation (antenatal care)

- **Median Cost per YLD:** USD $14077
- **IQR:** USD $9280 – $22193

Iron supplementation (all women of reproductive age)

- **Median Cost per YLD:** USD $26423
- **IQR:** USD $16997 – $39783

Antenatal preventative IPTp-SP

- **Median Cost per YLD:** >USD 100k
- **IQR:** >USD 100k

Staple food supplementation

- **Median Cost per YLD:** USD $195
- **IQR:** USD $124 – $303

Economic Considerations

Cost-effectiveness thresholds (CET, in USD per DALY averted) in **Slovakia** is as follows:

- **Estimates from Pichon-Riviere et al. (Lower Bound):** USD $11990
- **Estimates from Pichon-Riviere et al. (Upper Bound):** USD $13948
- **CET equal to per capita GDP:** USD $24470
- **CET equal to 2.3 (LMICs) or 1.7 (HICs) times per capita GDP:** USD $34258

⬆ Back to Table of Contents

Anaemia Reduction in Slovenia

National Target

For our baseline CET = 1 x GDP per capita, the national target for anaemia in **Slovenia** should be a reduction in prevalence of **24%**. This target is estimated with a 95% uncertainty interval from **9%** to **42%**.

For CET based on Pichon-Riviere, the national target for anaemia in **Slovenia** should be a reduction in prevalence of **21%**. This target is estimated with a 95% uncertainty interval from **8%** to **39%**.

For a near-infinite CET, the national target for anaemia in **Slovenia** should be a reduction in prevalence of **26%**. This target is estimated with a 95% uncertainty interval from **14%** to **42%**.

Input parameters

- Among women of reproductive age in **Slovenia**, the prevalence of overall anaemia is **17.6%**
  - **Mild anaemia:** 11.9%
  - **Moderate anaemia:** 5.4%
  - **Severe anaemia:** 0.3%
- There is no data on malaria prevalence; assume 0%.

Intervention coverage and costs

Iron supplementation (antenatal care)

- **Current coverage:** 81.0%
- **Maximum feasible coverage:** 100.0%
- **Estimated unit cost:** USD $41.14 (9.44 - 68.81)

Iron supplementation (all women of reproductive age)

- **Current coverage:** 33.4%
- **Maximum feasible coverage:** 100.0%
- **Estimated unit cost:** USD $23.45 (3.74 - 31.80)

Antenatal preventative IPTp-SP

- **Current coverage:** 0.0%

- **Maximum feasible coverage:** 100.0%
- **Estimated unit cost:** USD $0.85 (0.49 - 1.86)

Staple food supplementation

- **Current coverage:** 30.2%
- **Maximum feasible coverage:** 100.0%
- **Estimated unit cost:** USD $0.69 (0.52 - 1.02)

Cost-Effectiveness Analysis

The estimated cost per Year of Life Disabled (YLD) averted for each intervention in **Slovenia** is given below, with interquartile ranges (IQR):

Iron supplementation (antenatal care)

- **Median Cost per YLD:** USD $18091
- **IQR:** USD $12343 – $28636

Iron supplementation (all women of reproductive age)

- **Median Cost per YLD:** USD $23314
- **IQR:** USD $15824 – $33597

Antenatal preventative IPTp-SP

- **Median Cost per YLD:** >USD 100k
- **IQR:** >USD 100k

Staple food supplementation

- **Median Cost per YLD:** USD $3391
- **IQR:** USD $2426 – $4881

Economic Considerations

Cost-effectiveness thresholds (CET, in USD per DALY averted) in **Slovenia** is as follows:

- **Estimates from Pichon-Riviere et al. (Lower Bound):** USD $20585

- **Estimates from Pichon-Riviere et al. (Upper Bound):** USD $24123
- **CET equal to per capita GDP:** USD $32164
- **CET equal to 2.3 (LMICs) or 1.7 (HICs) times per capita GDP:** USD $45029

⬆ Back to Table of Contents

Anaemia Reduction in Solomon Islands

National Target

For our baseline CET = 1 x GDP per capita, the national target for anaemia in **Solomon Islands** should be a reduction in prevalence of **23%**. This target is estimated with a 95% uncertainty interval from **1%** to **40%**.

For CET based on Pichon-Riviere, the national target for anaemia in **Solomon Islands** should be a reduction in prevalence of **12%**. This target is estimated with a 95% uncertainty interval from **0%** to **36%**.

For a near-infinite CET, the national target for anaemia in **Solomon Islands** should be a reduction in prevalence of **24%**. This target is estimated with a 95% uncertainty interval from **13%** to **41%**.

Input parameters

- Among women of reproductive age in **Solomon Islands**, the prevalence of overall anaemia is **44.8%**
  - **Mild anaemia:** 26.1%
  - **Moderate anaemia:** 17.5%
  - **Severe anaemia:** 1.2%
- The prevalence of malaria is **2.3%**.

Intervention coverage and costs

Iron supplementation (antenatal care)

- **Current coverage:** 84.2%
- **Maximum feasible coverage:** 89.2%
- **Estimated unit cost:** USD $4.69 (2.27 - 8.24)

Iron supplementation (all women of reproductive age)

- **Current coverage:** 34.7%
- **Maximum feasible coverage:** 84.2%
- **Estimated unit cost:** USD $3.82 (1.96 - 8.33)

Antenatal preventative IPTp-SP

- **Current coverage:** 35.0%
- **Maximum feasible coverage:** 79.2%
- **Estimated unit cost:** USD $3.00 (1.57 - 5.26)

Staple food supplementation

- **Current coverage:** 1.0%
- **Maximum feasible coverage:** 74.8%
- **Estimated unit cost:** USD $0.57 (0.08 - 1.75)

Cost-Effectiveness Analysis

The estimated cost per Year of Life Disabled (YLD) averted for each intervention in **Solomon Islands** is given below, with interquartile ranges (IQR):

Iron supplementation (antenatal care)

- **Median Cost per YLD:** USD $1116
- **IQR:** USD $614 – >USD 100k

Iron supplementation (all women of reproductive age)

- **Median Cost per YLD:** USD $1665
- **IQR:** USD $1146 – $2401

Antenatal preventative IPTp-SP

- **Median Cost per YLD:** USD $15119
- **IQR:** USD $10966 – $20016

Staple food supplementation

- **Median Cost per YLD:** USD $898
- **IQR:** USD $574 – $1440

Economic Considerations

Cost-effectiveness thresholds (CET, in USD per DALY averted) in **Solomon Islands** is as follows:

- **Estimates from Pichon-Riviere et al. (Lower Bound):** USD $463
- **Estimates from Pichon-Riviere et al. (Upper Bound):** USD $925
- **CET equal to per capita GDP:** USD $2203
- **CET equal to 2.3 (LMICs) or 1.7 (HICs) times per capita GDP:** USD $5067

⬆ Back to Table of Contents

Anaemia Reduction in Somalia

National Target

For our baseline CET = 1 x GDP per capita, the national target for anaemia in **Somalia** should be a reduction in prevalence of **1%**. This target is estimated with a 95% uncertainty interval from **0%** to **26%**.

For CET based on Pichon-Riviere, the national target for anaemia in **Somalia** should be a reduction in prevalence of **0%**. This target is estimated with a 95% uncertainty interval from **0%** to **0%**.

For a near-infinite CET, the national target for anaemia in **Somalia** should be a reduction in prevalence of **14%**. This target is estimated with a 95% uncertainty interval from **6%** to **28%**.

Input parameters

- Among women of reproductive age in **Somalia**, the prevalence of overall anaemia is **55.7%**
  - **Mild anaemia:** 38.5%
  - **Moderate anaemia:** 16.8%
  - **Severe anaemia:** 0.5%
- The prevalence of malaria is **6.8%**.

Intervention coverage and costs

Iron supplementation (antenatal care)

- **Current coverage:** 36.3%
- **Maximum feasible coverage:** 41.3%
- **Estimated unit cost:** USD $2.67 (1.22 - 5.28)

Iron supplementation (all women of reproductive age)

- **Current coverage:** 15.0%
- **Maximum feasible coverage:** 36.3%
- **Estimated unit cost:** USD $3.17 (2.10 - 10.06)

Antenatal preventative IPTp-SP

- **Current coverage:** 0.0%
- **Maximum feasible coverage:** 24.0%
- **Estimated unit cost:** USD $1.46 (0.78 - 3.02)

Staple food supplementation

- **Current coverage:** 56.0%
- **Maximum feasible coverage:** 100.0%
- **Estimated unit cost:** USD $0.72 (0.11 - 2.24)

Cost-Effectiveness Analysis

The estimated cost per Year of Life Disabled (YLD) averted for each intervention in **Somalia** is given below, with interquartile ranges (IQR):

Iron supplementation (antenatal care)

- **Median Cost per YLD:** USD $652
- **IQR:** USD $415 – >USD 100k

Iron supplementation (all women of reproductive age)

- **Median Cost per YLD:** USD $1927
- **IQR:** USD $1308 – $2866

Antenatal preventative IPTp-SP

- **Median Cost per YLD:** USD $3977
- **IQR:** USD $2972 – $5525

Staple food supplementation

- **Median Cost per YLD:** USD $1081
- **IQR:** USD $672 – $1794

Economic Considerations

Cost-effectiveness thresholds (CET, in USD per DALY averted) in **Somalia** is as follows:

- **Estimates from Pichon-Riviere et al. (Lower Bound):** USD $135
- **Estimates from Pichon-Riviere et al. (Upper Bound):** USD $155
- **CET equal to per capita GDP:** USD $644
- **CET equal to 2.3 (LMICs) or 1.7 (HICs) times per capita GDP:** USD $1481

⬆ Back to Table of Contents

Anaemia Reduction in South Africa

National Target

For our baseline CET = 1 x GDP per capita, the national target for anaemia in **South Africa** should be a reduction in prevalence of **17%**. This target is estimated with a 95% uncertainty interval from **7%** to **32%**.

For CET based on Pichon-Riviere, the national target for anaemia in **South Africa** should be a reduction in prevalence of **16%**. This target is estimated with a 95% uncertainty interval from **5%** to **31%**.

For a near-infinite CET, the national target for anaemia in **South Africa** should be a reduction in prevalence of **17%**. This target is estimated with a 95% uncertainty interval from **8%** to **31%**.

Input parameters

- Among women of reproductive age in **South Africa**, the prevalence of overall anaemia is **34.8%**
  - **Mild anaemia:** 18.3%
  - **Moderate anaemia:** 14.7%
  - **Severe anaemia:** 1.8%
- The prevalence of malaria is **0.0%**.

Intervention coverage and costs

Iron supplementation (antenatal care)

- **Current coverage:** 89.6%
- **Maximum feasible coverage:** 93.1%
- **Estimated unit cost:** USD $7.33 (3.50 - 11.96)

Iron supplementation (all women of reproductive age)

- **Current coverage:** 36.9%
- **Maximum feasible coverage:** 93.1%
- **Estimated unit cost:** USD $5.02 (2.48 - 9.79)

Antenatal preventative IPTp-SP

- **Current coverage:** 27.8%
- **Maximum feasible coverage:** 93.1%
- **Estimated unit cost:** USD $4.99 (2.52 - 8.11)

Staple food supplementation

- **Current coverage:** 40.1%
- **Maximum feasible coverage:** 76.0%
- **Estimated unit cost:** USD $1.31 (1.24 - 2.44)

Cost-Effectiveness Analysis

The estimated cost per Year of Life Disabled (YLD) averted for each intervention in **South Africa** is given below, with interquartile ranges (IQR):

Iron supplementation (antenatal care)

- **Median Cost per YLD:** USD $1859
- **IQR:** USD $1077 – >USD 100k

Iron supplementation (all women of reproductive age)

- **Median Cost per YLD:** USD $2444
- **IQR:** USD $1742 – $3364

Antenatal preventative IPTp-SP

- **Median Cost per YLD:** >USD 100k
- **IQR:** >USD 100k

Staple food supplementation

- **Median Cost per YLD:** USD $1976
- **IQR:** USD $1440 – $2819

Economic Considerations

Cost-effectiveness thresholds (CET, in USD per DALY averted) in **South Africa** is as follows:

- **Estimates from Pichon-Riviere et al. (Lower Bound):** USD $3127
- **Estimates from Pichon-Riviere et al. (Upper Bound):** USD $4252
- **CET equal to per capita GDP:** USD $6253
- **CET equal to 2.3 (LMICs) or 1.7 (HICs) times per capita GDP:** USD $14382

⬆ Back to Table of Contents

Anaemia Reduction in South Korea

National Target

For our baseline CET = 1 x GDP per capita, the national target for anaemia in **South Korea** should be a reduction in prevalence of **22%**. This target is estimated with a 95% uncertainty interval from **0%** to **41%**.

For CET based on Pichon-Riviere, the national target for anaemia in **South Korea** should be a reduction in prevalence of **21%**. This target is estimated with a 95% uncertainty interval from **0%** to **41%**.

For a near-infinite CET, the national target for anaemia in **South Korea** should be a reduction in prevalence of **29%**. This target is estimated with a 95% uncertainty interval from **16%** to **47%**.

Input parameters

- Among women of reproductive age in **South Korea**, the prevalence of overall anaemia is **9.2%**
  - **Mild anaemia:** 7.3%
  - **Moderate anaemia:** 1.9%
  - **Severe anaemia:** 0.1%
- The prevalence of malaria is **0.0%**.

Intervention coverage and costs

Iron supplementation (antenatal care)

- **Current coverage:** NaN%
- **Maximum feasible coverage:** NaN%
- **Estimated unit cost:** USD $71.74 (24.73 - 115.83)

Iron supplementation (all women of reproductive age)

- **Current coverage:** NaN%
- **Maximum feasible coverage:** NaN%
- **Estimated unit cost:** USD $41.90 (12.96 - 60.41)

Antenatal preventative IPTp-SP

- **Current coverage:** NaN%
- **Maximum feasible coverage:** NaN%

- **Estimated unit cost:** USD $24.38 (12.26 - 38.18)

Staple food supplementation

- **Current coverage:** NaN%
- **Maximum feasible coverage:** NaN%
- **Estimated unit cost:** USD $0.60 (0.17 - 1.61)

Cost-Effectiveness Analysis

The estimated cost per Year of Life Disabled (YLD) averted for each intervention in **South Korea** is given below, with interquartile ranges (IQR):

Iron supplementation (antenatal care)

- **Median Cost per YLD:** USD $97159
- **IQR:** USD $62612 – >USD 100k

Iron supplementation (all women of reproductive age)

- **Median Cost per YLD:** >USD 100k
- **IQR:** USD $89760 – >USD 100k

Antenatal preventative IPTp-SP

- **Median Cost per YLD:** >USD 100k
- **IQR:** >USD 100k

Staple food supplementation

- **Median Cost per YLD:** USD $9542
- **IQR:** USD $5805 – $15649

Economic Considerations

Cost-effectiveness thresholds (CET, in USD per DALY averted) in **South Korea** is as follows:

- **Estimates from Pichon-Riviere et al. (Lower Bound):** USD $16561
- **Estimates from Pichon-Riviere et al. (Upper Bound):** USD $24179

- **CET equal to per capita GDP:** USD $33121
- **CET equal to 2.3 (LMICs) or 1.7 (HICs) times per capita GDP:** USD $46370

⬆ Back to Table of Contents

Anaemia Reduction in South Sudan

National Target

For our baseline CET = 1 x GDP per capita, the national target for anaemia in **South Sudan** should be a reduction in prevalence of **23%**. This target is estimated with a 95% uncertainty interval from **0%** to **43%**.

For CET based on Pichon-Riviere, the national target for anaemia in **South Sudan** should be a reduction in prevalence of **0%**. This target is estimated with a 95% uncertainty interval from **0%** to **28%**.

For a near-infinite CET, the national target for anaemia in **South Sudan** should be a reduction in prevalence of **28%**. This target is estimated with a 95% uncertainty interval from **14%** to **47%**.

Input parameters

- Among women of reproductive age in **South Sudan**, the prevalence of overall anaemia is **43.0%**
  - **Mild anaemia:** 28.0%
  - **Moderate anaemia:** 14.4%
  - **Severe anaemia:** 0.6%
- The prevalence of malaria is **27.6%**.

Intervention coverage and costs

Iron supplementation (antenatal care)

- **Current coverage:** 77.9%
- **Maximum feasible coverage:** 82.9%
- **Estimated unit cost:** USD $2.31 (0.99 - 4.56)

Iron supplementation (all women of reproductive age)

- **Current coverage:** 32.1%
- **Maximum feasible coverage:** 77.9%
- **Estimated unit cost:** USD $2.43 (1.22 - 5.57)

Antenatal preventative IPTp-SP

- **Current coverage:** 53.1%
- **Maximum feasible coverage:** 58.1%
- **Estimated unit cost:** USD $1.24 (0.65 - 2.64)

Staple food supplementation

- **Current coverage:** 3.6%
- **Maximum feasible coverage:** 100.0%
- **Estimated unit cost:** USD $0.34 (0.10 - 1.21)

Cost-Effectiveness Analysis

The estimated cost per Year of Life Disabled (YLD) averted for each intervention in **South Sudan** is given below, with interquartile ranges (IQR):

Iron supplementation (antenatal care)

- **Median Cost per YLD:** USD $636
- **IQR:** USD $356 – >USD 100k

Iron supplementation (all women of reproductive age)

- **Median Cost per YLD:** USD $1335
- **IQR:** USD $883 – $1930

Antenatal preventative IPTp-SP

- **Median Cost per YLD:** USD $1705
- **IQR:** USD $1074 – >USD 100k

Staple food supplementation

- **Median Cost per YLD:** USD $763

- **IQR:** USD $478 – $1218

Economic Considerations

Cost-effectiveness thresholds (CET, in USD per DALY averted) in **South Sudan** is as follows:

- **Estimates from Pichon-Riviere et al. (Lower Bound):** USD $536
- **Estimates from Pichon-Riviere et al. (Upper Bound):** USD $257
- **CET equal to per capita GDP:** USD $1072
- **CET equal to 2.3 (LMICs) or 1.7 (HICs) times per capita GDP:** USD $2465

⬆ Back to Table of Contents

Anaemia Reduction in Spain

National Target

For our baseline CET = 1 x GDP per capita, the national target for anaemia in **Spain** should be a reduction in prevalence of **16%**. This target is estimated with a 95% uncertainty interval from **6%** to **34%**.

For CET based on Pichon-Riviere, the national target for anaemia in **Spain** should be a reduction in prevalence of **16%**. This target is estimated with a 95% uncertainty interval from **6%** to **33%**.

For a near-infinite CET, the national target for anaemia in **Spain** should be a reduction in prevalence of **24%**. This target is estimated with a 95% uncertainty interval from **13%** to **39%**.

Input parameters

- Among women of reproductive age in **Spain**, the prevalence of overall anaemia is **8.3%**
  - **Mild anaemia:** 6.8%
  - **Moderate anaemia:** 1.5%
  - **Severe anaemia:** 0.0%
- There is no data on malaria prevalence; assume 0%.

Intervention coverage and costs

Iron supplementation (antenatal care)

- **Current coverage:** 81.0%
- **Maximum feasible coverage:** 100.0%
- **Estimated unit cost:** USD $59.51 (18.86 - 95.94)

Iron supplementation (all women of reproductive age)

- **Current coverage:** 33.4%
- **Maximum feasible coverage:** 100.0%
- **Estimated unit cost:** USD $36.58 (12.69 - 69.90)

Antenatal preventative IPTp-SP

- **Current coverage:** 0.0%
- **Maximum feasible coverage:** 100.0%
- **Estimated unit cost:** USD $16.27 (8.20 - 27.23)

Staple food supplementation

- **Current coverage:** 36.8%
- **Maximum feasible coverage:** 97.6%
- **Estimated unit cost:** USD $0.16 (0.06 - 0.32)

Cost-Effectiveness Analysis

The estimated cost per Year of Life Disabled (YLD) averted for each intervention in **Spain** is given below, with interquartile ranges (IQR):

Iron supplementation (antenatal care)

- **Median Cost per YLD:** >USD 100k
- **IQR:** USD $66620 – >USD 100k

Iron supplementation (all women of reproductive age)

- **Median Cost per YLD:** >USD 100k
- **IQR:** >USD 100k

Antenatal preventative IPTp-SP

- **Median Cost per YLD:** >USD 100k
- **IQR:** >USD 100k

Staple food supplementation

- **Median Cost per YLD:** USD $2810
- **IQR:** USD $1864 – $4395

Economic Considerations

Cost-effectiveness thresholds (CET, in USD per DALY averted) in **Spain** is as follows:

- **Estimates from Pichon-Riviere et al. (Lower Bound):** USD $23201
- **Estimates from Pichon-Riviere et al. (Upper Bound):** USD $27449
- **CET equal to per capita GDP:** USD $32677
- **CET equal to 2.3 (LMICs) or 1.7 (HICs) times per capita GDP:** USD $45748

⬆ Back to Table of Contents

Anaemia Reduction in Sri Lanka

National Target

For our baseline CET = 1 x GDP per capita, the national target for anaemia in **Sri Lanka** should be a reduction in prevalence of **17%**. This target is estimated with a 95% uncertainty interval from **6%** to **29%**.

For CET based on Pichon-Riviere, the national target for anaemia in **Sri Lanka** should be a reduction in prevalence of **10%**. This target is estimated with a 95% uncertainty interval from **0%** to **24%**.

For a near-infinite CET, the national target for anaemia in **Sri Lanka** should be a reduction in prevalence of **18%**. This target is estimated with a 95% uncertainty interval from **9%** to **29%**.

Input parameters

- Among women of reproductive age in **Sri Lanka**, the prevalence of overall anaemia is **36.3%**
  - **Mild anaemia:** 21.9%
  - **Moderate anaemia:** 13.6%

    - **Severe anaemia:** 0.9%
- The prevalence of malaria is **0.0%**.

Intervention coverage and costs

Iron supplementation (antenatal care)

- **Current coverage:** 86.9%
- **Maximum feasible coverage:** 88.9%
- **Estimated unit cost:** USD $5.58 (2.72 - 9.69)

Iron supplementation (all women of reproductive age)

- **Current coverage:** 35.8%
- **Maximum feasible coverage:** 88.9%
- **Estimated unit cost:** USD $3.90 (2.42 - 11.94)

Antenatal preventative IPTp-SP

- **Current coverage:** 0.0%
- **Maximum feasible coverage:** 88.9%
- **Estimated unit cost:** USD $3.62 (1.87 - 6.13)

Staple food supplementation

- **Current coverage:** 2.2%
- **Maximum feasible coverage:** 49.0%
- **Estimated unit cost:** USD $0.59 (0.09 - 1.82)

Cost-Effectiveness Analysis

The estimated cost per Year of Life Disabled (YLD) averted for each intervention in **Sri Lanka** is given below, with interquartile ranges (IQR):

Iron supplementation (antenatal care)

- **Median Cost per YLD:** USD $1644
- **IQR:** USD $882 – >USD 100k

Iron supplementation (all women of reproductive age)

- **Median Cost per YLD:** USD $2690
- **IQR:** USD $1861 – $3992

Antenatal preventative IPTp-SP

- **Median Cost per YLD:** >USD 100k
- **IQR:** >USD 100k

Staple food supplementation

- **Median Cost per YLD:** USD $1172
- **IQR:** USD $727 – $1838

Economic Considerations

Cost-effectiveness thresholds (CET, in USD per DALY averted) in **Sri Lanka** is as follows:

- **Estimates from Pichon-Riviere et al. (Lower Bound):** USD $689
- **Estimates from Pichon-Riviere et al. (Upper Bound):** USD $1340
- **CET equal to per capita GDP:** USD $3828
- **CET equal to 2.3 (LMICs) or 1.7 (HICs) times per capita GDP:** USD $8804

⬆ Back to Table of Contents

Anaemia Reduction in St. Kitts & Nevis

National Target

For our baseline CET = 1 x GDP per capita, the national target for anaemia in **St. Kitts & Nevis** should be a reduction in prevalence of **24%**. This target is estimated with a 95% uncertainty interval from **10%** to **42%**.

For CET based on Pichon-Riviere, the national target for anaemia in **St. Kitts & Nevis** should be a reduction in prevalence of **22%**. This target is estimated with a 95% uncertainty interval from **8%** to **40%**.

For a near-infinite CET, the national target for anaemia in **St. Kitts & Nevis** should be a reduction in prevalence of **26%**. This target is estimated with a 95% uncertainty interval from **14%** to

42%.

Input parameters

- Among women of reproductive age in **St. Kitts & Nevis**, the prevalence of overall anaemia is **36.1%**
  - **Mild anaemia:** 22.8%
  - **Moderate anaemia:** 12.6%
  - **Severe anaemia:** 0.7%
- There is no data on malaria prevalence; assume 0%.

Intervention coverage and costs

Iron supplementation (antenatal care)

- **Current coverage:** NaN%
- **Maximum feasible coverage:** NaN%
- **Estimated unit cost:** USD $NaN (NaN - NaN)

Iron supplementation (all women of reproductive age)

- **Current coverage:** NaN%
- **Maximum feasible coverage:** NaN%
- **Estimated unit cost:** USD $NaN (NaN - NaN)

Antenatal preventative IPTp-SP

- **Current coverage:** NaN%
- **Maximum feasible coverage:** NaN%
- **Estimated unit cost:** USD $NaN (NaN - NaN)

Staple food supplementation

- **Current coverage:** NaN%
- **Maximum feasible coverage:** NaN%
- **Estimated unit cost:** USD $NaN (NaN - NaN)

Cost-Effectiveness Analysis

The estimated cost per Year of Life Disabled (YLD) averted for each intervention in **St. Kitts & Nevis** is given below, with interquartile ranges (IQR):

Iron supplementation (antenatal care)

- **Median Cost per YLD:** USD $10897
- **IQR:** USD $7373 – $17028

Iron supplementation (all women of reproductive age)

- **Median Cost per YLD:** USD $15599
- **IQR:** USD $10993 – $22793

Antenatal preventative IPTp-SP

- **Median Cost per YLD:** >USD 100k
- **IQR:** >USD 100k

Staple food supplementation

- **Median Cost per YLD:** USD $866
- **IQR:** USD $539 – $1339

Economic Considerations

Cost-effectiveness thresholds (CET, in USD per DALY averted) in **St. Kitts & Nevis** is as follows:

- **Estimates from Pichon-Riviere et al. (Lower Bound):** USD $NaN
- **Estimates from Pichon-Riviere et al. (Upper Bound):** USD $NaN
- **CET equal to per capita GDP:** USD $NaN
- **CET equal to 2.3 (LMICs) or 1.7 (HICs) times per capita GDP:** USD $NaN

⬆ Back to Table of Contents

Anaemia Reduction in St. Lucia

National Target

For our baseline CET = 1 x GDP per capita, the national target for anaemia in **St. Lucia** should be a reduction in prevalence of **18%**. This target is estimated with a 95% uncertainty interval from **7%** to **31%**.

For CET based on Pichon-Riviere, the national target for anaemia in **St. Lucia** should be a reduction in prevalence of **16%**. This target is estimated with a 95% uncertainty interval from **5%** to **31%**.

For a near-infinite CET, the national target for anaemia in **St. Lucia** should be a reduction in prevalence of **18%**. This target is estimated with a 95% uncertainty interval from **8%** to **32%**.

Input parameters

- Among women of reproductive age in **St. Lucia**, the prevalence of overall anaemia is **37.6%**
  - **Mild anaemia:** 22.4%
  - **Moderate anaemia:** 14.3%
  - **Severe anaemia:** 1.0%
- There is no data on malaria prevalence; assume 0%.

Intervention coverage and costs

Iron supplementation (antenatal care)

- **Current coverage:** 88.3%
- **Maximum feasible coverage:** 93.3%
- **Estimated unit cost:** USD $16.02 (7.92 - 25.28)

Iron supplementation (all women of reproductive age)

- **Current coverage:** 36.4%
- **Maximum feasible coverage:** 88.3%
- **Estimated unit cost:** USD $8.38 (4.19 - 15.15)

Antenatal preventative IPTp-SP

- **Current coverage:** 0.1%
- **Maximum feasible coverage:** 86.5%
- **Estimated unit cost:** USD $11.70 (5.91 - 18.39)

Staple food supplementation

- **Current coverage:** 57.2%
- **Maximum feasible coverage:** 100.0%
- **Estimated unit cost:** USD $0.37 (0.19 - 0.72)

Cost-Effectiveness Analysis

The estimated cost per Year of Life Disabled (YLD) averted for each intervention in **St. Lucia** is given below, with interquartile ranges (IQR):

Iron supplementation (antenatal care)

- **Median Cost per YLD:** USD $4064
- **IQR:** USD $2333 – >USD 100k

Iron supplementation (all women of reproductive age)

- **Median Cost per YLD:** USD $3987
- **IQR:** USD $2813 – $5620

Antenatal preventative IPTp-SP

- **Median Cost per YLD:** >USD 100k
- **IQR:** >USD 100k

Staple food supplementation

- **Median Cost per YLD:** USD $506
- **IQR:** USD $335 – $770

Economic Considerations

Cost-effectiveness thresholds (CET, in USD per DALY averted) in **St. Lucia** is as follows:

- **Estimates from Pichon-Riviere et al. (Lower Bound):** USD $4474
- **Estimates from Pichon-Riviere et al. (Upper Bound):** USD $6151
- **CET equal to per capita GDP:** USD $13980

- **CET equal to 2.3 (LMICs) or 1.7 (HICs) times per capita GDP:** USD $32154

⬆ Back to Table of Contents

Anaemia Reduction in St. Vincent & Grenadines

National Target

For our baseline CET = 1 x GDP per capita, the national target for anaemia in **St. Vincent & Grenadines** should be a reduction in prevalence of **22%**. This target is estimated with a 95% uncertainty interval from **10%** to **38%**.

For CET based on Pichon-Riviere, the national target for anaemia in **St. Vincent & Grenadines** should be a reduction in prevalence of **19%**. This target is estimated with a 95% uncertainty interval from **8%** to **36%**.

For a near-infinite CET, the national target for anaemia in **St. Vincent & Grenadines** should be a reduction in prevalence of **22%**. This target is estimated with a 95% uncertainty interval from **11%** to **36%**.

Input parameters

- Among women of reproductive age in **St. Vincent & Grenadines**, the prevalence of overall anaemia is **39.0%**
  - **Mild anaemia:** 23.1%
  - **Moderate anaemia:** 15.0%
  - **Severe anaemia:** 1.0%
- There is no data on malaria prevalence; assume 0%.

Intervention coverage and costs

Iron supplementation (antenatal care)

- **Current coverage:** NaN%
- **Maximum feasible coverage:** NaN%
- **Estimated unit cost:** USD $NaN (NaN - NaN)

Iron supplementation (all women of reproductive age)

- **Current coverage:** NaN%
- **Maximum feasible coverage:** NaN%
- **Estimated unit cost:** USD $NaN (NaN - NaN)

Antenatal preventative IPTp-SP

- **Current coverage:** NaN%
- **Maximum feasible coverage:** NaN%
- **Estimated unit cost:** USD $NaN (NaN - NaN)

Staple food supplementation

- **Current coverage:** NaN%
- **Maximum feasible coverage:** NaN%
- **Estimated unit cost:** USD $NaN (NaN - NaN)

Cost-Effectiveness Analysis

The estimated cost per Year of Life Disabled (YLD) averted for each intervention in **St. Vincent & Grenadines** is given below, with interquartile ranges (IQR):

Iron supplementation (antenatal care)

- **Median Cost per YLD:** USD $3049
- **IQR:** USD $1745 – >USD 100k

Iron supplementation (all women of reproductive age)

- **Median Cost per YLD:** USD $3637
- **IQR:** USD $2561 – $5384

Antenatal preventative IPTp-SP

- **Median Cost per YLD:** >USD 100k
- **IQR:** >USD 100k

Staple food supplementation

- **Median Cost per YLD:** USD $888

- **IQR:** USD $619 – $1329

Economic Considerations

Cost-effectiveness thresholds (CET, in USD per DALY averted) in **St. Vincent & Grenadines** is as follows:

- **Estimates from Pichon-Riviere et al. (Lower Bound):** USD $NaN
- **Estimates from Pichon-Riviere et al. (Upper Bound):** USD $NaN
- **CET equal to per capita GDP:** USD $NaN
- **CET equal to 2.3 (LMICs) or 1.7 (HICs) times per capita GDP:** USD $NaN

⬆ Back to Table of Contents

Anaemia Reduction in Sudan

National Target

For our baseline CET = 1 x GDP per capita, the national target for anaemia in **Sudan** should be a reduction in prevalence of **24%**. This target is estimated with a 95% uncertainty interval from **9%** to **41%**.

For CET based on Pichon-Riviere, the national target for anaemia in **Sudan** should be a reduction in prevalence of **18%**. This target is estimated with a 95% uncertainty interval from **7%** to **36%**.

For a near-infinite CET, the national target for anaemia in **Sudan** should be a reduction in prevalence of **27%**. This target is estimated with a 95% uncertainty interval from **16%** to **43%**.

Input parameters

- Among women of reproductive age in **Sudan**, the prevalence of overall anaemia is **44.5%**
  - **Mild anaemia:** 26.4%
  - **Moderate anaemia:** 17.0%
  - **Severe anaemia:** 1.1%
- The prevalence of malaria is **4.2%**.

Intervention coverage and costs

Iron supplementation (antenatal care)

- **Current coverage:** 36.3%
- **Maximum feasible coverage:** 71.7%
- **Estimated unit cost:** USD $4.21 (1.98 - 7.49)

Iron supplementation (all women of reproductive age)

- **Current coverage:** 15.0%
- **Maximum feasible coverage:** 71.7%
- **Estimated unit cost:** USD $4.12 (2.05 - 8.60)

Antenatal preventative IPTp-SP

- **Current coverage:** 0.0%
- **Maximum feasible coverage:** 71.7%
- **Estimated unit cost:** USD $2.72 (1.41 - 4.93)

Staple food supplementation

- **Current coverage:** 10.2%
- **Maximum feasible coverage:** 80.0%
- **Estimated unit cost:** USD $0.04 (0.01 - 0.14)

Cost-Effectiveness Analysis

The estimated cost per Year of Life Disabled (YLD) averted for each intervention in **Sudan** is given below, with interquartile ranges (IQR):

Iron supplementation (antenatal care)

- **Median Cost per YLD:** USD $781
- **IQR:** USD $568 – $1053

Iron supplementation (all women of reproductive age)

- **Median Cost per YLD:** USD $1910
- **IQR:** USD $1309 – $2747

Antenatal preventative IPTp-SP

- **Median Cost per YLD:** USD $11469
- **IQR:** USD $8750 – $15636

Staple food supplementation

- **Median Cost per YLD:** USD $71
- **IQR:** USD $46 – $114

Economic Considerations

Cost-effectiveness thresholds (CET, in USD per DALY averted) in **Sudan** is as follows:

- **Estimates from Pichon-Riviere et al. (Lower Bound):** USD $205
- **Estimates from Pichon-Riviere et al. (Upper Bound):** USD $636
- **CET equal to per capita GDP:** USD $2272
- **CET equal to 2.3 (LMICs) or 1.7 (HICs) times per capita GDP:** USD $5227

⬆ Back to Table of Contents

Anaemia Reduction in Suriname

National Target

For our baseline CET = 1 x GDP per capita, the national target for anaemia in **Suriname** should be a reduction in prevalence of **27%**. This target is estimated with a 95% uncertainty interval from **12%** to **45%**.

For CET based on Pichon-Riviere, the national target for anaemia in **Suriname** should be a reduction in prevalence of **25%**. This target is estimated with a 95% uncertainty interval from **11%** to **43%**.

For a near-infinite CET, the national target for anaemia in **Suriname** should be a reduction in prevalence of **26%**. This target is estimated with a 95% uncertainty interval from **13%** to **43%**.

Input parameters

- Among women of reproductive age in **Suriname**, the prevalence of overall anaemia is **40.8%**

- **Mild anaemia:** 24.0%
- **Moderate anaemia:** 15.7%
- **Severe anaemia:** 1.1%

- The prevalence of malaria is **0.0%**.

Intervention coverage and costs

Iron supplementation (antenatal care)

- **Current coverage:** 88.3%
- **Maximum feasible coverage:** 93.3%
- **Estimated unit cost:** USD $6.52 (3.16 - 11.37)

Iron supplementation (all women of reproductive age)

- **Current coverage:** 36.4%
- **Maximum feasible coverage:** 88.3%
- **Estimated unit cost:** USD $4.78 (2.41 - 10.47)

Antenatal preventative IPTp-SP

- **Current coverage:** 0.1%
- **Maximum feasible coverage:** 68.0%
- **Estimated unit cost:** USD $4.43 (2.28 - 7.82)

Staple food supplementation

- **Current coverage:** 17.2%
- **Maximum feasible coverage:** 100.0%
- **Estimated unit cost:** USD $0.50 (0.07 - 1.50)

Cost-Effectiveness Analysis

The estimated cost per Year of Life Disabled (YLD) averted for each intervention in **Suriname** is given below, with interquartile ranges (IQR):

Iron supplementation (antenatal care)

- **Median Cost per YLD:** USD $1482
- **IQR:** USD $909 – >USD 100k

Iron supplementation (all women of reproductive age)

- **Median Cost per YLD:** USD $2291
- **IQR:** USD $1597 – $3301

Antenatal preventative IPTp-SP

- **Median Cost per YLD:** >USD 100k
- **IQR:** >USD 100k

Staple food supplementation

- **Median Cost per YLD:** USD $892
- **IQR:** USD $534 – $1357

Economic Considerations

Cost-effectiveness thresholds (CET, in USD per DALY averted) in **Suriname** is as follows:

- **Estimates from Pichon-Riviere et al. (Lower Bound):** USD $3702
- **Estimates from Pichon-Riviere et al. (Upper Bound):** USD $5098
- **CET equal to per capita GDP:** USD $6069
- **CET equal to 2.3 (LMICs) or 1.7 (HICs) times per capita GDP:** USD $13959

⬆ Back to Table of Contents

Anaemia Reduction in Sweden

National Target

For our baseline CET = 1 x GDP per capita, the national target for anaemia in **Sweden** should be a reduction in prevalence of **16%**. This target is estimated with a 95% uncertainty interval from **6%** to **34%**.

For CET based on Pichon-Riviere, the national target for anaemia in **Sweden** should be a reduction in prevalence of **15%**. This target is estimated with a 95% uncertainty interval from

6% to **32%**.

For a near-infinite CET, the national target for anaemia in **Sweden** should be a reduction in prevalence of **24%**. This target is estimated with a 95% uncertainty interval from **13%** to **40%**.

Input parameters

- Among women of reproductive age in **Sweden**, the prevalence of overall anaemia is **7.6%**
  - **Mild anaemia:** 6.3%
  - **Moderate anaemia:** 1.3%
  - **Severe anaemia:** 0.0%
- There is no data on malaria prevalence; assume 0%.

Intervention coverage and costs

Iron supplementation (antenatal care)

- **Current coverage:** 81.0%
- **Maximum feasible coverage:** 100.0%
- **Estimated unit cost:** USD $97.69 (37.95 - 153.61)

Iron supplementation (all women of reproductive age)

- **Current coverage:** 33.4%
- **Maximum feasible coverage:** 100.0%
- **Estimated unit cost:** USD $60.44 (24.63 - 106.05)

Antenatal preventative IPTp-SP

- **Current coverage:** 0.0%
- **Maximum feasible coverage:** 100.0%
- **Estimated unit cost:** USD $45.09 (22.61 - 70.82)

Staple food supplementation

- **Current coverage:** 36.8%
- **Maximum feasible coverage:** 100.0%
- **Estimated unit cost:** USD $0.16 (0.06 - 0.32)

Cost-Effectiveness Analysis

The estimated cost per Year of Life Disabled (YLD) averted for each intervention in **Sweden** is given below, with interquartile ranges (IQR):

Iron supplementation (antenatal care)

- **Median Cost per YLD:** >USD 100k
- **IQR:** >USD 100k

Iron supplementation (all women of reproductive age)

- **Median Cost per YLD:** >USD 100k
- **IQR:** >USD 100k

Antenatal preventative IPTp-SP

- **Median Cost per YLD:** >USD 100k
- **IQR:** >USD 100k

Staple food supplementation

- **Median Cost per YLD:** USD $2926
- **IQR:** USD $1878 – $4654

Economic Considerations

Cost-effectiveness thresholds (CET, in USD per DALY averted) in **Sweden** is as follows:

- **Estimates from Pichon-Riviere et al. (Lower Bound):** USD $47296
- **Estimates from Pichon-Riviere et al. (Upper Bound):** USD $55179
- **CET equal to per capita GDP:** USD $56305
- **CET equal to 2.3 (LMICs) or 1.7 (HICs) times per capita GDP:** USD $78827

⬆ Back to Table of Contents

Anaemia Reduction in Switzerland

National Target

For our baseline CET = 1 x GDP per capita, the national target for anaemia in **Switzerland** should be a reduction in prevalence of **16%**. This target is estimated with a 95% uncertainty interval from **6%** to **34%**.

For CET based on Pichon-Riviere, the national target for anaemia in **Switzerland** should be a reduction in prevalence of **16%**. This target is estimated with a 95% uncertainty interval from **6%** to **33%**.

For a near-infinite CET, the national target for anaemia in **Switzerland** should be a reduction in prevalence of **25%**. This target is estimated with a 95% uncertainty interval from **13%** to **41%**.

Input parameters

- Among women of reproductive age in **Switzerland**, the prevalence of overall anaemia is **12.9%**
  - **Mild anaemia:** 12.0%
  - **Moderate anaemia:** 0.9%
  - **Severe anaemia:** 0.0%
- There is no data on malaria prevalence; assume 0%.

Intervention coverage and costs

Iron supplementation (antenatal care)

- **Current coverage:** 81.0%
- **Maximum feasible coverage:** 100.0%
- **Estimated unit cost:** USD $141.35 (59.74 - 220.40)

Iron supplementation (all women of reproductive age)

- **Current coverage:** 33.4%
- **Maximum feasible coverage:** 100.0%
- **Estimated unit cost:** USD $88.33 (38.66 - 148.69)

Antenatal preventative IPTp-SP

- **Current coverage:** 0.0%

- **Maximum feasible coverage:** 100.0%
- **Estimated unit cost:** USD $78.62 (39.38 - 122.00)

Staple food supplementation

- **Current coverage:** 37.9%
- **Maximum feasible coverage:** 100.0%
- **Estimated unit cost:** USD $0.11 (0.04 - 0.20)

Cost-Effectiveness Analysis

The estimated cost per Year of Life Disabled (YLD) averted for each intervention in **Switzerland** is given below, with interquartile ranges (IQR):

Iron supplementation (antenatal care)

- **Median Cost per YLD:** >USD 100k
- **IQR:** USD $81623 – >USD 100k

Iron supplementation (all women of reproductive age)

- **Median Cost per YLD:** >USD 100k
- **IQR:** >USD 100k

Antenatal preventative IPTp-SP

- **Median Cost per YLD:** >USD 100k
- **IQR:** >USD 100k

Staple food supplementation

- **Median Cost per YLD:** USD $933
- **IQR:** USD $569 – $1616

Economic Considerations

Cost-effectiveness thresholds (CET, in USD per DALY averted) in **Switzerland** is as follows:

- **Estimates from Pichon-Riviere et al. (Lower Bound):** USD $87996

- **Estimates from Pichon-Riviere et al. (Upper Bound):** USD $102995
- **CET equal to per capita GDP:** USD $99995
- **CET equal to 2.3 (LMICs) or 1.7 (HICs) times per capita GDP:** USD $139993

⬆ Back to Table of Contents

Anaemia Reduction in Syria

National Target

For our baseline CET = 1 x GDP per capita, the national target for anaemia in **Syria** should be a reduction in prevalence of **9%**. This target is estimated with a 95% uncertainty interval from **2%** to **23%**.

For CET based on Pichon-Riviere, the national target for anaemia in **Syria** should be a reduction in prevalence of **8%**. This target is estimated with a 95% uncertainty interval from **0%** to **23%**.

For a near-infinite CET, the national target for anaemia in **Syria** should be a reduction in prevalence of **14%**. This target is estimated with a 95% uncertainty interval from **6%** to **27%**.

Input parameters

- Among women of reproductive age in **Syria**, the prevalence of overall anaemia is **35.3%**
  - **Mild anaemia:** 22.0%
  - **Moderate anaemia:** 12.6%
  - **Severe anaemia:** 0.7%
- The prevalence of malaria is **0.0%**.

Intervention coverage and costs

Iron supplementation (antenatal care)

- **Current coverage:** NaN%
- **Maximum feasible coverage:** NaN%
- **Estimated unit cost:** USD $2.94 (1.34 - 5.57)

Iron supplementation (all women of reproductive age)

- **Current coverage:** NaN%
- **Maximum feasible coverage:** NaN%
- **Estimated unit cost:** USD $2.66 (1.33 - 6.31)

Antenatal preventative IPTp-SP

- **Current coverage:** NaN%
- **Maximum feasible coverage:** NaN%
- **Estimated unit cost:** USD $1.77 (0.94 - 3.49)

Staple food supplementation

- **Current coverage:** NaN%
- **Maximum feasible coverage:** NaN%
- **Estimated unit cost:** USD $0.04 (0.01 - 0.12)

Cost-Effectiveness Analysis

The estimated cost per Year of Life Disabled (YLD) averted for each intervention in **Syria** is given below, with interquartile ranges (IQR):

Iron supplementation (antenatal care)

- **Median Cost per YLD:** USD $811
- **IQR:** USD $570 – $1149

Iron supplementation (all women of reproductive age)

- **Median Cost per YLD:** USD $1858
- **IQR:** USD $1290 – $2722

Antenatal preventative IPTp-SP

- **Median Cost per YLD:** >USD 100k
- **IQR:** >USD 100k

Staple food supplementation

- **Median Cost per YLD:** USD $71

- **IQR:** USD $44 – $111

Economic Considerations

Cost-effectiveness thresholds (CET, in USD per DALY averted) in **Syria** is as follows:

- **Estimates from Pichon-Riviere et al. (Lower Bound):** USD $370
- **Estimates from Pichon-Riviere et al. (Upper Bound):** USD $101
- **CET equal to per capita GDP:** USD $421
- **CET equal to 2.3 (LMICs) or 1.7 (HICs) times per capita GDP:** USD $967

⬆ Back to Table of Contents

Anaemia Reduction in São Tomé & Príncipe

National Target

For our baseline CET = 1 x GDP per capita, the national target for anaemia in **São Tomé & Príncipe** should be a reduction in prevalence of **27%**. This target is estimated with a 95% uncertainty interval from **11%** to **46%**.

For CET based on Pichon-Riviere, the national target for anaemia in **São Tomé & Príncipe** should be a reduction in prevalence of **21%**. This target is estimated with a 95% uncertainty interval from **0%** to **42%**.

For a near-infinite CET, the national target for anaemia in **São Tomé & Príncipe** should be a reduction in prevalence of **28%**. This target is estimated with a 95% uncertainty interval from **15%** to **46%**.

Input parameters

- Among women of reproductive age in **São Tomé & Príncipe**, the prevalence of overall anaemia is **NaN%**
    - **Mild anaemia:** NaN%
    - **Moderate anaemia:** NaN%
    - **Severe anaemia:** NaN%
- There is no data on malaria prevalence; assume 0%.

Intervention coverage and costs

Iron supplementation (antenatal care)

- **Current coverage:** NaN%
- **Maximum feasible coverage:** NaN%
- **Estimated unit cost:** USD $NaN (NaN - NaN)

Iron supplementation (all women of reproductive age)

- **Current coverage:** NaN%
- **Maximum feasible coverage:** NaN%
- **Estimated unit cost:** USD $NaN (NaN - NaN)

Antenatal preventative IPTp-SP

- **Current coverage:** NaN%
- **Maximum feasible coverage:** NaN%
- **Estimated unit cost:** USD $NaN (NaN - NaN)

Staple food supplementation

- **Current coverage:** NaN%
- **Maximum feasible coverage:** NaN%
- **Estimated unit cost:** USD $NaN (NaN - NaN)

Cost-Effectiveness Analysis

The estimated cost per Year of Life Disabled (YLD) averted for each intervention in **São Tomé & Príncipe** is given below, with interquartile ranges (IQR):

Iron supplementation (antenatal care)

- **Median Cost per YLD:** USD $959
- **IQR:** USD $573 – >USD 100k

Iron supplementation (all women of reproductive age)

- **Median Cost per YLD:** USD $1567
- **IQR:** USD $1082 – $2180

Antenatal preventative IPTp-SP

- **Median Cost per YLD:** USD $41102
- **IQR:** USD $30850 – $53598

Staple food supplementation

- **Median Cost per YLD:** USD $647
- **IQR:** USD $404 – $1007

Economic Considerations

Cost-effectiveness thresholds (CET, in USD per DALY averted) in **São Tomé & Príncipe** is as follows:

- **Estimates from Pichon-Riviere et al. (Lower Bound):** USD $NaN
- **Estimates from Pichon-Riviere et al. (Upper Bound):** USD $NaN
- **CET equal to per capita GDP:** USD $NaN
- **CET equal to 2.3 (LMICs) or 1.7 (HICs) times per capita GDP:** USD $NaN

⬆ Back to Table of Contents

Anaemia Reduction in Tajikistan

National Target

For our baseline CET = 1 x GDP per capita, the national target for anaemia in **Tajikistan** should be a reduction in prevalence of **25%**. This target is estimated with a 95% uncertainty interval from **11%** to **46%**.

For CET based on Pichon-Riviere, the national target for anaemia in **Tajikistan** should be a reduction in prevalence of **23%**. This target is estimated with a 95% uncertainty interval from **0%** to **42%**.

For a near-infinite CET, the national target for anaemia in **Tajikistan** should be a reduction in prevalence of **34%**. This target is estimated with a 95% uncertainty interval from **20%** to **52%**.

Input parameters

- Among women of reproductive age in **Tajikistan**, the prevalence of overall anaemia is **41.2%**
  - **Mild anaemia:** 21.9%
  - **Moderate anaemia:** 17.9%
  - **Severe anaemia:** 1.3%
- The prevalence of malaria is **0.0%**.

Intervention coverage and costs

Iron supplementation (antenatal care)

- **Current coverage:** 47.1%
- **Maximum feasible coverage:** 93.8%
- **Estimated unit cost:** USD $3.66 (1.77 - 7.91)

Iron supplementation (all women of reproductive age)

- **Current coverage:** 19.4%
- **Maximum feasible coverage:** 93.8%
- **Estimated unit cost:** USD $3.45 (1.77 - 9.19)

Antenatal preventative IPTp-SP

- **Current coverage:** 0.0%
- **Maximum feasible coverage:** 93.8%
- **Estimated unit cost:** USD $2.53 (1.34 - 5.74)

Staple food supplementation

- **Current coverage:** 1.9%
- **Maximum feasible coverage:** 100.0%
- **Estimated unit cost:** USD $0.08 (0.01 - 0.27)

Cost-Effectiveness Analysis

The estimated cost per Year of Life Disabled (YLD) averted for each intervention in **Tajikistan** is given below, with interquartile ranges (IQR):

Iron supplementation (antenatal care)

- **Median Cost per YLD:** USD $734
- **IQR:** USD $522 – $998

Iron supplementation (all women of reproductive age)

- **Median Cost per YLD:** USD $1891
- **IQR:** USD $1312 – $2757

Antenatal preventative IPTp-SP

- **Median Cost per YLD:** >USD 100k
- **IQR:** >USD 100k

Staple food supplementation

- **Median Cost per YLD:** USD $142
- **IQR:** USD $91 – $221

Economic Considerations

Cost-effectiveness thresholds (CET, in USD per DALY averted) in **Tajikistan** is as follows:

- **Estimates from Pichon-Riviere et al. (Lower Bound):** USD $131
- **Estimates from Pichon-Riviere et al. (Upper Bound):** USD $416
- **CET equal to per capita GDP:** USD $1189
- **CET equal to 2.3 (LMICs) or 1.7 (HICs) times per capita GDP:** USD $2735

⬆ Back to Table of Contents

Anaemia Reduction in Tanzania

National Target

For our baseline CET = 1 x GDP per capita, the national target for anaemia in **Tanzania** should be a reduction in prevalence of **8%**. This target is estimated with a 95% uncertainty interval from **0%** to **22%**.

For CET based on Pichon-Riviere, the national target for anaemia in **Tanzania** should be a reduction in prevalence of **0%**. This target is estimated with a 95% uncertainty interval from **0%** to **10%**.

For a near-infinite CET, the national target for anaemia in **Tanzania** should be a reduction in prevalence of **14%**. This target is estimated with a 95% uncertainty interval from **8%** to **24%**.

Input parameters

- Among women of reproductive age in **Tanzania**, the prevalence of overall anaemia is **50.0%**
  - **Mild anaemia:** 28.9%
  - **Moderate anaemia:** 20.0%
  - **Severe anaemia:** 1.1%
- The prevalence of malaria is **7.9%**.

Intervention coverage and costs

Iron supplementation (antenatal care)

- **Current coverage:** 80.9%
- **Maximum feasible coverage:** 89.7%
- **Estimated unit cost:** USD $2.77 (1.20 - 5.03)

Iron supplementation (all women of reproductive age)

- **Current coverage:** 33.4%
- **Maximum feasible coverage:** 89.7%
- **Estimated unit cost:** USD $2.70 (1.83 - 9.06)

Antenatal preventative IPTp-SP

- **Current coverage:** 55.2%
- **Maximum feasible coverage:** 89.7%
- **Estimated unit cost:** USD $1.46 (0.76 - 2.79)

Staple food supplementation

- **Current coverage:** 1.9%
- **Maximum feasible coverage:** 24.8%
- **Estimated unit cost:** USD $0.35 (0.11 - 1.44)

Cost-Effectiveness Analysis

The estimated cost per Year of Life Disabled (YLD) averted for each intervention in **Tanzania** is given below, with interquartile ranges (IQR):

Iron supplementation (antenatal care)

- **Median Cost per YLD:** USD $460
- **IQR:** USD $297 – >USD 100k

Iron supplementation (all women of reproductive age)

- **Median Cost per YLD:** USD $1429
- **IQR:** USD $981 – $2100

Antenatal preventative IPTp-SP

- **Median Cost per YLD:** USD $2669
- **IQR:** USD $2031 – $3554

Staple food supplementation

- **Median Cost per YLD:** USD $653
- **IQR:** USD $417 – $1033

Economic Considerations

Cost-effectiveness thresholds (CET, in USD per DALY averted) in **Tanzania** is as follows:

- **Estimates from Pichon-Riviere et al. (Lower Bound):** USD $157
- **Estimates from Pichon-Riviere et al. (Upper Bound):** USD $315
- **CET equal to per capita GDP:** USD $1211

- **CET equal to 2.3 (LMICs) or 1.7 (HICs) times per capita GDP:** USD $2785

⬆ Back to Table of Contents

Anaemia Reduction in Thailand

National Target

For our baseline CET = 1 x GDP per capita, the national target for anaemia in **Thailand** should be a reduction in prevalence of **29%**. This target is estimated with a 95% uncertainty interval from **10%** to **49%**.

For CET based on Pichon-Riviere, the national target for anaemia in **Thailand** should be a reduction in prevalence of **23%**. This target is estimated with a 95% uncertainty interval from **0%** to **44%**.

For a near-infinite CET, the national target for anaemia in **Thailand** should be a reduction in prevalence of **29%**. This target is estimated with a 95% uncertainty interval from **15%** to **47%**.

Input parameters

- Among women of reproductive age in **Thailand**, the prevalence of overall anaemia is **23.2%**
    - **Mild anaemia:** 12.2%
    - **Moderate anaemia:** 9.9%
    - **Severe anaemia:** 1.1%
- The prevalence of malaria is **0.0%**.

Intervention coverage and costs

Iron supplementation (antenatal care)

- **Current coverage:** 90.8%
- **Maximum feasible coverage:** 95.8%
- **Estimated unit cost:** USD $6.76 (3.24 - 11.00)

Iron supplementation (all women of reproductive age)

- **Current coverage:** 37.4%
- **Maximum feasible coverage:** 90.8%

- **Estimated unit cost:** USD $4.01 (2.01 - 8.58)

Antenatal preventative IPTp-SP

- **Current coverage:** 0.0%
- **Maximum feasible coverage:** 80.8%
- **Estimated unit cost:** USD $4.51 (2.32 - 7.41)

Staple food supplementation

- **Current coverage:** 2.0%
- **Maximum feasible coverage:** 100.0%
- **Estimated unit cost:** USD $0.77 (0.20 - 2.15)

Cost-Effectiveness Analysis

The estimated cost per Year of Life Disabled (YLD) averted for each intervention in **Thailand** is given below, with interquartile ranges (IQR):

Iron supplementation (antenatal care)

- **Median Cost per YLD:** USD $2484
- **IQR:** USD $1386 – >USD 100k

Iron supplementation (all women of reproductive age)

- **Median Cost per YLD:** USD $2881
- **IQR:** USD $2038 – $4180

Antenatal preventative IPTp-SP

- **Median Cost per YLD:** >USD 100k
- **IQR:** >USD 100k

Staple food supplementation

- **Median Cost per YLD:** USD $2049
- **IQR:** USD $1326 – $3167

Economic Considerations

Cost-effectiveness thresholds (CET, in USD per DALY averted) in **Thailand** is as follows:

- **Estimates from Pichon-Riviere et al. (Lower Bound):** USD $1936
- **Estimates from Pichon-Riviere et al. (Upper Bound):** USD $2654
- **CET equal to per capita GDP:** USD $7172
- **CET equal to 2.3 (LMICs) or 1.7 (HICs) times per capita GDP:** USD $16495

⬆ Back to Table of Contents

Anaemia Reduction in Timor-Leste

National Target

For our baseline CET = 1 x GDP per capita, the national target for anaemia in **Timor-Leste** should be a reduction in prevalence of **2%**. This target is estimated with a 95% uncertainty interval from **0%** to **39%**.

For CET based on Pichon-Riviere, the national target for anaemia in **Timor-Leste** should be a reduction in prevalence of **0%**. This target is estimated with a 95% uncertainty interval from **0%** to **27%**.

For a near-infinite CET, the national target for anaemia in **Timor-Leste** should be a reduction in prevalence of **26%**. This target is estimated with a 95% uncertainty interval from **13%** to **43%**.

Input parameters

- Among women of reproductive age in **Timor-Leste**, the prevalence of overall anaemia is **32.1%**
  - **Mild anaemia:** 22.9%
  - **Moderate anaemia:** 8.9%
  - **Severe anaemia:** 0.2%
- The prevalence of malaria is **0.0%**.

Intervention coverage and costs

Iron supplementation (antenatal care)

- **Current coverage:** 85.7%
- **Maximum feasible coverage:** 90.7%
- **Estimated unit cost:** USD $3.29 (1.52 - 5.99)

Iron supplementation (all women of reproductive age)

- **Current coverage:** 35.3%
- **Maximum feasible coverage:** 85.7%
- **Estimated unit cost:** USD $2.83 (1.44 - 6.77)

Antenatal preventative IPTp-SP

- **Current coverage:** 0.0%
- **Maximum feasible coverage:** 84.6%
- **Estimated unit cost:** USD $1.92 (1.02 - 3.62)

Staple food supplementation

- **Current coverage:** 1.6%
- **Maximum feasible coverage:** 80.7%
- **Estimated unit cost:** USD $0.56 (0.11 - 1.79)

Cost-Effectiveness Analysis

The estimated cost per Year of Life Disabled (YLD) averted for each intervention in **Timor-Leste** is given below, with interquartile ranges (IQR):

Iron supplementation (antenatal care)

- **Median Cost per YLD:** USD $1444
- **IQR:** USD $822 – >USD 100k

Iron supplementation (all women of reproductive age)

- **Median Cost per YLD:** USD $2670
- **IQR:** USD $1840 – $4043

Antenatal preventative IPTp-SP

- **Median Cost per YLD:** >USD 100k
- **IQR:** >USD 100k

Staple food supplementation

- **Median Cost per YLD:** USD $1917
- **IQR:** USD $1194 – $2993

Economic Considerations

Cost-effectiveness thresholds (CET, in USD per DALY averted) in **Timor-Leste** is as follows:

- **Estimates from Pichon-Riviere et al. (Lower Bound):** USD $379
- **Estimates from Pichon-Riviere et al. (Upper Bound):** USD $725
- **CET equal to per capita GDP:** USD $1649
- **CET equal to 2.3 (LMICs) or 1.7 (HICs) times per capita GDP:** USD $3792

⬆ Back to Table of Contents

Anaemia Reduction in Togo

National Target

For our baseline CET = 1 x GDP per capita, the national target for anaemia in **Togo** should be a reduction in prevalence of **11%**. This target is estimated with a 95% uncertainty interval from **0%** to **24%**.

For CET based on Pichon-Riviere, the national target for anaemia in **Togo** should be a reduction in prevalence of **0%**. This target is estimated with a 95% uncertainty interval from **0%** to **13%**.

For a near-infinite CET, the national target for anaemia in **Togo** should be a reduction in prevalence of **15%**. This target is estimated with a 95% uncertainty interval from **8%** to **25%**.

Input parameters

- Among women of reproductive age in **Togo**, the prevalence of overall anaemia is **67.8%**
  - **Mild anaemia:** 40.4%
  - **Moderate anaemia:** 26.6%
  - **Severe anaemia:** 0.8%

- The prevalence of malaria is **19.6%**.

Intervention coverage and costs

Iron supplementation (antenatal care)

- **Current coverage:** 86.1%
- **Maximum feasible coverage:** 91.1%
- **Estimated unit cost:** USD $2.49 (1.12 - 4.92)

Iron supplementation (all women of reproductive age)

- **Current coverage:** 35.5%
- **Maximum feasible coverage:** 86.1%
- **Estimated unit cost:** USD $2.68 (1.75 - 9.25)

Antenatal preventative IPTp-SP

- **Current coverage:** 67.6%
- **Maximum feasible coverage:** 72.6%
- **Estimated unit cost:** USD $1.23 (0.64 - 2.42)

Staple food supplementation

- **Current coverage:** 2.0%
- **Maximum feasible coverage:** 33.2%
- **Estimated unit cost:** USD $0.39 (0.11 - 1.38)

Cost-Effectiveness Analysis

The estimated cost per Year of Life Disabled (YLD) averted for each intervention in **Togo** is given below, with interquartile ranges (IQR):

Iron supplementation (antenatal care)

- **Median Cost per YLD:** USD $384
- **IQR:** USD $230 – >USD 100k

Iron supplementation (all women of reproductive age)

- **Median Cost per YLD:** USD $1136
- **IQR:** USD $783 – $1677

Antenatal preventative IPTp-SP

- **Median Cost per YLD:** USD $1264
- **IQR:** USD $791 – >USD 100k

Staple food supplementation

- **Median Cost per YLD:** USD $526
- **IQR:** USD $321 – $820

Economic Considerations

Cost-effectiveness thresholds (CET, in USD per DALY averted) in **Togo** is as follows:

- **Estimates from Pichon-Riviere et al. (Lower Bound):** USD $71
- **Estimates from Pichon-Riviere et al. (Upper Bound):** USD $243
- **CET equal to per capita GDP:** USD $1013
- **CET equal to 2.3 (LMICs) or 1.7 (HICs) times per capita GDP:** USD $2330

⬆ Back to Table of Contents

Anaemia Reduction in Tonga

National Target

For our baseline CET = 1 x GDP per capita, the national target for anaemia in **Tonga** should be a reduction in prevalence of **25%**. This target is estimated with a 95% uncertainty interval from **10%** to **43%**.

For CET based on Pichon-Riviere, the national target for anaemia in **Tonga** should be a reduction in prevalence of **21%**. This target is estimated with a 95% uncertainty interval from **8%** to **41%**.

For a near-infinite CET, the national target for anaemia in **Tonga** should be a reduction in prevalence of **27%**. This target is estimated with a 95% uncertainty interval from **14%** to **44%**.

Input parameters

- Among women of reproductive age in **Tonga**, the prevalence of overall anaemia is **35.9%**
  - **Mild anaemia:** 21.9%
  - **Moderate anaemia:** 13.2%
  - **Severe anaemia:** 0.8%
- There is no data on malaria prevalence; assume 0%.

Intervention coverage and costs

Iron supplementation (antenatal care)

- **Current coverage:** 84.2%
- **Maximum feasible coverage:** 88.0%
- **Estimated unit cost:** USD $8.05 (3.95 - 14.13)

Iron supplementation (all women of reproductive age)

- **Current coverage:** 34.7%
- **Maximum feasible coverage:** 88.0%
- **Estimated unit cost:** USD $5.93 (3.00 - 12.63)

Antenatal preventative IPTp-SP

- **Current coverage:** 0.0%
- **Maximum feasible coverage:** 88.0%
- **Estimated unit cost:** USD $5.79 (2.97 - 10.29)

Staple food supplementation

- **Current coverage:** 15.5%
- **Maximum feasible coverage:** 100.0%
- **Estimated unit cost:** USD $0.07 (0.00 - 0.11)

Cost-Effectiveness Analysis

The estimated cost per Year of Life Disabled (YLD) averted for each intervention in **Tonga** is given below, with interquartile ranges (IQR):

Iron supplementation (antenatal care)

- **Median Cost per YLD:** USD $2517
- **IQR:** USD $1365 – >USD 100k

Iron supplementation (all women of reproductive age)

- **Median Cost per YLD:** USD $3383
- **IQR:** USD $2401 – $4944

Antenatal preventative IPTp-SP

- **Median Cost per YLD:** >USD 100k
- **IQR:** >USD 100k

Staple food supplementation

- **Median Cost per YLD:** USD $95
- **IQR:** USD $62 – $148

Economic Considerations

Cost-effectiveness thresholds (CET, in USD per DALY averted) in **Tonga** is as follows:

- **Estimates from Pichon-Riviere et al. (Lower Bound):** USD $1416
- **Estimates from Pichon-Riviere et al. (Upper Bound):** USD $1947
- **CET equal to per capita GDP:** USD $4426
- **CET equal to 2.3 (LMICs) or 1.7 (HICs) times per capita GDP:** USD $10180

⬆ Back to Table of Contents

Anaemia Reduction in Trinidad & Tobago

National Target

For our baseline CET = 1 x GDP per capita, the national target for anaemia in **Trinidad & Tobago** should be a reduction in prevalence of **17%**. This target is estimated with a 95% uncertainty interval from **5%** to **33%**.

For CET based on Pichon-Riviere, the national target for anaemia in **Trinidad & Tobago** should be a reduction in prevalence of **12%**. This target is estimated with a 95% uncertainty interval from **4%** to **29%**.

For a near-infinite CET, the national target for anaemia in **Trinidad & Tobago** should be a reduction in prevalence of **20%**. This target is estimated with a 95% uncertainty interval from **11%** to **35%**.

Input parameters

- Among women of reproductive age in **Trinidad & Tobago**, the prevalence of overall anaemia is **36.5%**
  - **Mild anaemia:** 23.0%
  - **Moderate anaemia:** 12.8%
  - **Severe anaemia:** 0.7%
- There is no data on malaria prevalence; assume 0%.

Intervention coverage and costs

Iron supplementation (antenatal care)

- **Current coverage:** NaN%
- **Maximum feasible coverage:** NaN%
- **Estimated unit cost:** USD $NaN (NaN - NaN)

Iron supplementation (all women of reproductive age)

- **Current coverage:** NaN%
- **Maximum feasible coverage:** NaN%
- **Estimated unit cost:** USD $NaN (NaN - NaN)

Antenatal preventative IPTp-SP

- **Current coverage:** NaN%

- **Maximum feasible coverage:** NaN%
- **Estimated unit cost:** USD $NaN (NaN - NaN)

Staple food supplementation

- **Current coverage:** NaN%
- **Maximum feasible coverage:** NaN%
- **Estimated unit cost:** USD $NaN (NaN - NaN)

Cost-Effectiveness Analysis

The estimated cost per Year of Life Disabled (YLD) averted for each intervention in **Trinidad & Tobago** is given below, with interquartile ranges (IQR):

Iron supplementation (antenatal care)

- **Median Cost per YLD:** USD $12405
- **IQR:** USD $8784 – $19868

Iron supplementation (all women of reproductive age)

- **Median Cost per YLD:** USD $17673
- **IQR:** USD $12538 – $24528

Antenatal preventative IPTp-SP

- **Median Cost per YLD:** >USD 100k
- **IQR:** >USD 100k

Staple food supplementation

- **Median Cost per YLD:** USD $854
- **IQR:** USD $606 – $1237

Economic Considerations

Cost-effectiveness thresholds (CET, in USD per DALY averted) in **Trinidad & Tobago** is as follows:

- **Estimates from Pichon-Riviere et al. (Lower Bound):** USD $NaN
- **Estimates from Pichon-Riviere et al. (Upper Bound):** USD $NaN
- **CET equal to per capita GDP:** USD $NaN
- **CET equal to 2.3 (LMICs) or 1.7 (HICs) times per capita GDP:** USD $NaN

⬆ Back to Table of Contents

Anaemia Reduction in Tunisia

National Target

For our baseline CET = 1 x GDP per capita, the national target for anaemia in **Tunisia** should be a reduction in prevalence of **11%**. This target is estimated with a 95% uncertainty interval from **1%** to **25%**.

For CET based on Pichon-Riviere, the national target for anaemia in **Tunisia** should be a reduction in prevalence of **5%**. This target is estimated with a 95% uncertainty interval from **0%** to **22%**.

For a near-infinite CET, the national target for anaemia in **Tunisia** should be a reduction in prevalence of **13%**. This target is estimated with a 95% uncertainty interval from **6%** to **25%**.

Input parameters

- Among women of reproductive age in **Tunisia**, the prevalence of overall anaemia is **26.8%**
  - **Mild anaemia:** 15.0%
  - **Moderate anaemia:** 10.8%
  - **Severe anaemia:** 1.0%
- There is no data on malaria prevalence; assume 0%.

Intervention coverage and costs

Iron supplementation (antenatal care)

- **Current coverage:** 58.4%
- **Maximum feasible coverage:** 61.4%
- **Estimated unit cost:** USD $5.80 (2.74 - 9.62)

Iron supplementation (all women of reproductive age)

- **Current coverage:** 24.1%
- **Maximum feasible coverage:** 61.4%
- **Estimated unit cost:** USD $3.90 (1.95 - 8.53)

Antenatal preventative IPTp-SP

- **Current coverage:** 0.0%
- **Maximum feasible coverage:** 61.4%
- **Estimated unit cost:** USD $3.79 (1.95 - 6.39)

Staple food supplementation

- **Current coverage:** 88.6%
- **Maximum feasible coverage:** 100.0%
- **Estimated unit cost:** USD $0.02 (0.00 - 0.06)

Cost-Effectiveness Analysis

The estimated cost per Year of Life Disabled (YLD) averted for each intervention in **Tunisia** is given below, with interquartile ranges (IQR):

Iron supplementation (antenatal care)

- **Median Cost per YLD:** USD $1412
- **IQR:** USD $1011 – $1968

Iron supplementation (all women of reproductive age)

- **Median Cost per YLD:** USD $2772
- **IQR:** USD $1927 – $3917

Antenatal preventative IPTp-SP

- **Median Cost per YLD:** >USD 100k
- **IQR:** >USD 100k

Staple food supplementation

- **Median Cost per YLD:** USD $62
- **IQR:** USD $32 – >USD 100k

Economic Considerations

Cost-effectiveness thresholds (CET, in USD per DALY averted) in **Tunisia** is as follows:

- **Estimates from Pichon-Riviere et al. (Lower Bound):** USD $1091
- **Estimates from Pichon-Riviere et al. (Upper Bound):** USD $2142
- **CET equal to per capita GDP:** USD $3895
- **CET equal to 2.3 (LMICs) or 1.7 (HICs) times per capita GDP:** USD $8959

⬆ Back to Table of Contents

Anaemia Reduction in Turkey

National Target

For our baseline CET = 1 x GDP per capita, the national target for anaemia in **Turkey** should be a reduction in prevalence of **17%**. This target is estimated with a 95% uncertainty interval from **6%** to **32%**.

For CET based on Pichon-Riviere, the national target for anaemia in **Turkey** should be a reduction in prevalence of **13%**. This target is estimated with a 95% uncertainty interval from **4%** to **29%**.

For a near-infinite CET, the national target for anaemia in **Turkey** should be a reduction in prevalence of **17%**. This target is estimated with a 95% uncertainty interval from **8%** to **30%**.

Input parameters

- Among women of reproductive age in **Turkey**, the prevalence of overall anaemia is **30.9%**
  - **Mild anaemia:** 20.3%
  - **Moderate anaemia:** 10.1%
  - **Severe anaemia:** 0.5%
- The prevalence of malaria is **0.0%**.

Intervention coverage and costs

Iron supplementation (antenatal care)

- **Current coverage:** 86.6%
- **Maximum feasible coverage:** 97.6%
- **Estimated unit cost:** USD $12.33 (6.09 - 22.77)

Iron supplementation (all women of reproductive age)

- **Current coverage:** 35.7%
- **Maximum feasible coverage:** 97.6%
- **Estimated unit cost:** USD $7.77 (3.92 - 17.59)

Antenatal preventative IPTp-SP

- **Current coverage:** 0.0%
- **Maximum feasible coverage:** 97.6%
- **Estimated unit cost:** USD $9.30 (4.73 - 17.81)

Staple food supplementation

- **Current coverage:** 60.9%
- **Maximum feasible coverage:** 100.0%
- **Estimated unit cost:** USD $0.36 (0.20 - 0.53)

Cost-Effectiveness Analysis

The estimated cost per Year of Life Disabled (YLD) averted for each intervention in **Turkey** is given below, with interquartile ranges (IQR):

Iron supplementation (antenatal care)

- **Median Cost per YLD:** USD $5116
- **IQR:** USD $2825 – >USD 100k

Iron supplementation (all women of reproductive age)

- **Median Cost per YLD:** USD $6051
- **IQR:** USD $4132 – $8882

Antenatal preventative IPTp-SP

- **Median Cost per YLD:** >USD 100k
- **IQR:** >USD 100k

Staple food supplementation

- **Median Cost per YLD:** USD $610
- **IQR:** USD $418 – $894

Economic Considerations

Cost-effectiveness thresholds (CET, in USD per DALY averted) in **Turkey** is as follows:

- **Estimates from Pichon-Riviere et al. (Lower Bound):** USD $4155
- **Estimates from Pichon-Riviere et al. (Upper Bound):** USD $5584
- **CET equal to per capita GDP:** USD $12986
- **CET equal to 2.3 (LMICs) or 1.7 (HICs) times per capita GDP:** USD $29867

⬆ Back to Table of Contents

Anaemia Reduction in Turkmenistan

National Target

For our baseline CET = 1 x GDP per capita, the national target for anaemia in **Turkmenistan** should be a reduction in prevalence of **16%**. This target is estimated with a 95% uncertainty interval from **8%** to **28%**.

For CET based on Pichon-Riviere, the national target for anaemia in **Turkmenistan** should be a reduction in prevalence of **16%**. This target is estimated with a 95% uncertainty interval from **8%** to **28%**.

For a near-infinite CET, the national target for anaemia in **Turkmenistan** should be a reduction in prevalence of **16%**. This target is estimated with a 95% uncertainty interval from **9%** to **28%**.

Input parameters

- Among women of reproductive age in **Turkmenistan**, the prevalence of overall anaemia is **33.9%**

- Mild anaemia: 17.8%
    - **Moderate anaemia:** 14.6%
    - **Severe anaemia:** 1.5%
- The prevalence of malaria is **0.0%**.

Intervention coverage and costs

Iron supplementation (antenatal care)

- **Current coverage:** 38.1%
- **Maximum feasible coverage:** 98.6%
- **Estimated unit cost:** USD $1.50 (0.69 - 3.34)

Iron supplementation (all women of reproductive age)

- **Current coverage:** 15.7%
- **Maximum feasible coverage:** 98.6%
- **Estimated unit cost:** USD $1.70 (0.89 - 5.29)

Antenatal preventative IPTp-SP

- **Current coverage:** 0.0%
- **Maximum feasible coverage:** 98.6%
- **Estimated unit cost:** USD $0.56 (0.36 - 1.47)

Staple food supplementation

- **Current coverage:** 100.0%
- **Maximum feasible coverage:** 100.0%
- **Estimated unit cost:** USD $0.11 (0.01 - 0.24)

Cost-Effectiveness Analysis

The estimated cost per Year of Life Disabled (YLD) averted for each intervention in **Turkmenistan** is given below, with interquartile ranges (IQR):

Iron supplementation (antenatal care)

- **Median Cost per YLD:** USD $351
- **IQR:** USD $254 – $485

Iron supplementation (all women of reproductive age)

- **Median Cost per YLD:** USD $1139
- **IQR:** USD $788 – $1708

Antenatal preventative IPTp-SP

- **Median Cost per YLD:** >USD 100k
- **IQR:** >USD 100k

Staple food supplementation

- **Median Cost per YLD:** >USD 100k
- **IQR:** USD $202 – >USD 100k

Economic Considerations

Cost-effectiveness thresholds (CET, in USD per DALY averted) in **Turkmenistan** is as follows:

- **Estimates from Pichon-Riviere et al. (Lower Bound):** USD $3676
- **Estimates from Pichon-Riviere et al. (Upper Bound):** USD $5055
- **CET equal to per capita GDP:** USD $9191
- **CET equal to 2.3 (LMICs) or 1.7 (HICs) times per capita GDP:** USD $21139

⬆ Back to Table of Contents

Anaemia Reduction in Tuvalu

National Target

For our baseline CET = 1 x GDP per capita, the national target for anaemia in **Tuvalu** should be a reduction in prevalence of **25%**. This target is estimated with a 95% uncertainty interval from **11%** to **45%**.

For CET based on Pichon-Riviere, the national target for anaemia in **Tuvalu** should be a reduction in prevalence of **23%**. This target is estimated with a 95% uncertainty interval from

7% to **42%**.

For a near-infinite CET, the national target for anaemia in **Tuvalu** should be a reduction in prevalence of **27%**. This target is estimated with a 95% uncertainty interval from **14%** to **44%**.

Input parameters

- Among women of reproductive age in **Tuvalu**, the prevalence of overall anaemia is **41.6%**
  - **Mild anaemia:** 25.5%
  - **Moderate anaemia:** 15.2%
  - **Severe anaemia:** 0.9%
- There is no data on malaria prevalence; assume 0%.

Intervention coverage and costs

Iron supplementation (antenatal care)

- **Current coverage:** 84.2%
- **Maximum feasible coverage:** 89.2%
- **Estimated unit cost:** USD $7.33 (3.60 - 13.12)

Iron supplementation (all women of reproductive age)

- **Current coverage:** 34.7%
- **Maximum feasible coverage:** 84.2%
- **Estimated unit cost:** USD $5.98 (3.52 - 16.02)

Antenatal preventative IPTp-SP

- **Current coverage:** 0.0%
- **Maximum feasible coverage:** 60.0%
- **Estimated unit cost:** USD $5.24 (2.70 - 9.47)

Staple food supplementation

- **Current coverage:** 15.5%
- **Maximum feasible coverage:** 100.0%
- **Estimated unit cost:** USD $0.47 (0.07 - 1.39)

Cost-Effectiveness Analysis

The estimated cost per Year of Life Disabled (YLD) averted for each intervention in **Tuvalu** is given below, with interquartile ranges (IQR):

Iron supplementation (antenatal care)

- **Median Cost per YLD:** USD $1926
- **IQR:** USD $1114 – >USD 100k

Iron supplementation (all women of reproductive age)

- **Median Cost per YLD:** USD $3564
- **IQR:** USD $2439 – $5099

Antenatal preventative IPTp-SP

- **Median Cost per YLD:** >USD 100k
- **IQR:** >USD 100k

Staple food supplementation

- **Median Cost per YLD:** USD $831
- **IQR:** USD $537 – $1377

Economic Considerations

Cost-effectiveness thresholds (CET, in USD per DALY averted) in **Tuvalu** is as follows:

- **Estimates from Pichon-Riviere et al. (Lower Bound):** USD $2186
- **Estimates from Pichon-Riviere et al. (Upper Bound):** USD $3170
- **CET equal to per capita GDP:** USD $5465
- **CET equal to 2.3 (LMICs) or 1.7 (HICs) times per capita GDP:** USD $12570

⬆ Back to Table of Contents

Anaemia Reduction in Uganda

National Target

For our baseline CET = 1 x GDP per capita, the national target for anaemia in **Uganda** should be a reduction in prevalence of **8%**. This target is estimated with a 95% uncertainty interval from **0%** to **23%**.

For CET based on Pichon-Riviere, the national target for anaemia in **Uganda** should be a reduction in prevalence of **0%**. This target is estimated with a 95% uncertainty interval from **0%** to **0%**.

For a near-infinite CET, the national target for anaemia in **Uganda** should be a reduction in prevalence of **18%**. This target is estimated with a 95% uncertainty interval from **10%** to **28%**.

Input parameters

- Among women of reproductive age in **Uganda**, the prevalence of overall anaemia is **31.0%**
  - **Mild anaemia:** 19.8%
  - **Moderate anaemia:** 10.7%
  - **Severe anaemia:** 0.5%
- The prevalence of malaria is **19.7%**.

Intervention coverage and costs

Iron supplementation (antenatal care)

- **Current coverage:** 89.1%
- **Maximum feasible coverage:** 95.8%
- **Estimated unit cost:** USD $2.64 (1.11 - 4.75)

Iron supplementation (all women of reproductive age)

- **Current coverage:** 36.7%
- **Maximum feasible coverage:** 95.8%
- **Estimated unit cost:** USD $2.47 (1.23 - 5.91)

Antenatal preventative IPTp-SP

- **Current coverage:** 71.7%

- **Maximum feasible coverage:** 95.8%
- **Estimated unit cost:** USD $1.40 (0.73 - 2.74)

Staple food supplementation

- **Current coverage:** 0.6%
- **Maximum feasible coverage:** 40.1%
- **Estimated unit cost:** USD $0.17 (0.11 - 1.27)

Cost-Effectiveness Analysis

The estimated cost per Year of Life Disabled (YLD) averted for each intervention in **Uganda** is given below, with interquartile ranges (IQR):

Iron supplementation (antenatal care)

- **Median Cost per YLD:** USD $1032
- **IQR:** USD $540 – >USD 100k

Iron supplementation (all women of reproductive age)

- **Median Cost per YLD:** USD $2007
- **IQR:** USD $1340 – $2800

Antenatal preventative IPTp-SP

- **Median Cost per YLD:** USD $2416
- **IQR:** USD $1758 – $3369

Staple food supplementation

- **Median Cost per YLD:** USD $1072
- **IQR:** USD $631 – $1799

Economic Considerations

Cost-effectiveness thresholds (CET, in USD per DALY averted) in **Uganda** is as follows:

- **Estimates from Pichon-Riviere et al. (Lower Bound):** USD $51

- **Estimates from Pichon-Riviere et al. (Upper Bound):** USD $172
- **CET equal to per capita GDP:** USD $1014
- **CET equal to 2.3 (LMICs) or 1.7 (HICs) times per capita GDP:** USD $2333

⬆ Back to Table of Contents

Anaemia Reduction in Ukraine

National Target

For our baseline CET = 1 x GDP per capita, the national target for anaemia in **Ukraine** should be a reduction in prevalence of **25%**. This target is estimated with a 95% uncertainty interval from **10%** to **45%**.

For CET based on Pichon-Riviere, the national target for anaemia in **Ukraine** should be a reduction in prevalence of **22%**. This target is estimated with a 95% uncertainty interval from **9%** to **40%**.

For a near-infinite CET, the national target for anaemia in **Ukraine** should be a reduction in prevalence of **32%**. This target is estimated with a 95% uncertainty interval from **18%** to **49%**.

Input parameters

- Among women of reproductive age in **Ukraine**, the prevalence of overall anaemia is **18.6%**
  - **Mild anaemia:** 9.0%
  - **Moderate anaemia:** 8.3%
  - **Severe anaemia:** 1.3%
- There is no data on malaria prevalence; assume 0%.

Intervention coverage and costs

Iron supplementation (antenatal care)

- **Current coverage:** 56.4%
- **Maximum feasible coverage:** 99.0%
- **Estimated unit cost:** USD $10.09 (5.01 - 18.10)

Iron supplementation (all women of reproductive age)

- **Current coverage:** 23.2%
- **Maximum feasible coverage:** 99.0%
- **Estimated unit cost:** USD $7.47 (3.76 - 15.49)

Antenatal preventative IPTp-SP

- **Current coverage:** 0.0%
- **Maximum feasible coverage:** 99.0%
- **Estimated unit cost:** USD $7.88 (4.02 - 14.22)

Staple food supplementation

- **Current coverage:** 1.7%
- **Maximum feasible coverage:** 90.6%
- **Estimated unit cost:** USD $0.09 (0.03 - 0.33)

Cost-Effectiveness Analysis

The estimated cost per Year of Life Disabled (YLD) averted for each intervention in **Ukraine** is given below, with interquartile ranges (IQR):

Iron supplementation (antenatal care)

- **Median Cost per YLD:** USD $3166
- **IQR:** USD $2339 – $4226

Iron supplementation (all women of reproductive age)

- **Median Cost per YLD:** USD $6160
- **IQR:** USD $4498 – $8918

Antenatal preventative IPTp-SP

- **Median Cost per YLD:** >USD 100k
- **IQR:** >USD 100k

Staple food supplementation

- **Median Cost per YLD:** USD $352

- **IQR:** USD $228 – $539

Economic Considerations

Cost-effectiveness thresholds (CET, in USD per DALY averted) in **Ukraine** is as follows:

- **Estimates from Pichon-Riviere et al. (Lower Bound):** USD $1347
- **Estimates from Pichon-Riviere et al. (Upper Bound):** USD $2694
- **CET equal to per capita GDP:** USD $5181
- **CET equal to 2.3 (LMICs) or 1.7 (HICs) times per capita GDP:** USD $11917

⬆ Back to Table of Contents

Anaemia Reduction in United Arab Emirates

National Target

For our baseline CET = 1 x GDP per capita, the national target for anaemia in **United Arab Emirates** should be a reduction in prevalence of **21%**. This target is estimated with a 95% uncertainty interval from **9%** to **36%**.

For CET based on Pichon-Riviere, the national target for anaemia in **United Arab Emirates** should be a reduction in prevalence of **15%**. This target is estimated with a 95% uncertainty interval from **5%** to **33%**.

For a near-infinite CET, the national target for anaemia in **United Arab Emirates** should be a reduction in prevalence of **22%**. This target is estimated with a 95% uncertainty interval from **12%** to **36%**.

Input parameters

- Among women of reproductive age in **United Arab Emirates**, the prevalence of overall anaemia is **50.3%**
  - **Mild anaemia:** 38.1%
  - **Moderate anaemia:** 12.0%
  - **Severe anaemia:** 0.2%
- There is no data on malaria prevalence; assume 0%.

Intervention coverage and costs

Iron supplementation (antenatal care)

- **Current coverage:** 81.0%
- **Maximum feasible coverage:** 100.0%
- **Estimated unit cost:** USD $74.41 (26.37 - 116.44)

Iron supplementation (all women of reproductive age)

- **Current coverage:** 33.4%
- **Maximum feasible coverage:** 100.0%
- **Estimated unit cost:** USD $45.19 (16.86 - 81.75)

Antenatal preventative IPTp-SP

- **Current coverage:** 0.0%
- **Maximum feasible coverage:** 100.0%
- **Estimated unit cost:** USD $26.88 (13.51 - 41.93)

Staple food supplementation

- **Current coverage:** 50.9%
- **Maximum feasible coverage:** 99.9%
- **Estimated unit cost:** USD $0.25 (0.05 - 0.68)

Cost-Effectiveness Analysis

The estimated cost per Year of Life Disabled (YLD) averted for each intervention in **United Arab Emirates** is given below, with interquartile ranges (IQR):

Iron supplementation (antenatal care)

- **Median Cost per YLD:** USD $15608
- **IQR:** USD $10460 – $24886

Iron supplementation (all women of reproductive age)

- **Median Cost per YLD:** USD $25362
- **IQR:** USD $17711 – $36383

Antenatal preventative IPTp-SP

- **Median Cost per YLD:** >USD 100k
- **IQR:** >USD 100k

Staple food supplementation

- **Median Cost per YLD:** USD $662
- **IQR:** USD $415 – $1044

Economic Considerations

Cost-effectiveness thresholds (CET, in USD per DALY averted) in **United Arab Emirates** is as follows:

- **Estimates from Pichon-Riviere et al. (Lower Bound):** USD $16953
- **Estimates from Pichon-Riviere et al. (Upper Bound):** USD $19601
- **CET equal to per capita GDP:** USD $52977
- **CET equal to 2.3 (LMICs) or 1.7 (HICs) times per capita GDP:** USD $74168

⬆ Back to Table of Contents

Anaemia Reduction in United Kingdom

National Target

For our baseline CET = 1 x GDP per capita, the national target for anaemia in **United Kingdom** should be a reduction in prevalence of **16%**. This target is estimated with a 95% uncertainty interval from **6%** to **33%**.

For CET based on Pichon-Riviere, the national target for anaemia in **United Kingdom** should be a reduction in prevalence of **16%**. This target is estimated with a 95% uncertainty interval from **6%** to **33%**.

For a near-infinite CET, the national target for anaemia in **United Kingdom** should be a reduction in prevalence of **25%**. This target is estimated with a 95% uncertainty interval from **13%** to **41%**.

Input parameters

- Among women of reproductive age in **United Kingdom**, the prevalence of overall anaemia is **8.3%**
  - **Mild anaemia:** 6.6%
  - **Moderate anaemia:** 1.6%
  - **Severe anaemia:** 0.1%
- There is no data on malaria prevalence; assume 0%.

Intervention coverage and costs

Iron supplementation (antenatal care)

- **Current coverage:** 81.0%
- **Maximum feasible coverage:** 100.0%
- **Estimated unit cost:** USD $92.67 (35.45 - 147.48)

Iron supplementation (all women of reproductive age)

- **Current coverage:** 33.4%
- **Maximum feasible coverage:** 100.0%
- **Estimated unit cost:** USD $58.22 (21.22 - 84.76)

Antenatal preventative IPTp-SP

- **Current coverage:** 0.0%
- **Maximum feasible coverage:** 100.0%
- **Estimated unit cost:** USD $43.06 (21.60 - 67.51)

Staple food supplementation

- **Current coverage:** 35.7%
- **Maximum feasible coverage:** 99.9%
- **Estimated unit cost:** USD $0.27 (0.17 - 0.42)

Cost-Effectiveness Analysis

The estimated cost per Year of Life Disabled (YLD) averted for each intervention in **United Kingdom** is given below, with interquartile ranges (IQR):

Iron supplementation (antenatal care)

- **Median Cost per YLD:** >USD 100k
- **IQR:** USD $94863 – >USD 100k

Iron supplementation (all women of reproductive age)

- **Median Cost per YLD:** >USD 100k
- **IQR:** >USD 100k

Antenatal preventative IPTp-SP

- **Median Cost per YLD:** >USD 100k
- **IQR:** >USD 100k

Staple food supplementation

- **Median Cost per YLD:** USD $3690
- **IQR:** USD $2620 – $5541

Economic Considerations

Cost-effectiveness thresholds (CET, in USD per DALY averted) in **United Kingdom** is as follows:

- **Estimates from Pichon-Riviere et al. (Lower Bound):** USD $36650
- **Estimates from Pichon-Riviere et al. (Upper Bound):** USD $43003
- **CET equal to per capita GDP:** USD $48867
- **CET equal to 2.3 (LMICs) or 1.7 (HICs) times per capita GDP:** USD $68413

⬆ Back to Table of Contents

Anaemia Reduction in United States

National Target

For our baseline CET = 1 x GDP per capita, the national target for anaemia in **United States** should be a reduction in prevalence of **11%**. This target is estimated with a 95% uncertainty interval from **2%** to **27%**.

For CET based on Pichon-Riviere, the national target for anaemia in **United States** should be a reduction in prevalence of **12%**. This target is estimated with a 95% uncertainty interval from **2%** to **30%**.

For a near-infinite CET, the national target for anaemia in **United States** should be a reduction in prevalence of **19%**. This target is estimated with a 95% uncertainty interval from **9%** to **32%**.

Input parameters

- Among women of reproductive age in **United States**, the prevalence of overall anaemia is **12.3%**
  - **Mild anaemia:** 9.1%
  - **Moderate anaemia:** 3.1%
  - **Severe anaemia:** 0.2%
- There is no data on malaria prevalence; assume 0%.

Intervention coverage and costs

Iron supplementation (antenatal care)

- **Current coverage:** 81.0%
- **Maximum feasible coverage:** 100.0%
- **Estimated unit cost:** USD $NaN (NaN - NaN)

Iron supplementation (all women of reproductive age)

- **Current coverage:** 33.4%
- **Maximum feasible coverage:** 100.0%
- **Estimated unit cost:** USD $NaN (NaN - NaN)

Antenatal preventative IPTp-SP

- **Current coverage:** 0.0%
- **Maximum feasible coverage:** 100.0%

- **Estimated unit cost:** USD $NaN (NaN - NaN)

Staple food supplementation

- **Current coverage:** 63.2%
- **Maximum feasible coverage:** 100.0%
- **Estimated unit cost:** USD $NaN (NaN - NaN)

Cost-Effectiveness Analysis

The estimated cost per Year of Life Disabled (YLD) averted for each intervention in **United States** is given below, with interquartile ranges (IQR):

Iron supplementation (antenatal care)

- **Median Cost per YLD:** USD $92178
- **IQR:** USD $64189 – >USD 100k

Iron supplementation (all women of reproductive age)

- **Median Cost per YLD:** >USD 100k
- **IQR:** USD $99357 – >USD 100k

Antenatal preventative IPTp-SP

- **Median Cost per YLD:** >USD 100k
- **IQR:** >USD 100k

Staple food supplementation

- **Median Cost per YLD:** USD $2878
- **IQR:** USD $1944 – $4443

Economic Considerations

Cost-effectiveness thresholds (CET, in USD per DALY averted) in **United States** is as follows:

- **Estimates from Pichon-Riviere et al. (Lower Bound):** USD $NaN
- **Estimates from Pichon-Riviere et al. (Upper Bound):** USD $NaN

- **CET equal to per capita GDP:** USD $NaN
- **CET equal to 2.3 (LMICs) or 1.7 (HICs) times per capita GDP:** USD $NaN

⬆ Back to Table of Contents

Anaemia Reduction in Uruguay

National Target

For our baseline CET = 1 x GDP per capita, the national target for anaemia in **Uruguay** should be a reduction in prevalence of **15%**. This target is estimated with a 95% uncertainty interval from **0%** to **29%**.

For CET based on Pichon-Riviere, the national target for anaemia in **Uruguay** should be a reduction in prevalence of **14%**. This target is estimated with a 95% uncertainty interval from **0%** to **30%**.

For a near-infinite CET, the national target for anaemia in **Uruguay** should be a reduction in prevalence of **23%**. This target is estimated with a 95% uncertainty interval from **13%** to **37%**.

Input parameters

- Among women of reproductive age in **Uruguay**, the prevalence of overall anaemia is **12.5%**
  - **Mild anaemia:** 10.9%
  - **Moderate anaemia:** 1.6%
  - **Severe anaemia:** 0.0%
- There is no data on malaria prevalence; assume 0%.

Intervention coverage and costs

Iron supplementation (antenatal care)

- **Current coverage:** 81.0%
- **Maximum feasible coverage:** 97.0%
- **Estimated unit cost:** USD $59.31 (18.81 - 95.04)

Iron supplementation (all women of reproductive age)

- **Current coverage:** 33.4%

- **Maximum feasible coverage:** 97.0%
- **Estimated unit cost:** USD $34.92 (9.54 - 48.24)

Antenatal preventative IPTp-SP

- **Current coverage:** 0.0%
- **Maximum feasible coverage:** 97.0%
- **Estimated unit cost:** USD $16.69 (8.41 - 25.95)

Staple food supplementation

- **Current coverage:** 14.1%
- **Maximum feasible coverage:** 69.1%
- **Estimated unit cost:** USD $0.77 (0.55 - 1.23)

Cost-Effectiveness Analysis

The estimated cost per Year of Life Disabled (YLD) averted for each intervention in **Uruguay** is given below, with interquartile ranges (IQR):

Iron supplementation (antenatal care)

- **Median Cost per YLD:** USD $51563
- **IQR:** USD $31675 – >USD 100k

Iron supplementation (all women of reproductive age)

- **Median Cost per YLD:** USD $65170
- **IQR:** USD $43248 – >USD 100k

Antenatal preventative IPTp-SP

- **Median Cost per YLD:** >USD 100k
- **IQR:** >USD 100k

Staple food supplementation

- **Median Cost per YLD:** USD $6060
- **IQR:** USD $3968 – $10125

Economic Considerations

Cost-effectiveness thresholds (CET, in USD per DALY averted) in **Uruguay** is as follows:

- **Estimates from Pichon-Riviere et al. (Lower Bound):** USD $15118
- **Estimates from Pichon-Riviere et al. (Upper Bound):** USD $17826
- **CET equal to per capita GDP:** USD $22565
- **CET equal to 2.3 (LMICs) or 1.7 (HICs) times per capita GDP:** USD $31590

⬆ Back to Table of Contents

Anaemia Reduction in Uzbekistan

National Target

For our baseline CET = 1 x GDP per capita, the national target for anaemia in **Uzbekistan** should be a reduction in prevalence of **30%**. This target is estimated with a 95% uncertainty interval from **15%** to **47%**.

For CET based on Pichon-Riviere, the national target for anaemia in **Uzbekistan** should be a reduction in prevalence of **23%**. This target is estimated with a 95% uncertainty interval from **10%** to **43%**.

For a near-infinite CET, the national target for anaemia in **Uzbekistan** should be a reduction in prevalence of **30%**. This target is estimated with a 95% uncertainty interval from **18%** to **48%**.

Input parameters

- Among women of reproductive age in **Uzbekistan**, the prevalence of overall anaemia is **53.0%**
    - **Mild anaemia:** 24.9%
    - **Moderate anaemia:** 25.4%
    - **Severe anaemia:** 2.7%
- The prevalence of malaria is **0.0%**.

Intervention coverage and costs

Iron supplementation (antenatal care)

- **Current coverage:** 50.2%
- **Maximum feasible coverage:** 94.4%
- **Estimated unit cost:** USD $4.08 (1.99 - 9.01)

Iron supplementation (all women of reproductive age)

- **Current coverage:** 20.7%
- **Maximum feasible coverage:** 94.4%
- **Estimated unit cost:** USD $3.56 (1.80 - 9.50)

Antenatal preventative IPTp-SP

- **Current coverage:** 0.0%
- **Maximum feasible coverage:** 94.4%
- **Estimated unit cost:** USD $2.95 (1.55 - 6.80)

Staple food supplementation

- **Current coverage:** 1.5%
- **Maximum feasible coverage:** 81.6%
- **Estimated unit cost:** USD $0.06 (0.01 - 0.18)

Cost-Effectiveness Analysis

The estimated cost per Year of Life Disabled (YLD) averted for each intervention in **Uzbekistan** is given below, with interquartile ranges (IQR):

Iron supplementation (antenatal care)

- **Median Cost per YLD:** USD $520
- **IQR:** USD $377 – $724

Iron supplementation (all women of reproductive age)

- **Median Cost per YLD:** USD $1244
- **IQR:** USD $826 – $1713

Antenatal preventative IPTp-SP

- **Median Cost per YLD:** >USD 100k
- **IQR:** >USD 100k

Staple food supplementation

- **Median Cost per YLD:** USD $60
- **IQR:** USD $38 – $92

Economic Considerations

Cost-effectiveness thresholds (CET, in USD per DALY averted) in **Uzbekistan** is as follows:

- **Estimates from Pichon-Riviere et al. (Lower Bound):** USD $524
- **Estimates from Pichon-Riviere et al. (Upper Bound):** USD $1048
- **CET equal to per capita GDP:** USD $2496
- **CET equal to 2.3 (LMICs) or 1.7 (HICs) times per capita GDP:** USD $5741

⬆ Back to Table of Contents

Anaemia Reduction in Vanuatu

National Target

For our baseline CET = 1 x GDP per capita, the national target for anaemia in **Vanuatu** should be a reduction in prevalence of **26%**. This target is estimated with a 95% uncertainty interval from **10%** to **46%**.

For CET based on Pichon-Riviere, the national target for anaemia in **Vanuatu** should be a reduction in prevalence of **18%**. This target is estimated with a 95% uncertainty interval from **0%** to **40%**.

For a near-infinite CET, the national target for anaemia in **Vanuatu** should be a reduction in prevalence of **28%**. This target is estimated with a 95% uncertainty interval from **15%** to **46%**.

Input parameters

- Among women of reproductive age in **Vanuatu**, the prevalence of overall anaemia is **51.8%**
  - **Mild anaemia:** 33.1%

    - **Moderate anaemia:** 17.8%
    - **Severe anaemia:** 0.8%
- The prevalence of malaria is **0.0%**.

Intervention coverage and costs

Iron supplementation (antenatal care)

- **Current coverage:** 84.2%
- **Maximum feasible coverage:** 89.2%
- **Estimated unit cost:** USD $6.64 (3.29 - 11.39)

Iron supplementation (all women of reproductive age)

- **Current coverage:** 34.7%
- **Maximum feasible coverage:** 84.2%
- **Estimated unit cost:** USD $6.12 (3.57 - 15.19)

Antenatal preventative IPTp-SP

- **Current coverage:** 35.0%
- **Maximum feasible coverage:** 79.2%
- **Estimated unit cost:** USD $4.45 (2.30 - 7.44)

Staple food supplementation

- **Current coverage:** 8.4%
- **Maximum feasible coverage:** 100.0%
- **Estimated unit cost:** USD $0.53 (0.08 - 1.62)

Cost-Effectiveness Analysis

The estimated cost per Year of Life Disabled (YLD) averted for each intervention in **Vanuatu** is given below, with interquartile ranges (IQR):

Iron supplementation (antenatal care)

- **Median Cost per YLD:** USD $1358

- **IQR:** USD $767 – >USD 100k

Iron supplementation (all women of reproductive age)

- **Median Cost per YLD:** USD $2795
- **IQR:** USD $1935 – $4272

Antenatal preventative IPTp-SP

- **Median Cost per YLD:** >USD 100k
- **IQR:** >USD 100k

Staple food supplementation

- **Median Cost per YLD:** USD $821
- **IQR:** USD $518 – $1282

Economic Considerations

Cost-effectiveness thresholds (CET, in USD per DALY averted) in **Vanuatu** is as follows:

- **Estimates from Pichon-Riviere et al. (Lower Bound):** USD $471
- **Estimates from Pichon-Riviere et al. (Upper Bound):** USD $909
- **CET equal to per capita GDP:** USD $3367
- **CET equal to 2.3 (LMICs) or 1.7 (HICs) times per capita GDP:** USD $7744

⬆ Back to Table of Contents

Anaemia Reduction in Venezuela

National Target

For our baseline CET = 1 x GDP per capita, the national target for anaemia in **Venezuela** should be a reduction in prevalence of **27%**. This target is estimated with a 95% uncertainty interval from **0%** to **47%**.

For CET based on Pichon-Riviere, the national target for anaemia in **Venezuela** should be a reduction in prevalence of **22%**. This target is estimated with a 95% uncertainty interval from **0%** to **46%**.

For a near-infinite CET, the national target for anaemia in **Venezuela** should be a reduction in prevalence of **29%**. This target is estimated with a 95% uncertainty interval from **16%** to **48%**.

Input parameters

- Among women of reproductive age in **Venezuela**, the prevalence of overall anaemia is **19.8%**
  - **Mild anaemia:** 15.0%
  - **Moderate anaemia:** 4.7%
  - **Severe anaemia:** 0.2%
- The prevalence of malaria is **0.2%**.

Intervention coverage and costs

Iron supplementation (antenatal care)

- **Current coverage:** 88.3%
- **Maximum feasible coverage:** 93.3%
- **Estimated unit cost:** USD $10.51 (5.20 - 17.19)

Iron supplementation (all women of reproductive age)

- **Current coverage:** 36.4%
- **Maximum feasible coverage:** 88.3%
- **Estimated unit cost:** USD $6.49 (3.74 - 15.85)

Antenatal preventative IPTp-SP

- **Current coverage:** 0.1%
- **Maximum feasible coverage:** 86.5%
- **Estimated unit cost:** USD $7.38 (3.76 - 11.89)

Staple food supplementation

- **Current coverage:** 0.9%
- **Maximum feasible coverage:** 100.0%
- **Estimated unit cost:** USD $1.52 (1.01 - 2.73)

Cost-Effectiveness Analysis

The estimated cost per Year of Life Disabled (YLD) averted for each intervention in **Venezuela** is given below, with interquartile ranges (IQR):

Iron supplementation (antenatal care)

- **Median Cost per YLD:** USD $8840
- **IQR:** USD $4591 – >USD 100k

Iron supplementation (all women of reproductive age)

- **Median Cost per YLD:** USD $11117
- **IQR:** USD $7355 – $17050

Antenatal preventative IPTp-SP

- **Median Cost per YLD:** >USD 100k
- **IQR:** >USD 100k

Staple food supplementation

- **Median Cost per YLD:** USD $7649
- **IQR:** USD $5216 – $11399

Economic Considerations

Cost-effectiveness thresholds (CET, in USD per DALY averted) in **Venezuela** is as follows:

- **Estimates from Pichon-Riviere et al. (Lower Bound):** USD $2237
- **Estimates from Pichon-Riviere et al. (Upper Bound):** USD $9266
- **CET equal to per capita GDP:** USD $15976
- **CET equal to 2.3 (LMICs) or 1.7 (HICs) times per capita GDP:** USD $36744

⬆ Back to Table of Contents

Anaemia Reduction in Vietnam

National Target

For our baseline CET = 1 x GDP per capita, the national target for anaemia in **Vietnam** should be a reduction in prevalence of **14%**. This target is estimated with a 95% uncertainty interval from **5%** to **24%**.

For CET based on Pichon-Riviere, the national target for anaemia in **Vietnam** should be a reduction in prevalence of **9%**. This target is estimated with a 95% uncertainty interval from **0%** to **23%**.

For a near-infinite CET, the national target for anaemia in **Vietnam** should be a reduction in prevalence of **15%**. This target is estimated with a 95% uncertainty interval from **7%** to **24%**.

Input parameters

- Among women of reproductive age in **Vietnam**, the prevalence of overall anaemia is **25.0%**
  - **Mild anaemia:** 12.7%
  - **Moderate anaemia:** 11.0%
  - **Severe anaemia:** 1.3%
- The prevalence of malaria is **0.0%**.

Intervention coverage and costs

Iron supplementation (antenatal care)

- **Current coverage:** 84.2%
- **Maximum feasible coverage:** 87.9%
- **Estimated unit cost:** USD $3.45 (1.68 - 6.50)

Iron supplementation (all women of reproductive age)

- **Current coverage:** 34.7%
- **Maximum feasible coverage:** 87.9%
- **Estimated unit cost:** USD $2.69 (1.36 - 6.69)

Antenatal preventative IPTp-SP

- **Current coverage:** 35.0%

- **Maximum feasible coverage:** 87.9%
- **Estimated unit cost:** USD $2.15 (1.15 - 3.99)

Staple food supplementation

- **Current coverage:** 0.8%
- **Maximum feasible coverage:** 29.6%
- **Estimated unit cost:** USD $0.67 (0.10 - 2.07)

Cost-Effectiveness Analysis

The estimated cost per Year of Life Disabled (YLD) averted for each intervention in **Vietnam** is given below, with interquartile ranges (IQR):

Iron supplementation (antenatal care)

- **Median Cost per YLD:** USD $1439
- **IQR:** USD $714 – >USD 100k

Iron supplementation (all women of reproductive age)

- **Median Cost per YLD:** USD $1944
- **IQR:** USD $1348 – $2799

Antenatal preventative IPTp-SP

- **Median Cost per YLD:** >USD 100k
- **IQR:** >USD 100k

Staple food supplementation

- **Median Cost per YLD:** USD $1544
- **IQR:** USD $981 – $2490

Economic Considerations

Cost-effectiveness thresholds (CET, in USD per DALY averted) in **Vietnam** is as follows:

- **Estimates from Pichon-Riviere et al. (Lower Bound):** USD $956

- **Estimates from Pichon-Riviere et al. (Upper Bound):** USD $1913
- **CET equal to per capita GDP:** USD $4347
- **CET equal to 2.3 (LMICs) or 1.7 (HICs) times per capita GDP:** USD $9998

⬆ Back to Table of Contents

Anaemia Reduction in Yemen

National Target

For our baseline CET = 1 x GDP per capita, the national target for anaemia in **Yemen** should be a reduction in prevalence of **9%**. This target is estimated with a 95% uncertainty interval from **1%** to **19%**.

For CET based on Pichon-Riviere, the national target for anaemia in **Yemen** should be a reduction in prevalence of **1%**. This target is estimated with a 95% uncertainty interval from **0%** to **11%**.

For a near-infinite CET, the national target for anaemia in **Yemen** should be a reduction in prevalence of **10%**. This target is estimated with a 95% uncertainty interval from **5%** to **19%**.

Input parameters

- Among women of reproductive age in **Yemen**, the prevalence of overall anaemia is **62.4%**
  - **Mild anaemia:** 18.7%
  - **Moderate anaemia:** 35.8%
  - **Severe anaemia:** 7.9%
- The prevalence of malaria is **1.7%**.

Intervention coverage and costs

Iron supplementation (antenatal care)

- **Current coverage:** NaN%
- **Maximum feasible coverage:** NaN%
- **Estimated unit cost:** USD $2.44 (1.06 - 4.70)

Iron supplementation (all women of reproductive age)

- **Current coverage:** NaN%
- **Maximum feasible coverage:** NaN%
- **Estimated unit cost:** USD $2.36 (1.19 - 6.13)

Antenatal preventative IPTp-SP

- **Current coverage:** NaN%
- **Maximum feasible coverage:** NaN%
- **Estimated unit cost:** USD $1.30 (0.70 - 2.79)

Staple food supplementation

- **Current coverage:** NaN%
- **Maximum feasible coverage:** NaN%
- **Estimated unit cost:** USD $0.16 (0.03 - 0.50)

Cost-Effectiveness Analysis

The estimated cost per Year of Life Disabled (YLD) averted for each intervention in **Yemen** is given below, with interquartile ranges (IQR):

Iron supplementation (antenatal care)

- **Median Cost per YLD:** USD $187
- **IQR:** USD $144 – $250

Iron supplementation (all women of reproductive age)

- **Median Cost per YLD:** USD $506
- **IQR:** USD $346 – $712

Antenatal preventative IPTp-SP

- **Median Cost per YLD:** USD $4981
- **IQR:** USD $3771 – $6414

Staple food supplementation

- **Median Cost per YLD:** >USD 100k

- **IQR:** USD $114 – >USD 100k

Economic Considerations

Cost-effectiveness thresholds (CET, in USD per DALY averted) in **Yemen** is as follows:

- **Estimates from Pichon-Riviere et al. (Lower Bound):** USD $143
- **Estimates from Pichon-Riviere et al. (Upper Bound):** USD $156
- **CET equal to per capita GDP:** USD $650
- **CET equal to 2.3 (LMICs) or 1.7 (HICs) times per capita GDP:** USD $1496

⬆ Back to Table of Contents

Anaemia Reduction in Zambia

National Target

For our baseline CET = 1 x GDP per capita, the national target for anaemia in **Zambia** should be a reduction in prevalence of **12%**. This target is estimated with a 95% uncertainty interval from **0%** to **26%**.

For CET based on Pichon-Riviere, the national target for anaemia in **Zambia** should be a reduction in prevalence of **0%**. This target is estimated with a 95% uncertainty interval from **0%** to **17%**.

For a near-infinite CET, the national target for anaemia in **Zambia** should be a reduction in prevalence of **16%**. This target is estimated with a 95% uncertainty interval from **8%** to **26%**.

Input parameters

- Among women of reproductive age in **Zambia**, the prevalence of overall anaemia is **41.3%**
  - **Mild anaemia:** 21.2%
  - **Moderate anaemia:** 18.6%
  - **Severe anaemia:** 1.4%
- The prevalence of malaria is **14.7%**.

Intervention coverage and costs

Iron supplementation (antenatal care)

- **Current coverage:** 97.4%
- **Maximum feasible coverage:** 100.0%
- **Estimated unit cost:** USD $2.63 (1.18 - 5.15)

Iron supplementation (all women of reproductive age)

- **Current coverage:** 40.2%
- **Maximum feasible coverage:** 97.4%
- **Estimated unit cost:** USD $2.72 (1.81 - 8.58)

Antenatal preventative IPTp-SP

- **Current coverage:** 80.8%
- **Maximum feasible coverage:** 96.6%
- **Estimated unit cost:** USD $1.33 (0.69 - 2.66)

Staple food supplementation

- **Current coverage:** 0.0%
- **Maximum feasible coverage:** 36.6%
- **Estimated unit cost:** USD $0.69 (0.12 - 1.26)

Cost-Effectiveness Analysis

The estimated cost per Year of Life Disabled (YLD) averted for each intervention in **Zambia** is given below, with interquartile ranges (IQR):

Iron supplementation (antenatal care)

- **Median Cost per YLD:** >USD 100k
- **IQR:** USD $403 – >USD 100k

Iron supplementation (all women of reproductive age)

- **Median Cost per YLD:** USD $1578
- **IQR:** USD $1048 – $2212

Antenatal preventative IPTp-SP

- **Median Cost per YLD:** USD $2291
- **IQR:** USD $1460 – >USD 100k

Staple food supplementation

- **Median Cost per YLD:** USD $825
- **IQR:** USD $553 – $1205

Economic Considerations

Cost-effectiveness thresholds (CET, in USD per DALY averted) in **Zambia** is as follows:

- **Estimates from Pichon-Riviere et al. (Lower Bound):** USD $260
- **Estimates from Pichon-Riviere et al. (Upper Bound):** USD $520
- **CET equal to per capita GDP:** USD $1369
- **CET equal to 2.3 (LMICs) or 1.7 (HICs) times per capita GDP:** USD $3149

⬆ Back to Table of Contents

Anaemia Reduction in Zimbabwe

National Target

For our baseline CET = 1 x GDP per capita, the national target for anaemia in **Zimbabwe** should be a reduction in prevalence of **11%**. This target is estimated with a 95% uncertainty interval from **0%** to **23%**.

For CET based on Pichon-Riviere, the national target for anaemia in **Zimbabwe** should be a reduction in prevalence of **1%**. This target is estimated with a 95% uncertainty interval from **0%** to **18%**.

For a near-infinite CET, the national target for anaemia in **Zimbabwe** should be a reduction in prevalence of **16%**. This target is estimated with a 95% uncertainty interval from **9%** to **26%**.

Input parameters

- Among women of reproductive age in **Zimbabwe**, the prevalence of overall anaemia is **35.0%**

  - **Mild anaemia:** 19.2%
  - **Moderate anaemia:** 14.7%
  - **Severe anaemia:** 1.2%
- The prevalence of malaria is **3.9%**.

Intervention coverage and costs

Iron supplementation (antenatal care)

- **Current coverage:** 84.9%
- **Maximum feasible coverage:** 91.4%
- **Estimated unit cost:** USD $4.12 (1.87 - 7.24)

Iron supplementation (all women of reproductive age)

- **Current coverage:** 35.0%
- **Maximum feasible coverage:** 91.4%
- **Estimated unit cost:** USD $3.70 (2.33 - 10.88)

Antenatal preventative IPTp-SP

- **Current coverage:** 7.3%
- **Maximum feasible coverage:** 91.4%
- **Estimated unit cost:** USD $2.62 (1.34 - 4.73)

Staple food supplementation

- **Current coverage:** 0.5%
- **Maximum feasible coverage:** 37.1%
- **Estimated unit cost:** USD $0.73 (0.10 - 1.22)

Cost-Effectiveness Analysis

The estimated cost per Year of Life Disabled (YLD) averted for each intervention in **Zimbabwe** is given below, with interquartile ranges (IQR):

Iron supplementation (antenatal care)

- **Median Cost per YLD:** USD $1027
- **IQR:** USD $655 – >USD 100k

Iron supplementation (all women of reproductive age)

- **Median Cost per YLD:** USD $2380
- **IQR:** USD $1668 – $3481

Antenatal preventative IPTp-SP

- **Median Cost per YLD:** USD $9228
- **IQR:** USD $7241 – $12121

Staple food supplementation

- **Median Cost per YLD:** USD $951
- **IQR:** USD $620 – $1374

Economic Considerations

Cost-effectiveness thresholds (CET, in USD per DALY averted) in **Zimbabwe** is as follows:

- **Estimates from Pichon-Riviere et al. (Lower Bound):** USD $430
- **Estimates from Pichon-Riviere et al. (Upper Bound):** USD $844
- **CET equal to per capita GDP:** USD $1592
- **CET equal to 2.3 (LMICs) or 1.7 (HICs) times per capita GDP:** USD $3663
